# Analysis of protein structures containing HEPES and MES molecules

**DOI:** 10.1002/pro.4415

**Published:** 2022-08-26

**Authors:** Joanna Magdalena Macnar, Dariusz Brzezinski, Maksymilian Chruszcz, Dominik Gront

**Affiliations:** ^1^ Department of Molecular Physiology and Biological Physics University of Virginia Charlottesville Virginia USA; ^2^ College of Inter‐Faculty Individual Studies in Mathematics and Natural Sciences University of Warsaw Warsaw Poland; ^3^ Faculty of Chemistry, Biological and Chemical Research Center University of Warsaw Warsaw Poland; ^4^ Institute of Computing Science Poznan University of Technology Poznan Poland; ^5^ Center for Biocrystallographic Research, Institute of Bioorganic Chemistry Polish Academy of Sciences Poznan Poland; ^6^ Department of Chemistry and Biochemistry University of South Carolina Columbia South Carolina USA

**Keywords:** buffer molecules, HEPES, ligand validation, MES, multiple conformations

## Abstract

X‐ray crystallography is the main experimental method behind ligand–macromolecule complexes found in the Protein Data Bank (PDB). Applying bioinformatics methods to such structural data can fuel drug discovery, albeit under the condition that the information is correct. Regrettably, a small number of structures in the PDB are of suboptimal quality due to incorrectly identified and modeled ligands in protein–ligand complexes. In this paper, we combine a theoretical‐graph approach, nuclear density estimates, bioinformatics methods, and prior chemical knowledge to analyze two non‐physiological ligands, HEPES and MES, that are frequent components of crystallization and purifications buffers. Our analysis includes quantum mechanics calculations and Cambridge Structure Database (CSD) queries to define the ideal conformation of these ligands, geometry analysis of PDB deposits regarding several quality factors, and a search for homologous structures to identify other small molecules that could bind in place of the parasitic ligand. Our results highlight the need for careful refinement of macromolecule–ligand complexes and better validation tools that integrate results from all relevant resources.

## Abstract 2

PDB Code(s): 3K4L, 3PYI, 5T6L, 6BB0, 1PJX, 3O4P, 6WCF, 3DKE, 3E10, 6G38, 4E8R, 4Z91, 3E9F, 1MOS, 1MOQ, 2ESB, 1VHR, 4P66 and 6NNI;

**Abbreviations**
CSD: Cambridge Structural Database
DFT: density functional theory
EDO: ethane‐1,2‐diol
FOL: (2S)‐2‐[[4‐[(2‐amino‐4‐oxo‐3H‐pteridin‐6‐yl)methylamino]benzoyl]amino]pentanedioic acid, folic acid
GOL: propane‐1,2,3‐triol
HEPES: 2‐[4‐(2‐hydroxyethyl)piperazin‐1‐yl]ethanesulfonic acid
KDE: kernel density estimation
MES: 2‐morpholin‐4‐ylethanesulfonic acid
MTX: (2S)‐2‐[[4‐[(2,4‐diaminopteridin‐6‐yl)methyl‐methylamino]benzoyl]amino]pentanedioic acid, methotrexate
PanDDA: Pan‐dataset density analysis
PDB: Protein Data Bank
QM: quantum mechanics
RSCC: real‐space correlation coefficient
RSR: real space *R* factor

## 1 INTRODUCTION

X‐ray crystallography is the most widely applied method for elucidating high resolution structures of proteins. The growing number of Protein Data Bank (PDB)^1^, ^2^ entries (approximately 10,000 new structures every year) gives access to untenable amounts of data that lead to a better understanding of macromolecular machinery and thus life. This rapid growth is paralleled by significant improvements in validation procedures^3^, ^4^ that have, in turn, a substantial impact on the quality of recently deposited structures. However, validating protein–ligand complexes,^5^ which correspond to 70% of X‐ray PDB entries^6^ and are an essential source of information for drug discovery,^7^ is still a significant challenge.

Small molecule ligands can be intentionally co‐crystallized with the macromolecule or soaked in after crystallization. Therefore, the binding of such small molecules to macromolecules can happen during the expression, purification, and crystallization stages. In particular, inorganic ions, such as sulfates or metal cations, are frequently present in the crystallization buffer and are often “unintentional” binding agents. On the other hand, the binding studies of organic compounds are essential for elucidating molecular mechanisms on which physiological processes depend. Therefore, the distinction between small‐molecule crystallization buffers and organic compounds during modeling can significantly impact the biological interpretation of a given structure.

The successful identification of small molecules in X‐ray structures heavily relies on the tools and methodology used during model refinement. Typically, the model building and initial refinement are performed with a macromolecular model only. The remaining unexplained electron density blobs must be identified as solvent or small‐molecule agents. The placement of water molecules is usually straightforward and can be performed almost entirely automatically by one of the available refinement programs.^8^, ^9^ The identification of ligands and their refinement is much more difficult. For high‐resolution structures, ligands are usually placed manually or with the help of one of the available ligand building tools, such as Coot,^10^ X‐LIGAND,^11^ ARP/wARP,^12^ LigandFit,^13^ or AFITT.^14^ An essential part of this process is the application of restraints to ligand geometry, which helps find the optimal compromise between the best fit into the electron density and the correct geometry of the ligand. Counterintuitively, finding the applicable agreement for lower resolution data is often a less difficult task due to the poor quality of the electron density, which makes the ligand identification an ambiguous task. Therefore, for low‐resolution data, ligand refinement is often based on expert knowledge rather than the refinement algorithm.

Unfortunately, at resolutions worse than 2.5 Å it is not always possible to distinguish between various ligands.^10^ Therefore, additional information, regarding the composition of crystallization buffers and precipitants, and the addition of co‐crystallization agents, is needed to elucidate the ligands present in the structure. Unfortunately, this information is not always available, even for the author of the deposit, as crystallization is frequently performed long before diffraction experiments and often by a different researcher. For example, it was shown in several papers^15^, ^16^ that authors associated a low‐resolution electron density blob with a functional ligand that was used during soaking instead of a HEPES molecule that was present in crystallization buffer.

**FIGURE 1:**
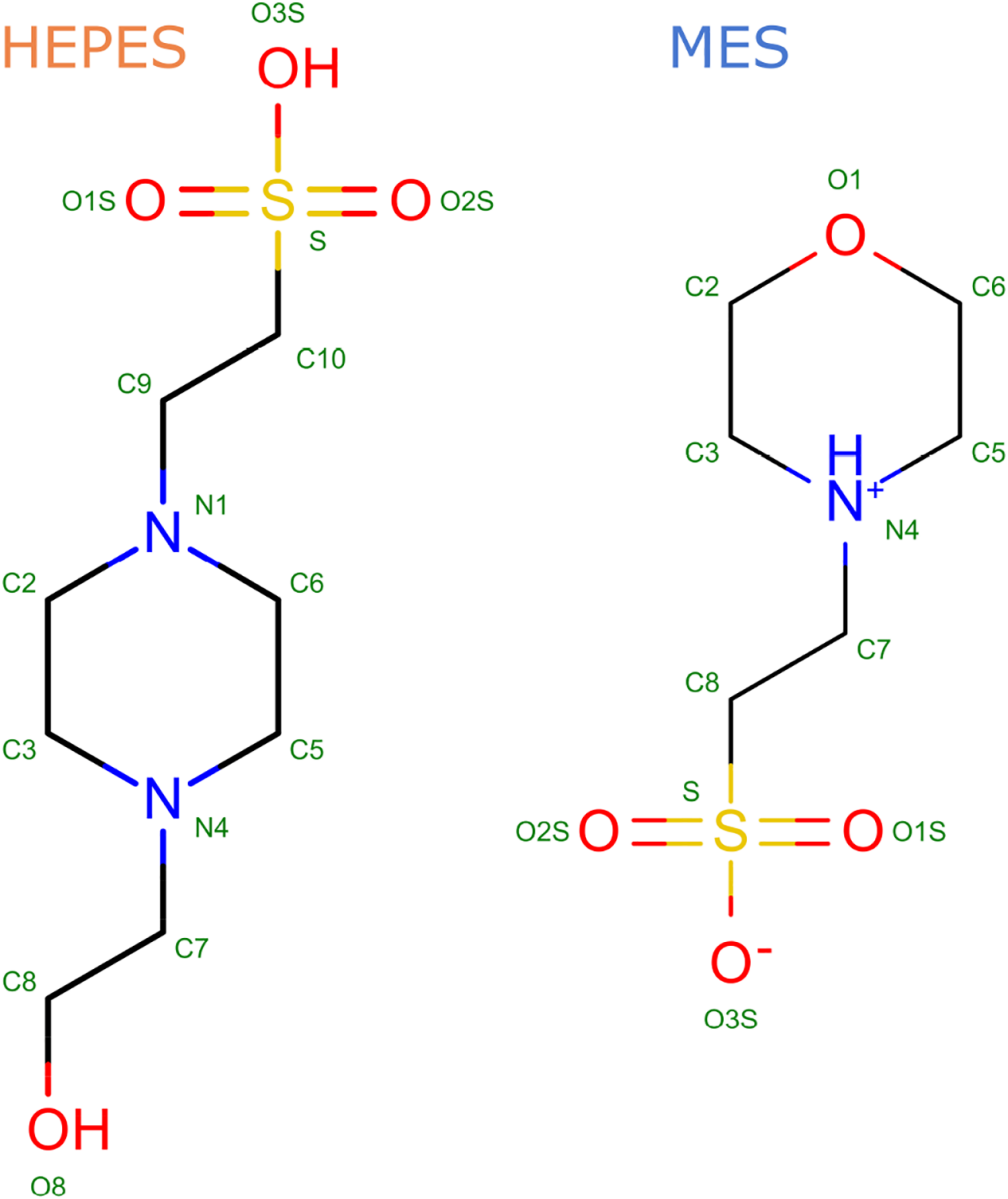
Structural formula of HEPES (left) and MES (right) molecules, atom numbers in subscript are given according to PDB^1^ nomenclature. HEPES, 2‐[4‐(2‐hydroxyethyl)piperazin‐1‐yl]ethanesulfonic acid; MES, 2‐morpholin‐4‐ylethanesulfonic acid; PDB, Protein Data Bank

In this paper, we discuss the pitfalls in modeling two commonly used buffering compounds in crystallization experiments—HEPES (EPE three‐letter code in PDB deposits) and MES. These compounds are six‐membered aliphatic heterocycles with a tail with a sulfonic group (Figure 1) and represent a larger family of crystallization buffers, including MOPS, MOPSO PIPES, POPSO, and EPPS. Using HEPES and MES as examples, we present an approach to organic small‐molecule ligand‐binding analysis and validation based on their geometry and chemical properties.

## 2 RESULTS

The validation of HEPES and MES ligands began with establishing their accurate geometric models. For this purpose, high‐resolution structures from the Cambridge Structural Database (CSD)^17^ and quantum mechanics (QM) calculations were used. With ideal structures defined, the next step of our validation consisted of several analyses of HEPES and MES molecules found in the PDB deposits to indicate possible modeling problems and their causes. To highlight the possible problems, we improved the modeling of ligands in selected structures. Finally, a closer look at the surroundings of HEPES and MES led us to investigate sets of similar structures in which different ligands were bound in analogous places.

### 2.1 Ideal geometry of HEPES and MES

Each small molecule validation procedure needs a set of reference geometries against which structural models can be compared. Such reference geometries can be either computed using QM simulations or found in ultra‐high‐resolution structures in the CSD. For the purposes of this study, we will analyze geometries obtained using both of these abovementioned sources.

The geometry of HEPES and MES molecules will be discussed in terms of their atom coordinates. Bond lengths and planar angles in all HEPES (and MES) molecules should be equal within some tolerance defined by thermal fluctuations and experimental errors. Most of the larger violations of the ideal geometry result in high energy strain, and, therefore, the only difference between conformations should be observed in torsion angles. For the HEPES molecule, up to 10 torsion angles may be defined between heavy atoms of the piperazine ring, including carbons attached directly to the two nitrogen atoms. Some of these values are redundant. For instance, only one out of three torsion angles related to the —SO_3_^−^ group is unique. The other two may be calculated assuming *sp* ^*3*^ hybridization on the S atom. There are two possible ways to describe the internal geometry of the C9 atom in HEPES: using C3‐C2‐N1‐C9 and C5‐C6‐N1‐C9 torsion angles (Figure 1). These two angles should be correlated. A similar situation occurs with C10, C7, and C8 atoms. In addition, assuming ideal geometry of bonds and angles, the six‐member piperazine ring conformations may be in either a chair or boat conformation. Hence, atoms C7 and C9 should adopt either equatorial (most likely) or axial conformation. Following the above observations, three torsion angles are crucial for the definition of HEPES/MES conformation: N1‐C2‐C3‐N4 (*t*_1_), C2‐C3‐N4‐C5 (*t*_2_), and C3‐N4‐C5‐C6 (*t*_3_) (atoms naming convention according to PDB standard, see Figure 1).

**FIGURE 2:**
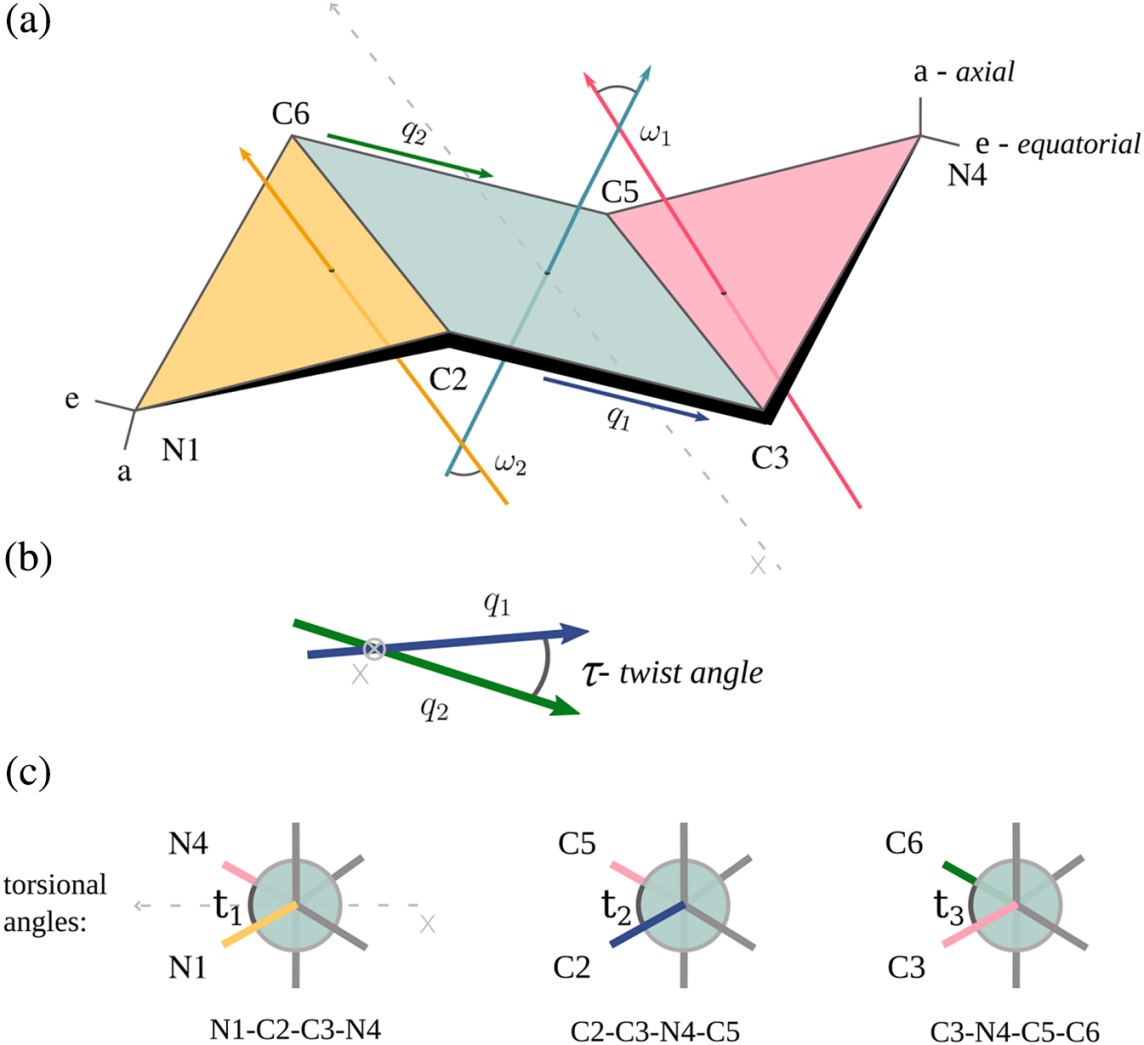
Geometric parameters of HEPES and MES. (a) Definition of the internal geometry of a six‐member ring used in this work. Two wings (pink and yellow triangles) create dihedral angles with the quadrilateral (colored in mint), denoted as *ω*_1_ or *ω*_2_. The multiplication of their values defines the chair, presented in this figure, and the boat conformation of the molecule (pink triangle turned down). Letters a and e denote axial and equatorial bonds, respectively. (b) A twist angle *τ* is described as a planar angle between vectors q_1_ (green) and q_2_ (blue), when looking along the *x*‐axis marked in dashed grey line, that corresponds to the two bonds and which are the planar part of the ring (mint quadrilateral). (c) Definitions of torsional angles: *t*_1_ between N1‐C2‐C3‐N4 atoms presented when looking along bond C2‐C3 and perpendicular to the *x*‐axis; *t*_2_ between C2‐C3‐N4‐C5 atoms presented when looking along bond C3‐N4; and *t*_3_ between C3‐N4‐C5‐C6 atoms presented when looking along bond N4‐C5. HEPES, 2‐[4‐(2‐hydroxyethyl)piperazin‐1‐yl]ethanesulfonic acid; MES, 2‐morpholin‐4‐ylethanesulfonic acid

To categorize conformations of HEPES and MES, we used two parameters: the geometry of a six‐member saturated ring and the length of bonds formed by the heterocycle. Geometry of its substituents was not taken into account because of high flexibility of these regions and the fact that it strongly depends on the ring conformation We start with a description of our approach to ring geometry validation. In the first step of the algorithm, we look for a pair of two opposing (parallel) bonds, that is, four atoms within a ring, such that a deviation from planarity is minimal (q_1_ and q_2_ in Figure 2). The deviation is measured by the planar angle between vectors corresponding to the two bonds (referred to further as a *twist angle τ*). Once the planar part of a ring has been established (mint quadrilateral in Figure 2), the two remaining atoms are considered as two *wings* (yellow and pink) attached to the quadrilateral part. Each wing creates a dihedral angle with the quadrilateral, denoted as *ω*_1_ or *ω*_2_. Using these two angles, we can classify a conformation of a ring as a *chair*, when *ω*_1_ ⋅ *ω*_2_ < 0 (which means that the two wings lie on different sides of the quadrilateral plane, as presented in Figure 2), or a *boat*, when *ω*_1_ ⋅ *ω*_2_ > 0. This approach gives us the possibility to analyze the model in respect of its current state, that does not have to be one with the lowest energy, for example when the wings' tips are formed by atoms C3 and C6.

In those two conformations, the value of twist angle *τ* is 0° for the chair and ~22° for the boat conformation. Since our definition of *ω* angles is based on the assumption that the quadrilateral part of a ring is close to planar, *ω* values are not accurate for twisted conformations, in which *τ* strongly deviates from the ideal values. Inspired by the newest small‐molecule validation protocol implemented by PDB,^4^ we choose a cutoff of 10° as a border between twisted and non‐twisted conformation. For example, when two wings form a chair in a given structure and the twist angle *τ* is 15°, the model will be classified as being in twisted chair conformation.

**FIGURE 3:**
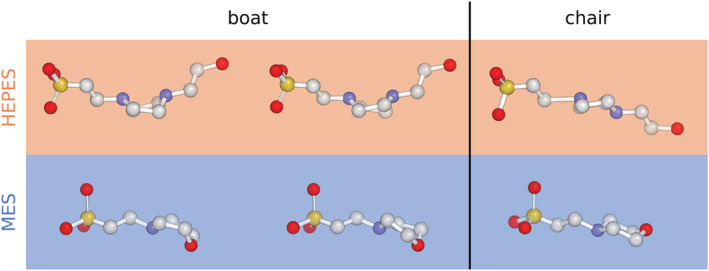
Ideal conformations of HEPES (top) and MES (bottom) in boats (left and central) and chair conformation (right) prepared using QM minimization. HEPES, 2‐[4‐(2‐hydroxyethyl)piperazin‐1‐yl]ethanesulfonic acid; MES, 2‐morpholin‐4‐ylethanesulfonic acid; QM, quantum mechanics

Visual inspection of high‐resolution structures of HEPES and MES deposited in CSD shows that all models are in a chair conformation. Also, ideal HEPES and MES ligands listed in the RCSB PDB's Ligand Expo^18^ page are in a chair conformation only. To prepare the ideal boat conformation, we performed model minimization using QM calculations based on density functional theory (DFT).^19^, ^20^ To check our approach, we also minimized the chair conformation of both ligands and validated the obtained models against those found in the CSD. Since the results were very similar (Table S1), we decided to use structures obtained via QM minimization as our standard for consistency (Figure 3).

### 2.2 Geometry of HEPES and MES in the PDB

Following the definition of ideal geometries, we analyzed HEPES and MES structures found in the PDB. In our analysis, we focused on three aspects:

- What are the differences between experimental structures found in the PDB and the ideal models?
- How common are errors of HEPES and MES conformations in the PDB?
- What are the possible causes of such errors?

#### 2.2.1 PDB versus QM

**FIGURE 4:**
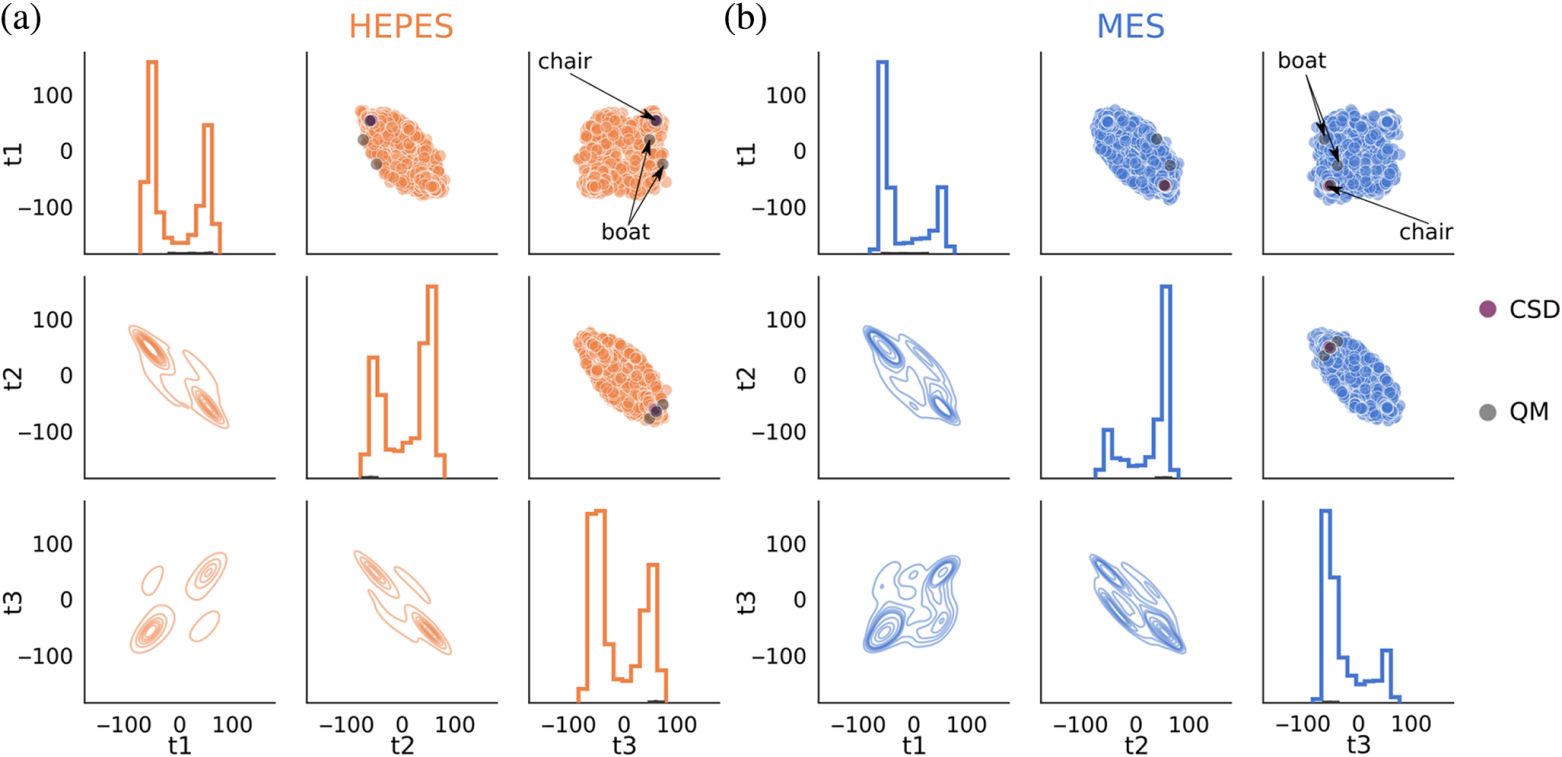
Scatter plots, KDE plots, and histograms showing three subsequent torsion angles from a six‐member ring of HEPES (a) and MES (b) found in the PDB. Results for the same ligands from CSD and QM calculations are shown in purple and dark gray, respectively. CSD and QM structures were omitted in the histograms and KDE plots. CSD, Cambridge Structural Database; HEPES, 2‐[4‐(2‐hydroxyethyl)piperazin‐1‐yl]ethanesulfonic acid; KDE, kernel density estimation; MES, 2‐morpholin‐4‐ylethanesulfonic acid; PDB, Protein Data Bank

First, we examined the torsion angles selected from the piperazine ring in HEPES: N1‐C2‐C3‐N4 (*t*_1_), C2‐C3‐N4‐C5 (*t*_2_), and C3‐N4‐C5‐C6 (*t*_3_) and corresponding atoms in MES. The scatter plots from Figure 4 show several different combinations of the torsion angles present in PDB depositions that contain HEPES and MES. Each of the three diagonal panels shows the histogram for each of the three torsion angles.

In the ideal case, there should be only one point on scatter plots for pairs of subsequent angles. For example, the ideal angle values for *t*_1_ and *t*_2_ should be around (60°, −60°), for *t*_2_ and *t*_3_ at (−60°, 60°) and *t*_1_ and *t*_3_ at (60°, 60°). These points describe a chair conformation. Observations in Figure 4 are close to the reference cases but exhibit significantly higher noise, representing structures with conformations far from the ideal. Moreover, we can observe many structures with *t*_1_, *t*_2_, and *t*_3_ equal to −60°, 60°, and −60°. These are caused by the presence of a second chair conformation, in which substituents are in axial position, but also by mirror images of the ideal chair in which the reflecting plane is placed along nitrogen atoms. To further investigate these conformations in HEPES, we calculated two more torsion angles, *t*_4_ (C2‐C3‐N4‐C7) and *t*_5_ (C3‐C2‐N1‐C9) to analyze the position of the substituents of deposits in chair conformation. In most cases, mirror reflections are present and few ligands are in the second chair conformation, but in some cases only one substituent is in axial position (Figure S1). This last group, with absolute value of *t*_4_ and *t*_5_ close to (180°, 90°) or (90°, 180°) is a set of rare outliers that is far away from the ideal geometry, but might constitute an interesting topic for future research. Boat conformations (*t*_1_ = 0°, *t*_2_ = −60°) are relatively rare (Figure 4) and, as mentioned earlier, were not present in the CSD‐derived dataset. This shows that the PDB hosts more conformers than the CSD, albeit mostly of lower quality (with potential modeling errors).

#### 2.2.2 Conformations and geometry errors

**FIGURE 5:**
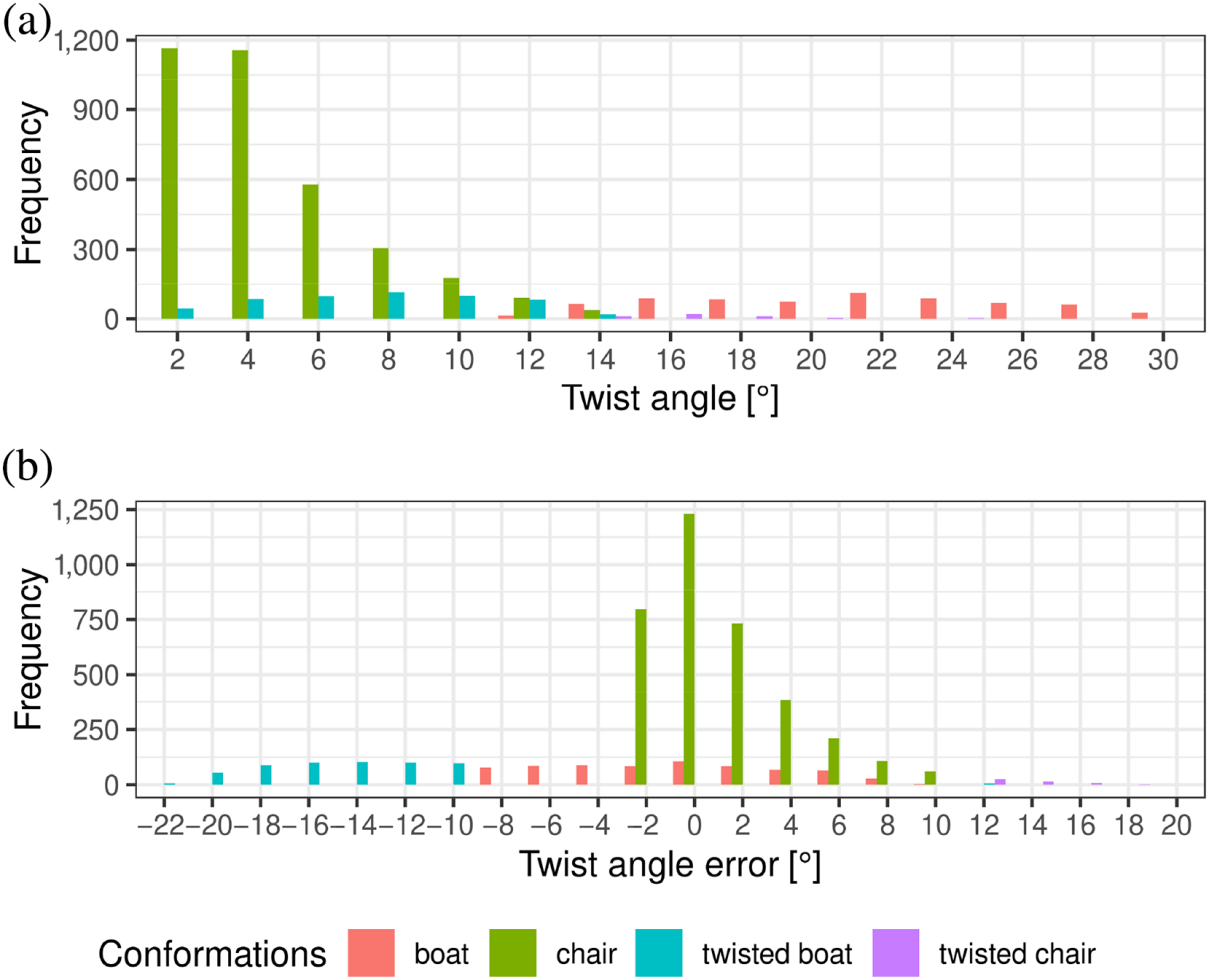
Count of twist angle (a) and twist angle error (b) distributions in PDB structures. PDB, Protein Data Bank

According to the definitions above, among 1,899 HEPES molecules in the PDB we found 184 twisted boats, 19 twisted chairs, 306 boats, and 1,390 chair conformers. The difference between the twist angle *τ* for the ideal conformation and the twisted one can be as big as 22° (Figure 5). Moreover, in some cases, *ω*_1_ or *ω*_2_ angles absolute values are within 0°–5°, which means the conformation is flat. This unusual and probably erroneous situation occurs at least in 143 structures (2.9%), out of which 49 have been classified as chairs, 22 as boats, 25 as twisted chairs, and 47 as twisted boats.

#### 2.2.3 Analysis of possible causes of the errors

**FIGURE 6:**
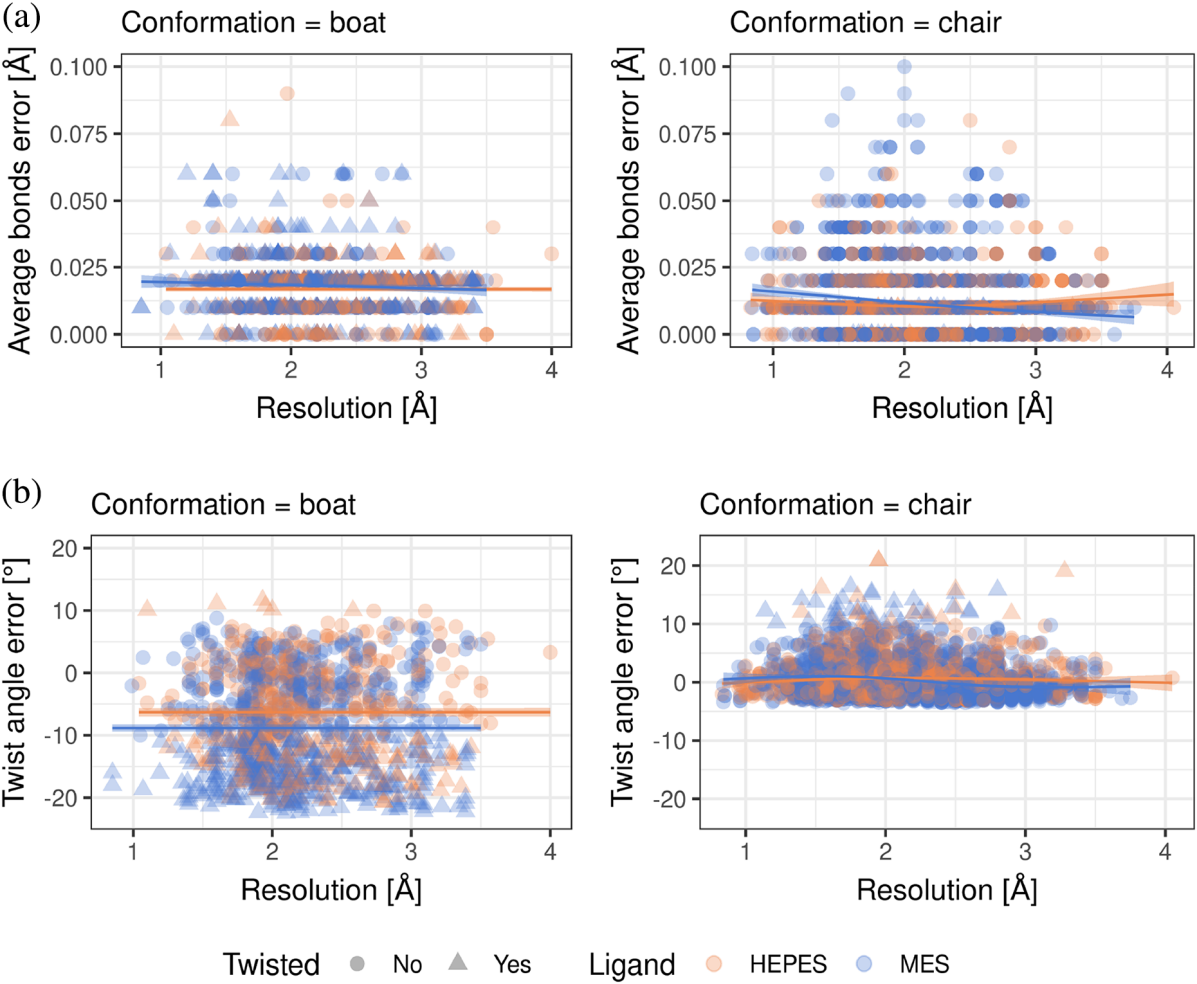
Difference in ring bond length (a) and twist angles (b), regarding ideal structures, with different resolutions. A regression model relating the *x* and *y* variables is shown as a solid line, and the confidence interval for the regression estimate is drawn using translucent bands around the regression line

Analyses of bond lengths and twist angles (Figure 6) show that, in general, there is no significant correlation between the resolution of the crystal structure and deviations of HEPES and MES covalent bond lengths from their ideal values. Moreover, the internal degrees of freedom (bond lengths, planar angles, and torsion angles) are not independent. Therefore, they cannot be considered separately. For example, one of the reasons is that a ring must be closed, and any deviation in one of its degrees of freedom must be compensated by the others.

We have also not observed a well‐pronounced correlation between the overall structure‐quality indicator, P(Q1),^21^ and the deviation from twist angles and ideal bond lengths (Figures S2 and S3). Furthermore, the correlation among the average atomic displacement parameter (ADP), traditionally called the *B*‐factor, and the twist angle error (Figure S2) was also relatively small.

To check correlation with other possible sources of twisted and suspicious conformations (bonds deviation, RSCC, RSR), we calculated Spearman's rank correlation coefficient (Figure S4). We did not find any new relationships between measured values and calculated geometry properties, with Spearman's correlation ranging from −0.16 to 0.1. Finally, we inspected whether structure genomics (SG) structures are better than non‐SG structures. Indeed, SG structures were better; however, they were not free from serious deviations (Figures S5 and S6).

The results presented above suggest that deviations from the ideal structures found in the PDB are systematic errors. They could be induced by using refining software that is unaware of heterocyclic rings in conformations other than a chair and boat in the minimal energy state and treats HEPES and MES as regular cyclohexane. When the resolution decreases, the ligand restraints start to outweigh all the deviations induced in preliminary model. This hypothesis is somewhat confirmed by the differences in average errors for chair and boat confirmations. Ligands in chair conformation are modeled with much lower errors, possibly due to the fact that the ideal geometry for HEPES and MES in databases such as RCSB PDB's Ligand Expo is only available for the chair conformation.

### 2.3 Case study: refinements of selected structures with twisted HEPES and MES

To highlight the importance of correct conformation assignment, we decided to find the twisted ligands from our study and improve them by correcting the HEPES or MES conformation and then enhancing the rest of the model. The re‐refinements were first performed for the most twisted ligands in the entire PDB and then for selected high‐resolution and low‐resolution structures with twisted ligands.

As the representatives of the twisted chair structures, we chose deposits with PDB IDs 3K4L, in which MES 902 from chain B deviated most from the ideal chair, and 6BB0, with twisted EPE 802 from chain B. Because EPE from chain C from 6BB0 was also twisted, we decided to refine it as well. Twisted boats were represented by a structure with PDB IDs 3PYI, in which MES 170 from chain B was not ideal, and 5T6L with twisted EPE 301 from chain L. Following the procedure described in Section 4.2, we showed that a non‐twisted chair fits the given density and that the overall structure can be improved (Table 1). In the case of 3PYI MES, both chair and boat fit to the density. In 5T6L only sulfonic and hydroxyl groups of HEPES appear to be ordered, while inspection of the electron density indicates that the middle part of the molecule is disordered and cannot be modeled unambiguously. Also, in chain A of 3PYI, one MES molecule can be indicated in the symmetrical place to the MES in chain B. The original and refined structures can be interactively compared using molstack^22^: https://molstack.bioreproducibility.org/collection/view/vOcU8V6WIXDPrbS9Mmcu/.

**TABLE 1:** Results of twisted chairs and twisted boat refinements calculated by HKL 3000

PDB ID	Resolution [Å]	Refinement	Conformation	*R* factor	*R* free	RSCC for ligand	MolProbity score
3K4L	1.75	Original	Twisted chair	0.183	0.241	0.914	1.38
		Ligand refinement	Chair	0.178	0.210	0.908	1.38
		Full refinement	Chair	**0.173**	**0.201**	**0.916**	**1.00**
3PYI	2.1	Original	Twisted boat	0.220	0.260	0.608	1.32
		Ligand refinement	Boat	0.222	0.259	0.535	1.32
		Full refinement	Chair	**0.205**	**0.262**	0.606	**1.03**
5T6L	2.1	Original	Twisted boat	0.203	0.234	0.911	1.98
		Ligand refinement	Boat	0.197	**0.230**	**0.926**	1.98
		Full refinement	Chair	**0.189**	0.242	0.921	**1.13**
6BB0	1.95	Original	Twisted chair Twisted boat	0.166	0.215	B: 0.942 C: 0.899	0.95
		Ligand refinement	Chair Chair	0.169	0.212	B: **0.931** C: 0.783	**0.95**
		Full refinement	Chair Chair	**0.162**	**0.205**	B: 0.926 C: **0.867**	1.24
3O4P	0.85	Original	Twisted boat	0.114	0.128	0.871	2.30
		Ligand refinement	Boat	0.123	0.136	0.817	2.29
		Full refinement	Boat	**0.119**	**0.128**	**0.889**	**2.17**
6WCF	1.06	Original	Twisted boat	0.139	0.164	0.930	1.20
		Ligand refinement	Boat	0.135	0.161	0.901	**1.20**
		Full refinement	Chair	**0.134**	**0.160**	**0.920**	1.28
3DKE	1.25	Original	Twisted boat	0.178	0.193	0.744	1.30
		Ligand refinement	Boat	0.169	**0.174**	**0.822**	**1.17**
		Full refinement	Boat	**0.161**	0.177	0.817	1.18
3E10	1.40	Original	Twist chair	0.154	0.154	0.968	1.30
		Ligand refinement	Chair	0.153	**0.157**	**0.971**	1.32
		Protein refinement	Chair	**0.149**	0.166	**0.971**	**1.27**
6G38	1.47	Original	Twisted boat	0.170	0.180	0.671	0.64
		Ligand refinement	Boat Chair	0.169	0.180	0.758 0.781	**0.64**
		Full refinement	Chair	**0.166**	**0.180**	**0.784**	0.79
4E8R ^a^	3.36	Original	Twisted boat	0.217	0.271	0.945	3.12
		Ligand refinement	Chair	0.161	**0.169**	0.835	3.12
		Full refinement	Chair	**0.160**	0.170	**0.864**	**2.56**
4Z91	3.39	Original	Twisted boat	0.231	0.261	0.794	2.40
		Ligand refinement	Boat	0.207	0.256	**0.781**	**2.40**
		Full refinement	Boat	**0.186**	**0.251**	0.698	2.57
3E9F	1.80	Original	Chair	0.188	0.220	0.96	1.46
		Ligand refinement	Chair	0.197	**0.180**	**0.97**	1.46
		Full refinement	Chair	**0.175**	0.193	**0.97**	**1.16**
1MOS	2.00	Original	Chair	0.213	0.229	0.97	1.82
		Ligand refinement	Chair	0.205	0.245	0.97	1.33
		Full refinement	Chair	**0.205**	**0.243**	**0.97**	**1.27**
1MOQ	1.57	Original	Chair	0.138	0.135	0.97	1.31
		Ligand refinement	Chair	0.133	**0.140**	**0.99**	1.29
		Full refinement	Chair	**0.121**	0.156	**0.99**	**1.26**

*Note*: Values for original structures are provided after 0 cycles of refinement using HKL 3000. The best values from re‐refinements are highlighted with bold. Order of entries follows the discussion in the text.

^a^ RNA structure.

Interestingly, local ligand refinement showed that, in some cases, twisted conformation could be improved by using newer software with basic restraints. Nevertheless, in most cases, introducing the ideal conformation and minor manual corrections improved the results. What worried us was that refinement programs could also eliminate conformational twists but leave the ligand beyond the global ring minimum.

The next group of our closer investigation was high‐resolution (0.86–1.47 Å) structures with highly twisted conformation. In the case of MES, we found more structures with high discrepancies. For HEPES, the highest difference in twist error was still very close to our 10° cutoff, reaching 12° deviation. In all cases, we were able to change the twisted conformation into a regular one with a good fit to the density (Table 1). During refinements of deposits with PDB IDs 3O4P and 1PJX, we noticed that both structures probably used the same model for molecular replacement since MES was identically twisted and other Molprobity errors were the same for those two structures. The authors of 3O4P confirmed that they used 1PJX as an input for their hydrogen refinements. Closer inspection and validation with CheckMyBlob^16^ suggested that it is possible that places where MES was placed could be occupied by a different ligand or water. Our refinement did not give unequivocal results which can suggest that part of the crystal contains MES and partly something else. Since authors of 1PJX could not be reached, only statistics of MES refinements in 3O4P are presented in Table 1. For comparison, we also refined two low‐resolution structures (Table 1). Again, we were able to correct the conformations of our ligands of interest.

The last group for closer inspection was composed of structures in which the ligand has *ω*_1_ and *ω*_2_ within the range [−5°; 5°]. We found three deposits with MES and no HEPES representative that fit to our boundaries. Also in this case we noticed errors from a model used for molecular replacement. Structure 1MOS was solved using 1MOQ as a reference. Refinements show that even basic local recalculations using current software change the “flat” ring to the chair conformation (Table 1).

**FIGURE 7:**
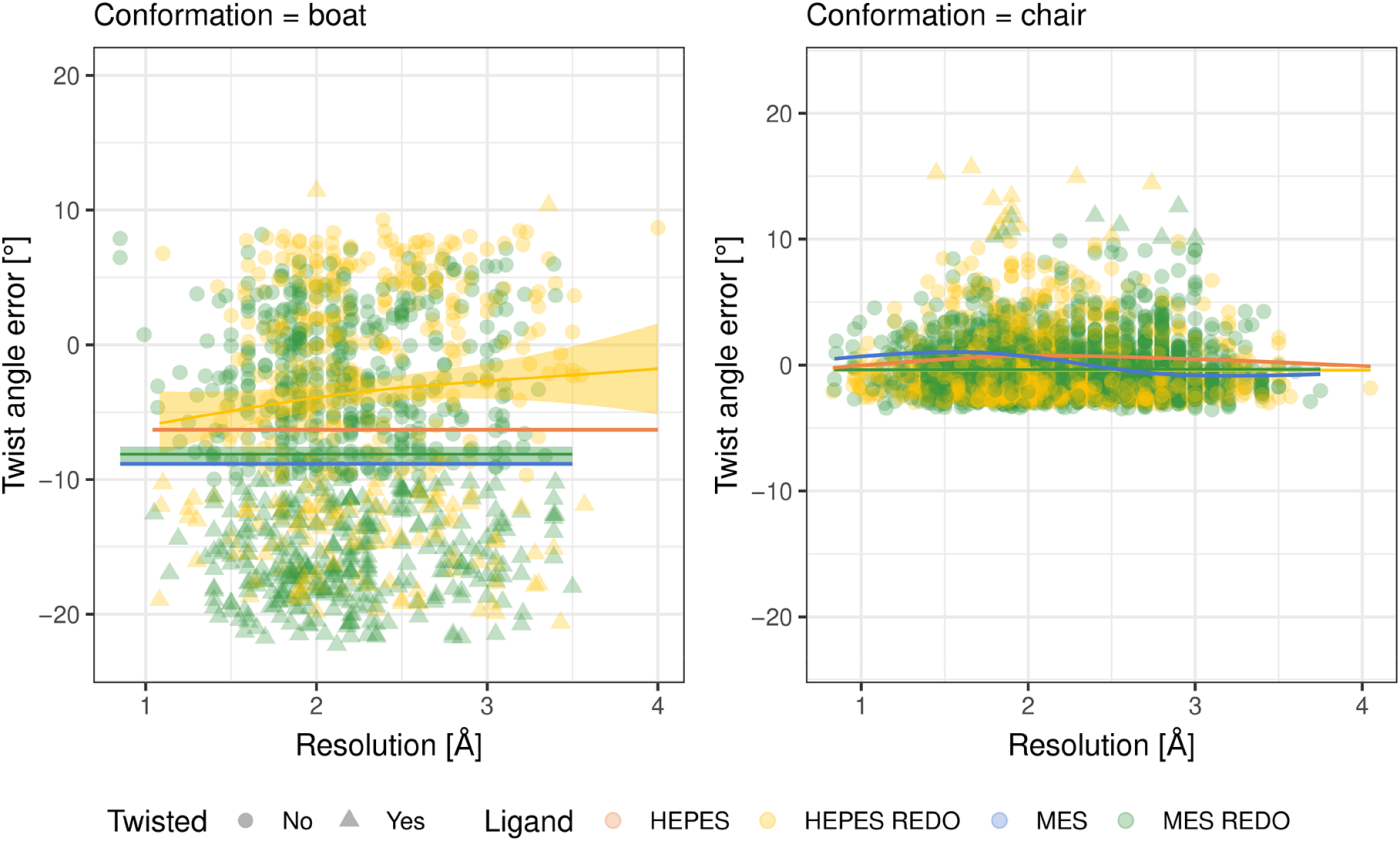
The deviation of twist angles from their ideal values in regard to the resolution of models deposited in PDB_REDO. A regression model relating the *x* and *y* variables is shown as a solid line, the confidence interval for the regression estimate is drawn using translucent bands around the regression line. Also regression models for HEPES and MES from rcsb.org are drawn with orange and blue, respectively. HEPES, 2‐[4‐(2‐hydroxyethyl)piperazin‐1‐yl]ethanesulfonic acid; MES, 2‐morpholin‐4‐ylethanesulfonic acid

Encouraged by these results, we decided to check if automatic refinement could address some of the twisted conformations. To verify this hypothesis, we performed the same analysis as made for PDB on the same dataset from PDB_REDO.^23^ We noticed that many structures have a lower deviation from the ideal value of the twist angle (Figure 7). Also, some of the extreme values of the torsional angles that were present in the PDB structures, are no longer observed in the re‐refined ones (Figure S7).

### 2.4 Ligands substituted by HEPES and MES

A quick glimpse at the PDB database shows that the HEPES molecule is present in many deposits. HEPES is a component of many buffers; its two polar tails and two nitrogen atoms could form favorable interactions with protein sidechains. In this work, we also checked if the HEPES molecule may create an interaction pattern similar to the one created by a biologically relevant agent.

The searching procedure, that included sequence similarity search and local spatial comparisons, found 277,945 protein pairs (23.9% of all pairs considered) that shared a similar spatial local neighborhood (e.g., a cavity or a pocket) where a HEPES or MES agent was located in one protein of a pair and another small molecule in a similar location of the second protein. In the remaining 76.1% of cases, there was no ligand corresponding to HEPES or MES agent within a 5 Å radius. However, this generous cutoff does not guarantee that the two small molecules share a similar interaction pattern and most likely results in a considerable fraction of false‐positive hits. In further analyses, we considered only these pairs where HEPES or MES and other ligands were no further than 0.5 Å away, giving a high chance that the two small molecules share chemical interactions. The decreased cutoff value reduced the number of hits to 70,547 pairs.

**FIGURE 8:**
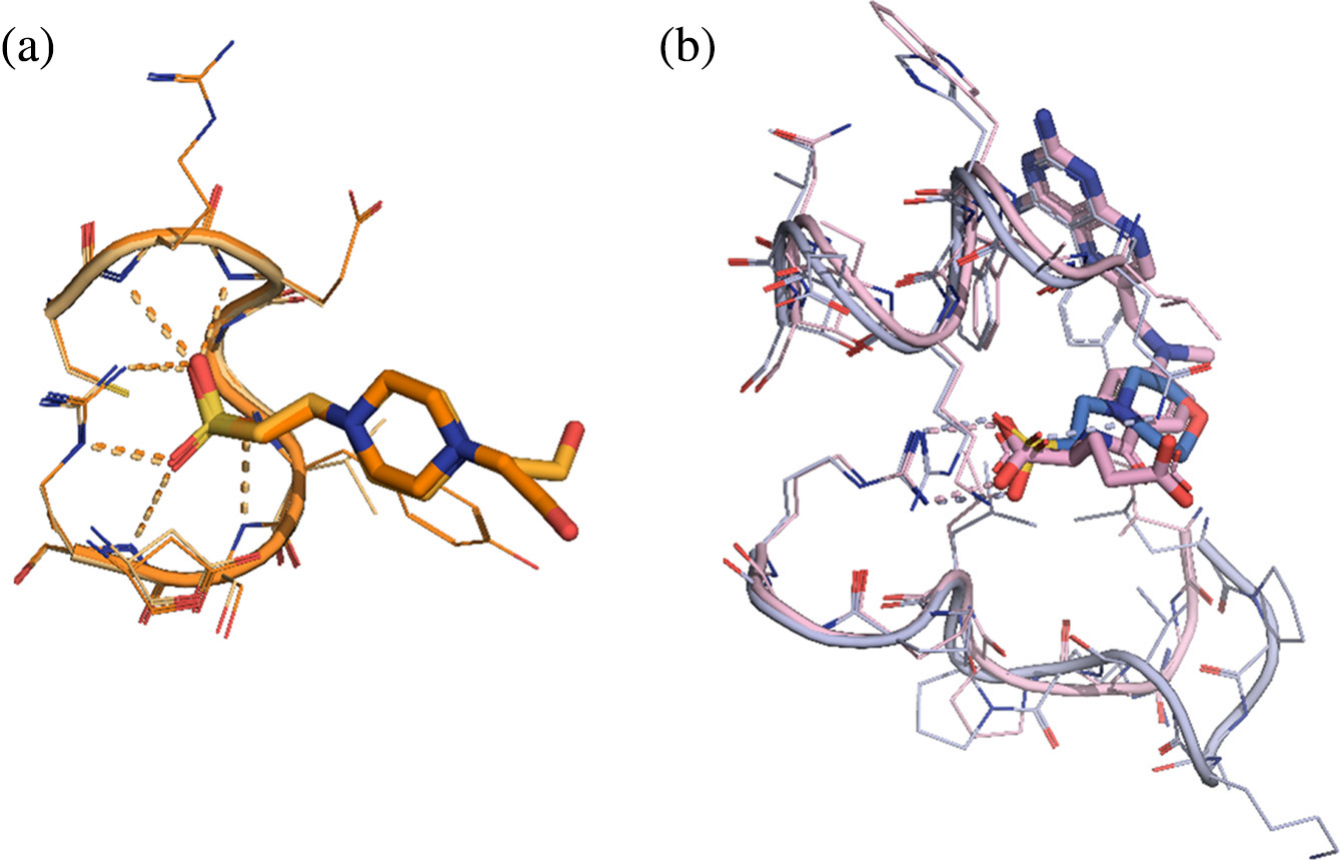
(a) HEPES molecule found in PDB ID 2ESB (light orange) crystal structure interacts with a protein neighborhood identically as HEPES found in PDB ID 1VHR (dark orange) protein. (b) MTX and MES molecules are bound in the respective parts of PDB ID 4P66 (blue) and PDB ID 6NNI (pink). Protein–ligand polar interactions are marked with thin dashed lines. HEPES, 2‐[4‐(2‐hydroxyethyl)piperazin‐1‐yl]ethanesulfonic acid; MES, 2‐morpholin‐4‐ylethanesulfonic acid; MTX, methotrexate; PDB, Protein Data Bank

The 30 most frequently occurring small molecules that HEPES or MES agents can substitute are listed in Table S1. The list corresponds to the 0.5 Å radius described above. However, analogous tables created for 0.25, 1.0, and 1.5 distances listed mostly the same compounds. The most popular were HEPES and MES. For a rather straightforward conclusion, a protein similar to the one crystallized with HEPES will also bind HEPES. An example is shown in Figure 8a, which shows HEPES molecules found in 2ESB and 1VHR. Both proteins belong to the tyrosine phosphatase family; however, they share only 24% identical residues. The first of these structures was solved with molecular replacement using the second one. Despite low sequence identity, HEPES molecule interactions are nearly identical between the two deposits.

The list in Table S1 contains also other agents used in crystallization buffers, such as glycerol or inorganic ions. Among biologically relevant ligands one can find coenzyme A (COA) and acetyl coenzyme A (CAA), folic acid and its derivative (FOL, MTX), and heme (HEM). An example is given in Figure 8b **—**a cavity from the structure deposited under PDB ID 6NNI ^24^ (containing MES molecule) has been superimposed on a respective part of the structure with PDB ID 4P66 ^25^ that binds methotrexate (MTX). The sequence identity between the two proteins (both dihydrofolate reductases) is 32.8%. The ligand‐binding pattern in these two cases is very similar, with one of the carboxyl groups acting as a sulfonic group. This shows that HEPES can be misidentified as a biologically relevant ligand, and instances of such cases have already been reported.^15^ Moreover, HEPES has also been shown to bind in the enzyme active site acting as an inhibitor.^26^

## 3 DISCUSSION AND CONCLUSIONS

HEPES and MES are biologically irrelevant ligands commonly found in crystallization buffers. These small molecules bind in an unspecific way to the protein, which opens a possibility for higher mobility of these agents in the crystal structure and could render them more difficult to refine. Indeed, frequently multiple conformers are observed in the density of a single structure. Moreover, as this study shows, many instances of HEPES and MES in the PDB deviate from the ideal chair and boat conformations. Especially in structures with lower resolution, conformations other than chair can be observed even when no protein‐ligand interactions stabilizing this uncommon and energetically not‐favorable shape are present. A more detailed inspection shows that some researchers put a HEPES molecule in its ideal geometry, selecting the conformer that fits best to the observed blob of electron density. In other cases, the authors apply unrestrained (or loosely restrained) refinement, resulting in molecule distortions. Moreover, standard sets of restraints do not force the proper geometry of a six‐member ring, which in many cases results in a relatively high twist angle value. Our study shows that these errors are not strongly connected with the resolution, temperature, and similar structural factors but most probably is the result of what was seen by the author of the model. The presented case study proves that there is still space for improvement in strange ligand conformations and that the discussed problem is not only a theoretical issue. Non‐specific binding allows for greater mobility of aliphatic chains in HEPES and MES. Therefore, many HEPES and MES conformations in PDB files are averaged over multiple conformers.

Our findings highlight the need for proper restraints during ligand refinement. In the case of HEPES and MES, such restraints could be obtained through quantum mechanics calculations and partially through the CSD. Importantly, alternative conformations of ligands should be provided as the ideal ones in the PDB and refinement software. Otherwise, inexperienced crystallographers guided by refinement software and PDB validation reports may steer toward suboptimal refinements. Therefore, there is a need for validation analyses similar to the one presented in this paper to highlight the need for multiple conformation restraints for ligands that would help avoid the presence of discussed molecular errors in the PDB.

## 4 MATERIALS AND METHODS

### 4.1 Searching for ideal geometry of HEPES and MES

As a reference set, five high‐resolution HEPES models and three high‐resolution MES models from the CSD^17^ were used for conformer comparisons (Table S2).

The ideal ligands from the PDB in the chair conformation, together with structures from CSD, were used as a benchmark for the models minimized using QM calculations based on density functional theory (DFT).^20^ The quantum chemical geometry optimizations were obtained by calculations using Spartan'10.^27^ Geometry optimization was performed using B3LYP functional^28^ and 6–311++G(2df,2p) basis set.

### 4.2 Analysis of HEPES and MES conformations in PDB

As of June 22, 2021, 2,115 HEPES and 3,112 MES molecules were found in 1,158 and 1,633 protein structures deposited to the PDB. HEPES and MES molecules, together with a vicinity of 5 Å radius, were automatically extracted from PDB models released up to that day. The *core::chemical* module from BioShell 3.0 software package^29^ has been used for this task. All structures solved using the PanDDA^30^ method were rejected. The incomplete HEPES and MES molecules were discarded; only the first variant was included in our analysis when two or more conformations were available. Structural properties (bond lengths, planar, and torsion angles) were calculated for each HEPES and MES structure. After rejecting incomplete and PanDDA structures, our analysis included 1,899 HEPES molecules from 1,048 protein structures. Similarly, we retrieved 3,043 MES molecules from 1,592 protein structures (Table S2).

Presumably, the geometry of a HEPES molecule is the result of two factors: the quality of experimental data such as resolution and restraints imposed in the refinement step. To inspect these factors, we compared geometrical properties (measured as the deviation from ideal bond lengths and planar angles of HEPES or MES) regarding the resolution and an overall structure–quality indicator, P(Q1).^21^ Also, assuming that the local quality of experimental data and local model agreement with it is reflected in temperature factors, we verified if there is a correlation between the average temperature factor for the analyzed molecules and their geometry.

### 4.3 Refinement of HEPES and MES conformations

To further investigate errors in HEPES and MES conformations, we chose two structures from each compound that deviated the most from the ideal conformation of a boat and a chair. In our procedure, we first briefly refined only ligands of interest from the structures using HKL 3000^31^ and Coot^10^ to check if the original state will improve when recent tools were used. Then, the ligands of our interest were manually refined. When the new conformation was confirmed to fit the density, the rest of the model was also manually improved following best practices.^32^ Finally, a careful check of the final structures was done using Molprobity.^33^ If the resulting ligand conformations and corresponding density looked suspicious, we used the CheckMyBlob web‐server^16^, ^34^ to check the possibility of HEPES or MES being substituted by other molecules.

The final 3K4L model was refined using 10 TLS groups as in the original structure. Also, during the final refinements of 3PYI, one NCS pair was used together with four TLS groups when the structure from PDB was prepared with one pair of NCS and two TLS groups.

### 4.4 Ligands substituted by HEPES

To find other ligands that share similar bonding patterns with HEPES, for each such protein we looked for other chains that also bind a ligand with similar amino acid arrangements in the same ligand‐binding region. We started from the 1,860 protein chains that bind HEPES and the 2,838 that bind MES. These sequences were used as queries for the PSI‐BLAST program^35^ employed to search for similar proteins in PDB. PSI‐BLAST returned many hits; each query‐hit pair was analyzed independently. A global sequence alignment was calculated for each pair, and a reference set of residue pairs was established. Such a set of residue pairs consisted of all residues of a query chain in atomic contacts with HEPES or MES paired with residues of a hit protein according to the alignment. Sets of less than three residue pairs were discarded. A rigid transformation has been established^36^ to superimpose query C‐alpha atoms on the respective hit C‐alpha atoms. Finally, the transformation was applied to a query HEPES or MES molecule, which effectively places that agent into the relevant region in a hit protein structure. Sequence alignments and spatial comparisons were calculated with the BioShell package.

## AUTHOR CONTRIBUTIONS

**Joanna Magdalena Macnar:** Conceptualization (equal); data curation (lead); formal analysis (lead); funding acquisition (equal); investigation (lead); methodology (equal); software (equal); visualization (lead); writing – review and editing (lead). **Dariusz Brzezinski:** Methodology (equal); visualization (supporting); writing – review and editing (equal). **Maksymilian Chruszcz:** Conceptualization (supporting); writing – original draft (supporting); writing – review and editing (supporting). **Dominik Gront:** Conceptualization (equal); formal analysis (supporting); funding acquisition (equal); methodology (equal); writing – original draft (lead); writing – review and editing (equal).

## CONFLICT OF INTEREST

The authors declare no potential conflict of interest.

## Supporting information


**Table S1** Supporting Information

**Table S2** Supporting Information

**Appendix S1** Supporting Information

**Appendix S2** Supporting Information

## Data Availability

Data available in article supplementary materials.
