# Supplementary material for: Analysis of protein structures containing HEPES and MES molecules
Source: Protein Sci. 2022 Aug 26;31(9):e4415. doi: 10.1002/pro.4415 (PMC9601878; doi:10.1002/pro.4415)
Supplement: Supplementary file 3 — Appendix S1 Supporting Information [file PRO-31-e4415-s004.pdf]

Analysis of protein structures containing HEPES and MES molecules

Joanna M. Macnar [1, 2, 3,*], Dariusz Brzezinski [1, 4, 5], Maksymilian Chruszcz[6], Dominik Gront [1,3]

[1]Department of Molecular Physiology and Biological Physics, University of Virginia, Charlottesville, VA 22901, USA
[2] College of Inter-Faculty Individual Studies in Mathematics and Natural Sciences, University of Warsaw, Stefana Banacha 2C, 02-097 Warsaw, Poland
[3] Faculty of Chemistry, Biological and Chemical Research Center, University of Warsaw, Pasteura 1, 02-093 Warsaw, Poland
[4] Institute of Computing Science, Poznan University of Technology, ul. Piotrowo 2, 60-965 Poznan, Poland
[5] Center for Biocrystallographic Research, Institute of Bioorganic Chemistry, Polish Academy of Sciences, Noskowskiego 12/14, 61-704 Poznan, Poland
[6] Department of Chemistry and Biochemistry, University of South Carolina, Columbia, SC 29208, USA
*corresponding author, mailing address: College of Inter-Faculty Individual Studies in Mathematics and Natural Sciences, University of Warsaw, Stefana Banacha 2C, 02-097 Warsaw, Poland;
Email: joanna.macnar @student.uw.edu.pl

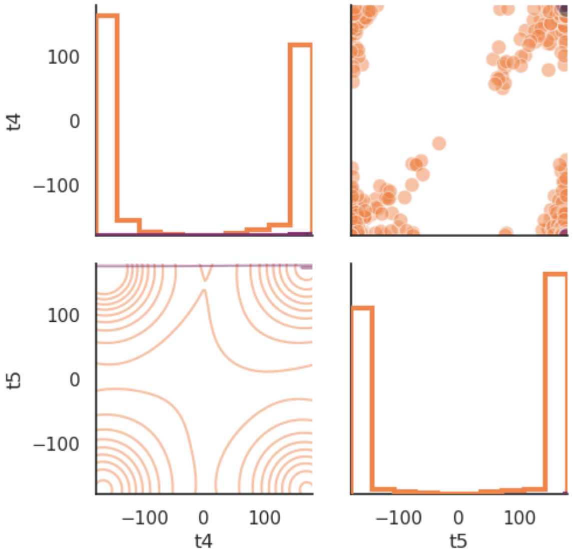

SI Figure 1 Scatter plots, kernel density estimation (KDE) plots, and histograms showing two torsion angles formed by the HEPES ring and its substituents in chair conformations. Structures from CSD and QM calculations model are shown in purple and dark gray, respectively. CSD and QM structures were omitted in the histograms and KDE plots.

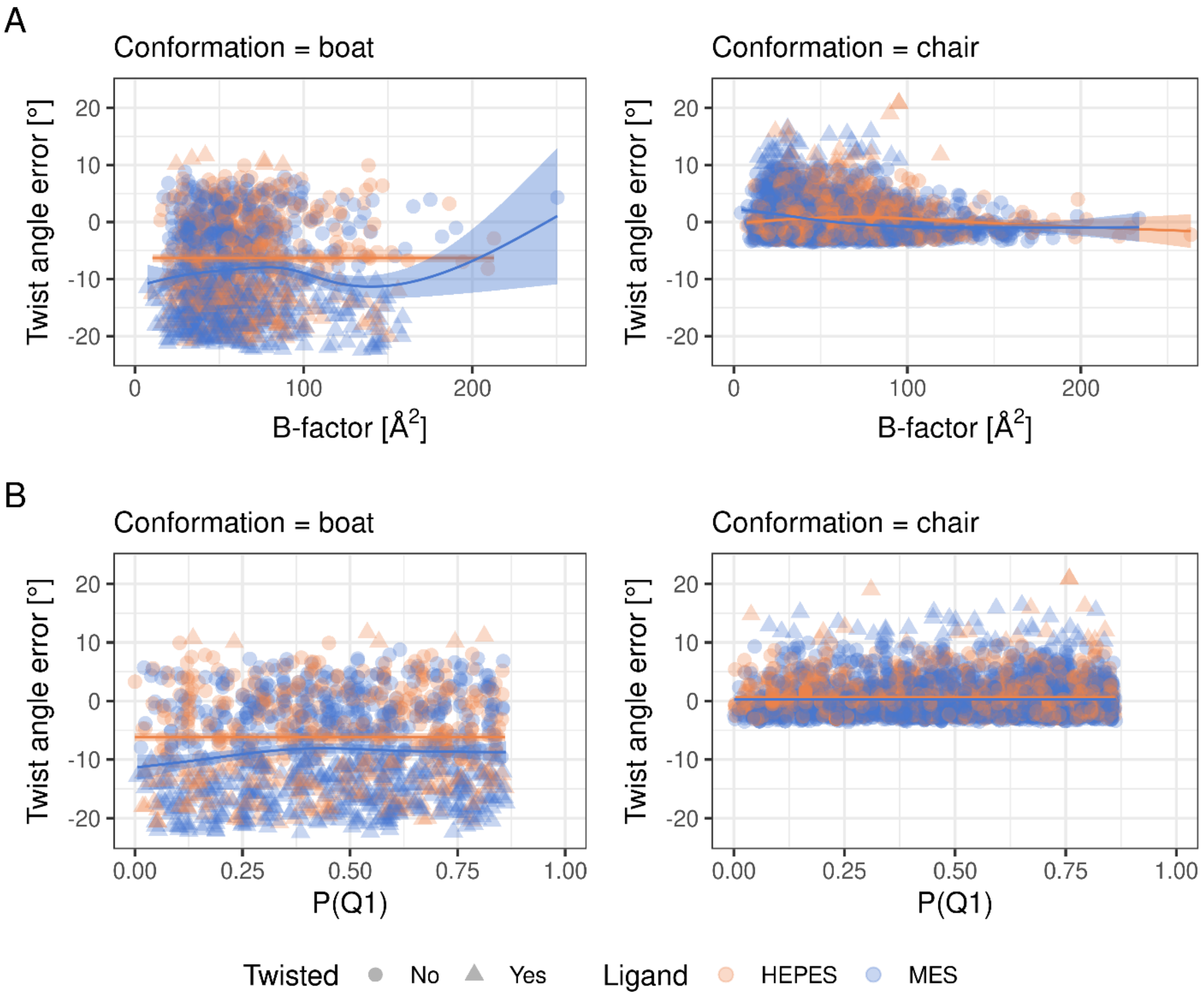

SI Figure 2 The average deviation of twist angle from their ideal values in regards to the average B-factor of ligand atoms (A), or an overall structure-quality indicator - P(Q1)(B)). A regression model relating the x and y variables is shown as a solid line, and the confidence interval for the regression estimate is drawn using translucent bands around the regression line

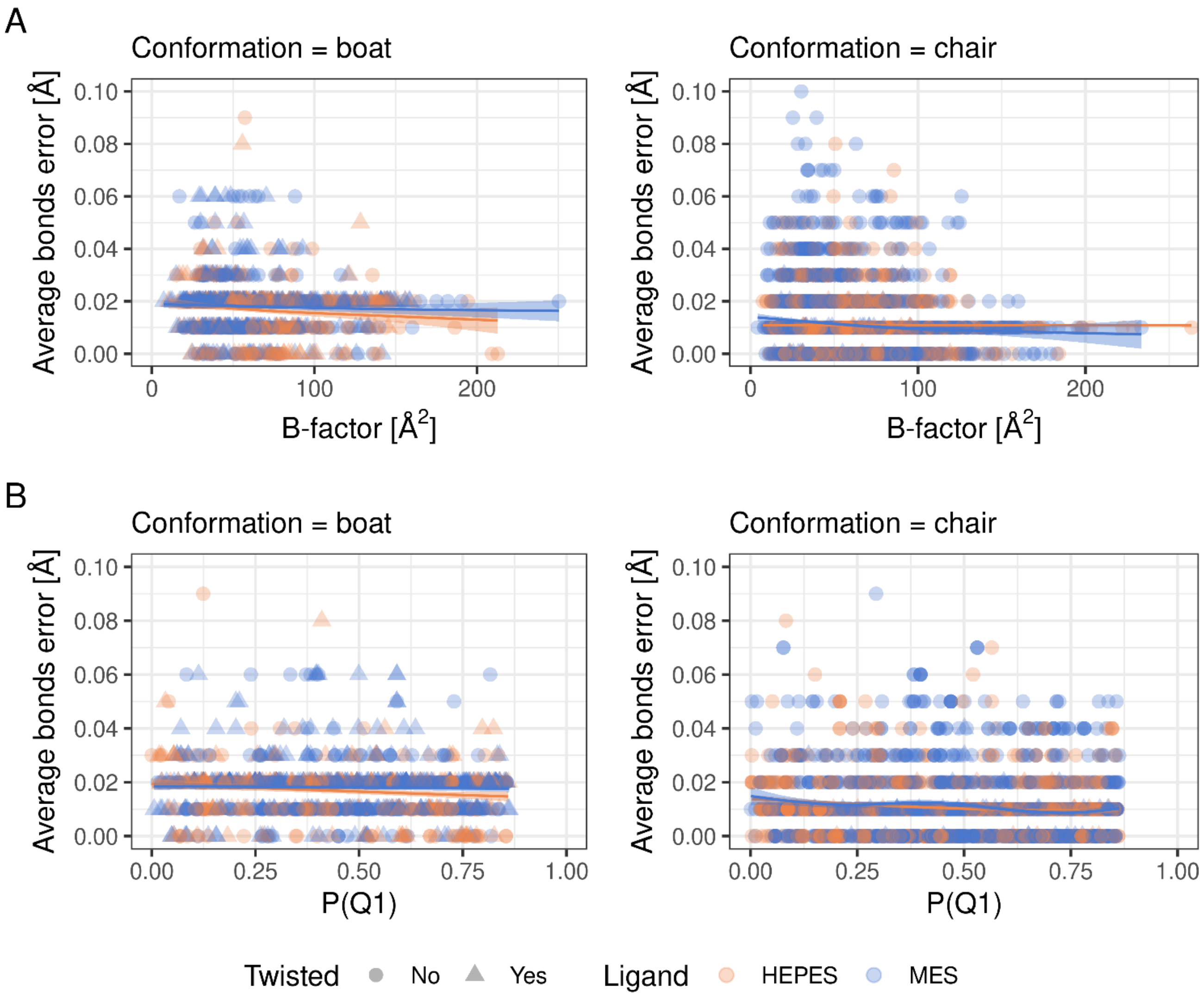

SI Figure 3 The average deviation of bond lengths from their ideal values in regards to the average B-factor of ligand atoms (A), or an overall structure-quality indicator - P(Q1)(B)~~)~~. A regression model relating the x and y variables is shown as a solid line, and the confidence interval for the regression estimate is drawn using translucent bands around the regression line

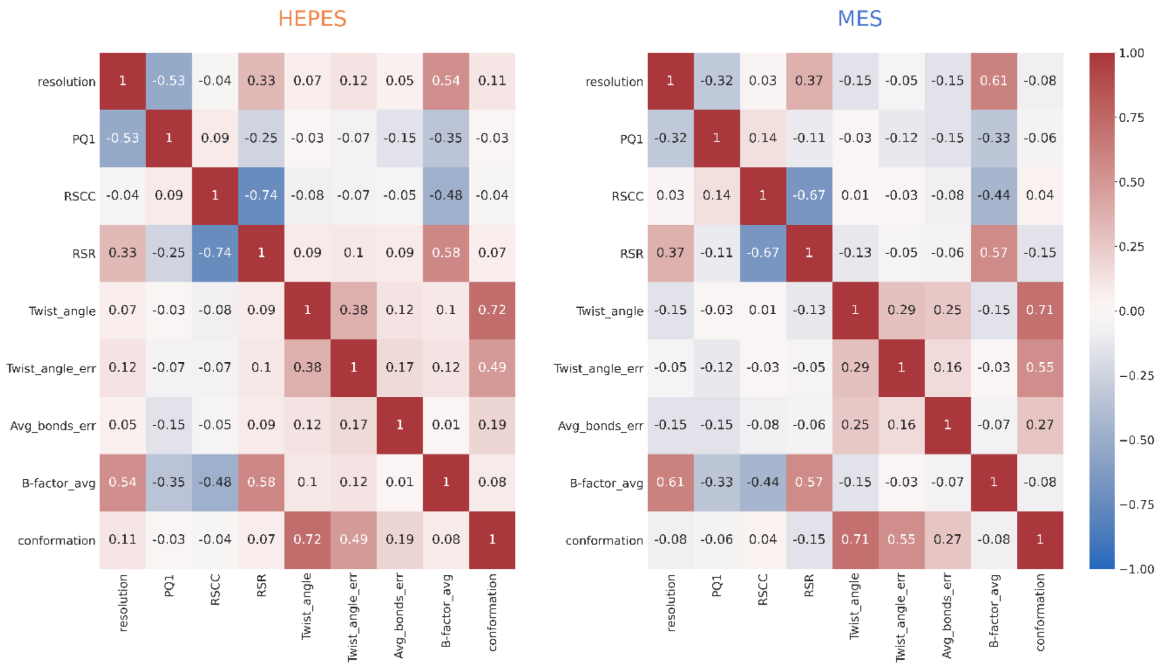

SI Figure 4 Heatmaps presenting Spearman's rank correlation coefficient for several parameters for HEPES (A) and MES (B) molecules

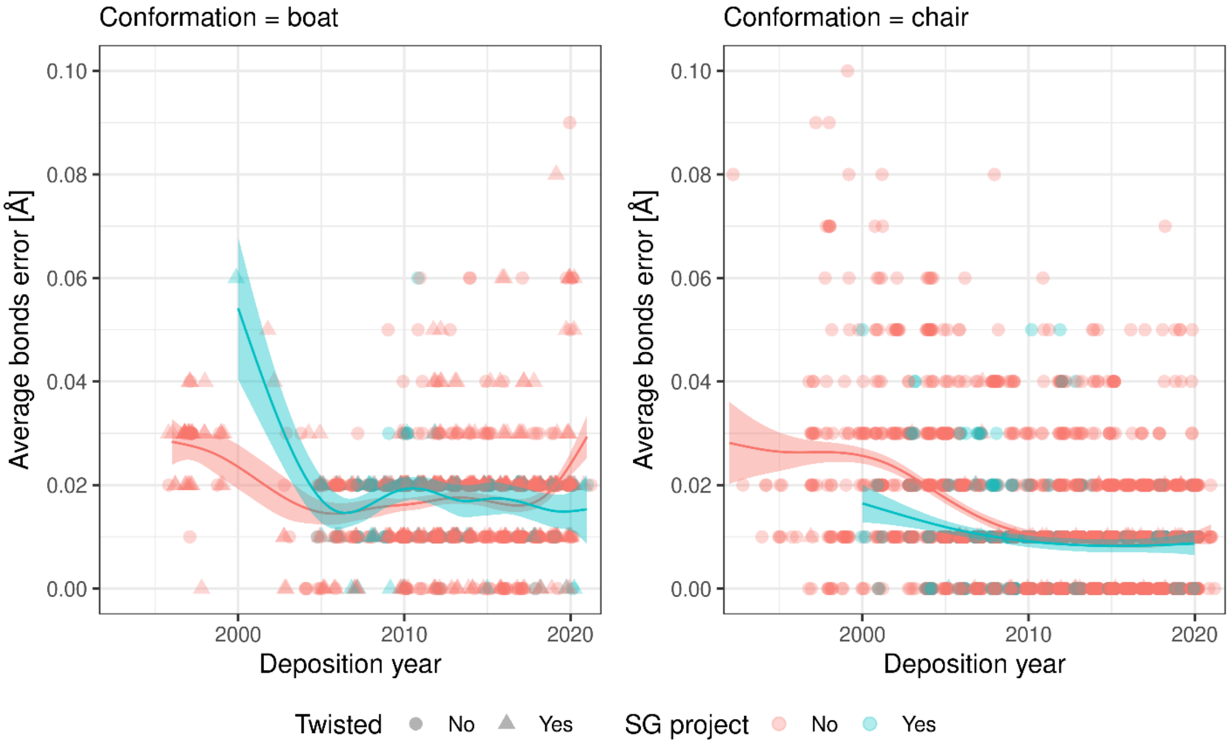

SI Figure 5 The absolute deviation of bonds error from their ideal values reported for structures from Structural Genomics and other projects in different years. A regression model relating x and y variables is shown as a solid line, and the confidence interval for the regression estimate is drawn using translucent bands around the regression line

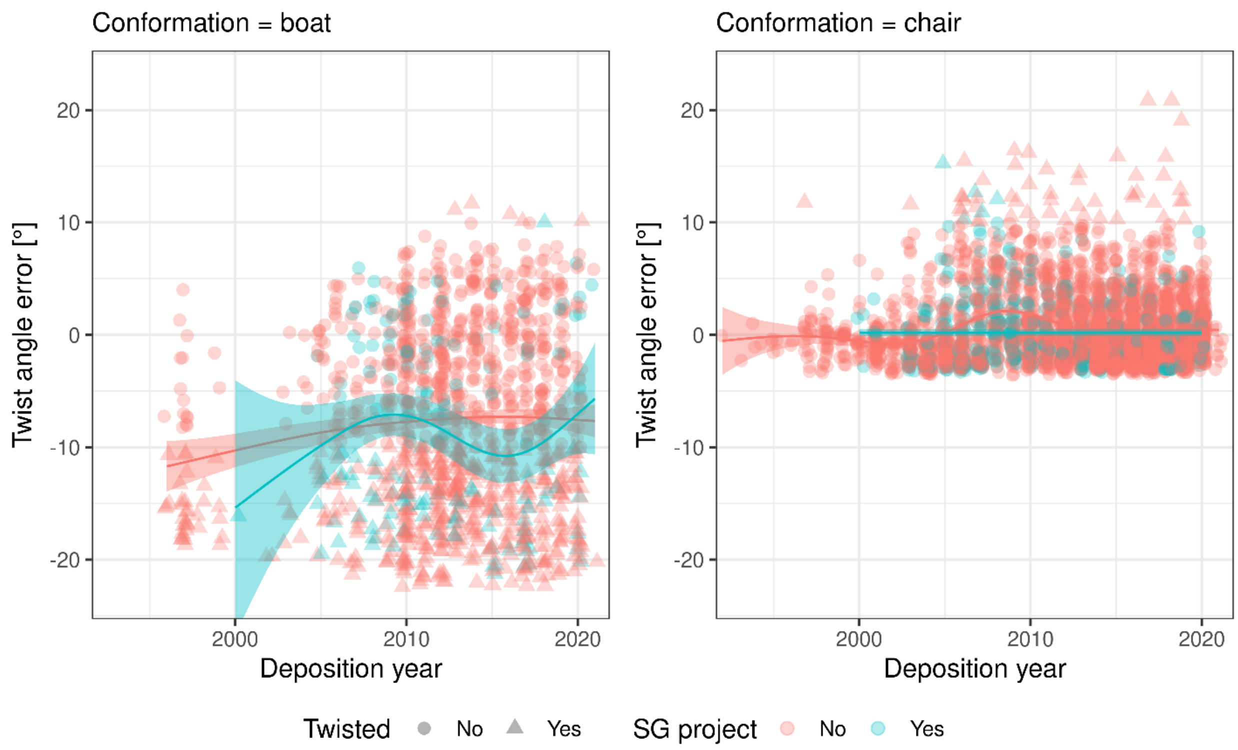

SI Figure 6 The deviation of twisted angles from their ideal values reported for structures from Structural Genomics and other projects in different years. A regression model relating x and y variables is shown as a solid line, and the confidence interval for the regression estimate is drawn using translucent bands around the regression line

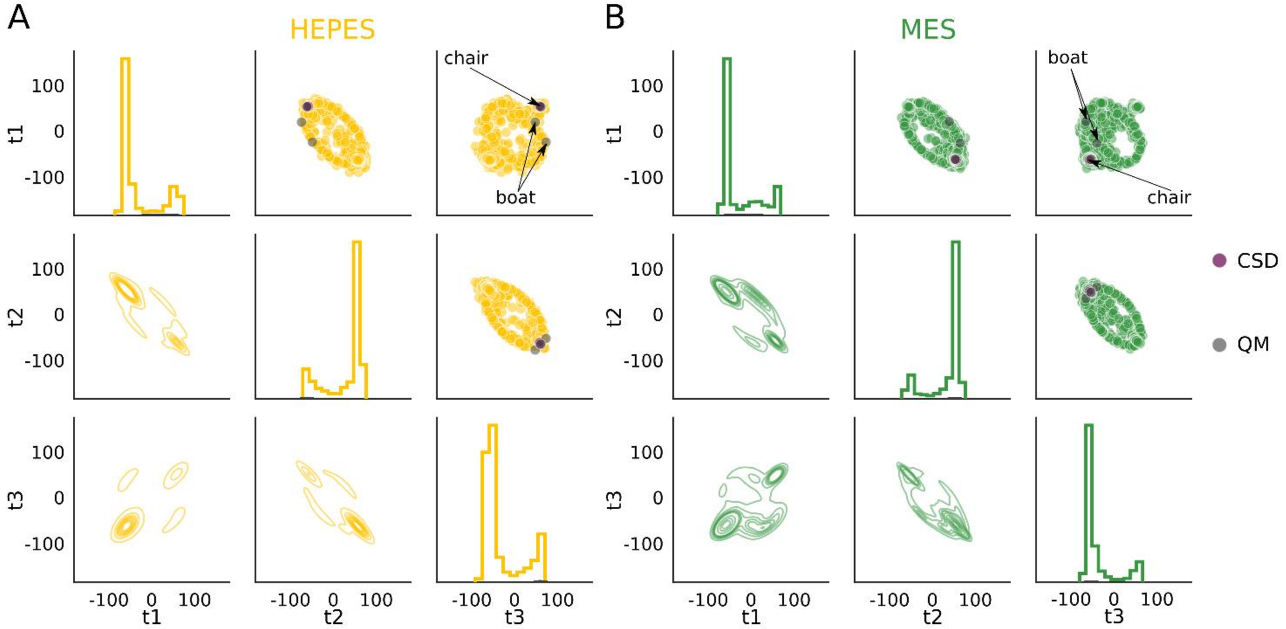

SI Figure 7 Scatter, KDE plots, and histograms showing three subsequent torsion angles from a six-member ring of HEPES (orange) and MES (blue) from PDB_REDO (no resolution filtration). In dark grey results for the same ligands from CSD are shown. CSD structures were omitted in the KDE plots

SI Table I The 30 most frequently occurring small molecules that HEPES or MES agents can substitute. The list corresponds to the molecules found within 0.5 Å from HEPES or MES as described in the core text

Counts	Ligand ID
**36674**	MES
**7832**	EPE
**2164**	SO4
**1158**	EDO
**974**	GOL
**750**	GSH
**607**	PO4
**562**	ACT
**388**	GLU
**355**	MTX
**284**	APR
**280**	DMS
**252**	KAI
**247**	ASP
**227**	ACO
**222**	FOL
**218**	CL
**215**	HEM
**210**	COA
**173**	PEG
**172**	MPD
**166**	CEF
**161**	FAD
**157**	BFO
**136**	3BV
**133**	NXL
**128**	ADP
**128**	AR6
**123**	CIT
**123**	ESY

SI Table II Experimental data and calculated resolutions for investigated structures of HEPES and MES from CSD

Ligand	CSD identifier	Conformation	Radiation wavelength λ [Å]	Maximum Θ [deg]	Resolution* [Å]
**HEPES**	WIRMOZ[1]	chair	1.54178	53.74	0.96
**HEPES**	WIRMOZ01[2]	chair	0.71073	25.00	0.84
**HEPES**	WIRMOZ02[3]	chair	0.71070	62.90	0.40
**HEPES**	WIRMOZ03[4]	chair	0.71073	55.85	0.43

**HEPES**	WIRMOZ04[5]	Chair	0.71073	55.85	0.43
**MES**	YATVUK[6]	chair	0.71070	18.83[&]	1.10[&]
**MES**	YATVUK01[7]	chair	0.81560	20.00	1.19
**MES**	YAMXUG[8]	chair	0.71073	26.50	0.80

* Calculated using Bragg's law
& Calculated using max sin(theta)/lambda provided by authors

SI References

1. Wouters J, Häming L, Sheldrick G (1996) HEPES. Acta Crystallogr. C 52:1687–1688.

2. Gao F, Yin C, Yang P, Xue G (2004) 2-[4-(2-Hydro-xy-ethyl)-piperazin-1-yl]-ethane-sulfonic acid. Acta Crystallogr. Sect. E Struct. Rep. Online 60:o1328–o1329.

3. Sledz P, Minor T, Chruszcz M (2009) Redetermination of 2-[4-(2-hydroxy-ethyl)piperazin-1-ium-1-yl]ethanesul-fonate at 100 K. Acta Crystallogr. Sect. E Struct. Rep. Online 65:o3027–o3028.

4. Śledź P, Kamiński R, Chruszcz M, Zimmerman MD, Minor W, Woźniak K (2010) An experimental charge density of HEPES. Acta Crystallogr. B 66:482–492.

5. Woińska M, Grabowsky S, Dominiak PM, Woźniak K, Jayatilaka D (2016) Hydrogen atoms can be located accurately and precisely by x-ray crystallography. Sci. Adv. 2:e1600192.

6. Christensen AN, Hazell RG, Lehmann MS, Nielsen M (1993) Crystal Structure of 2-[N-Morpholino] ethane Sulfonic Acid Hydrate, C6H15NO5S. Acta Chem. Scand. 47:753–753.

7. Kubicki M, Adamiak DA, Rypniewski WR, Olejniczak A (2007) A synchrotron redetermination of 2-(morpholinium-4-yl)ethanesulfonate monohydrate, including a disordered water mol-ecule. Acta Crystallogr. Sect. E Struct. Rep. Online 63:o2604–o2606.

8. Milić D, Renić M, Matković-Čalogović D (2005) Caesium chloride 2-(N-morpholinio)-ethane-sulfonate. Acta Crystallogr. Sect. E Struct. Rep. Online 61:m757–m758.
