# Supplementary material for: Analysis of protein structures containing HEPES and MES molecules
Source: Protein Sci. 2022 Aug 26;31(9):e4415. doi: 10.1002/pro.4415 (PMC9601878; doi:10.1002/pro.4415)
Supplement: Supplementary file 4 — Appendix S2 Supporting Information [file PRO-31-e4415-s002.pdf]

Data collection and refinement statistics for project **mes_3k4l** crystal **crystal1**

model **/home/asia/epe-like-validation/RESULTS/HKL_rerefine/most_twisted/3k4l/structure_mr/build_model_3/hkl_import.pdb_tls** vs
model **/home/asia/epe-like-validation/RESULTS/HKL_rerefine/most_twisted/3k4l/structure_mr/build_model_3/hkl_refine_42.pdb**

**Data collection**		
	**3K4L**	**Re-refinement**
Resolution (Å)	30.00 - 1.75 (1.80 - 1.75)	28.66 - 1.75
Wavelength (Å)	1.0379	
Space group	P43212	P43212
a, b, c (Å)	101.57, 101.57, 250.05	101.57, 101.57, 250.05
α, β, γ (°)	90, 90, 90	90, 90, 90
Completeness (%)	99.9 (99.9)	99.9 (99.9)
Reflections used	132005	
<I> / <Sigma I>	15.4 (2.5)	
Redundancy	7.3 (7.3)	
Rmerge	0.102 (0.883)	
Rpim		
CC1/2 last shell		
Wilson B factor (Å²)	17.3	
**Refinement**		
Rwork / Rfree	0.175 / 0.209	0.178 / 0.210
Resolution (Å)	30.00 - 1.75	28.66 - 1.75
Reflections all	130031	132005
Reflections for Rfree	1974, 1.5%	1974, 1.5%
Bond lengths rmsd (Å)	0.021	0.025
Bond angles rmsd (°)	1.99	2.29
Mean B value (Å²)	20	19
Number of protein atoms	8944	8944
Mean B value for protein atoms (Å²)	20	19
Number of water atoms (expected)	722 (1322)	722 (1322)
Mean B value for water atoms (Å²)	25	23
Number of ligand/ion atoms	166	166
Mean B value for ligand/ion atoms (Å²)	20	19
Clashscore	3.92	3.86
Clashscore percentile (100)	-1	-1
Rotamer outliers (<1%)	1.21	1.21
Ramachandran outliers (<0.2%)	0.00	0.00
Ramachandran favored (>98%)	97.25	97.25
Residues with bad bonds (<0%)	2.38	2.47
Residues with bad angles (<0.1%)	4.50	4.32
MolProbity score	1.38	1.38

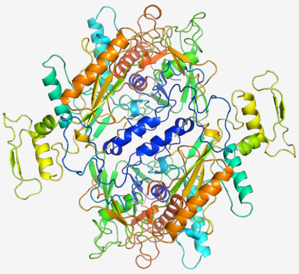

Map cc barchart:

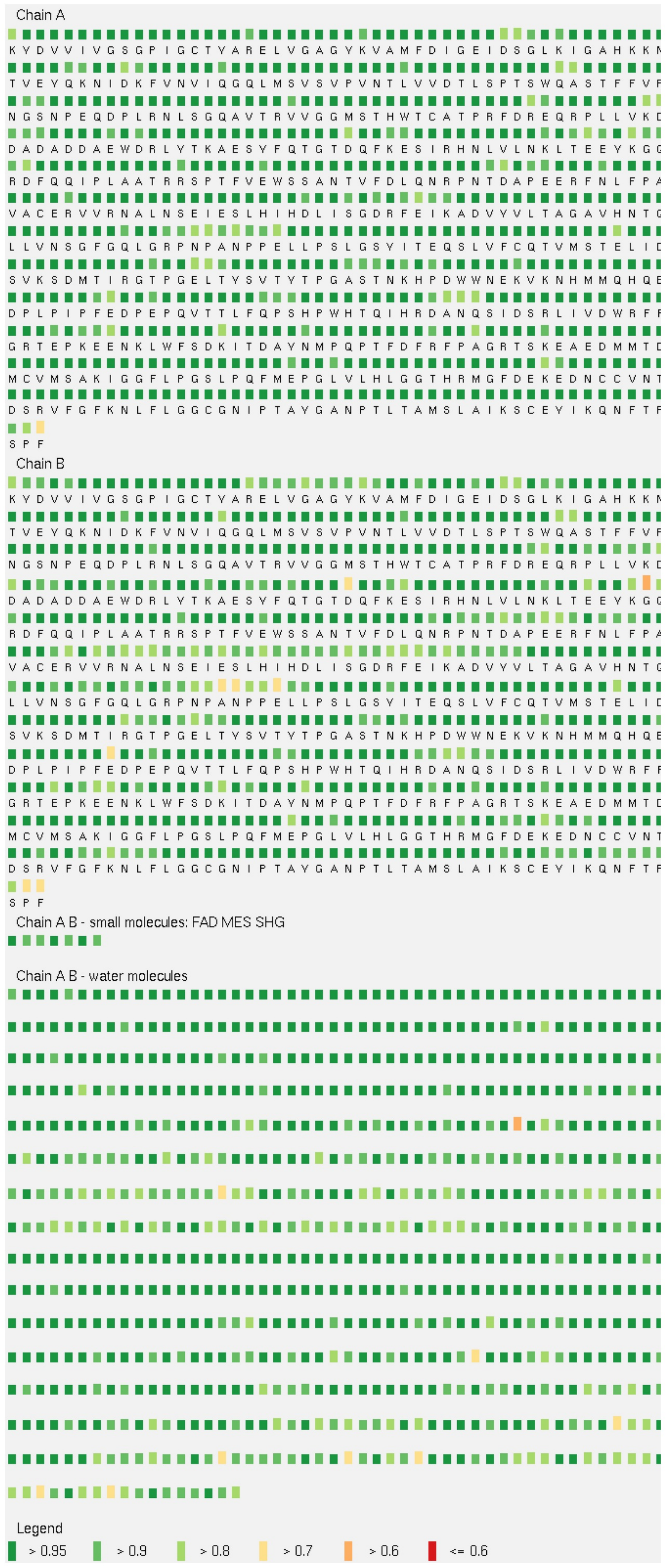

Small molecules with map:

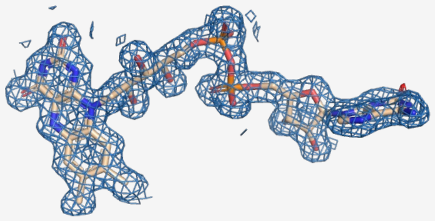

Small molecule 1: FLAVIN-ADENINE DINUCLEOTIDE (FAD) A 801 map cc 0.99

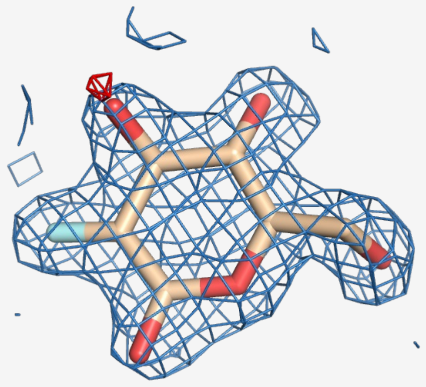

Small molecule 2: 2-deoxy-2-fluoro-beta-D-glucopyranos (SHG) A 901 map cc 0.93

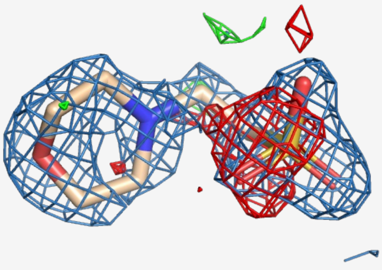

Small molecule 3: 2-(N-MORPHOLINO)-ETHANESULFONIC ACID (MES) A 902 map cc 0.93

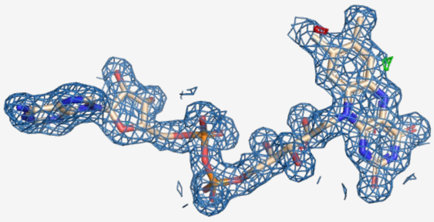

Small molecule 4: FLAVIN-ADENINE DINUCLEOTIDE (FAD) B 801 map cc 0.98

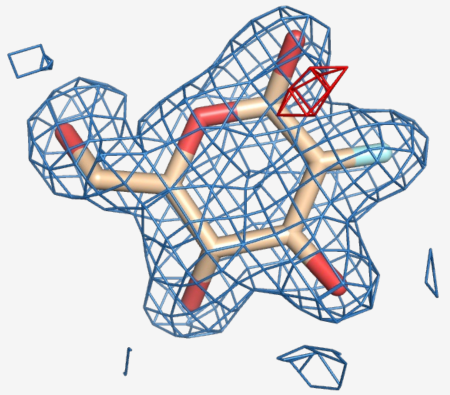

Small molecule 5: 2-deoxy-2-fluoro-beta-D-glucopyranos (SHG) B 901 map cc 0.92

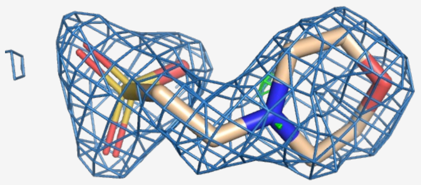

Small molecule 6: 2-(N-MORPHOLINO)-ETHANESULFONIC ACID (MES) B 903 map cc 0.98

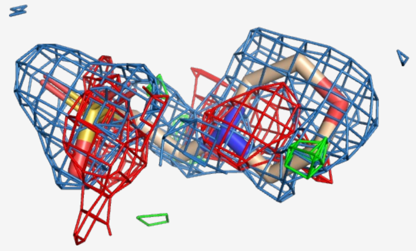

Small molecule 7: 2-(N-MORPHOLINO)-ETHANESULFONIC ACID (MES) B 902 map cc 0.91

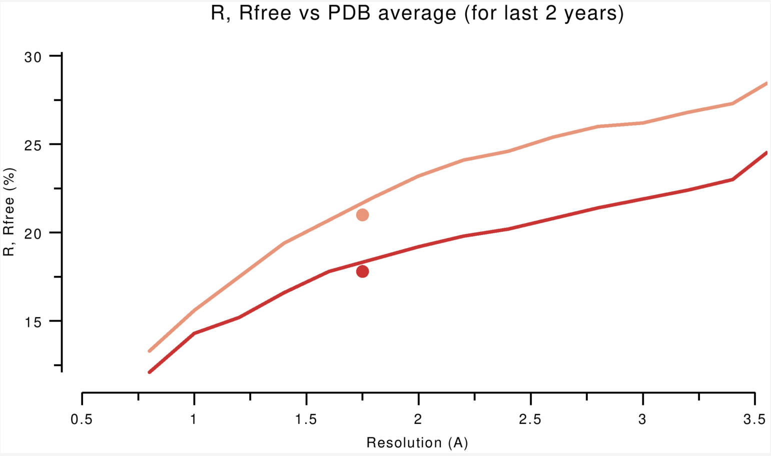

Credits:

HKL-3000

"Processing of X-ray Diffraction Data Collected in Oscillation Mode"
Z.Otwinowski, W.Minor
Methods in Enzymology, Volume 276: Macromolecular Crystallography, part A, p307-326 (1997)
"HKL-3000: the integration of data reduction and structure solution - from diffraction images to an initial model in minutes"
W.Minor, M.Cymborowski, Z.Otwinowski, M.Chruszcz
Acta Cryst. D62: 859-866 (2006)

CCP4 suite
"Overview of the CCP4 suite and current developments"
M.D.Winn et al.
Acta. Cryst. D67, 235-242 (2011)

Refmac5
"REFMAC5 for the refinement of macromolecular crystal structures"
G.N.Murshudov, P.Skubak, A.A.Lebedev, N.S.Pannu, R.A.Steiner, R.A.Nicholls, M.D.Winn, F.Long and A.A.Vagin
Acta Cryst. D67, 355-367 (2011)

Coot
"Features and Development of Coot"
P.Emsley, B.Lohkamp, W.Scott, and K.Cowtan
Acta Cryst. D66, 486-501 (2010)

Data collection and refinement statistics for project **mes_3k4l** crystal **crystal1**

model **/home/asia/epe-like-validation/RESULTS/HKL_rerefine/most_twisted/3k4l/structure_mr/build_model_3/hkl_import.pdb_tls** vs
model **/home/asia/epe-like-validation/RESULTS/HKL_rerefine/most_twisted/3k4l/structure_mr/build_model_3/hkl_refine_40.pdb_tls**

Data collection		
	**3K4L**	**Re-refinement**
Resolution (Å)	30.00 - 1.75 (1.80 - 1.75)	28.66 - 1.75
Wavelength (Å)	1.0379	
Space group	P43212	P43212
a, b, c (Å)	101.57, 101.57, 250.05	101.57, 101.57, 250.05
α, β, γ (°)	90, 90, 90	90, 90, 90
Completeness (%)	99.9 (99.9)	99.9 (99.9)
Reflections used	132005	
<I> / <Sigma I>	15.4 (2.5)	
Redundancy	7.3 (7.3)	
Rmerge	0.102 (0.883)	
Rpim		
CC1/2 last shell		
Wilson B factor (Å^2)	17.3	
**Refinement**		
Rwork / Rfree	0.175 / 0.209	0.173 / 0.201
Resolution (Å)	30.00 - 1.75	28.66 - 1.75
Reflections all	130031	132005
Reflections for Rfree	1974, 1.5%	1974, 1.5%
Bond lengths rmsd (Å)	0.021	0.015
Bond angles rmsd (°)	1.99	1.92
Mean B value (Å^2)	20	20
Number of protein atoms	8944	8988
Mean B value for protein atoms (Å^2)	20	19
Number of water atoms (expected)	722 (1322)	729 (1322)
Mean B value for water atoms (Å^2)	25	23
Number of ligand/ion atoms	166	166
Mean B value for ligand/ion atoms (Å^2)	20	19
Clashscore	3.92	2.00
Clashscore percentile (100)	-1	-1
Rotamer outliers (<1%)	1.21	0.70
Ramachandran outliers (<0.2%)	0.00	0.00
Ramachandran favored (>98%)	97.25	97.87
Residues with bad bonds (<0%)	2.38	1.14
Residues with bad angles (<0.1%)	4.50	2.64
MolProbity score	1.38	1.00

Map cc barchart:

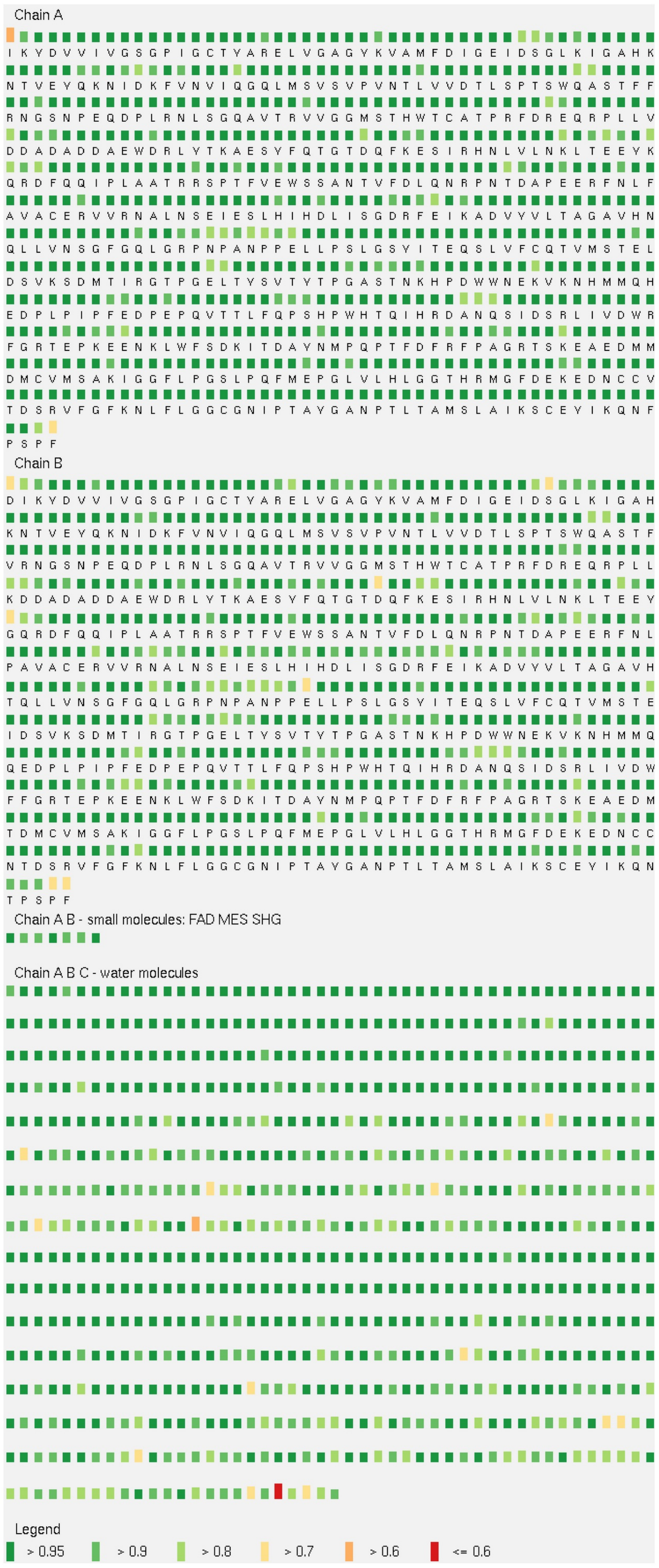

Small molecules with map:

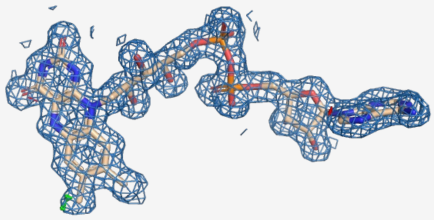

Small molecule 1: FLAVIN-ADENINE DINUCLEOTIDE (FAD) A 801 map cc 0.98

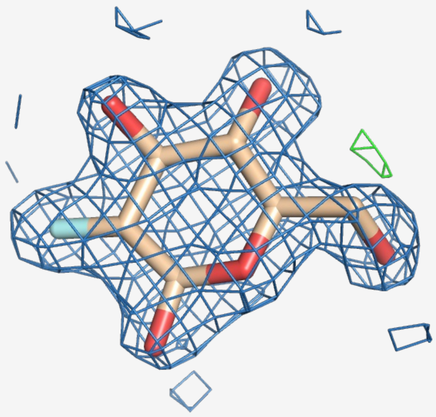

Small molecule 2: 2-deoxy-2-fluoro-beta-D-glucopyranos (SHG) A 901 map cc 0.94

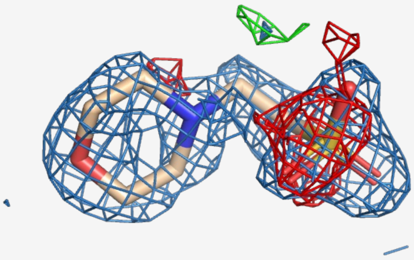

Small molecule 3: 2-(N-MORPHOLINO)-ETHANESULFONIC ACID (MES) A 902 map cc 0.94

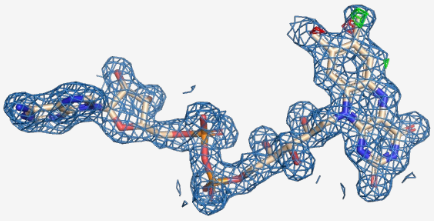

Small molecule 4: FLAVIN-ADENINE DINUCLEOTIDE (FAD) B 801 map cc 0.98

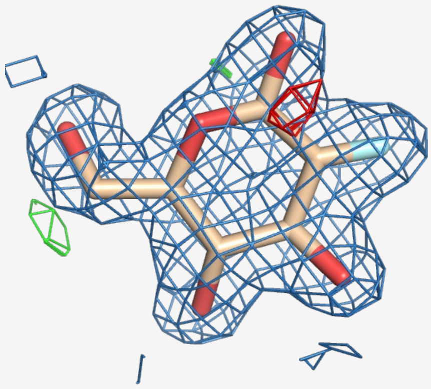

Small molecule 5: 2-deoxy-2-fluoro-beta-D-glucopyranos (SHG) B 901 map cc 0.93

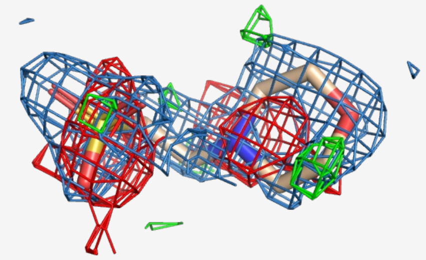

Small molecule 6: 2-(N-MORPHOLINO)-ETHANESULFONIC ACID (MES) B 902 map cc 0.92

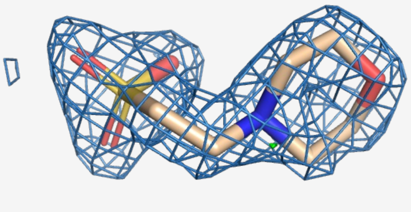

Small molecule 7: 2-(N-MORPHOLINO)-ETHANESULFONIC ACID (MES) B 903 map cc 0.99

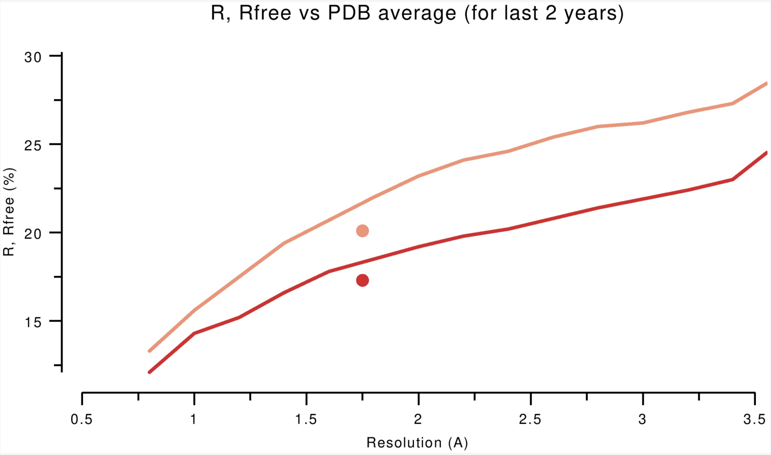

Credits:

HKL-3000

"Processing of X-ray Diffraction Data Collected in Oscillation Mode"
Z.Otwinowski, W.Minor
Methods in Enzymology, Volume 276: Macromolecular Crystallography, part A, p307-326 (1997)
"HKL-3000: the integration of data reduction and structure solution - from diffraction images to an initial model in minutes"
W.Minor, M.Cymborowski, Z.Otwinowski, M.Chruszcz
Acta Cryst. D62: 859-866 (2006)

CCP4 suite
"Overview of the CCP4 suite and current developments"
M.D.Winn et al.
Acta. Cryst. D67, 235-242 (2011)

Refmac5
"REFMAC5 for the refinement of macromolecular crystal structures"
G.N.Murshudov, P.Skubak, A.A.Lebedev, N.S.Pannu, R.A.Steiner, R.A.Nicholls, M.D.Winn, F.Long and A.A.Vagin
Acta Cryst. D67, 355-367 (2011)

Coot
"Features and Development of Coot"
P.Emsley, B.Lohkamp, W.Scott, and K.Cowtan
Acta Cryst. D66, 486-501 (2010)

Data collection and refinement statistics for project **mes_3pyi** crystal **crystal1**

model **/home/asia/epe-like-validation/RESULTS/HKL_rerefine/most_twisted/3pyi/structure_mr/build_model_2/hkl_import.pdb** vs
model **/home/asia/epe-like-validation/RESULTS/HKL_rerefine/most_twisted/3pyi/structure_mr/build_model_2/hkl_refine_45.pdb**

Data collection

	3PYI	Re-refinement
Resolution (Å)	73.15 - 2.10 (2.22 - 2.10)	50.68 - 2.10
Wavelength (Å)	1.0	
Space group	P212121	P212121
a, b, c (Å)	70.27, 73.15, 79.60	70.27, 73.15, 79.60
α, β, γ (°)	90, 90, 90	90, 90, 90
Completeness (%)	99.6 (99.3)	100.0 (100.0)
Reflections used	24368	
<I> / <Sigma I>	17.7 (4.0)	
Redundancy	3.6 (3.6)	
Rmerge	0.039	
Rpim		
CC1/2 last shell		
Wilson B factor (Å^2)	31.5	
**Refinement**		
Rwork / Rfree	0.210 / 0.257	0.222 / 0.259
Resolution (Å)	50.68 - 2.10	50.69 - 2.10
Reflections all	23106	24319
Reflections for Rfree	2220, 4.9%	1213, 5.0%
Bond lengths rmsd (Å)	0.007	0.015
Bond angles rmsd (°)	1.02	1.75
Mean B value (Å^2)	46	44
Number of protein atoms	2352	2352
Mean B value for protein atoms (Å^2)	45	42
Number of water atoms (expected)	146 (263)	146 (263)
Mean B value for water atoms (Å^2)	46	48
Number of ligand/ion atoms	49	49
Mean B value for ligand/ion atoms (Å^2)	94	88
Clashscore	5.93	5.92
Clashscore percentile (100)	-1	-1
Rotamer outliers (<1%)	0.00	0.00
Ramachandran outliers (<0.2%)	0.36	0.36
Ramachandran favored (>98%)	98.19	98.19
Residues with bad bonds (<0%)	0.00	0.18
Residues with bad angles (<0.1%)	0.00	0.00
MolProbity score	1.32	1.32

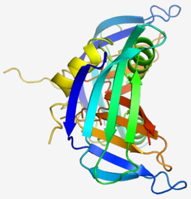

Map cc barchart:

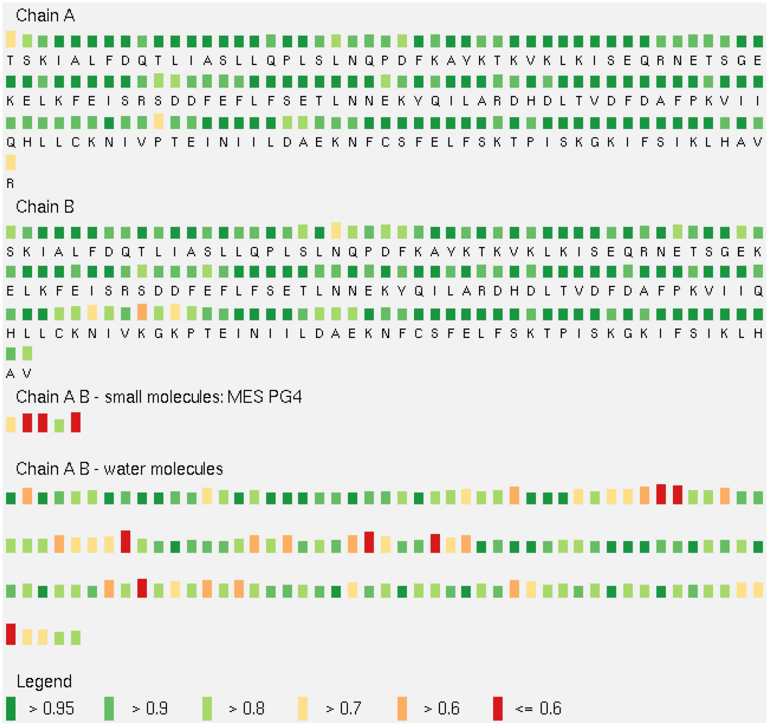

Small molecules with map:

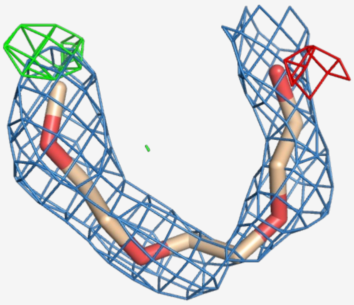

Small molecule 1: TETRAETHYLENE GLYCOL (PG4) B 169 map cc 0.76

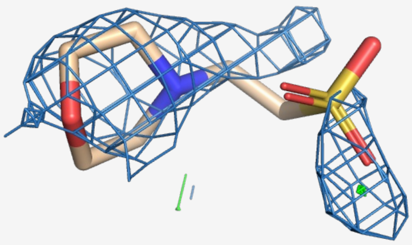

Small molecule 2: 2-(N-MORPHOLINO)-ETHANESULFONIC ACID (MES) B 170 map cc 0.54

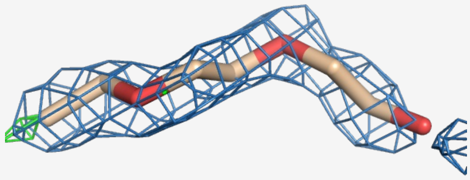

Small molecule 3: TETRAETHYLENE GLYCOL (PG4) B 171 map cc 0.54

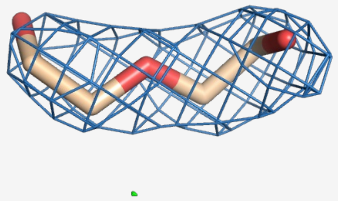

Small molecule 4: TETRAETHYLENE GLYCOL (PG4) A 169 map cc 0.88

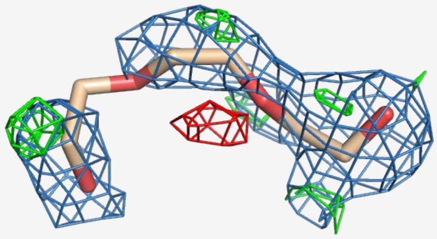

Small molecule 5: TETRAETHYLENE GLYCOL (PG4) A 170 map cc 0.51

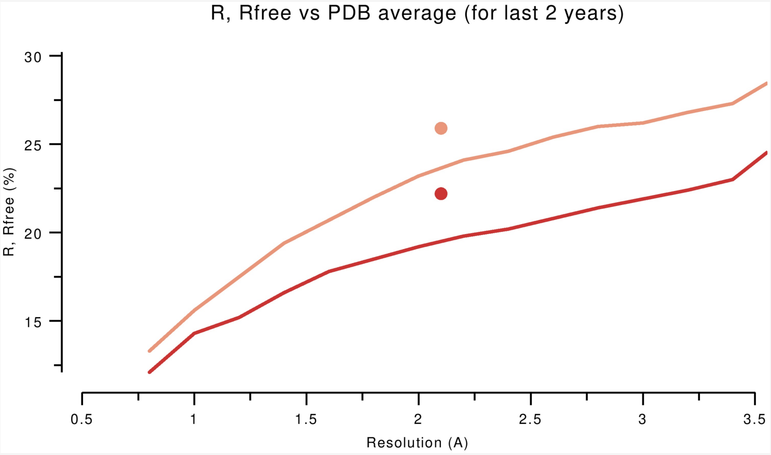

Credits:

HKL-3000

"Processing of X-ray Diffraction Data Collected in Oscillation Mode"
Z.Otwinowski, W.Minor
Methods in Enzymology, Volume 276: Macromolecular Crystallography, part A, p307-326 (1997)
"HKL-3000: the integration of data reduction and structure solution - from diffraction images to an initial model in minutes"
W.Minor, M.Cymborowski, Z.Otwinowski, M.Chruszcz
Acta Cryst. D62: 859-866 (2006)

CCP4 suite
"Overview of the CCP4 suite and current developments"
M.D.Winn et al.
Acta. Cryst. D67, 235-242 (2011)

Refmac5
"REFMAC5 for the refinement of macromolecular crystal structures"
G.N.Murshudov, P.Skubak, A.A.Lebedev, N.S.Pannu, R.A.Steiner, R.A.Nicholls, M.D.Winn, F.Long and A.A.Vagin
Acta Cryst. D67, 355-367 (2011)

Coot
"Features and Development of Coot"
P.Emsley, B.Lohkamp, W.Scott, and K.Cowtan
Acta Cryst. D66, 486-501 (2010)

Data collection and refinement statistics for project **mes_3pyi** crystal **crystal1**

model **/home/asia/epe-like-validation/RESULTS/HKL_rerefine/most_twisted/3pyi/structure_mr/build_model_2/hkl_import.pdb** vs
model **/home/asia/epe-like-validation/RESULTS/HKL_rerefine/most_twisted/3pyi/structure_mr/build_model_2/hkl_refine_36.pdb_tls**

Data collection		
	**3PYI**	**Re-refinement**
Resolution (Å)	73.15 - 2.10 (2.22 - 2.10)	50.68 - 2.10
Wavelength (Å)	1.0	
Space group	P212121	P212121
a, b, c (Å)	70.27, 73.15, 79.60	70.27, 73.15, 79.60
α, β, γ (°)	90, 90, 90	90, 90, 90
Completeness (%)	99.6 (99.3)	100.0 (100.0)
Reflections used	24368	
<I> / <Sigma I>	17.7 (4.0)	
Redundancy	3.6 (3.6)	
Rmerge	0.039	
Rpim		
CC1/2 last shell		
Wilson B factor (Å²)	31.5	
**Refinement**		
Rwork / Rfree	0.210 / 0.257	0.205 / 0.262
Resolution (Å)	50.68 - 2.10	50.69 - 2.10
Reflections all	23106	24319
Reflections for Rfree	2220, 4.9%	1213, 5.0%
Bond lengths rmsd (Å)	0.007	0.014
Bond angles rmsd (°)	1.02	1.91
Mean B value (Å²)	46	44
Number of protein atoms	2352	2340
Mean B value for protein atoms (Å²)	45	43
Number of water atoms (expected)	146 (263)	133 (263)
Mean B value for water atoms (Å²)	46	47
Number of ligand/ion atoms	49	51
Mean B value for ligand/ion atoms (Å²)	94	90
Clashscore	5.93	2.47
Clashscore percentile (100)	-1	-1
Rotamer outliers (<1%)	0.00	0.37
Ramachandran outliers (<0.2%)	0.36	0.00
Ramachandran favored (>98%)	98.19	98.19
Residues with bad bonds (<0%)	0.00	0.88
Residues with bad angles (<0.1%)	0.00	2.63
MolProbity score	1.32	1.03

Map cc barchart:

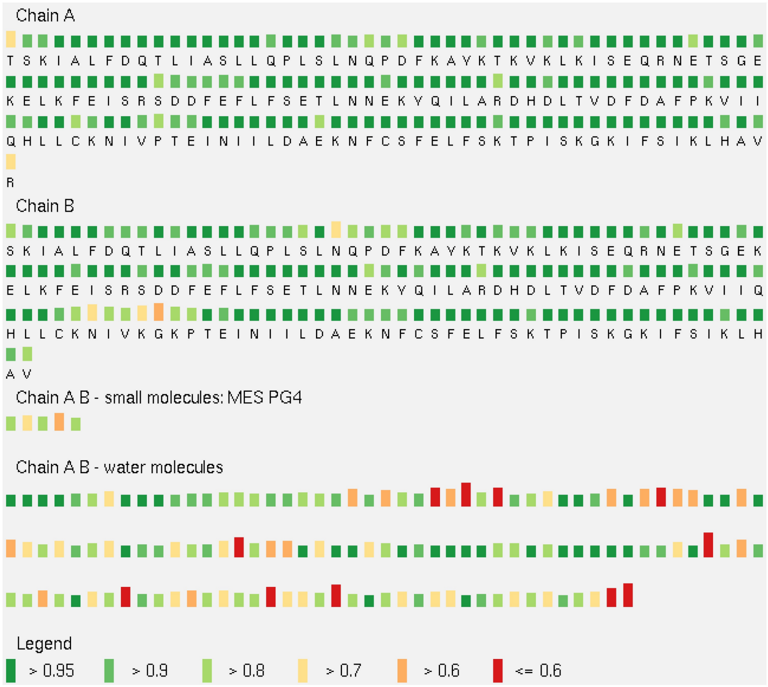

Small molecules with map:

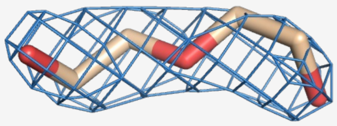

Small molecule 1: TETRAETHYLENE GLYCOL (PG4) A 169 map cc 0.85

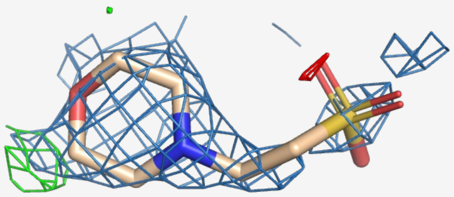

Small molecule 2: 2-(N-MORPHOLINO)-ETHANESULFONIC ACID (MES) A 170 map cc 0.77

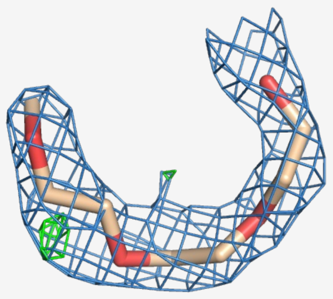

Small molecule 3: TETRAETHYLENE GLYCOL (PG4) B 169 map cc 0.82

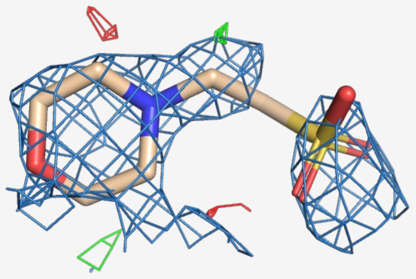

Small molecule 4: 2-(N-MORPHOLINO)-ETHANESULFONIC ACID (MES) B 170 map cc 0.61

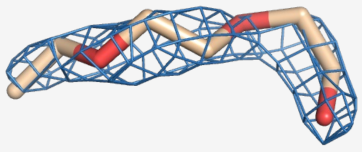

Small molecule 5: TETRAETHYLENE GLYCOL (PG4) B 171 map cc 0.87

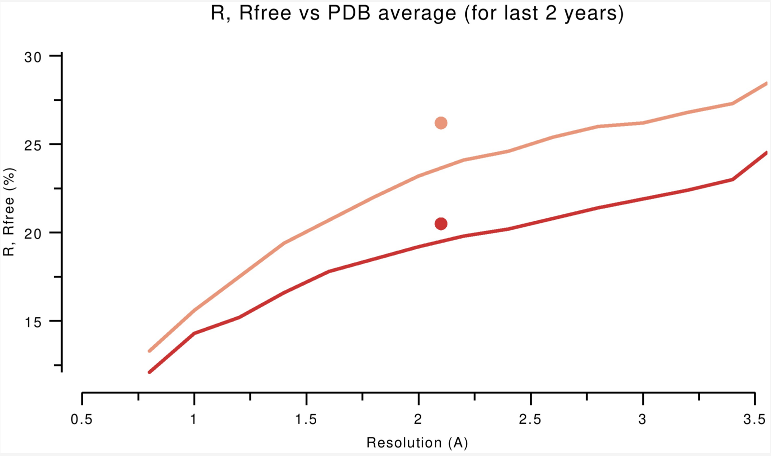

Credits:

HKL-3000

"Processing of X-ray Diffraction Data Collected in Oscillation Mode"
Z.Otwinowski, W.Minor
Methods in Enzymology, Volume 276: Macromolecular Crystallography, part A, p307-326 (1997)
"HKL-3000: the integration of data reduction and structure solution - from diffraction images to an initial model in minutes"
W.Minor, M.Cymborowski, Z.Otwinowski, M.Chruszcz
Acta Cryst. D62: 859-866 (2006)

CCP4 suite
"Overview of the CCP4 suite and current developments"
M.D.Winn et al.
Acta. Cryst. D67, 235-242 (2011)

Refmac5
"REFMAC5 for the refinement of macromolecular crystal structures"
G.N.Murshudov, P.Skubak, A.A.Lebedev, N.S.Pannu, R.A.Steiner, R.A.Nicholls, M.D.Winn, F.Long and A.A.Vagin
Acta Cryst. D67, 355-367 (2011)

Coot
"Features and Development of Coot"
P.Emsley, B.Lohkamp, W.Scott, and K.Cowtan
Acta Cryst. D66, 486-501 (2010)

Data collection and refinement statistics for project **hepes_5t6l** crystal **crystal1**

model **/home/asia/epe-like-validation/RESULTS/HKL_rerefine/most_twisted/5t6l/structure_mr/build_model_2/hkl_import.pdb** vs
model **/home/asia/epe-like-validation/RESULTS/HKL_rerefine/most_twisted/5t6l/structure_mr/build_model_2/hkl_refine_38.pdb**

**Data collection**		
	**5T6L**	**Re-refinement**
Resolution (Å)	47.14 - 2.10 (2.15 - 2.10)	47.14 - 2.10
Wavelength (Å)	1.03314	
Space group	P21	P21
a, b, c (Å)	41.10, 158.97, 99.69	41.10, 158.97, 99.69
α, β, γ (°)	90, 97.98, 90	90, 97.98, 90
Completeness (%)	97.5 (91.9)	100.0 (100.0)
Reflections used	72210	
<I> / <Sigma I>	9.3 (1.6)	
Redundancy	2.4 (2.3)	
Rmerge	0.074	
Rpim		
CC1/2 last shell		
Wilson B factor (Å^2)	31.3	
**Refinement**		
Rwork / Rfree	0.178 / 0.218	0.197 / 0.230
Resolution (Å)	47.14 - 2.10	47.14 - 2.10
Reflections all	72183	72118
Reflections for Rfree	3620, 5.0%	3615, 5.0%
Bond lengths rmsd (Å)	0.008	0.015
Bond angles rmsd (°)	0.88	1.68
Mean B value (Å^2)	53	52
Number of protein atoms	9023	9023
Mean B value for protein atoms (Å^2)	53	52
Number of water atoms (expected)	405 (2846)	405 (2846)
Mean B value for water atoms (Å^2)	45	47
Number of ligand/ion atoms	117	117
Mean B value for ligand/ion atoms (Å^2)	66	70
Clashscore	4.70	4.69
Clashscore percentile (100)	-1	-1
Rotamer outliers (<1%)	3.62	3.62
Ramachandran outliers (<0.2%)	0.69	0.69
Ramachandran favored (>98%)	95.52	95.52
Residues with bad bonds (<0%)	0.05	0.10
Residues with bad angles (<0.1%)	0.10	0.10
MolProbity score	1.98	1.98

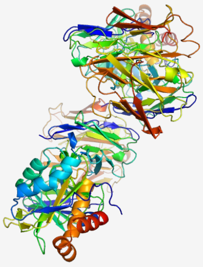

Map cc barchart:

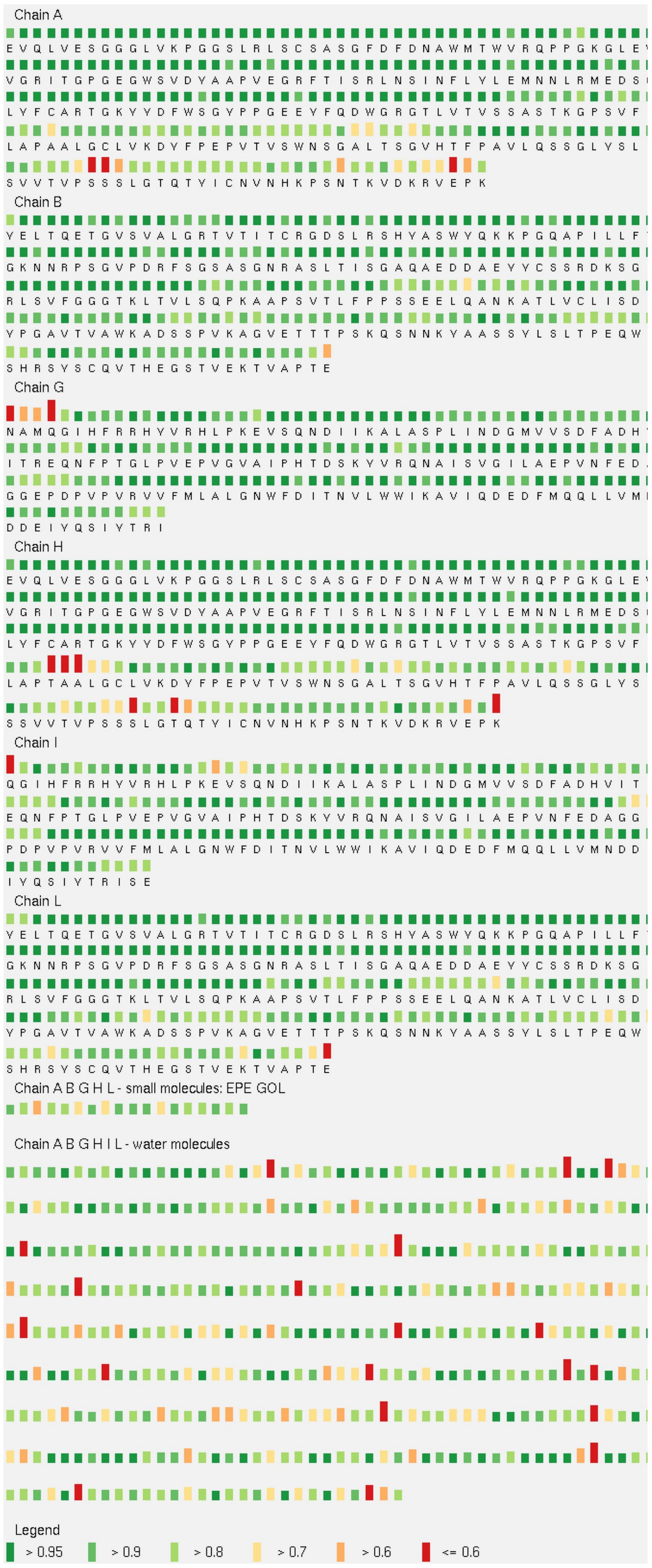

Small molecules with map:

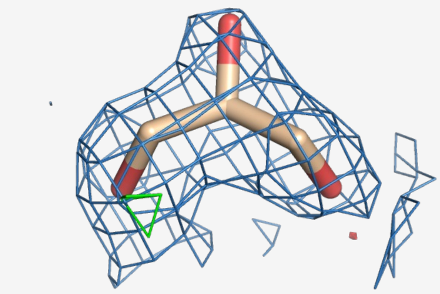

Small molecule 1: GLYCEROL (GOL) A 301 map cc 0.94

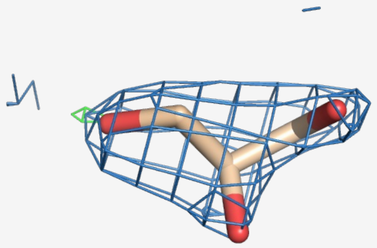

Small molecule 2: GLYCEROL (GOL) A 302 map cc 0.85

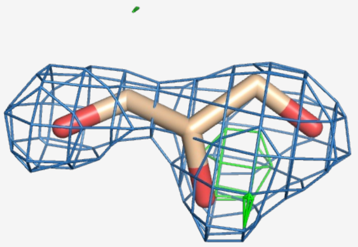

Small molecule 3: GLYCEROL (GOL) A 303 map cc 0.68

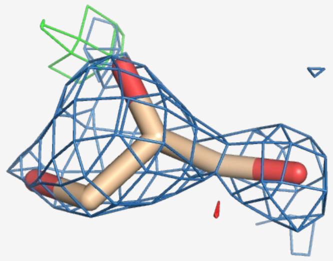

Small molecule 4: GLYCEROL (GOL) A 304 map cc 0.85

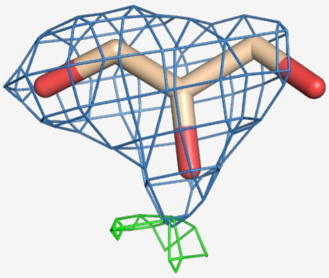

Small molecule 5: GLYCEROL (GOL) A 305 map cc 0.87

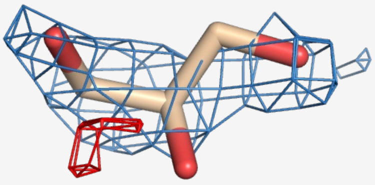

Small molecule 6: GLYCEROL (GOL) A 306 map cc 0.73

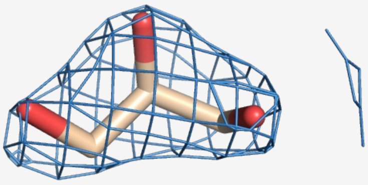

Small molecule 7: GLYCEROL (GOL) A 307 map cc 0.94

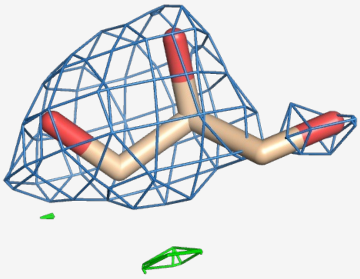

Small molecule 8: GLYCEROL (GOL) B 301 map cc 0.72

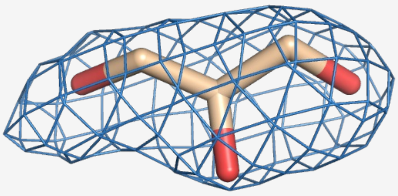

Small molecule 9: GLYCEROL (GOL) B 302 map cc 0.91

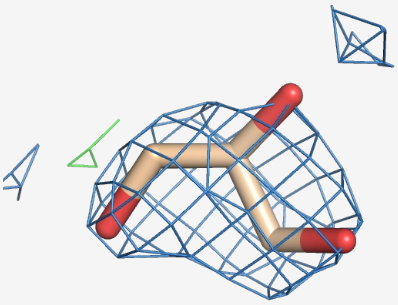

Small molecule 10: GLYCEROL (GOL) B 303 map cc 0.92

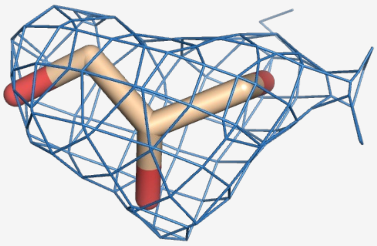

Small molecule 11: GLYCEROL (GOL) G 201 map cc 0.92

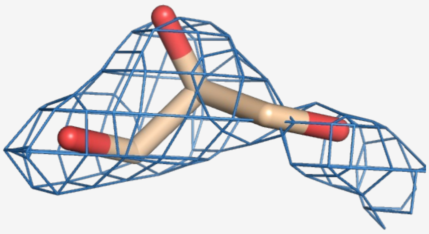

Small molecule 12: GLYCEROL (GOL) G 202 map cc 0.78

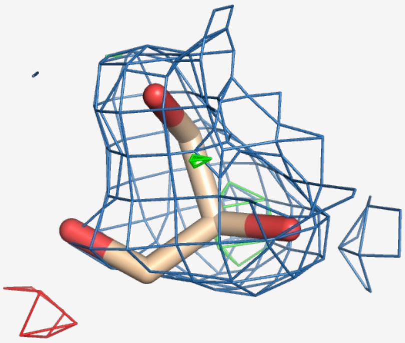

Small molecule 13: GLYCEROL (GOL) H 301 map cc 0.90

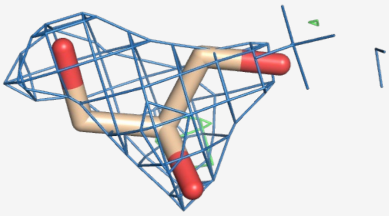

Small molecule 14: GLYCEROL (GOL) H 302 map cc 0.83

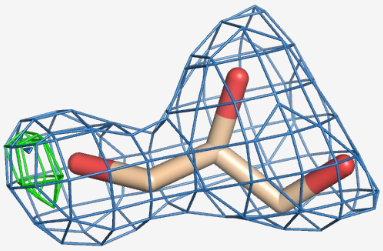

Small molecule 15: GLYCEROL (GOL) H 303 map cc 0.85

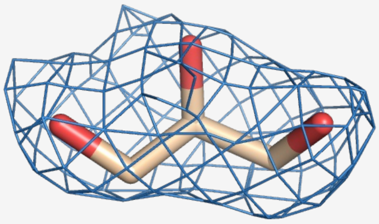

Small molecule 16: GLYCEROL (GOL) L 302 map cc 0.93

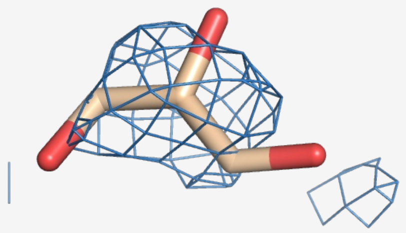

Small molecule 17: GLYCEROL (GOL) L 303 map cc 0.83

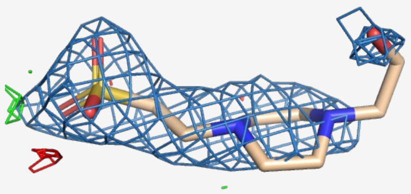

Small molecule 18: 4-(2-HYDROXYETHYL)-1-PIPERAZINEETHANESULFONICACID (EPE) L 301 map cc 0.93

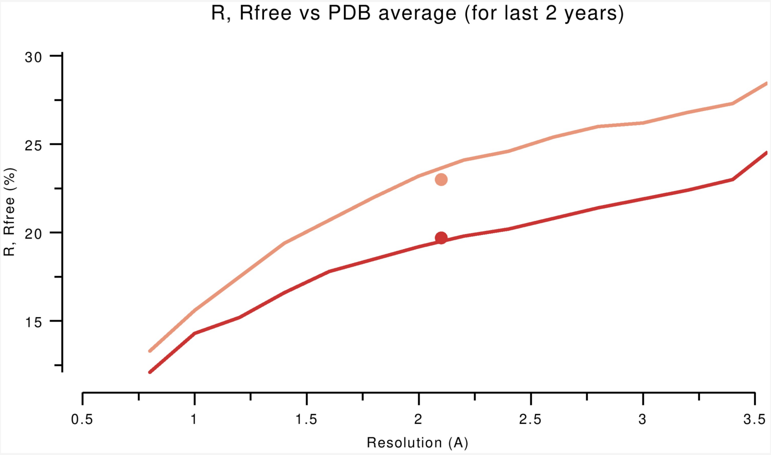

Credits:

HKL-3000
"Processing of X-ray Diffraction Data Collected in Oscillation Mode"
Z.Otwinowski, W.Minor
Methods in Enzymology, Volume 276: Macromolecular Crystallography, part A, p307-326 (1997)
"HKL-3000: the integration of data reduction and structure solution - from diffraction images to an initial model in minutes"
W.Minor, M.Cymborowski, Z.Otwinowski, M.Chruszcz
Acta Cryst. D62: 859-866 (2006)

CCP4 suite
"Overview of the CCP4 suite and current developments"
M.D.Winn et al.
Acta. Cryst. D67, 235-242 (2011)

Coot
"Features and Development of Coot"
P.Emsley, B.Lohkamp, W.Scott, and K.Cowtan
Acta Cryst. D66, 486-501 (2010)

Refmac5
"REFMAC5 for the refinement of macromolecular crystal structures"
G.N.Murshudov, P.Skubak, A.A.Lebedev, N.S.Pannu, R.A.Steiner, R.A.Nicholls, M.D.Winn, F.Long and A.A.Vagin
Acta Cryst. D67, 355-367 (2011)

Data collection and refinement statistics for project **hepes_5t6l** crystal **crystal1**

model **/home/asia/epe-like-validation/RESULTS/HKL_rerefine/most_twisted/5t6l/structure_mr/build_model_2/hkl_import.pdb** vs
model **/home/asia/epe-like-validation/RESULTS/HKL_rerefine/most_twisted/5t6l/structure_mr/build_model_2/hkl_refine_34.pdb_tls**

**Data collection**		
	**5T6L**	**Re-refinement**
Resolution (Å)	47.14 - 2.10 (2.15 - 2.10)	47.14 - 2.10
Wavelength (Å)	1.03314	
Space group	P21	P21
a, b, c (Å)	41.10, 158.97, 99.69	41.10, 158.97, 99.69
α, β, γ (°)	90, 97.98, 90	90, 97.98, 90
Completeness (%)	97.5 (91.9)	100.0 (100.0)
Reflections used	72210	
<I> / <Sigma I>	9.3 (1.6)	
Redundancy	2.4 (2.3)	
Rmerge	0.074	
Rpim		
CC1/2 last shell		
Wilson B factor (Å^2)	31.3	
**Refinement**		
Rwork / Rfree	0.178 / 0.218	0.199 / 0.237
Resolution (Å)	47.14 - 2.10	47.14 - 2.10
Reflections all	72183	72118
Reflections for Rfree	3620, 5.0%	3615, 5.0%
Bond lengths rmsd (Å)	0.008	0.013
Bond angles rmsd (°)	0.88	2.29
Mean B value (Å^2)	53	50
Number of protein atoms	9023	8878
Mean B value for protein atoms (Å^2)	53	50
Number of water atoms (expected)	405 (2846)	391 (2846)
Mean B value for water atoms (Å^2)	45	45
Number of ligand/ion atoms	117	117
Mean B value for ligand/ion atoms (Å^2)	66	67
Clashscore	4.70	3.13
Clashscore percentile (100)	-1	-1
Rotamer outliers (<1%)	3.62	2.63
Ramachandran outliers (<0.2%)	0.69	0.17
Ramachandran favored (>98%)	95.52	97.31
Residues with bad bonds (<0%)	0.05	0.91
Residues with bad angles (<0.1%)	0.10	3.04
MolProbity score	1.98	1.55

Map cc barchart:

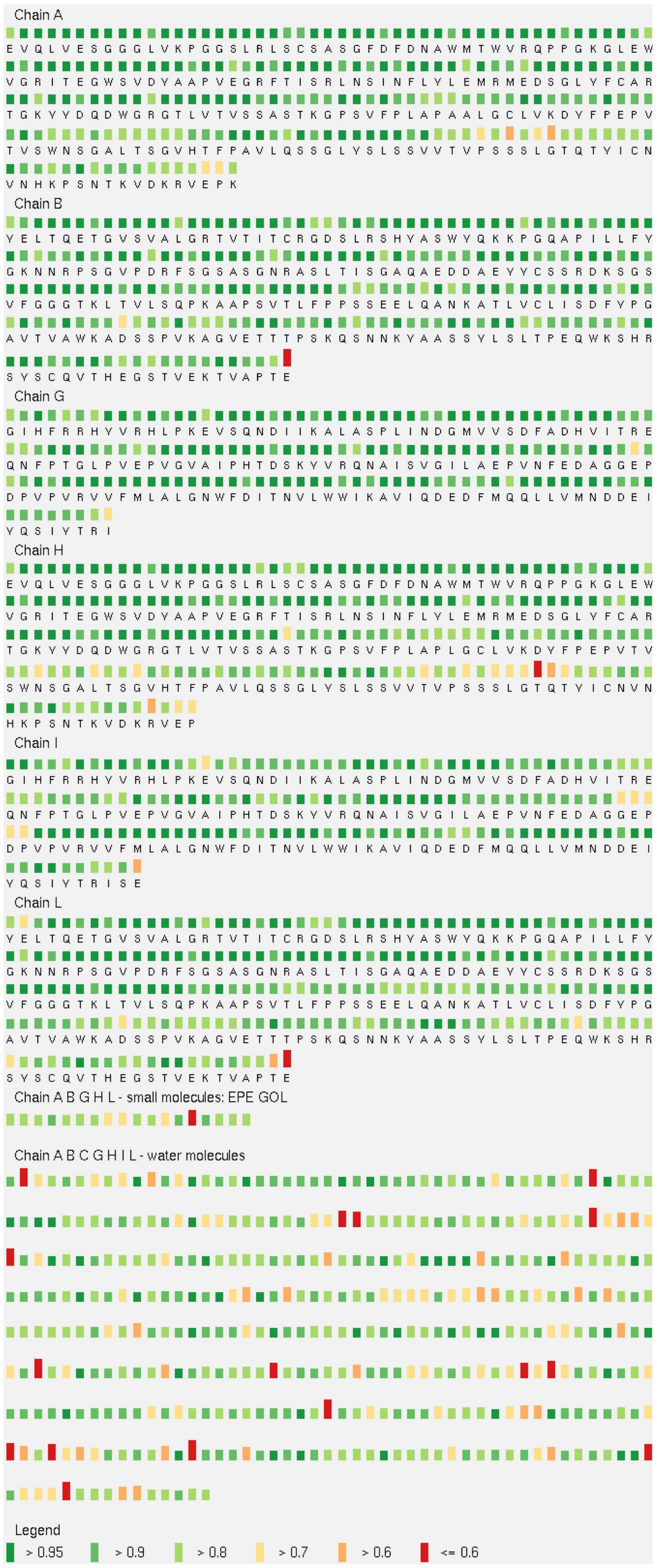

Small molecules with map:

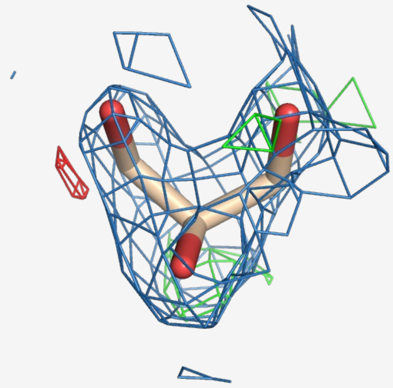

Small molecule 1: GLYCEROL (GOL) H 301 map cc 0.85

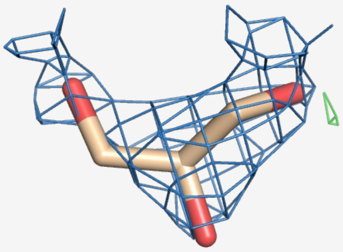

Small molecule 2: GLYCEROL (GOL) H 302 map cc 0.85

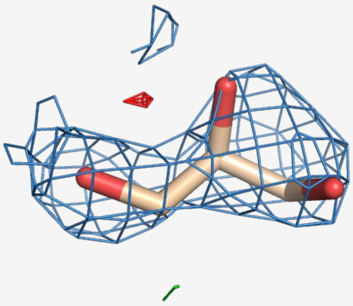

Small molecule 3: GLYCEROL (GOL) H 303 map cc 0.87

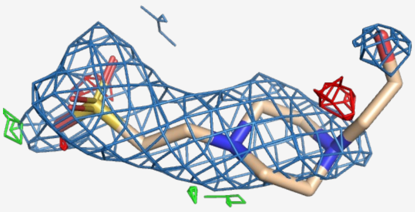

Small molecule 4: 4-(2-HYDROXYETHYL)-1-PIPERAZINEETHANESULFONICACID (EPE) L 301 map cc 0.91

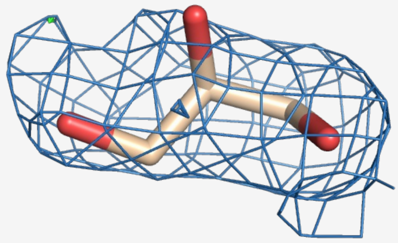

Small molecule 5: GLYCEROL (GOL) L 302 map cc 0.90

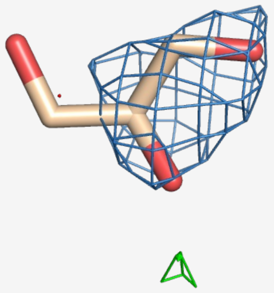

Small molecule 6: GLYCEROL (GOL) L 303 map cc 0.80

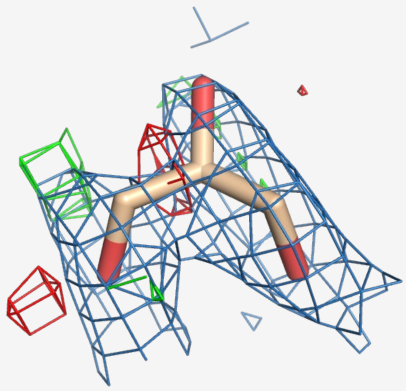

Small molecule 7: GLYCEROL (GOL) A 301 map cc 0.89

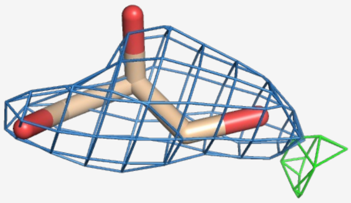

Small molecule 8: GLYCEROL (GOL) A 302 map cc 0.76

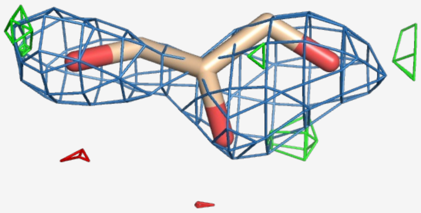

Small molecule 9: GLYCEROL (GOL) A 303 map cc 0.78

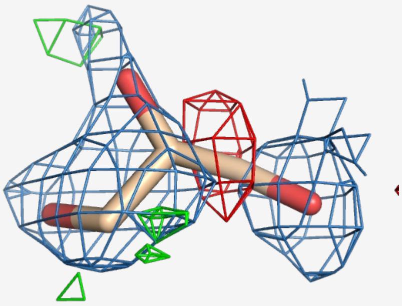

Small molecule 10: GLYCEROL (GOL) A 304 map cc 0.80

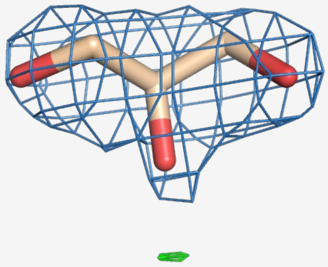

Small molecule 11: GLYCEROL (GOL) A 305 map cc 0.88

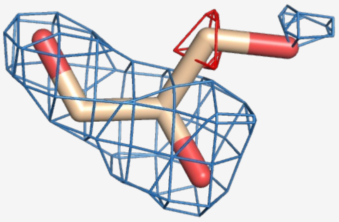

Small molecule 12: GLYCEROL (GOL) A 306 map cc 0.77

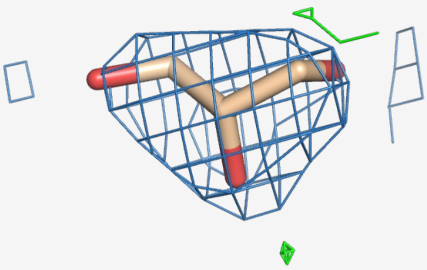

Small molecule 13: GLYCEROL (GOL) A 307 map cc 0.91

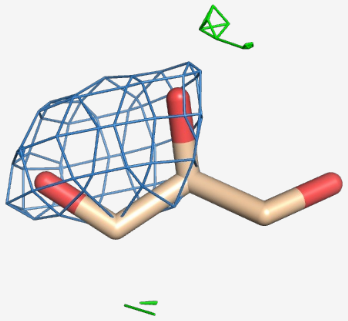

Small molecule 14: GLYCEROL (GOL) B 301 map cc 0.57

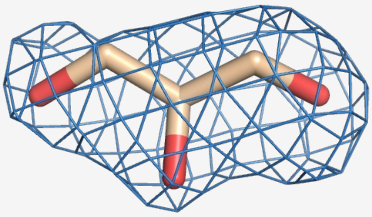

Small molecule 15: GLYCEROL (GOL) B 302 map cc 0.91

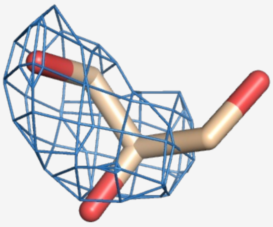

Small molecule 16: GLYCEROL (GOL) B 303 map cc 0.86

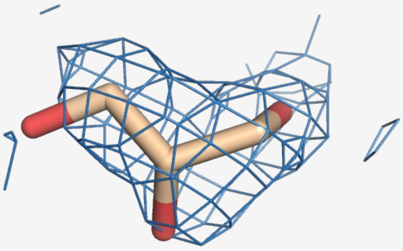

Small molecule 17: GLYCEROL (GOL) G 201 map cc 0.81

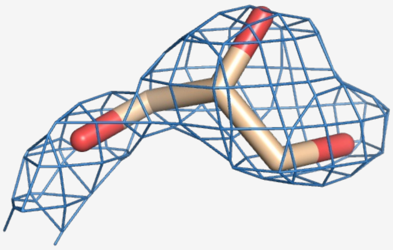

Small molecule 18: GLYCEROL (GOL) G 202 map cc 0.83

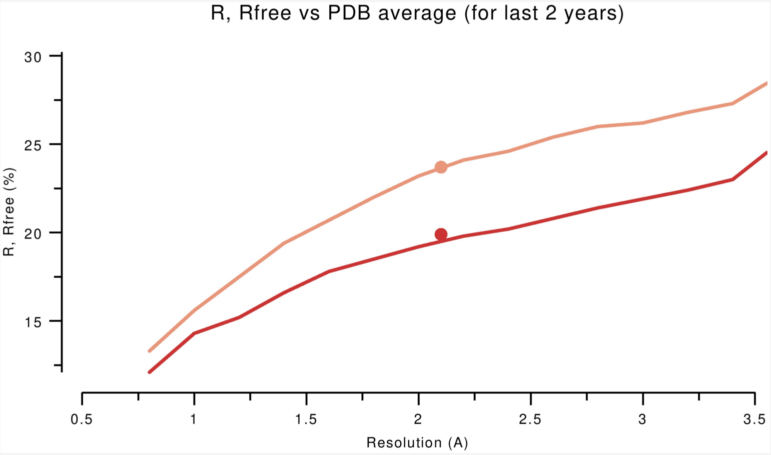

Credits:

HKL-3000
"Processing of X-ray Diffraction Data Collected in Oscillation Mode"
Z.Otwinowski, W.Minor
Methods in Enzymology, Volume 276: Macromolecular Crystallography, part A, p307-326 (1997)
"HKL-3000: the integration of data reduction and structure solution - from diffraction images to an initial model in minutes"
W.Minor, M.Cymborowski, Z.Otwinowski, M.Chruszcz
Acta Cryst. D62: 859-866 (2006)

CCP4 suite
"Overview of the CCP4 suite and current developments"
M.D.Winn et al.
Acta. Cryst. D67, 235-242 (2011)

Coot
"Features and Development of Coot"
P.Emsley, B.Lohkamp, W.Scott, and K.Cowtan
Acta Cryst. D66, 486-501 (2010)

Refmac5
"REFMAC5 for the refinement of macromolecular crystal structures"
G.N.Murshudov, P.Skubak, A.A.Lebedev, N.S.Pannu, R.A.Steiner, R.A.Nicholls, M.D.Winn, F.Long and A.A.Vagin
Acta Cryst. D67, 355-367 (2011)

Data collection and refinement statistics for project **hepes_6bb0** crystal **crystal1**

model **/home/asia/epe-like-validation/RESULTS/HKL_rerefine/most_twisted/6bb0/structure_mr/build_model_2/hkl_import.pdb** vs
model **/home/asia/epe-like-validation/RESULTS/HKL_rerefine/most_twisted/6bb0/structure_mr/build_model_2/hkl_refine_27.pdb**

**Data collection**		
	**6BB0**	**Re-refinement**
Resolution (Å)	39.00 - 1.95 (2.05 - 1.95)	47.01 - 1.95
Wavelength (Å)	0.9795	
Space group	P21	P21
a, b, c (Å)	78.12, 80.97, 103.21	78.12, 80.97, 103.21
α, β, γ (°)	90, 98.33, 90	90, 98.33, 90
Completeness (%)	97.8 (95.9)	97.9 (97.3)
Reflections used	91115	
<I> / <Sigma I>	16.6 (2.3)	
Redundancy	3.4 (3.4)	
Rmerge	0.046 (0.491)	
Rpim		
CC1/2 last shell		
Wilson B factor (Å^2)	32.1	
**Refinement**		
Rwork / Rfree	0.166 / 0.215	0.172 / 0.211
Resolution (Å)	38.65 - 1.95	38.68 - 1.95
Reflections all	89141	90971
Reflections for Rfree	1830, 2.0%	1830, 2.0%
Bond lengths rmsd (Å)	0.010	0.017
Bond angles rmsd (°)	1.51	1.94
Mean B value (Å^2)	38	36
Number of protein atoms	10132	10132
Mean B value for protein atoms (Å^2)	37	36
Number of water atoms (expected)	721 (1187)	721 (1187)
Mean B value for water atoms (Å^2)	37	38
Number of ligand/ion atoms	334	334
Mean B value for ligand/ion atoms (Å^2)	45	44
Clashscore	1.89	1.89
Clashscore percentile (100)	-1	-1
Rotamer outliers (<1%)	0.97	0.97
Ramachandran outliers (<0.2%)	0.16	0.16
Ramachandran favored (>98%)	98.05	98.05
Residues with bad bonds (<0%)	0.12	0.12
Residues with bad angles (<0.1%)	0.35	0.15
MolProbity score	0.95	0.95

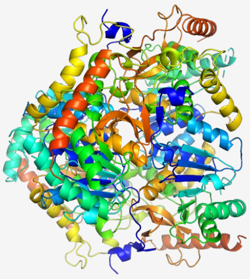

Map cc barchart:

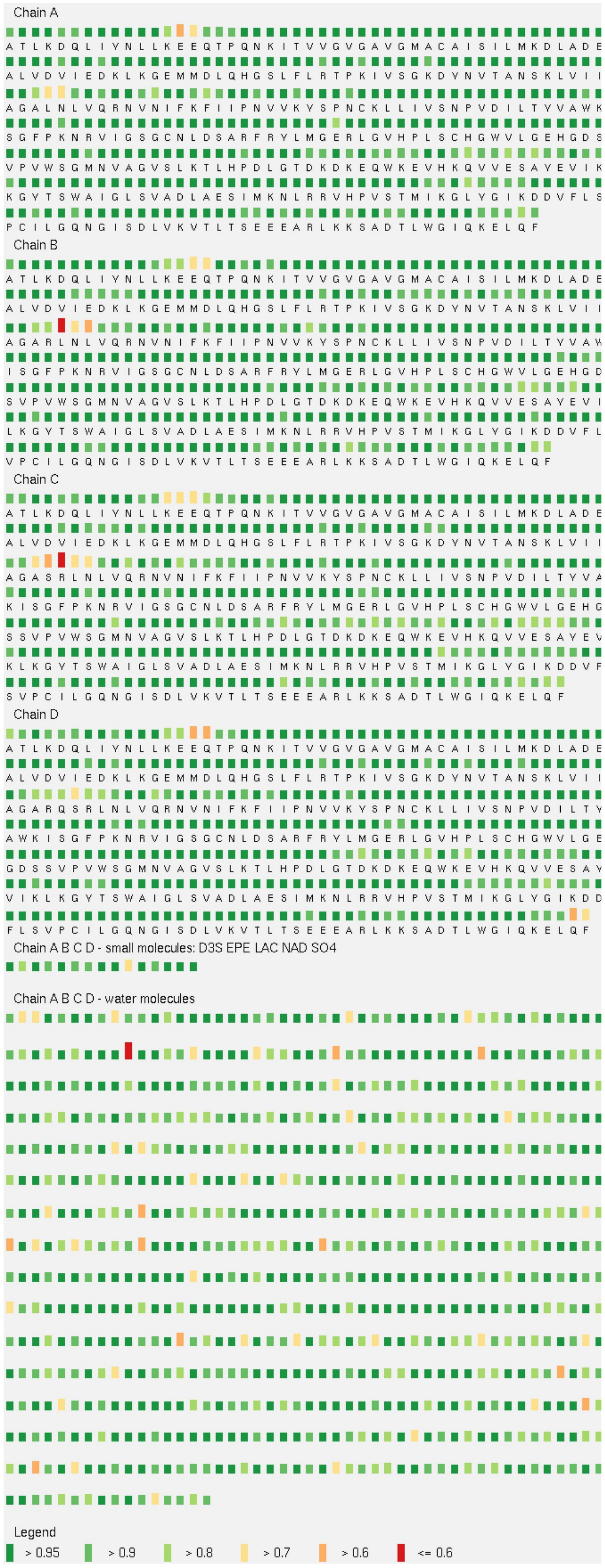

Small molecules with map:

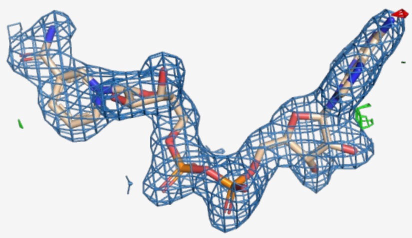

Small molecule 1: NICOTINAMIDE-ADENINE-DINUCLEOTIDE (NAD) A 801 map cc 0.96

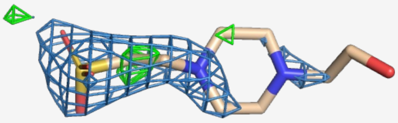

Small molecule 2: 4-(2-HYDROXYETHYL)-1-PIPERAZINEETHANESULFONICACID (EPE) A 802 map cc 0.88

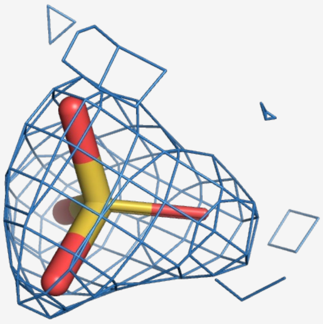

Small molecule 3: SULFATE ION (SO4) A 803 map cc 0.98

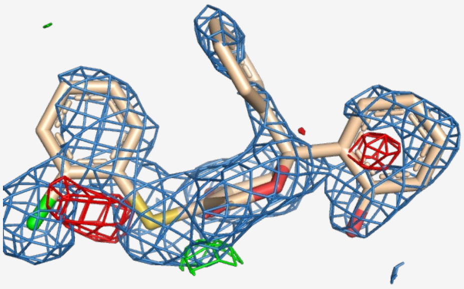

Small molecule 4: (6S)-3-[(2-chlorophenyl)sulfanyl]-4-hydroxy-6-(2-hydroxyphenyl)-6-phenyl-5,6-dihydro-2H-pyran-2-one (D3S) A 804 map cc 0.92

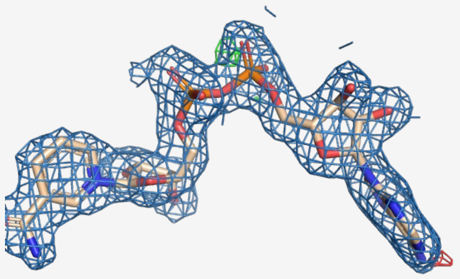

Small molecule 5: NICOTINAMIDE-ADENINE-DINUCLEOTIDE (NAD) B 801 map cc 0.98

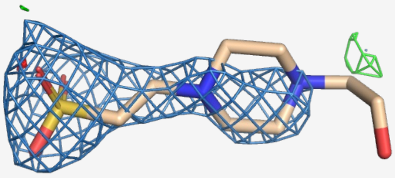

Small molecule 6: 4-(2-HYDROXYETHYL)-1-PIPERAZINEETHANESULFONICACID (EPE) B 802 map cc 0.93

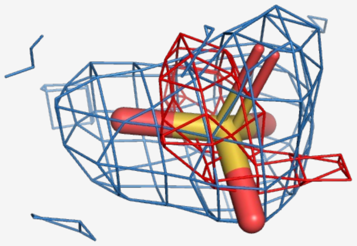

Small molecule 7: SULFATE ION (SO4) B 803 map cc 0.94

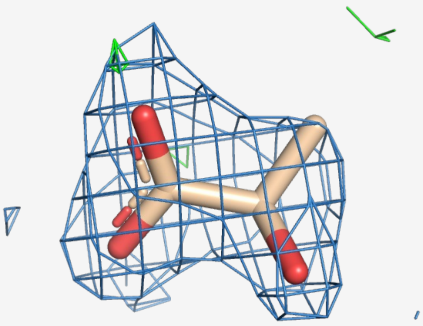

Small molecule 8: LACTIC ACID (LAC) B 804 map cc 0.95

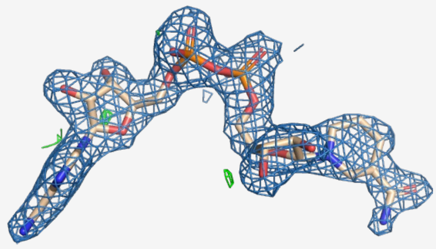

Small molecule 9: NICOTINAMIDE-ADENINE-DINUCLEOTIDE (NAD) C 801 map cc 0.96

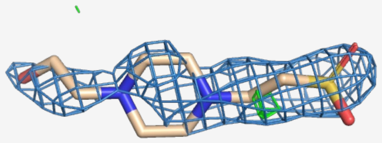

Small molecule 10: 4-(2-HYDROXYETHYL)-1-PIPERAZINEETHANESULFONICACID (EPE) C 802 map cc 0.78

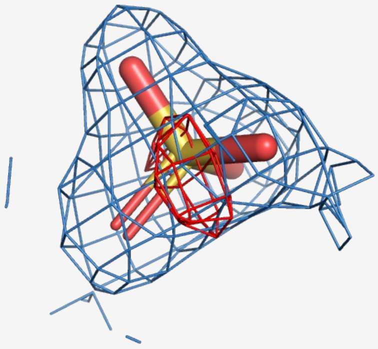

Small molecule 11: SULFATE ION (SO4) C 803 map cc 0.97

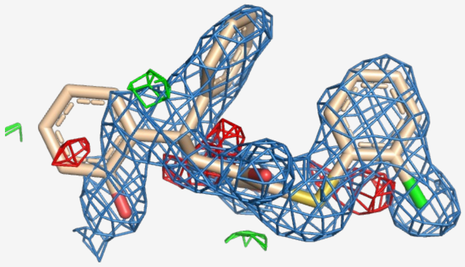

Small molecule 12: (6S)-3-[(2-chlorophenyl)sulfanyl]-4-hydroxy-6-(2-hydroxyphenyl)-6-phenyl-5,6-dihydro-2H-pyran-2-one (D3S) C 804 map cc 0.90

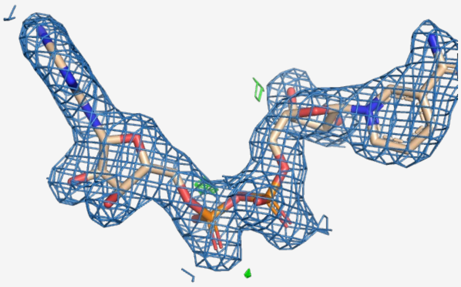

Small molecule 13: NICOTINAMIDE-ADENINE-DINUCLEOTIDE (NAD) D 801 map cc 0.98

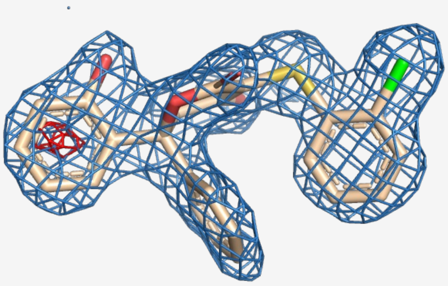

Small molecule 14: (6S)-3-[(2-chlorophenyl)sulfanyl]-4-hydroxy-6-(2-hydroxyphenyl)-6-phenyl-5,6-dihydro-2H-pyran-2-one (D3S) D 802 map cc 0.96

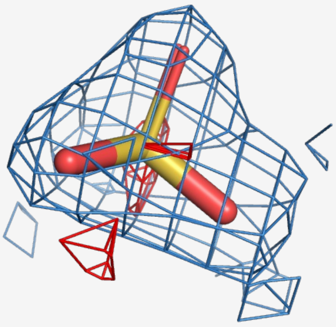

Small molecule 15: SULFATE ION (SO4) D 803 map cc 0.97

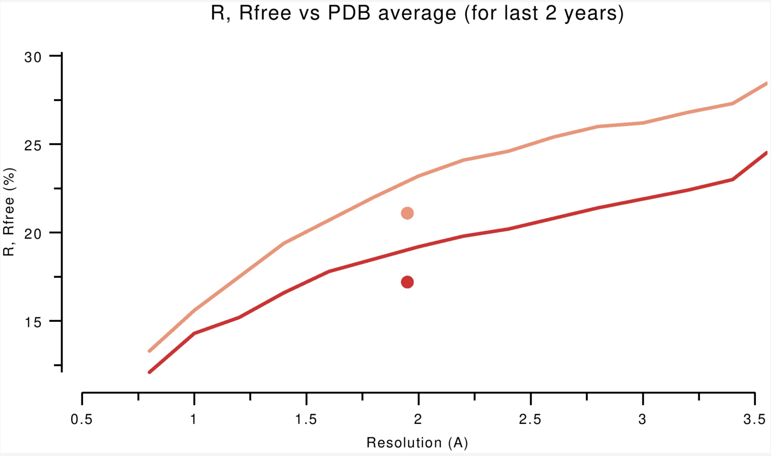

Credits:

HKL-3000
"Processing of X-ray Diffraction Data Collected in Oscillation Mode"
Z.Otwinowski, W.Minor
Methods in Enzymology, Volume 276: Macromolecular Crystallography, part A, p307-326 (1997)
"HKL-3000: the integration of data reduction and structure solution - from diffraction images to an initial model in minutes"
W.Minor, M.Cymborowski, Z.Otwinowski, M.Chruszcz
Acta Cryst. D62: 859-866 (2006)

CCP4 suite
"Overview of the CCP4 suite and current developments"
M.D.Winn et al.
Acta. Cryst. D67, 235-242 (2011)

Refmac5
"REFMAC5 for the refinement of macromolecular crystal structures"
G.N.Murshudov, P.Skubak, A.A.Lebedev, N.S.Pannu, R.A.Steiner, R.A.Nicholls, M.D.Winn, F.Long and A.A.Vagin
Acta Cryst. D67, 355-367 (2011)

Coot
"Features and Development of Coot"
P.Emsley, B.Lohkamp, W.Scott, and K.Cowtan
Acta Cryst. D66, 486-501 (2010)

Data collection and refinement statistics for project **hepes_6bb0** crystal **crystal1**

model **/home/asia/epe-like-validation/RESULTS/HKL_rerefine/most_twisted/6bb0/structure_mr/build_model_2/hkl_import.pdb** vs
model **/home/asia/epe-like-validation/RESULTS/HKL_rerefine/most_twisted/6bb0/structure_mr/build_model_2/hkl_refine_25.pdb_tls**

**Data collection**		
	**6BB0**	**Re-refinement**
Resolution (Å)	39.00 - 1.95 (2.05 - 1.95)	47.01 - 1.95
Wavelength (Å)	0.9795	
Space group	P21	P21
a, b, c (Å)	78.12, 80.97, 103.21	78.12, 80.97, 103.21
α, β, γ (°)	90, 98.33, 90	90, 98.33, 90
Completeness (%)	97.8 (95.9)	97.9 (97.3)
Reflections used	91115	
<I> / <Sigma I>	16.6 (2.3)	
Redundancy	3.4 (3.4)	
Rmerge	0.046 (0.491)	
Rpim		
CC1/2 last shell		
Wilson B factor (Å²)	32.1	
**Refinement**		
Rwork / Rfree	0.166 / 0.215	0.162 / 0.205
Resolution (Å)	38.65 - 1.95	38.68 - 1.95
Reflections all	89141	90971
Reflections for Rfree	1830, 2.0%	1830, 2.0%
Bond lengths rmsd (Å)	0.010	0.013
Bond angles rmsd (°)	1.51	1.80
Mean B value (Å²)	38	37
Number of protein atoms	10132	10113
Mean B value for protein atoms (Å²)	37	37
Number of water atoms (expected)	721 (1187)	724 (1187)
Mean B value for water atoms (Å²)	37	39
Number of ligand/ion atoms	334	334
Mean B value for ligand/ion atoms (Å²)	45	44
Clashscore	1.89	2.70
Clashscore percentile (100)	-1	-1
Rotamer outliers (<1%)	0.97	1.41
Ramachandran outliers (<0.2%)	0.16	0.08
Ramachandran favored (>98%)	98.05	97.66
Residues with bad bonds (<0%)	0.12	0.38
Residues with bad angles (<0.1%)	0.35	1.31
MolProbity score	0.95	1.24

Map cc barchart:

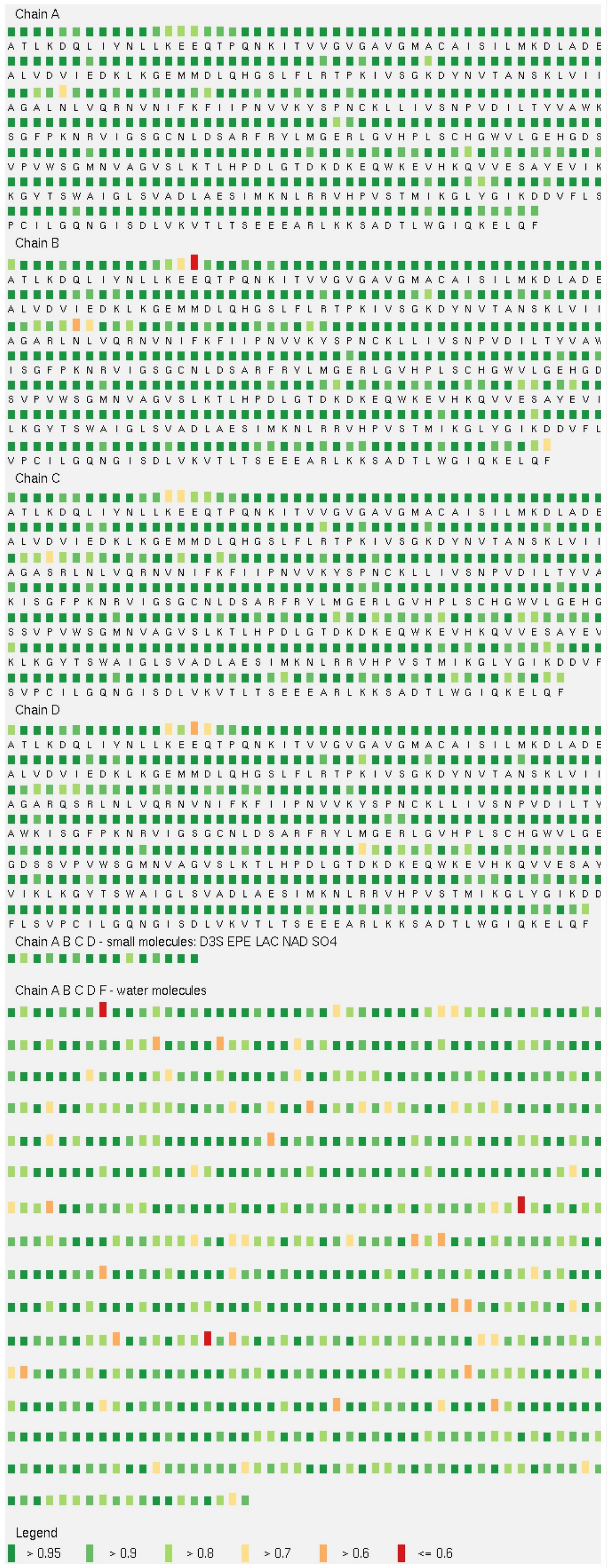

Small molecules with map:

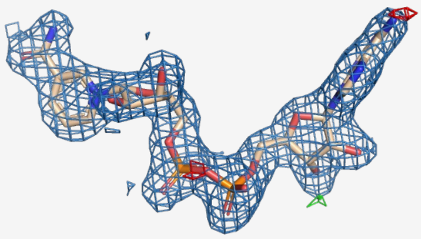

Small molecule 1: NICOTINAMIDE-ADENINE-DINUCLEOTIDE (NAD) A 801 map cc 0.97

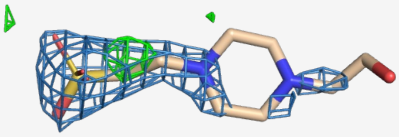

Small molecule 2: 4-(2-HYDROXYETHYL)-1-PIPERAZINEETHANESULFONICACID (EPE) A 802 map cc 0.90

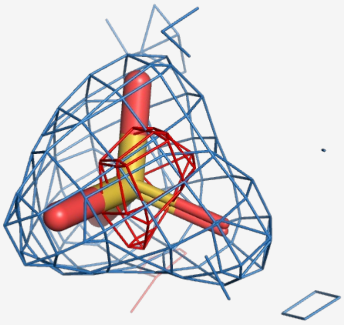

Small molecule 3: SULFATE ION (SO4) A 803 map cc 0.98

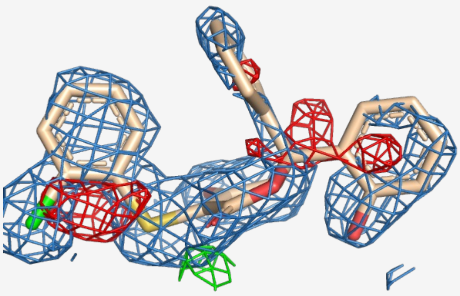

Small molecule 4: (6S)-3-[(2-chlorophenyl)sulfanyl]-4-hydroxy-6-(2-hydroxyphenyl)-6-phenyl-5,6-dihydro-2H-pyran-2-one (D3S) A 804 map cc 0.90

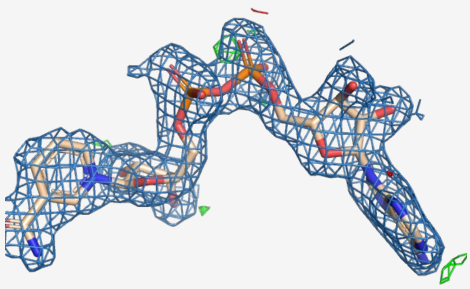

Small molecule 5: NICOTINAMIDE-ADENINE-DINUCLEOTIDE (NAD) B 801 map cc 0.98

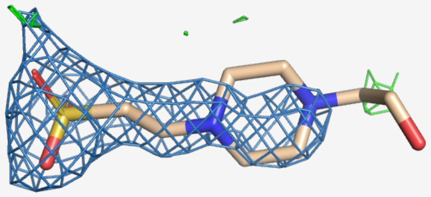

Small molecule 6: 4-(2-HYDROXYETHYL)-1-PIPERAZINEETHANESULFONICACID (EPE) B 802 map cc 0.93

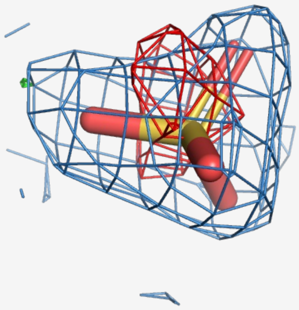

Small molecule 7: SULFATE ION (SO4) B 803 map cc 0.96

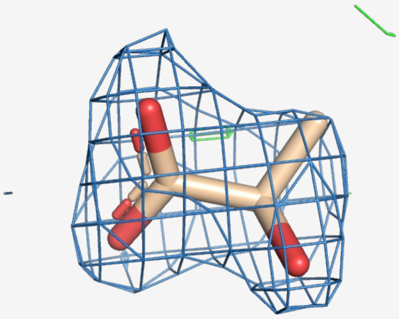

Small molecule 8: LACTIC ACID (LAC) B 804 map cc 0.97

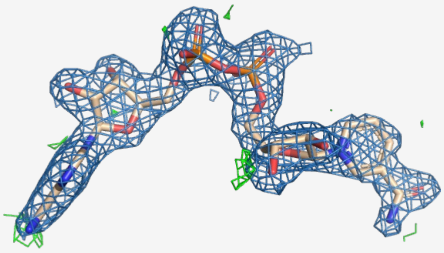

Small molecule 9: NICOTINAMIDE-ADENINE-DINUCLEOTIDE (NAD) C 801 map cc 0.96

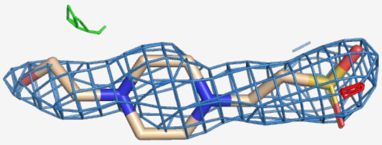

Small molecule 10: 4-(2-HYDROXYETHYL)-1-PIPERAZINEETHANESULFONICACID (EPE) C 802 map cc 0.87

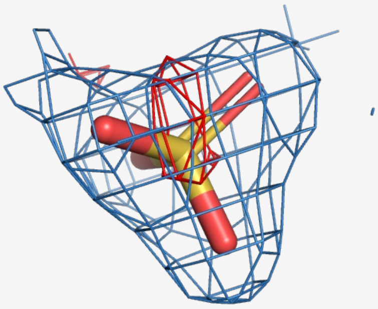

Small molecule 11: SULFATE ION (SO4) C 803 map cc 0.97

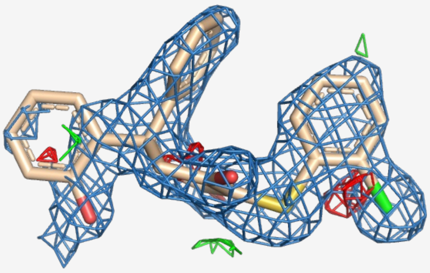

Small molecule 12: (6S)-3-[(2-chlorophenyl)sulfanyl]-4-hydroxy-6-(2-hydroxyphenyl)-6-phenyl-5,6-dihydro-2H-pyran-2-one (D3S) C 804 map cc 0.92

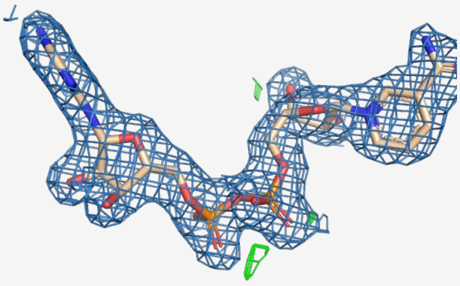

Small molecule 13: NICOTINAMIDE-ADENINE-DINUCLEOTIDE (NAD) D 801 map cc 0.98

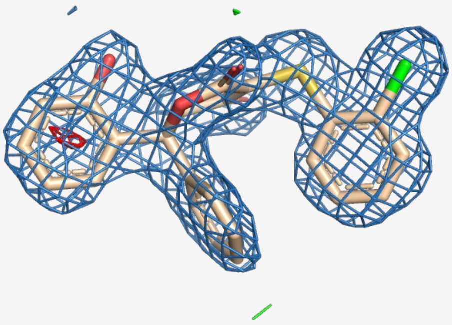

Small molecule 14: (6S)-3-[(2-chlorophenyl)sulfanyl]-4-hydroxy-6-(2-hydroxyphenyl)-6-phenyl-5,6-dihydro-2H-pyran-2-one (D3S) D 802 map cc 0.96

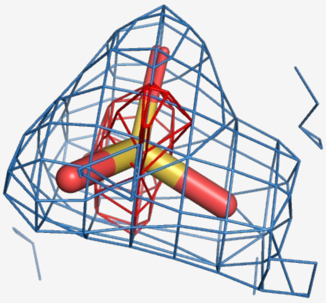

Small molecule 15: SULFATE ION (SO4) D 803 map cc 0.97

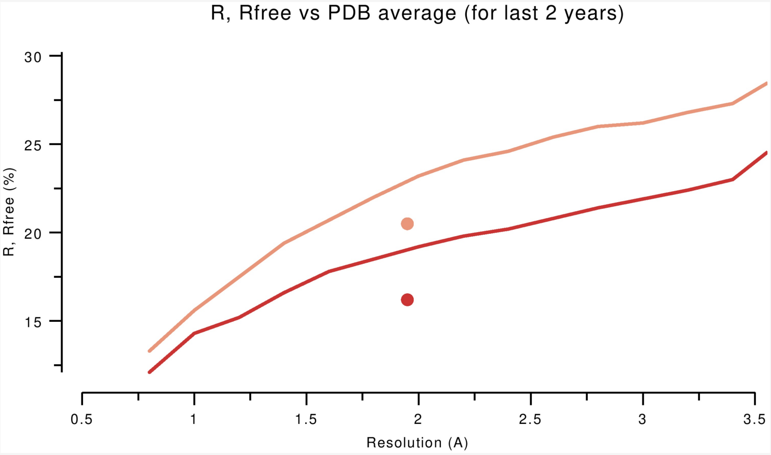

Credits:

HKL-3000
"Processing of X-ray Diffraction Data Collected in Oscillation Mode"
Z.Otwinowski, W.Minor
Methods in Enzymology, Volume 276: Macromolecular Crystallography, part A, p307-326 (1997)
"HKL-3000: the integration of data reduction and structure solution - from diffraction images to an initial model in minutes"
W.Minor, M.Cymborowski, Z.Otwinowski, M.Chruszcz
Acta Cryst. D62: 859-866 (2006)

CCP4 suite
"Overview of the CCP4 suite and current developments"
M.D.Winn et al.
Acta. Cryst. D67, 235-242 (2011)

Refmac5
"REFMAC5 for the refinement of macromolecular crystal structures"
G.N.Murshudov, P.Skubak, A.A.Lebedev, N.S.Pannu, R.A.Steiner, R.A.Nicholls, M.D.Winn, F.Long and A.A.Vagin
Acta Cryst. D67, 355-367 (2011)

Coot
"Features and Development of Coot"
P.Emsley, B.Lohkamp, W.Scott, and K.Cowtan
Acta Cryst. D66, 486-501 (2010)

Data collection and refinement statistics for project **mes_3o4p** crystal **crystal1**

model **/home/asia/epe-like-validation/RESULTS/HKL_rerefine/highres/3o4p/structure_mr/build_model_2/hkl_import.pdb** vs
model **/home/asia/epe-like-validation/RESULTS/HKL_rerefine/highres/3o4p/structure_mr/build_model_2/hkl_refine_496.pdb**

Data collection		
	**3O4P**	**Re-refinement**
Resolution (Å)	20.80 - 0.85 (-)	20.92 - 0.83
Wavelength (Å)	0.842	
Space group	P212121	P212121
a, b, c (Å)	43.11, 81.85, 86.47	43.11, 81.85, 86.47
α, β, γ (°)	90, 90, 90	90, 90, 90
Completeness (%)	93.8	100.0 (100.0)
Reflections used	264548	
<I> / <Sigma I>		
Redundancy		
Rmerge		
Rpim		
CC1/2 last shell		
Wilson B factor (Å²)	5.9	
**Refinement**		
Rwork / Rfree	0.103 / 0.121	0.123 / 0.136
Resolution (Å)	20.80 - 0.85	20.92 - 0.83
Reflections all	241251	255775
Reflections for Rfree	2463, %	2584, 1.0%
Bond lengths rmsd (Å)		0.037
Bond angles rmsd (°)		3.60
Mean B value (Å²)	12	12
Number of protein atoms	2657	2657
Mean B value for protein atoms (Å²)	10	9
Number of water atoms (expected)	481 (572)	481 (572)
Mean B value for water atoms (Å²)	21	21
Number of ligand/ion atoms	136	136
Mean B value for ligand/ion atoms (Å²)	29	30
Clashscore	29.10	28.22
Clashscore percentile (100)	-1	-1
Rotamer outliers (<1%)	1.30	1.30
Ramachandran outliers (<0.2%)	0.32	0.32
Ramachandran favored (>98%)	96.15	96.15
Residues with bad bonds (<0%)	5.69	5.53
Residues with bad angles (<0.1%)	9.13	8.97
MolProbity score	2.30	2.29

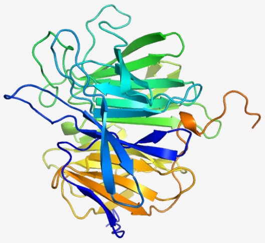

Map cc barchart:

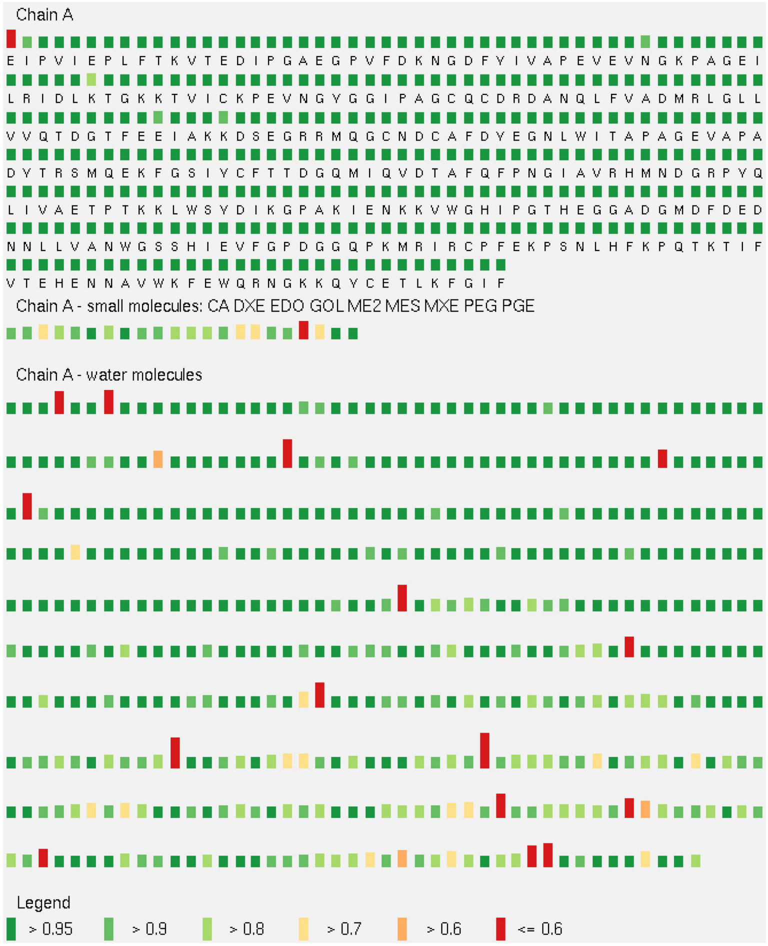

Small molecules with map:

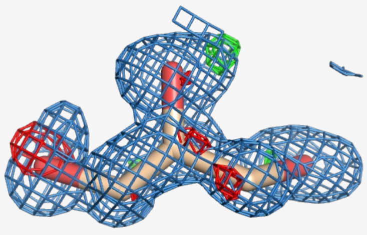

Small molecule 1: GLYCEROL (GOL) A 401 map cc 0.95

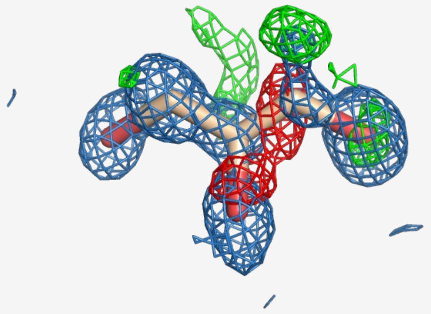

Small molecule 2: GLYCEROL (GOL) A 403 map cc 0.91

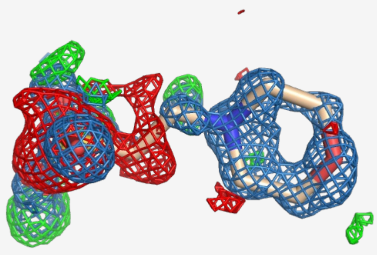

Small molecule 3: 2-(N-MORPHOLINO)-ETHANESULFONIC ACID (MES) A 411 map cc 0.80

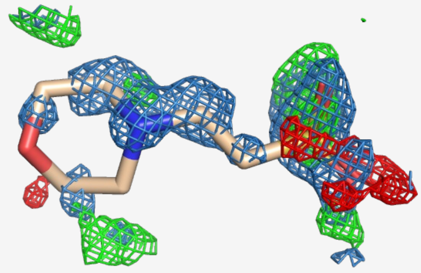

Small molecule 4: 2-(N-MORPHOLINO)-ETHANESULFONIC ACID (MES) A 412 map cc 0.82

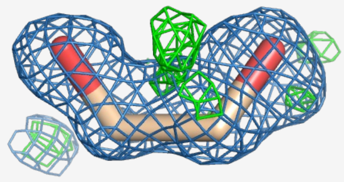

Small molecule 5: 1,2-ETHANEDIOL (EDO) A 421 map cc 0.93

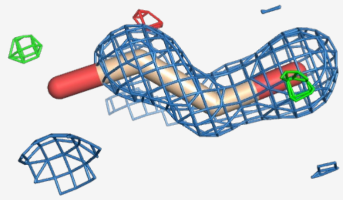

Small molecule 6: 1,2-ETHANEDIOL (EDO) A 422 map cc 0.95

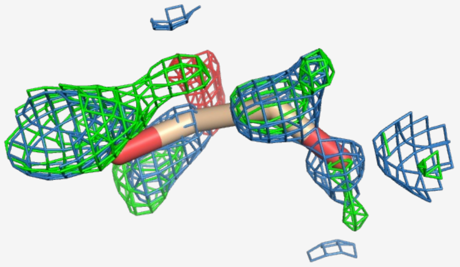

Small molecule 7: 1,2-ETHANEDIOL (EDO) A 423 map cc 0.81

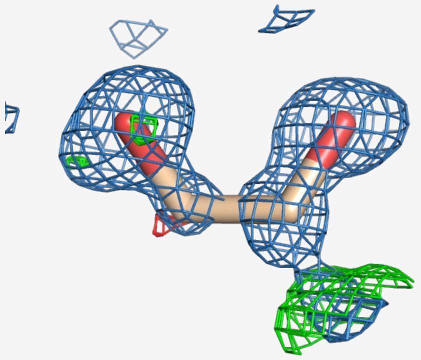

Small molecule 8: 1,2-ETHANEDIOL (EDO) A 424 map cc 0.95

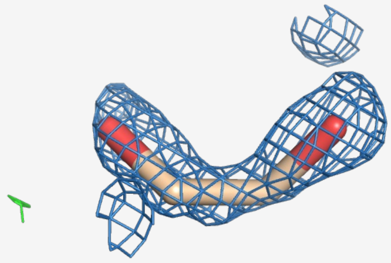

Small molecule 9: 1,2-ETHANEDIOL (EDO) A 425 map cc 0.93

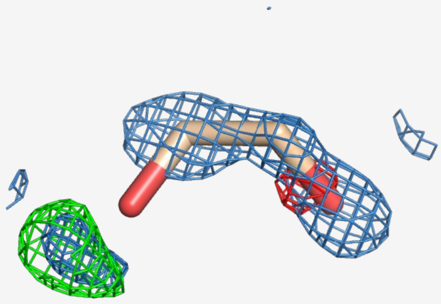

Small molecule 10: 1,2-ETHANEDIOL (EDO) A 426 map cc 0.93

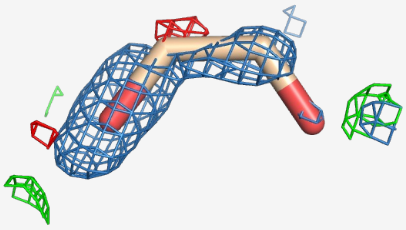

Small molecule 11: 1,2-ETHANEDIOL (EDO) A 427 map cc 0.88

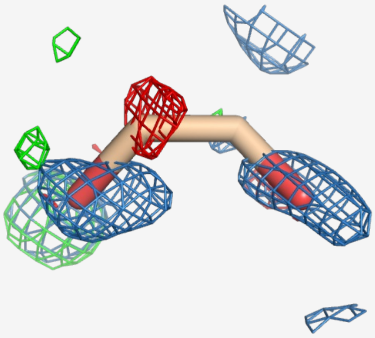

Small molecule 12: 1,2-ETHANEDIOL (EDO) A 428 map cc 0.90

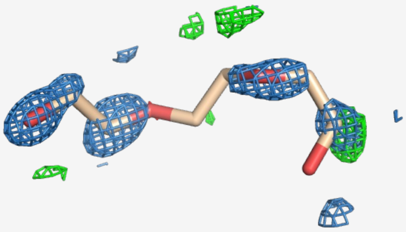

Small molecule 13: TRIETHYLENE GLYCOL (PGE) A 433 map cc 0.88

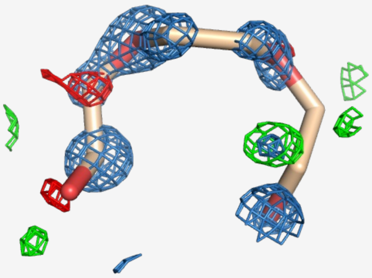

Small molecule 14: TRIETHYLENE GLYCOL (PGE) A 434 map cc 0.92

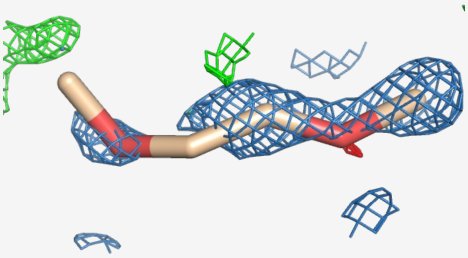

Small molecule 15: 1,2-DIMETHOXYETHANE (DXE) A 442 map cc 0.77

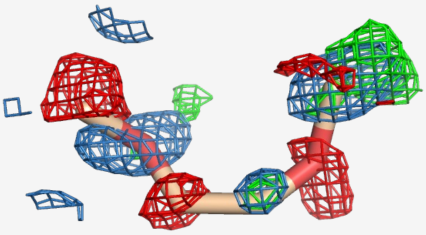

Small molecule 16: 1,2-DIMETHOXYETHANE (DXE) A 443 map cc 0.77

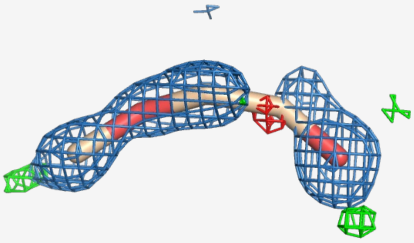

Small molecule 17: 2-METHOXYETHANOL (MXE) A 451 map cc 0.93

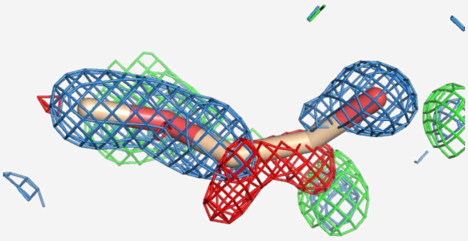

Small molecule 18: 2-METHOXYETHANOL (MXE) A 452 map cc 0.95

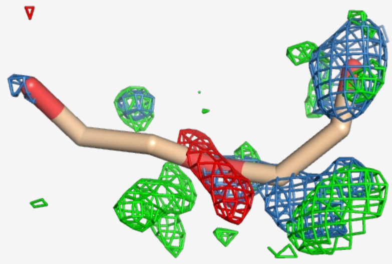

Small molecule 19: DI(HYDROXYETHYL)ETHER (PEG) A 461 map cc 0.56

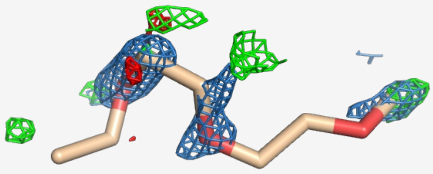

Small molecule 20: 1-ETHOXY-2-(2-METHOXYETHOXY)ETHANE (ME2) A 471 map cc 0.75

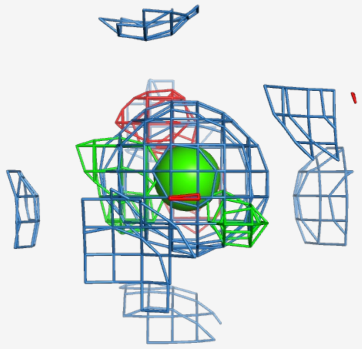

Small molecule 21: CALCIUM ION (CA) A 491 map cc 1.00

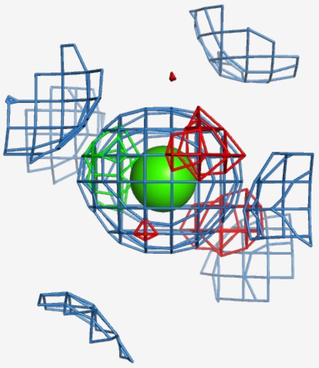

Small molecule 22: CALCIUM ION (CA) A 492 map cc 1.00

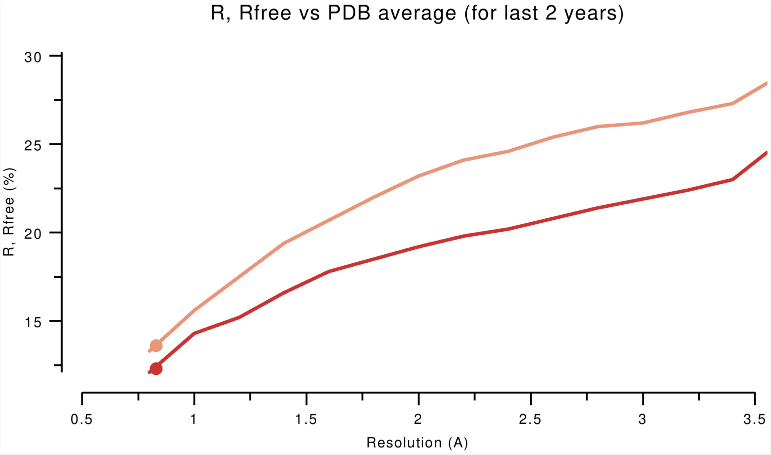

Credits:

HKL-3000
"Processing of X-ray Diffraction Data Collected in Oscillation Mode"
Z.Otwinowski, W.Minor
Methods in Enzymology, Volume 276: Macromolecular Crystallography, part A, p307-326 (1997)
"HKL-3000: the integration of data reduction and structure solution - from diffraction images to an initial model in minutes"
W.Minor, M.Cymborowski, Z.Otwinowski, M.Chruszcz
Acta Cryst. D62: 859-866 (2006)

CCP4 suite
"Overview of the CCP4 suite and current developments"
M.D.Winn et al.
Acta. Cryst. D67, 235-242 (2011)

Refmac5
"REFMAC5 for the refinement of macromolecular crystal structures"
G.N.Murshudov, P.Skubak, A.A.Lebedev, N.S.Pannu, R.A.Steiner, R.A.Nicholls, M.D.Winn, F.Long and A.A.Vagin
Acta Cryst. D67, 355-367 (2011)

Coot
"Features and Development of Coot"
P.Emsley, B.Lohkamp, W.Scott, and K.Cowtan
Acta Cryst. D66, 486-501 (2010)

Data collection and refinement statistics for project **mes_3o4p** crystal **crystal1**

model **/home/asia/epe-like-validation/RESULTS/HKL_rerefine/highres/3o4p/structure_mr/build_model_2/hkl_import.pdb** vs
model **/home/asia/epe-like-validation/RESULTS/HKL_rerefine/highres/3o4p/structure_mr/build_model_2/hkl_refine_501.pdb**

Data collection		
	**3O4P**	**Re-refinement**
Resolution (Å)	20.80 - 0.85 (-)	20.92 - 0.83
Wavelength (Å)	0.842	
Space group	P212121	P212121
a, b, c (Å)	43.11, 81.85, 86.47	43.11, 81.85, 86.47
α, β, γ (°)	90, 90, 90	90, 90, 90
Completeness (%)	93.8	100.0 (100.0)
Reflections used	264548	
<I> / <Sigma I>		
Redundancy		
Rmerge		
Rpim		
CC1/2 last shell		
Wilson B factor (Å²)	5.9	
**Refinement**		
Rwork / Rfree	0.103 / 0.121	0.120 / 0.131
Resolution (Å)	20.80 - 0.85	20.92 - 0.83
Reflections all	241251	255775
Reflections for Rfree	2463, %	2584, 1.0%
Bond lengths rmsd (Å)		0.035
Bond angles rmsd (°)		2.50
Mean B value (Å²)	12	11
Number of protein atoms	2657	2638
Mean B value for protein atoms (Å²)	10	8
Number of water atoms (expected)	481 (572)	484 (572)
Mean B value for water atoms (Å²)	21	19
Number of ligand/ion atoms	136	129
Mean B value for ligand/ion atoms (Å²)	29	24
Clashscore	29.10	18.70
Clashscore percentile (100)	-1	-1
Rotamer outliers (<1%)	1.30	1.63
Ramachandran outliers (<0.2%)	0.32	0.00
Ramachandran favored (>98%)	96.15	96.14
Residues with bad bonds (<0%)	5.69	1.07
Residues with bad angles (<0.1%)	9.13	4.70
MolProbity score	2.30	2.19

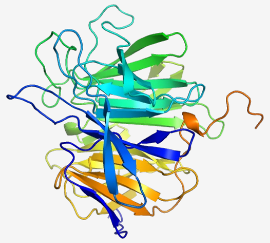

Map cc barchart:

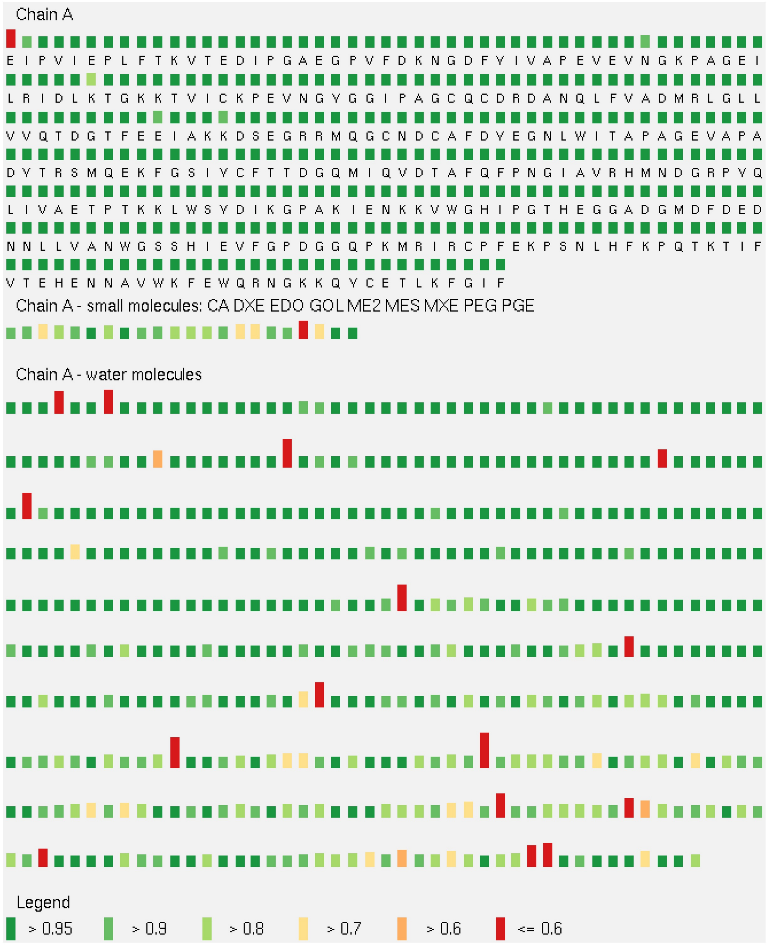

Small molecules with map:

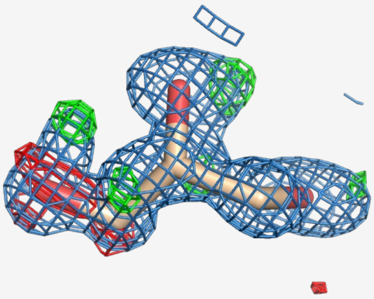

Small molecule 1: GLYCEROL (GOL) A 401 map cc 0.95

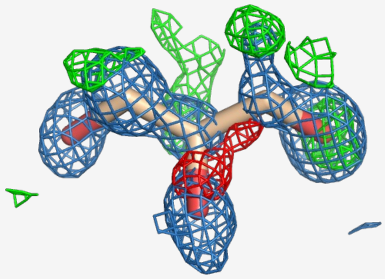

Small molecule 2: GLYCEROL (GOL) A 403 map cc 0.91

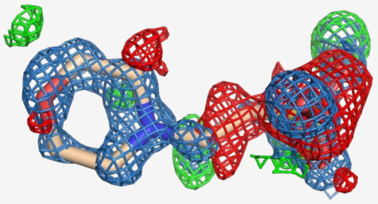

Small molecule 3: 2-(N-MORPHOLINO)-ETHANESULFONIC ACID (MES) A 411 map cc 0.80

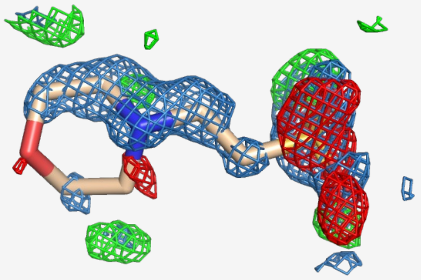

Small molecule 4: 2-(N-MORPHOLINO)-ETHANESULFONIC ACID (MES) A 412 map cc 0.82

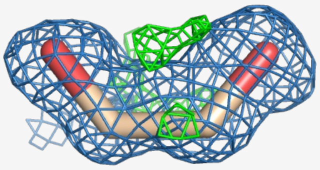

Small molecule 5: 1,2-ETHANEDIOL (EDO) A 421 map cc 0.93

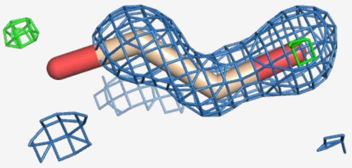

Small molecule 6: 1,2-ETHANEDIOL (EDO) A 422 map cc 0.95

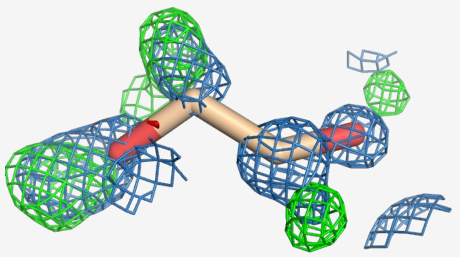

Small molecule 7: 1,2-ETHANEDIOL (EDO) A 423 map cc 0.81

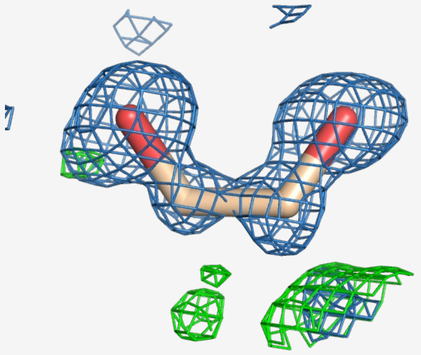

Small molecule 8: 1,2-ETHANEDIOL (EDO) A 424 map cc 0.95

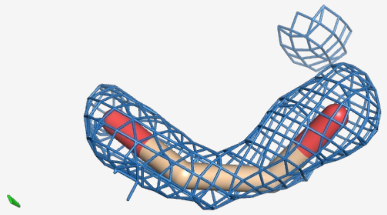

Small molecule 9: 1,2-ETHANEDIOL (EDO) A 425 map cc 0.93

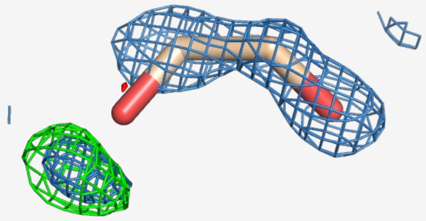

Small molecule 10: 1,2-ETHANEDIOL (EDO) A 426 map cc 0.93

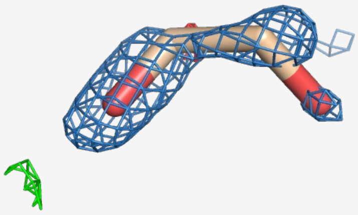

Small molecule 11: 1,2-ETHANEDIOL (EDO) A 427 map cc 0.88

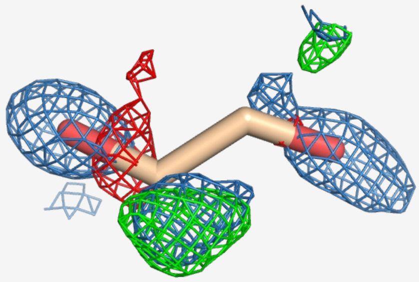

Small molecule 12: 1,2-ETHANEDIOL (EDO) A 428 map cc 0.90

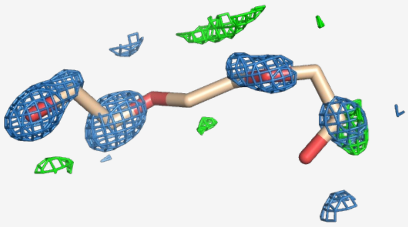

Small molecule 13: TRIETHYLENE GLYCOL (PGE) A 433 map cc 0.88

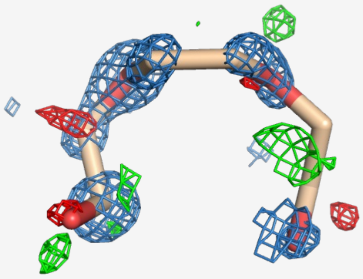

Small molecule 14: TRIETHYLENE GLYCOL (PGE) A 434 map cc 0.92

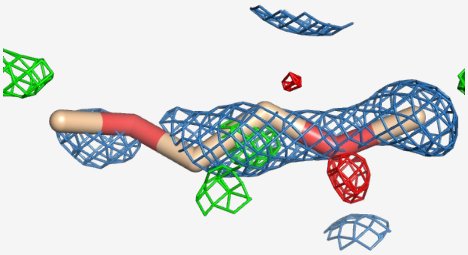

Small molecule 15: 1,2-DIMETHOXYETHANE (DXE) A 442 map cc 0.77

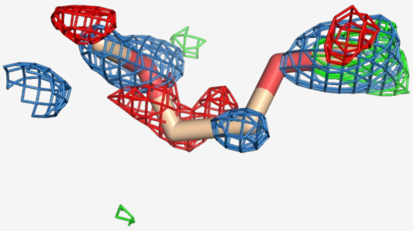

Small molecule 16: 1,2-DIMETHOXYETHANE (DXE) A 443 map cc 0.77

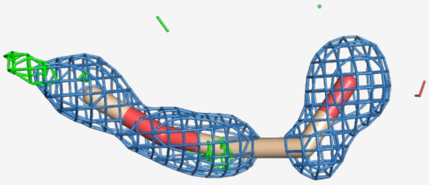

Small molecule 17: 2-METHOXYETHANOL (MXE) A 451 map cc 0.93

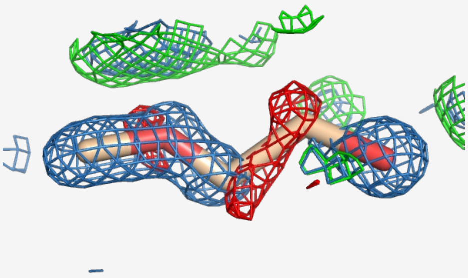

Small molecule 18: 2-METHOXYETHANOL (MXE) A 452 map cc 0.95

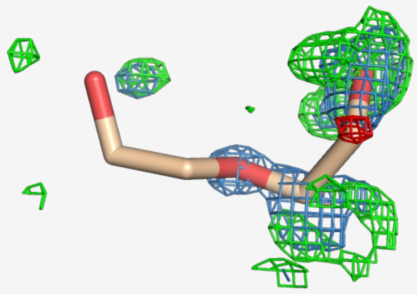

Small molecule 19: DI(HYDROXYETHYL)ETHER (PEG) A 461 map cc 0.56

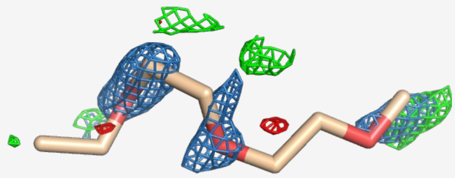

Small molecule 20: 1-ETHOXY-2-(2-METHOXYETHOXY)ETHANE (ME2) A 471 map cc 0.75

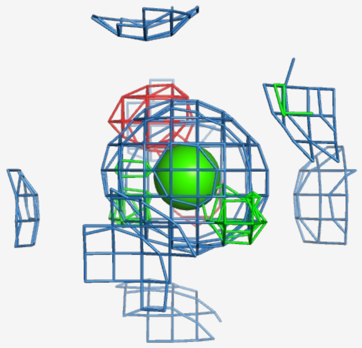

Small molecule 21: CALCIUM ION (CA) A 491 map cc 1.00

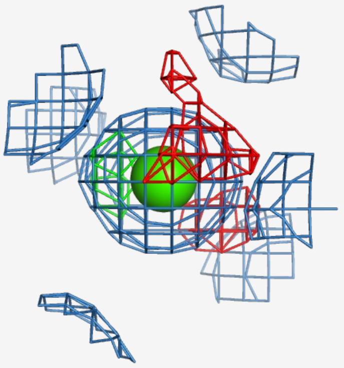

Small molecule 22: CALCIUM ION (CA) A 492 map cc 1.00

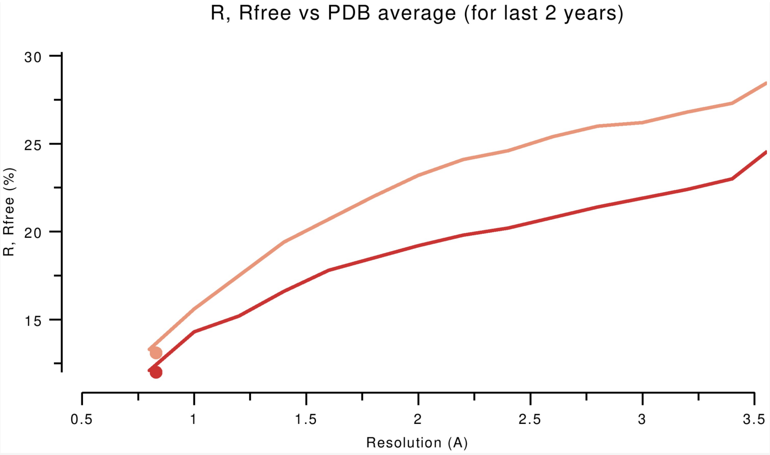

Credits:

HKL-3000
"Processing of X-ray Diffraction Data Collected in Oscillation Mode"
Z.Otwinowski, W.Minor
Methods in Enzymology, Volume 276: Macromolecular Crystallography, part A, p307-326 (1997)
"HKL-3000: the integration of data reduction and structure solution - from diffraction images to an initial model in minutes"
W.Minor, M.Cymborowski, Z.Otwinowski, M.Chruszcz
Acta Cryst. D62: 859-866 (2006)

CCP4 suite
"Overview of the CCP4 suite and current developments"
M.D.Winn et al.
Acta. Cryst. D67, 235-242 (2011)

Refmac5
"REFMAC5 for the refinement of macromolecular crystal structures"
G.N.Murshudov, P.Skubak, A.A.Lebedev, N.S.Pannu, R.A.Steiner, R.A.Nicholls, M.D.Winn, F.Long and A.A.Vagin
Acta Cryst. D67, 355-367 (2011)

Coot
"Features and Development of Coot"
P.Emsley, B.Lohkamp, W.Scott, and K.Cowtan
Acta Cryst. D66, 486-501 (2010)

Data collection and refinement statistics for project **mes_6wcf** crystal **crystal1**

model **/home/asia/epe-like-validation/RESULTS/HKL_rerefine/highres/6wcf/structure_mr/build_model_1/hkl_import.pdb** vs
model **/home/asia/epe-like-validation/RESULTS/HKL_rerefine/highres/6wcf/structure_mr/build_model_1/hkl_refine_55.pdb**

Data collection		
	**6WCF**	**Re-refinement**
Resolution (Å)	50.00 - 1.06 (1.09 - 1.06)	60.27 - 0.73
Wavelength (Å)	0.97918	
Space group	P21	P21
a, b, c (Å)	37.17, 33.18, 60.62	37.17, 33.18, 60.62
α, β, γ (°)	90, 96.11, 90	90, 96.11, 90
Completeness (%)	97.9 (94.8)	83.8 (0.0)
Reflections used	64283	
<I> / <Sigma I>	18.7 (1.5)	
Redundancy	5.9 (3.9)	
Rmerge	0.094	
Rpim		
CC1/2 last shell	0.56	
Wilson B factor (Å²)	8.9	
**Refinement**		
Rwork / Rfree	0.125 / 0.154	0.135 / 0.161
Resolution (Å)	33.13 - 1.06	33.14 - 1.06
Reflections all	64228	64261
Reflections for Rfree	3250, 5.1%	3252, 5.1%
Bond lengths rmsd (Å)	0.012	0.018
Bond angles rmsd (°)	1.41	1.98
Mean B value (Å²)	16	16
Number of protein atoms	1413	1413
Mean B value for protein atoms (Å²)	14	14
Number of water atoms (expected)	197 (323)	197 (323)
Mean B value for water atoms (Å²)	31	30
Number of ligand/ion atoms	24	24
Mean B value for ligand/ion atoms (Å²)	23	25
Clashscore	4.12	4.12
Clashscore percentile (100)	-1	-1
Rotamer outliers (<1%)	0.64	0.64
Ramachandran outliers (<0.2%)	0.00	0.00
Ramachandran favored (>98%)	100.00	100.00
Residues with bad bonds (<0%)	0.00	0.30
Residues with bad angles (<0.1%)	0.00	0.30
MolProbity score	1.20	1.20

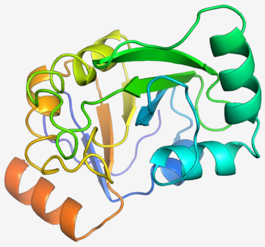

Map cc barchart:

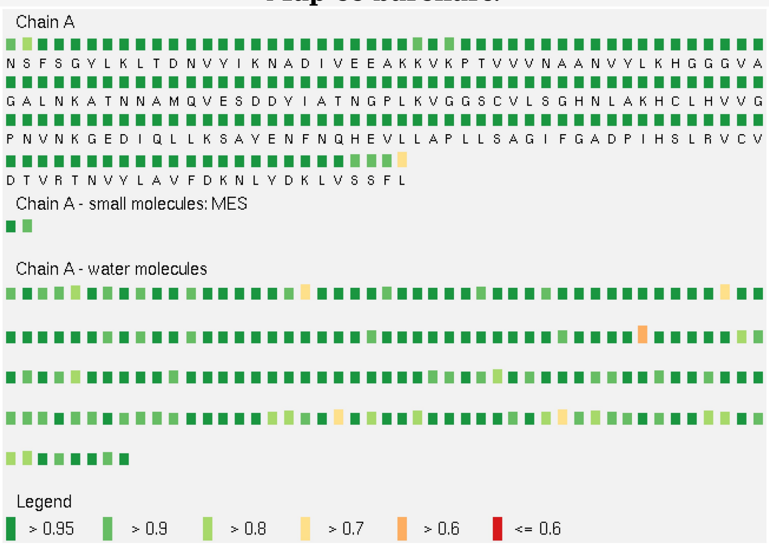

Small molecules with map:

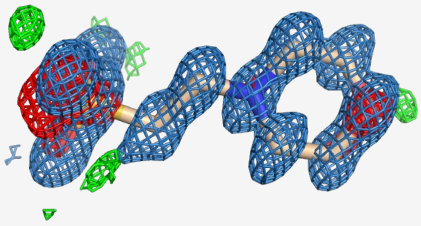

Small molecule 1: 2-(N-MORPHOLINO)-ETHANESULFONIC ACID (MES) A 201 map cc 0.99

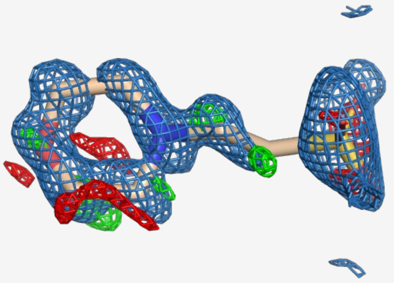

Small molecule 2: 2-(N-MORPHOLINO)-ETHANESULFONIC ACID (MES) A 202 map cc 0.93

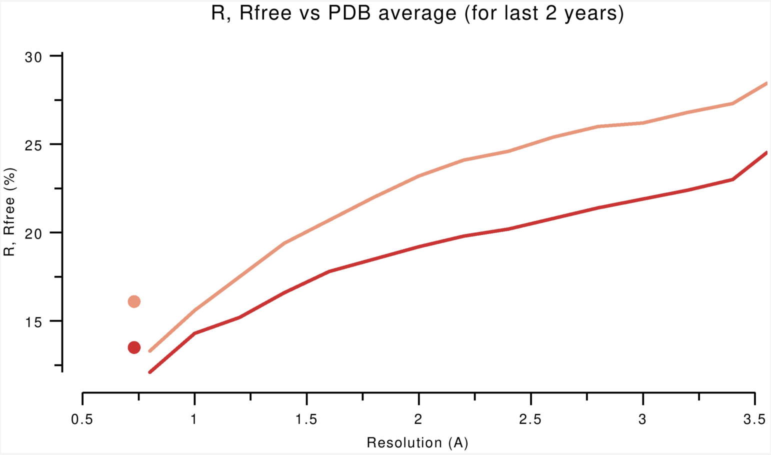

Credits:

HKL-3000
"Processing of X-ray Diffraction Data Collected in Oscillation Mode"
Z.Otwinowski, W.Minor
Methods in Enzymology, Volume 276: Macromolecular Crystallography, part A, p307-326 (1997)
"HKL-3000: the integration of data reduction and structure solution - from diffraction images to an initial model in minutes"
W.Minor, M.Cymborowski, Z.Otwinowski, M.Chruszcz
Acta Cryst. D62: 859-866 (2006)

CCP4 suite
"Overview of the CCP4 suite and current developments"
M.D.Winn et al.
Acta. Cryst. D67, 235-242 (2011)

Refmac5
"REFMAC5 for the refinement of macromolecular crystal structures"
G.N.Murshudov, P.Skubak, A.A.Lebedev, N.S.Pannu, R.A.Steiner, R.A.Nicholls, M.D.Winn, F.Long and A.A.Vagin
Acta Cryst. D67, 355-367 (2011)

Coot
"Features and Development of Coot"
P.Emsley, B.Lohkamp, W.Scott, and K.Cowtan
Acta Cryst. D66, 486-501 (2010)

Data collection and refinement statistics for project **mes_6wcf** crystal **crystal1**

model **/home/asia/epe-like-validation/RESULTS/HKL_rerefine/highres/6wcf/structure_mr/build_model_1/hkl_import.pdb** vs
model **/home/asia/epe-like-validation/RESULTS/HKL_rerefine/highres/6wcf/structure_mr/build_model_1/hkl_refine_203.pdb**

Data collection		
	**6WCF**	**Re-refinement**
Resolution (Å)	50.00 - 1.06 (1.09 - 1.06)	60.27 - 0.73
Wavelength (Å)	0.97918	
Space group	P21	P21
a, b, c (Å)	37.17, 33.18, 60.62	37.17, 33.18, 60.62
α, β, γ (°)	90, 96.11, 90	90, 96.11, 90
Completeness (%)	97.9 (94.8)	83.8 (0.0)
Reflections used	64283	
<I> / <Sigma I>	18.7 (1.5)	
Redundancy	5.9 (3.9)	
Rmerge	0.094	
Rpim		
CC1/2 last shell	0.56	
Wilson B factor (Å²)	8.9	
**Refinement**		
Rwork / Rfree	0.125 / 0.154	0.134 / 0.160
Resolution (Å)	33.13 - 1.06	33.14 - 1.06
Reflections all	64228	64261
Reflections for Rfree	3250, 5.1%	3252, 5.1%
Bond lengths rmsd (Å)	0.012	0.019
Bond angles rmsd (°)	1.41	2.06
Mean B value (Å²)	16	17
Number of protein atoms	1413	1413
Mean B value for protein atoms (Å²)	14	15
Number of water atoms (expected)	197 (323)	197 (323)
Mean B value for water atoms (Å²)	31	30
Number of ligand/ion atoms	24	24
Mean B value for ligand/ion atoms (Å²)	23	25
Clashscore	4.12	4.46
Clashscore percentile (100)	-1	-1
Rotamer outliers (<1%)	0.64	0.64
Ramachandran outliers (<0.2%)	0.00	0.00
Ramachandran favored (>98%)	100.00	100.00
Residues with bad bonds (<0%)	0.00	0.90
Residues with bad angles (<0.1%)	0.00	1.81
MolProbity score	1.20	1.22

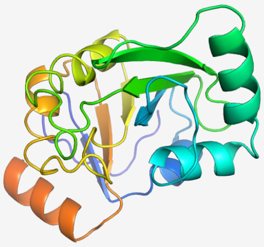

Map cc barchart:

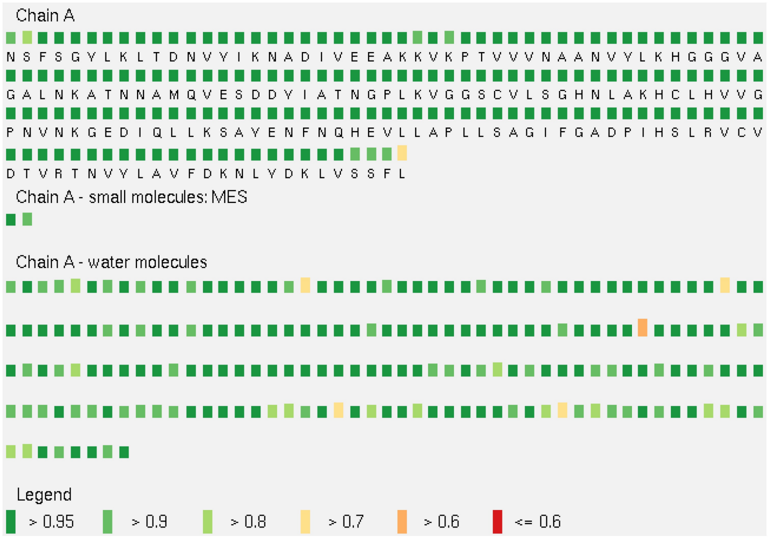

Small molecules with map:

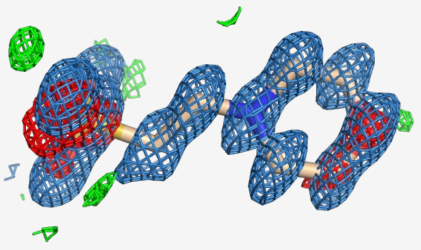

Small molecule 1: 2-(N-MORPHOLINO)-ETHANESULFONIC ACID (MES) A 201 map cc 0.99

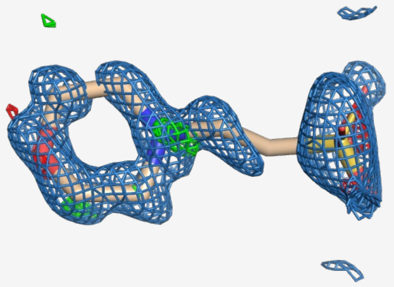

Small molecule 2: 2-(N-MORPHOLINO)-ETHANESULFONIC ACID (MES) A 202 map cc 0.93

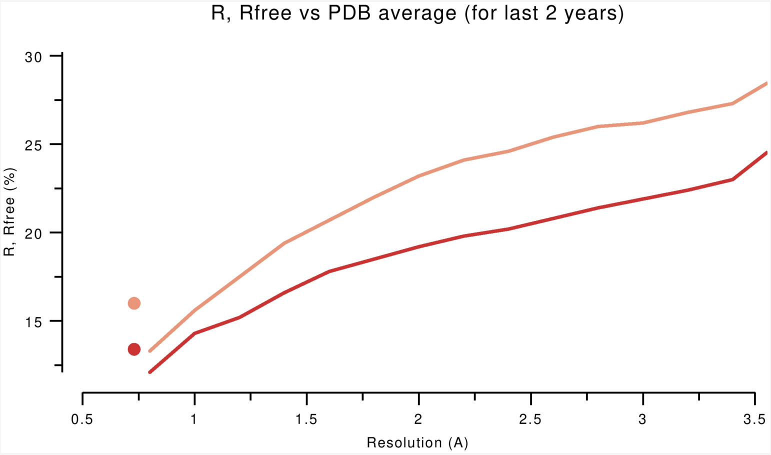

Credits:

HKL-3000
"Processing of X-ray Diffraction Data Collected in Oscillation Mode"
Z.Otwinowski, W.Minor
Methods in Enzymology, Volume 276: Macromolecular Crystallography, part A, p307-326 (1997)
"HKL-3000: the integration of data reduction and structure solution - from diffraction images to an initial model in minutes"
W.Minor, M.Cymborowski, Z.Otwinowski, M.Chruszcz
Acta Cryst. D62: 859-866 (2006)

CCP4 suite
"Overview of the CCP4 suite and current developments"
M.D.Winn et al.
Acta. Cryst. D67, 235-242 (2011)

Refmac5
"REFMAC5 for the refinement of macromolecular crystal structures"
G.N.Murshudov, P.Skubak, A.A.Lebedev, N.S.Pannu, R.A.Steiner, R.A.Nicholls, M.D.Winn, F.Long and A.A.Vagin
Acta Cryst. D67, 355-367 (2011)

Coot
"Features and Development of Coot"
P.Emsley, B.Lohkamp, W.Scott, and K.Cowtan
Acta Cryst. D66, 486-501 (2010)

Data collection and refinement statistics for project **hepes_3dke** crystal **crystal1**

model **/home/asia/epe-like-validation/RESULTS/HKL_rerefine/highres/3dke/structure_mr/build_model_1/hkl_import.pdb_tls** vs
model **/home/asia/epe-like-validation/RESULTS/HKL_rerefine/highres/3dke/structure_mr/build_model_1/hkl_refine_7.pdb**

**Data collection**		
	**3DKE**	**Re-refinement**
Resolution (Å)	52.20 - 1.25 (1.27 - 1.25)	52.16 - 1.25
Wavelength (Å)	0.9796,0.9797,0.9742,0.9807	
Space group	P3221	P3221
a, b, c (Å)	60.23, 60.23, 96.68	60.23, 60.23, 96.68
α, β, γ (°)	90, 90, 120	90, 90, 120
Completeness (%)	99.9 (100.0)	100.0 (100.0)
Reflections used	56299	
<I> / <Sigma I>	23.2 (13.9)	
Redundancy	9.6 (7.1)	
Rmerge	0.115	
Rpim		
CC1/2 last shell		
Wilson B factor (Å²)	10.9	
**Refinement**		
Rwork / Rfree	0.155 / 0.173	0.169 / 0.173
Resolution (Å)	52.13 - 1.25	52.16 - 1.25
Reflections all	53423	56108
Reflections for Rfree	2851, 5.1%	2789, 5.0%
Bond lengths rmsd (Å)	0.007	0.016
Bond angles rmsd (°)	1.14	1.89
Mean B value (Å²)	15	14
Number of protein atoms	1334	1334
Mean B value for protein atoms (Å²)	12	12
Number of water atoms (expected)	256 (242)	256 (242)
Mean B value for water atoms (Å²)	27	24
Number of ligand/ion atoms	57	57
Mean B value for ligand/ion atoms (Å²)	22	25
Clashscore	4.24	4.24
Clashscore percentile (100)	-1	-1
Rotamer outliers (<1%)	0.00	0.00
Ramachandran outliers (<0.2%)	0.00	0.00
Ramachandran favored (>98%)	97.53	97.53
Residues with bad bonds (<0%)	0.00	0.30
Residues with bad angles (<0.1%)	0.30	0.61
MolProbity score	1.30	1.30

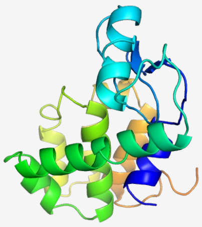

Map cc barchart:

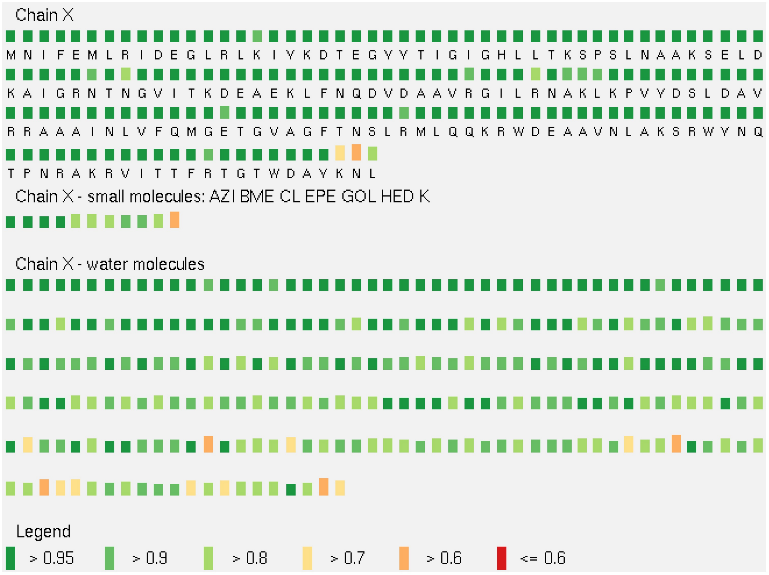

Small molecules with map:

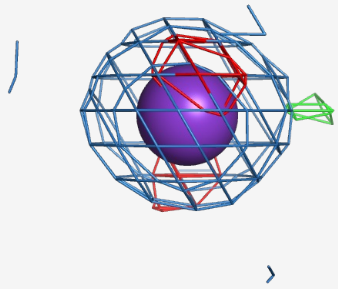

Small molecule 1: POTASSIUM ION (K) X 601 map cc 0.99

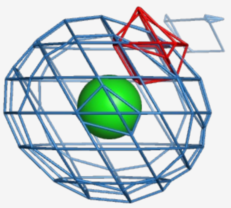

Small molecule 2: CHLORIDE ION (CL) X 701 map cc 0.99

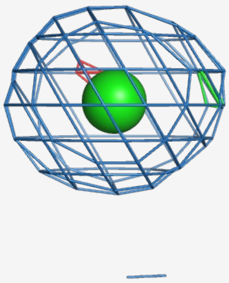

Small molecule 3: CHLORIDE ION (CL) X 702 map cc 0.99

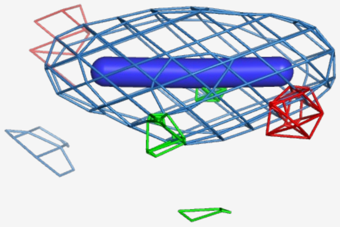

Small molecule 4: AZIDE ION (AZI) X 401 map cc 0.98

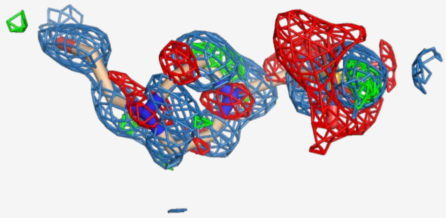

Small molecule 5: 4-(2-HYDROXYETHYL)-1-PIPERAZINEETHANESULFONICACID (EPE) X 901 map cc 0.86

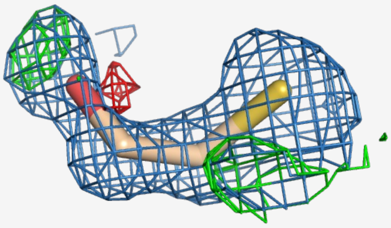

Small molecule 6: BETA-MERCAPTOETHANOL (BME) X 805 map cc 0.86

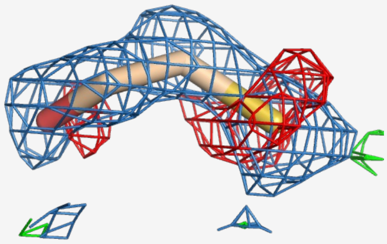

Small molecule 7: BETA-MERCAPTOETHANOL (BME) X 806 map cc 0.89

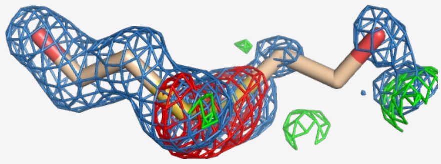

Small molecule 8: 2-HYDROXYETHYL DISULFIDE (HED) X 801 map cc 0.93

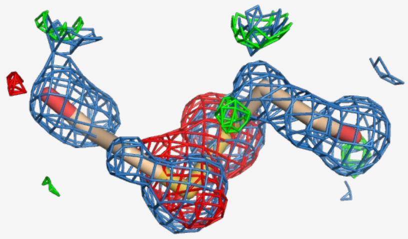

Small molecule 9: 2-HYDROXYETHYL DISULFIDE (HED) X 802 map cc 0.91

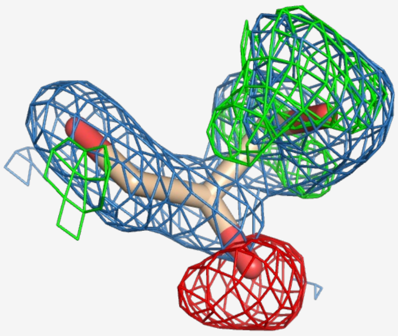

Small molecule 10: GLYCEROL (GOL) X 501 map cc 0.82

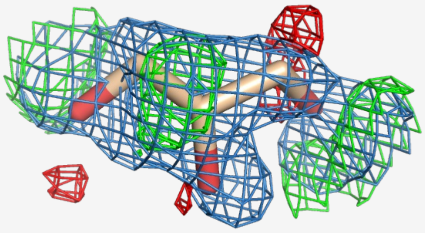

Small molecule 11: GLYCEROL (GOL) X 502 map cc 0.70

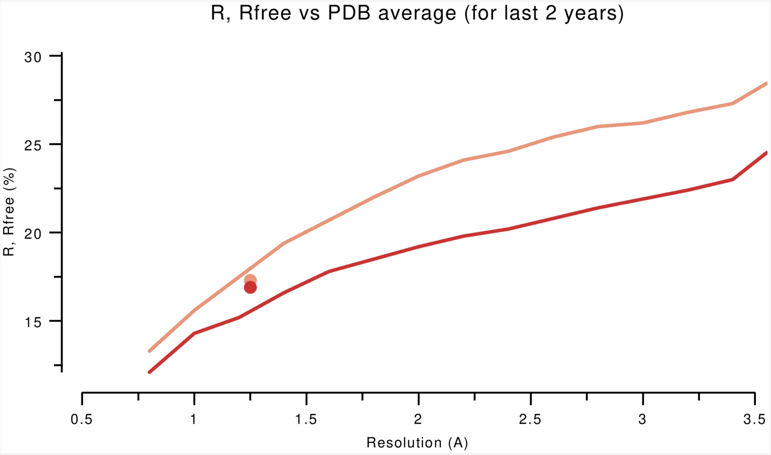

Credits:

HKL-3000

"Processing of X-ray Diffraction Data Collected in Oscillation Mode"
Z.Otwinowski, W.Minor
Methods in Enzymology, Volume 276: Macromolecular Crystallography, part A, p307-326 (1997)
"HKL-3000: the integration of data reduction and structure solution - from diffraction images to an initial model in minutes"
W.Minor, M.Cymborowski, Z.Otwinowski, M.Chruszcz
Acta Cryst. D62: 859-866 (2006)

CCP4 suite
"Overview of the CCP4 suite and current developments"
M.D.Winn et al.
Acta. Cryst. D67, 235-242 (2011)

Refmac5
"REFMAC5 for the refinement of macromolecular crystal structures"
G.N.Murshudov, P.Skubak, A.A.Lebedev, N.S.Pannu, R.A.Steiner, R.A.Nicholls, M.D.Winn, F.Long and A.A.Vagin
Acta Cryst. D67, 355-367 (2011)

Coot
"Features and Development of Coot"
P.Emsley, B.Lohkamp, W.Scott, and K.Cowtan
Acta Cryst. D66, 486-501 (2010)

Data collection and refinement statistics for project **hepes_3dke** crystal **crystal1**

model **/home/asia/epe-like-validation/RESULTS/HKL_rerefine/highres/3dke/structure_mr/build_model_1/hkl_import.pdb_tls** vs
model **/home/asia/epe-like-validation/RESULTS/HKL_rerefine/highres/3dke/structure_mr/build_model_1/hkl_refine_13.pdb**

	3DKE	Re-refinement
**Data collection**		
Resolution (Å)	52.20 - 1.25 (1.27 - 1.25)	52.16 - 1.25
Wavelength (Å)	0.9796,0.9797,0.9742,0.9807	
Space group	P3221	P3221
a, b, c (Å)	60.23, 60.23, 96.68	60.23, 60.23, 96.68
α, β, γ (°)	90, 90, 120	90, 90, 120
Completeness (%)	99.9 (100.0)	100.0 (100.0)
Reflections used	56299	
<I> / <Sigma I>	23.2 (13.9)	
Redundancy	9.6 (7.1)	
Rmerge	0.115	
Rpim		
CC1/2 last shell		
Wilson B factor (Å²)	10.9	
**Refinement**		
Rwork / Rfree	0.155 / 0.173	0.160 / 0.175
Resolution (Å)	52.13 - 1.25	52.16 - 1.25
Reflections all	53423	56108
Reflections for Rfree	2851, 5.1%	2789, 5.0%
Bond lengths rmsd (Å)	0.007	0.020
Bond angles rmsd (°)	1.14	2.40
Mean B value (Å²)	15	14
Number of protein atoms	1334	1334
Mean B value for protein atoms (Å²)	12	11
Number of water atoms (expected)	256 (242)	256 (242)
Mean B value for water atoms (Å²)	27	24
Number of ligand/ion atoms	57	57
Mean B value for ligand/ion atoms (Å²)	22	24
Clashscore	4.24	5.30
Clashscore percentile (100)	-1	-1
Rotamer outliers (<1%)	0.00	0.00
Ramachandran outliers (<0.2%)	0.00	0.00
Ramachandran favored (>98%)	97.53	98.15
Residues with bad bonds (<0%)	0.00	1.52
Residues with bad angles (<0.1%)	0.30	3.66
MolProbity score	1.30	1.28

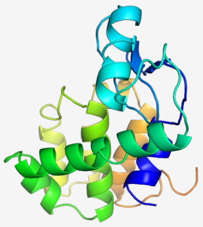

Map cc barchart:

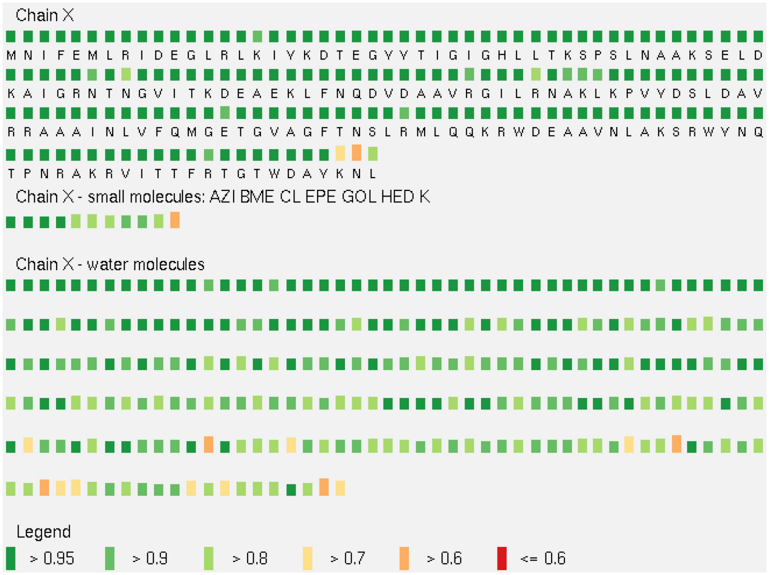

Small molecules with map:

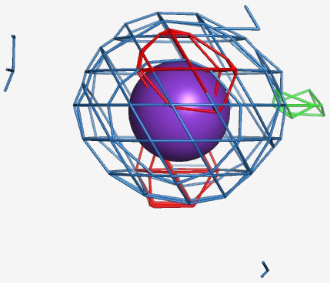

Small molecule 1: POTASSIUM ION (K) X 601 map cc 0.99

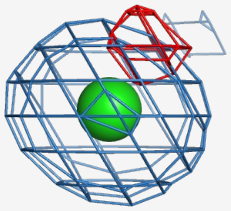

Small molecule 2: CHLORIDE ION (CL) X 701 map cc 0.99

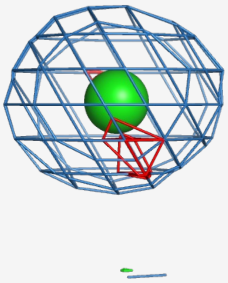

Small molecule 3: CHLORIDE ION (CL) X 702 map cc 0.99

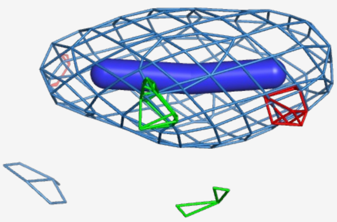

Small molecule 4: AZIDE ION (AZI) X 401 map cc 0.98

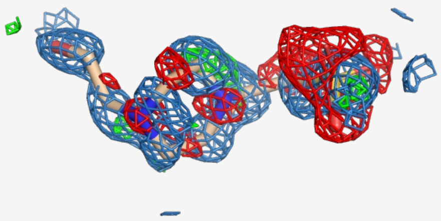

Small molecule 5: 4-(2-HYDROXYETHYL)-1-PIPERAZINEETHANESULFONICACID (EPE) X 901 map cc 0.86

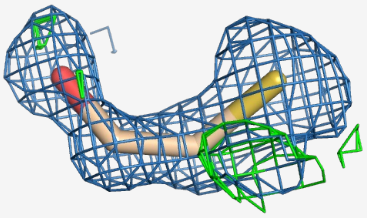

Small molecule 6: BETA-MERCAPTOETHANOL (BME) X 805 map cc 0.86

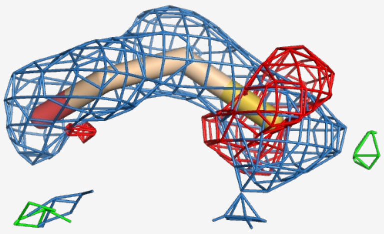

Small molecule 7: BETA-MERCAPTOETHANOL (BME) X 806 map cc 0.89

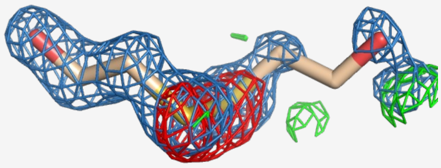

Small molecule 8: 2-HYDROXYETHYL DISULFIDE (HED) X 801 map cc 0.93

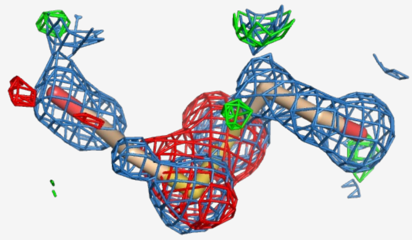

Small molecule 9: 2-HYDROXYETHYL DISULFIDE (HED) X 802 map cc 0.91

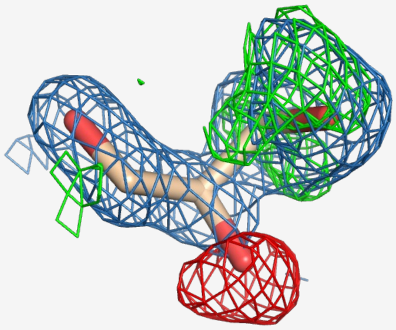

Small molecule 10: GLYCEROL (GOL) X 501 map cc 0.82

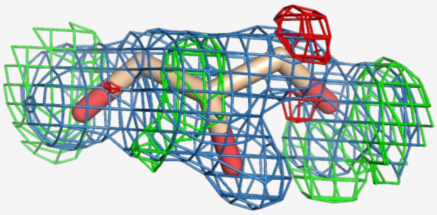

Small molecule 11: GLYCEROL (GOL) X 502 map cc 0.70

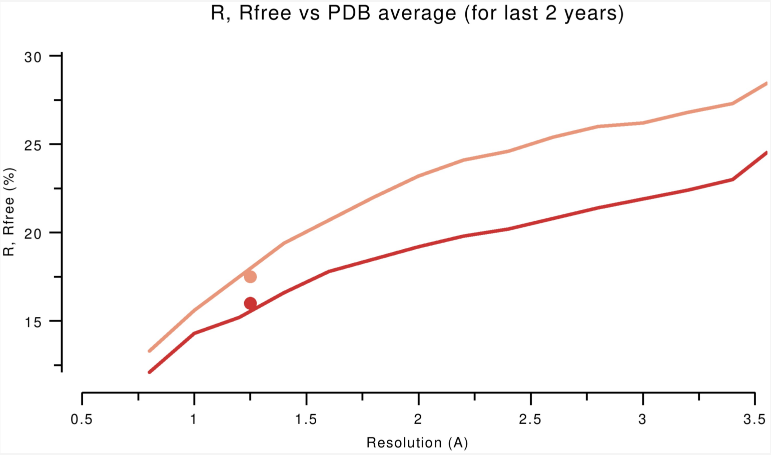

Credits:

HKL-3000

"Processing of X-ray Diffraction Data Collected in Oscillation Mode"
Z.Otwinowski, W.Minor
Methods in Enzymology, Volume 276: Macromolecular Crystallography, part A, p307-326 (1997)
"HKL-3000: the integration of data reduction and structure solution - from diffraction images to an initial model in minutes"
W.Minor, M.Cymborowski, Z.Otwinowski, M.Chruszcz
Acta Cryst. D62: 859-866 (2006)

CCP4 suite
"Overview of the CCP4 suite and current developments"
M.D.Winn et al.
Acta. Cryst. D67, 235-242 (2011)

Refmac5
"REFMAC5 for the refinement of macromolecular crystal structures"
G.N.Murshudov, P.Skubak, A.A.Lebedev, N.S.Pannu, R.A.Steiner, R.A.Nicholls, M.D.Winn, F.Long and A.A.Vagin
Acta Cryst. D67, 355-367 (2011)

Coot
"Features and Development of Coot"
P.Emsley, B.Lohkamp, W.Scott, and K.Cowtan
Acta Cryst. D66, 486-501 (2010)

Data collection and refinement statistics for project **hepes_3e10** crystal **crystal1**

model **/home/asia/epe-like-validation/RESULTS/HKL_rerefine/highres/3e10/structure_mr/build_model_1/hkl_import.pdb** vs
model **/home/asia/epe-like-validation/RESULTS/HKL_rerefine/highres/3e10/structure_mr/build_model_1/hkl_refine_3.pdb**

**Data collection**		
	**3E10**	**Re-refinement**
Resolution (Å)	29.27 - 1.40 (1.44 - 1.40)	29.27 - 1.40
Wavelength (Å)	0.91837,0.97908,0.97849	
Space group	P21212	P21212
a, b, c (Å)	82.08, 83.53, 51.89	82.08, 83.53, 51.89
α, β, γ (°)	90, 90, 90	90, 90, 90
Completeness (%)	100.0	100.0 (100.0)
Reflections used	70997	
<I> / <Sigma I>	(1.5)	
Redundancy	3.7 (3.6)	
Rmerge	0.096	
Rpim		
CC1/2 last shell		
Wilson B factor (Å^2)	10.6	
**Refinement**		
Rwork / Rfree	0.130 / 0.168	0.153 / 0.157
Resolution (Å)	29.27 - 1.40	29.27 - 1.40
Reflections all	70948	70948
Reflections for Rfree	3583, 5.1%	3485, 4.9%
Bond lengths rmsd (Å)	0.015	0.019
Bond angles rmsd (°)	1.57	2.07
Mean B value (Å^2)	13	14
Number of protein atoms	2860	2860
Mean B value for protein atoms (Å^2)	11	11
Number of water atoms (expected)	519 (454)	519 (454)
Mean B value for water atoms (Å^2)	27	28
Number of ligand/ion atoms	134	134
Mean B value for ligand/ion atoms (Å^2)	16	17
Clashscore	3.75	3.91
Clashscore percentile (100)	-1	-1
Rotamer outliers (<1%)	0.96	0.96
Ramachandran outliers (<0.2%)	0.00	0.00
Ramachandran favored (>98%)	97.26	97.26
Residues with bad bonds (<0%)	0.30	0.30
Residues with bad angles (<0.1%)	0.00	0.00
MolProbity score	1.30	1.32

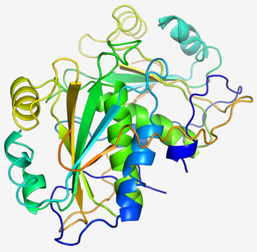

Map cc barchart:

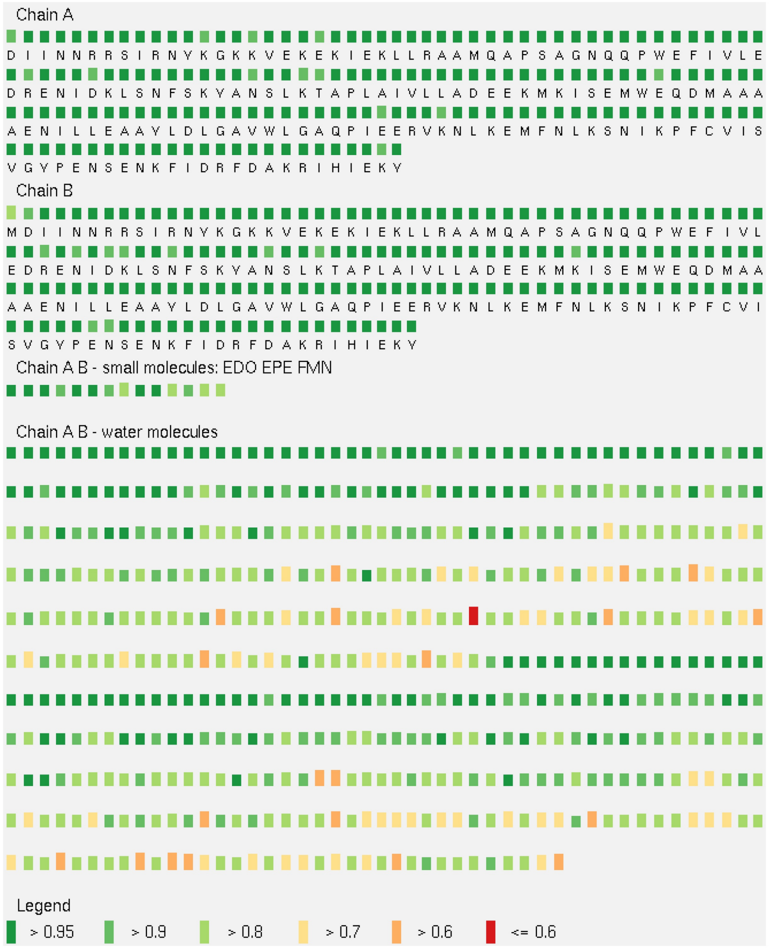

Small molecules with map:

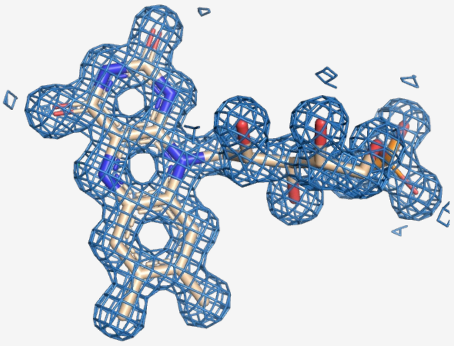

Small molecule 1: FLAVINMONONUCLEOTIDE (FMN) A 501 map cc 0.99

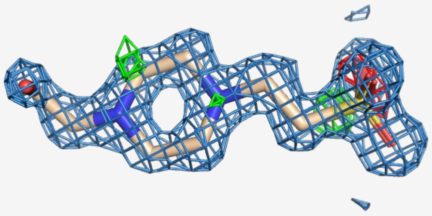

Small molecule 2: 4-(2-HYDROXYETHYL)-1-PIPERAZINEETHANESULFONICACID (EPE) A 502 map cc 0.98

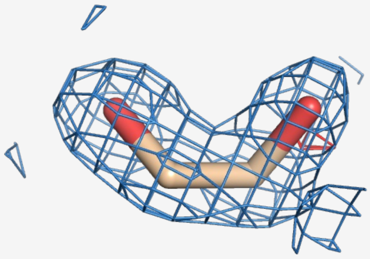

Small molecule 3: 1,2-ETHANEDIOL (EDO) A 503 map cc 0.96

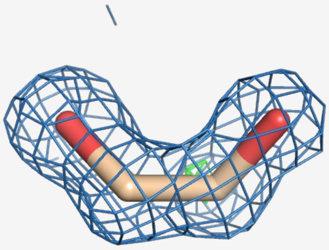

Small molecule 4: 1,2-ETHANEDIOL (EDO) A 504 map cc 0.95

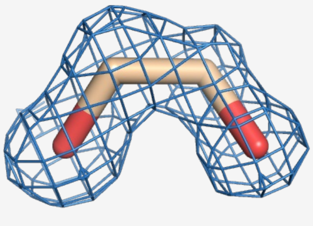

Small molecule 5: 1,2-ETHANEDIOL (EDO) A 505 map cc 0.98

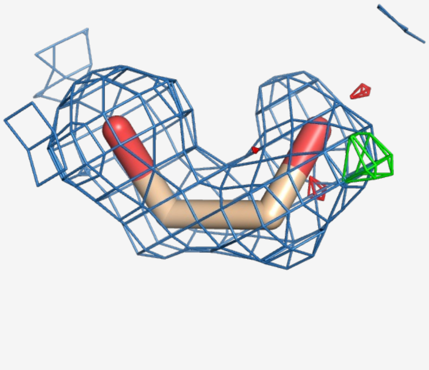

Small molecule 6: 1,2-ETHANEDIOL (EDO) A 506 map cc 0.96

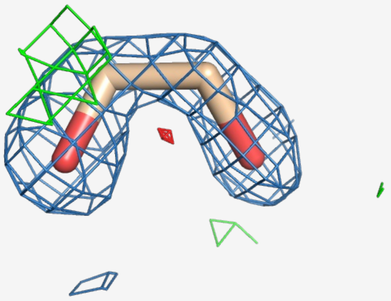

Small molecule 7: 1,2-ETHANEDIOL (EDO) A 507 map cc 0.94

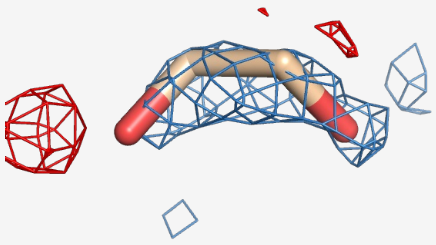

Small molecule 8: 1,2-ETHANEDIOL (EDO) A 508 map cc 0.83

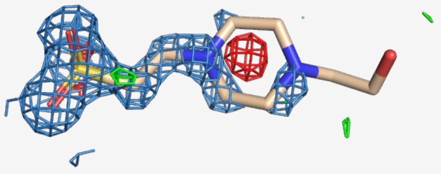

Small molecule 9: 4-(2-HYDROXYETHYL)-1-PIPERAZINEETHANESULFONICACID (EPE) B 502 map cc 0.97

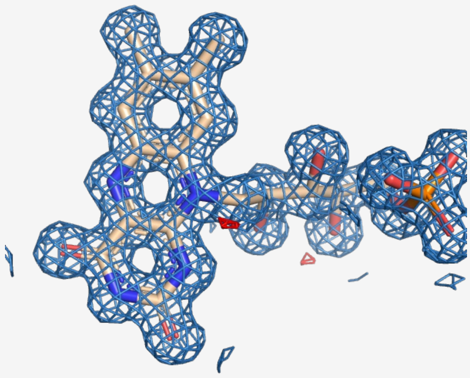

Small molecule 10: FLAVINMONONUCLEOTIDE (FMN) B 501 map cc 0.99

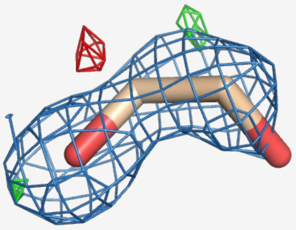

Small molecule 11: 1,2-ETHANEDIOL (EDO) B 503 map cc 0.89

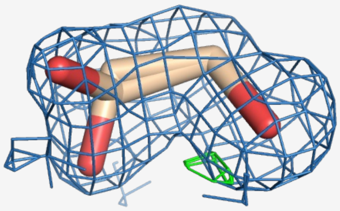

Small molecule 12: 1,2-ETHANEDIOL (EDO) B 504 map cc 0.94

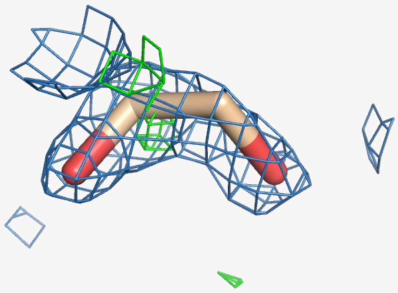

Small molecule 13: 1,2-ETHANEDIOL (EDO) B 505 map cc 0.87

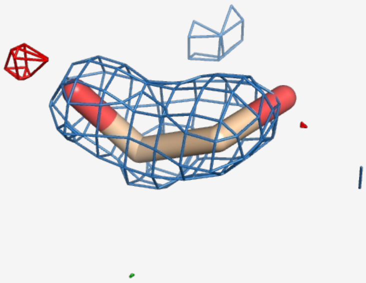

Small molecule 14: 1,2-ETHANEDIOL (EDO) B 506 map cc 0.87

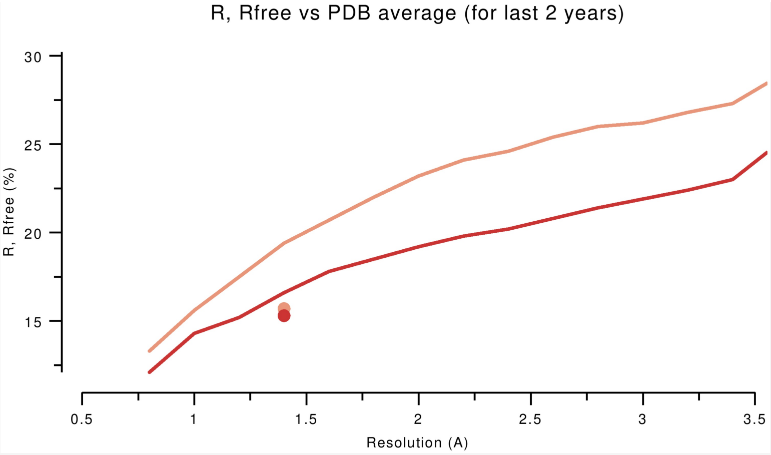

Credits:

HKL-3000
"Processing of X-ray Diffraction Data Collected in Oscillation Mode"
Z.Otwinowski, W.Minor
Methods in Enzymology, Volume 276: Macromolecular Crystallography, part A, p307-326 (1997)
"HKL-3000: the integration of data reduction and structure solution - from diffraction images to an initial model in minutes"
W.Minor, M.Cymborowski, Z.Otwinowski, M.Chruszcz
Acta Cryst. D62: 859-866 (2006)

CCP4 suite
"Overview of the CCP4 suite and current developments"
M.D.Winn et al.
Acta. Cryst. D67, 235-242 (2011)

Refmac5
"REFMAC5 for the refinement of macromolecular crystal structures"
G.N.Murshudov, P.Skubak, A.A.Lebedev, N.S.Pannu, R.A.Steiner, R.A.Nicholls, M.D.Winn, F.Long and A.A.Vagin
Acta Cryst. D67, 355-367 (2011)

Coot
"Features and Development of Coot"
P.Emsley, B.Lohkamp, W.Scott, and K.Cowtan
Acta Cryst. D66, 486-501 (2010)

Data collection and refinement statistics for project **hepes_3e10** crystal **crystal1**

model **/home/asia/epe-like-validation/RESULTS/HKL_rerefine/highres/3e10/structure_mr/build_model_1/hkl_import.pdb** vs
model **/home/asia/epe-like-validation/RESULTS/HKL_rerefine/highres/3e10/structure_mr/build_model_1/hkl_refine_4.pdb**

**Data collection**		
	**3E10**	**Re-refinement**
Resolution (Å)	29.27 - 1.40 (1.44 - 1.40)	29.27 - 1.40
Wavelength (Å)	0.91837,0.97908,0.97849	
Space group	P21212	P21212
a, b, c (Å)	82.08, 83.53, 51.89	82.08, 83.53, 51.89
α, β, γ (°)	90, 90, 90	90, 90, 90
Completeness (%)	100.0	100.0 (100.0)
Reflections used	70997	
<I> / <Sigma I>	(1.5)	
Redundancy	3.7 (3.6)	
Rmerge	0.096	
Rpim		
CC1/2 last shell		
Wilson B factor (Å^2)	10.6	
**Refinement**		
Rwork / Rfree	0.130 / 0.168	0.149 / 0.166
Resolution (Å)	29.27 - 1.40	29.27 - 1.40
Reflections all	70948	70948
Reflections for Rfree	3583, 5.1%	3485, 4.9%
Bond lengths rmsd (Å)	0.015	0.015
Bond angles rmsd (°)	1.57	1.96
Mean B value (Å^2)	13	14
Number of protein atoms	2860	2860
Mean B value for protein atoms (Å^2)	11	11
Number of water atoms (expected)	519 (454)	519 (454)
Mean B value for water atoms (Å^2)	27	28
Number of ligand/ion atoms	134	134
Mean B value for ligand/ion atoms (Å^2)	16	17
Clashscore	3.75	3.42
Clashscore percentile (100)	-1	-1
Rotamer outliers (<1%)	0.96	0.64
Ramachandran outliers (<0.2%)	0.00	0.00
Ramachandran favored (>98%)	97.26	97.26
Residues with bad bonds (<0%)	0.30	0.75
Residues with bad angles (<0.1%)	0.00	1.05
MolProbity score	1.30	1.27

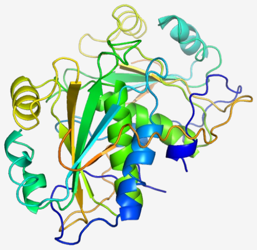

Map cc barchart:

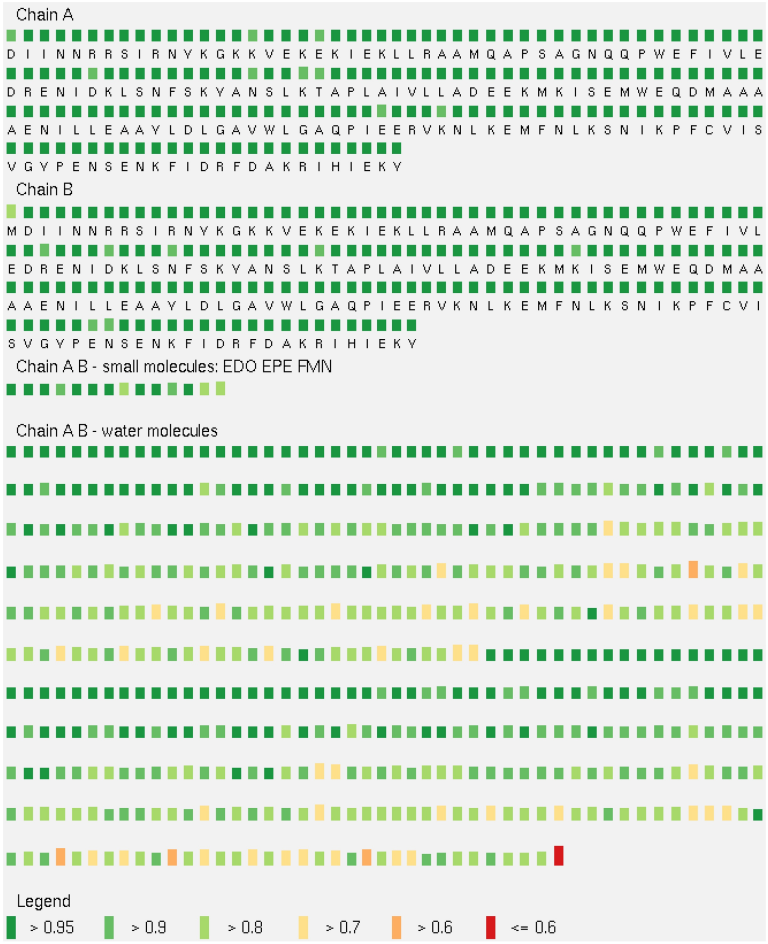

Small molecules with map:

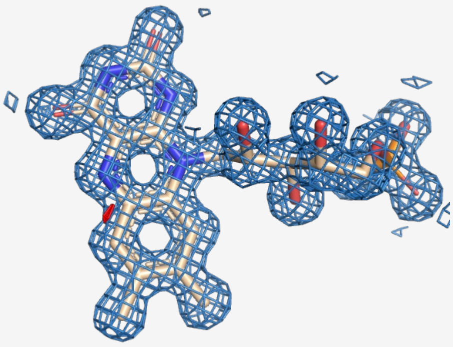

Small molecule 1: FLAVINMONONUCLEOTIDE (FMN) A 501 map cc 0.99

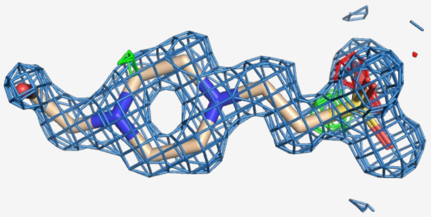

Small molecule 2: 4-(2-HYDROXYETHYL)-1-PIPERAZINEETHANESULFONICACID (EPE) A 502 map cc 0.98

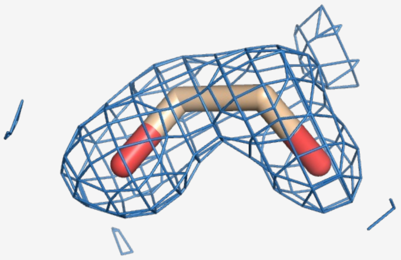

Small molecule 3: 1,2-ETHANEDIOL (EDO) A 503 map cc 0.97

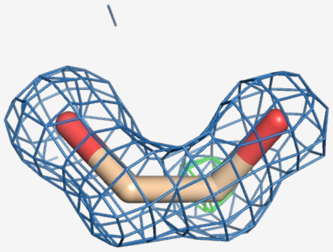

Small molecule 4: 1,2-ETHANEDIOL (EDO) A 504 map cc 0.95

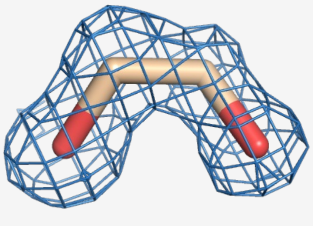

Small molecule 5: 1,2-ETHANEDIOL (EDO) A 505 map cc 0.98

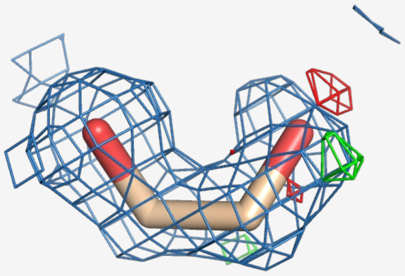

Small molecule 6: 1,2-ETHANEDIOL (EDO) A 506 map cc 0.96

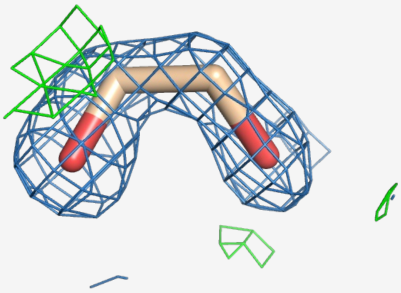

Small molecule 7: 1,2-ETHANEDIOL (EDO) A 507 map cc 0.97

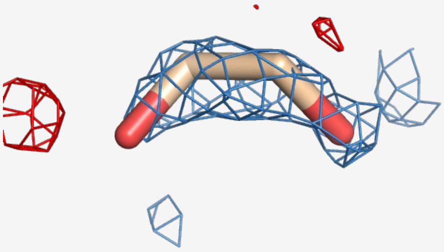

Small molecule 8: 1,2-ETHANEDIOL (EDO) A 508 map cc 0.89

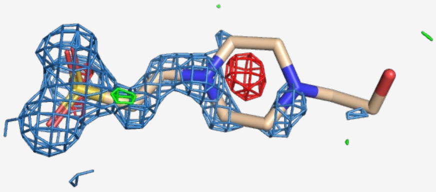

Small molecule 9: 4-(2-HYDROXYETHYL)-1-PIPERAZINEETHANESULFONICACID (EPE) B 502 map cc 0.97

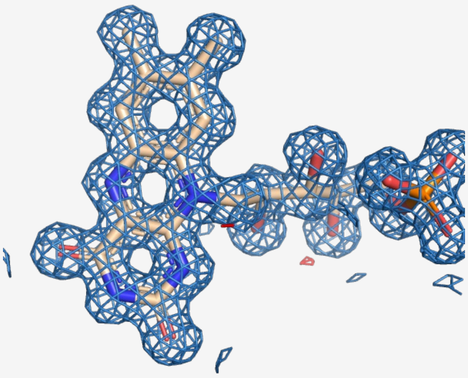

Small molecule 10: FLAVINMONONUCLEOTIDE (FMN) B 501 map cc 0.99

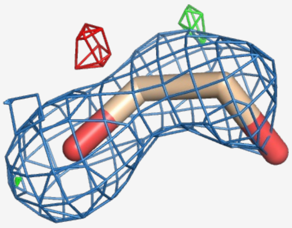

Small molecule 11: 1,2-ETHANEDIOL (EDO) B 503 map cc 0.91

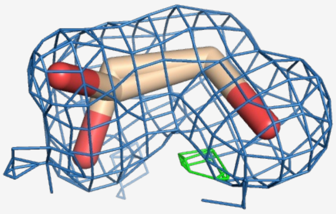

Small molecule 12: 1,2-ETHANEDIOL (EDO) B 504 map cc 0.96

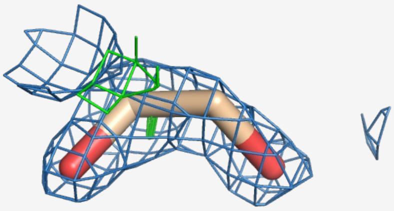

Small molecule 13: 1,2-ETHANEDIOL (EDO) B 505 map cc 0.89

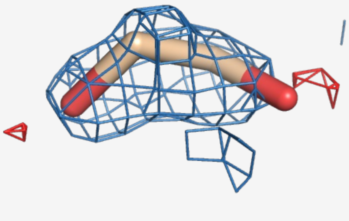

Small molecule 14: 1,2-ETHANEDIOL (EDO) B 506 map cc 0.81

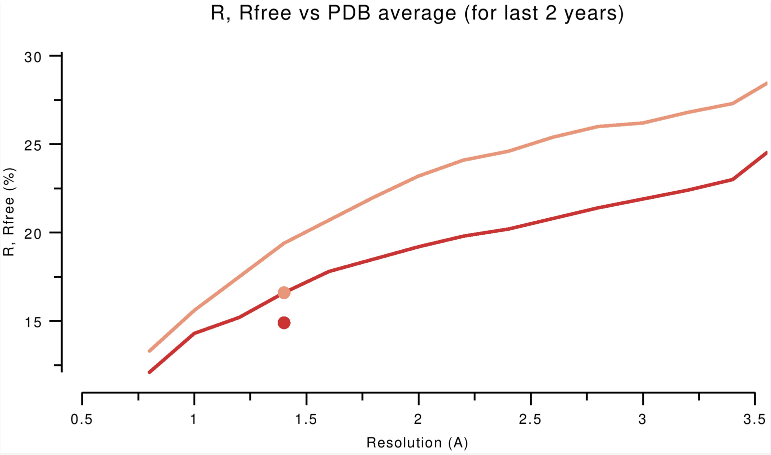

Credits:

HKL-3000
"Processing of X-ray Diffraction Data Collected in Oscillation Mode"
Z.Otwinowski, W.Minor
Methods in Enzymology, Volume 276: Macromolecular Crystallography, part A, p307-326 (1997)
"HKL-3000: the integration of data reduction and structure solution - from diffraction images to an initial model in minutes"
W.Minor, M.Cymborowski, Z.Otwinowski, M.Chruszcz
Acta Cryst. D62: 859-866 (2006)

CCP4 suite
"Overview of the CCP4 suite and current developments"
M.D.Winn et al.
Acta. Cryst. D67, 235-242 (2011)

Refmac5
"REFMAC5 for the refinement of macromolecular crystal structures"
G.N.Murshudov, P.Skubak, A.A.Lebedev, N.S.Pannu, R.A.Steiner, R.A.Nicholls, M.D.Winn, F.Long and A.A.Vagin
Acta Cryst. D67, 355-367 (2011)

Coot
"Features and Development of Coot"
P.Emsley, B.Lohkamp, W.Scott, and K.Cowtan
Acta Cryst. D66, 486-501 (2010)

Data collection and refinement statistics for project **hepes_6g38** crystal **crystal1**

model **/home/asia/epe-like-validation/RESULTS/HKL_rerefine/highres/6g38/structure_mr/build_model_1/hkl_import.pdb** vs
model **/home/asia/epe-like-validation/RESULTS/HKL_rerefine/highres/6g38/structure_mr/build_model_1/hkl_refine_6.pdb**

**Data collection**		
	**6G38**	**Re-refinement**
Resolution (Å)	24.05 - 1.43 (1.45 - 1.43)	56.13 - 1.47
Wavelength (Å)	0.9794	
Space group	P212121	P212121
a, b, c (Å)	78.56, 78.76, 80.02	78.56, 78.76, 80.02
α, β, γ (°)	90, 90, 90	90, 90, 90
Completeness (%)	99.9 (98.3)	100.0 (100.0)
Reflections used	92144	
<I> / <Sigma I>	8.6 (1.2)	
Redundancy	5.9 (4.2)	
Rmerge	0.102	
Rpim		
CC1/2 last shell	0.38	
Wilson B factor (Å^2)	19.5	
**Refinement**		
Rwork / Rfree	0.143 / 0.165	0.169 / 0.180
Resolution (Å)	24.05 - 1.47	24.06 - 1.47
Reflections all	80839	85009
Reflections for Rfree	4169, 4.9%	4169, 4.9%
Bond lengths rmsd (Å)	0.012	0.016
Bond angles rmsd (°)	1.54	1.94
Mean B value (Å^2)	23	22
Number of protein atoms	2595	2595
Mean B value for protein atoms (Å^2)	22	21
Number of water atoms (expected)	269 (461)	269 (461)
Mean B value for water atoms (Å^2)	30	30
Number of ligand/ion atoms	43	43
Mean B value for ligand/ion atoms (Å^2)	45	45
Clashscore	0.38	0.38
Clashscore percentile (100)	-1	-1
Rotamer outliers (<1%)	0.35	0.35
Ramachandran outliers (<0.2%)	0.00	0.00
Ramachandran favored (>98%)	98.77	98.77
Residues with bad bonds (<0%)	0.46	0.46
Residues with bad angles (<0.1%)	0.46	0.46
MolProbity score	0.64	0.64

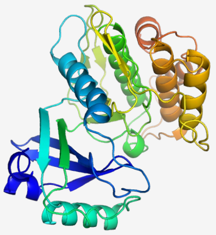

Map cc barchart:

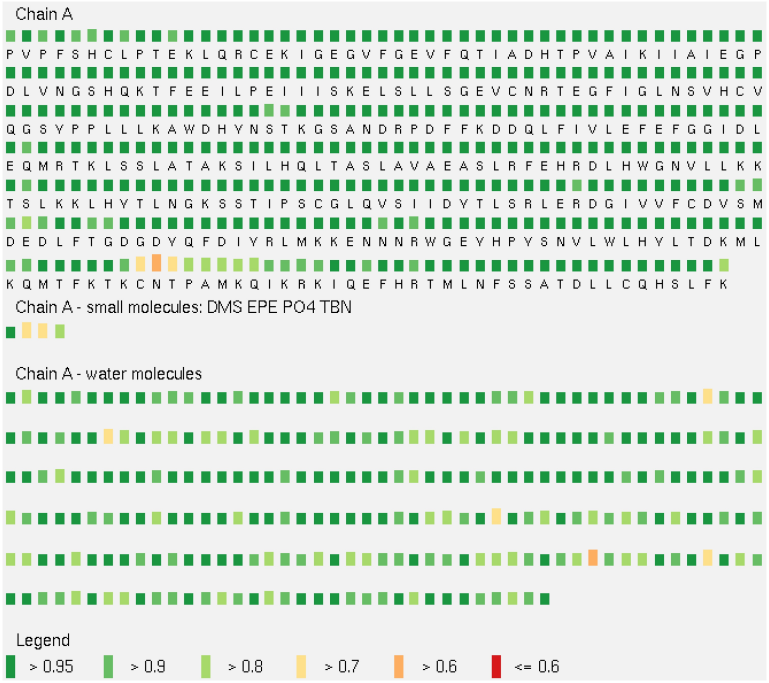

Small molecules with map:

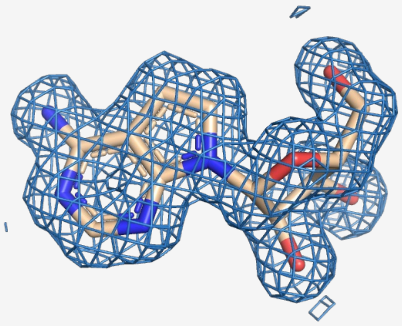

Small molecule 1: '2-(4-AMINO-PYRROLO[2,3-D]PYRIMIDIN-7-YL)-5-HYDROXYMETHYL-TETRAHYDRO-FURAN-3,4-DIOL (TBN) A 800 map cc 0.99

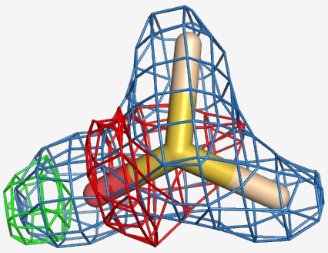

Small molecule 2: DIMETHYL SULFOXIDE (DMS) A 801 map cc 0.73

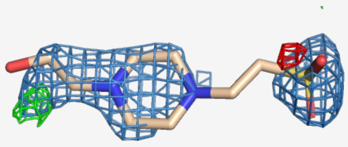

Small molecule 3: 4-(2-HYDROXYETHYL)-1-PIPERAZINEETHANESULFONICACID (EPE) A 802 map cc 0.78

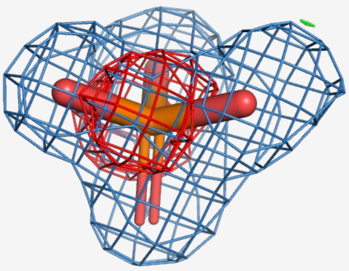

Small molecule 4: PHOSPHATE ION (PO4) A 803 map cc 0.85

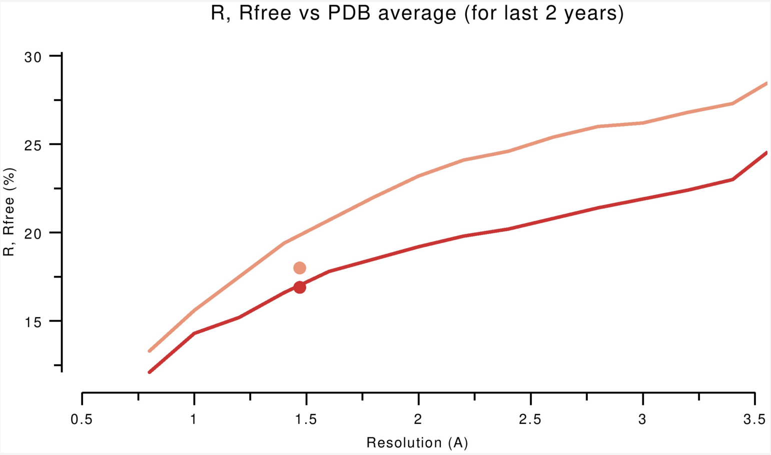

Credits:

HKL-3000
"Processing of X-ray Diffraction Data Collected in Oscillation Mode"
Z.Otwinowski, W.Minor
Methods in Enzymology, Volume 276: Macromolecular Crystallography, part A, p307-326 (1997)
"HKL-3000: the integration of data reduction and structure solution - from diffraction images to an initial model in minutes"
W.Minor, M.Cymborowski, Z.Otwinowski, M.Chruszcz
Acta Cryst. D62: 859-866 (2006)

CCP4 suite
"Overview of the CCP4 suite and current developments"
M.D.Winn et al.
Acta. Cryst. D67, 235-242 (2011)

Refmac5
"REFMAC5 for the refinement of macromolecular crystal structures"
G.N.Murshudov, P.Skubak, A.A.Lebedev, N.S.Pannu, R.A.Steiner, R.A.Nicholls, M.D.Winn, F.Long and A.A.Vagin
Acta Cryst. D67, 355-367 (2011)

Coot
"Features and Development of Coot"
P.Emsley, B.Lohkamp, W.Scott, and K.Cowtan
Acta Cryst. D66, 486-501 (2010)

Data collection and refinement statistics for project **hepes_6g38** crystal **crystal1**

model **/home/asia/epe-like-validation/RESULTS/HKL_rerefine/highres/6g38/structure_mr/build_model_1/hkl_import.pdb** vs
model **/home/asia/epe-like-validation/RESULTS/HKL_rerefine/highres/6g38/structure_mr/build_model_1/hkl_refine_7.pdb**

Data collection		
	**6G38**	**Re-refinement**
Resolution (Å)	24.05 - 1.43 (1.45 - 1.43)	56.13 - 1.47
Wavelength (Å)	0.9794	
Space group	P212121	P212121
a, b, c (Å)	78.56, 78.76, 80.02	78.56, 78.76, 80.02
α, β, γ (°)	90, 90, 90	90, 90, 90
Completeness (%)	99.9 (98.3)	100.0 (100.0)
Reflections used	92144	
<I> / <Sigma I>	8.6 (1.2)	
Redundancy	5.9 (4.2)	
Rmerge	0.102	
Rpim		
CC1/2 last shell	0.38	
Wilson B factor (Å²)	19.5	
**Refinement**		
Rwork / Rfree	0.143 / 0.165	0.166 / 0.180
Resolution (Å)	24.05 - 1.47	24.06 - 1.47
Reflections all	80839	85009
Reflections for Rfree	4169, 4.9%	4169, 4.9%
Bond lengths rmsd (Å)	0.012	0.015
Bond angles rmsd (°)	1.54	1.96
Mean B value (Å²)	23	22
Number of protein atoms	2595	2595
Mean B value for protein atoms (Å²)	22	21
Number of water atoms (expected)	269 (461)	269 (461)
Mean B value for water atoms (Å²)	30	30
Number of ligand/ion atoms	43	43
Mean B value for ligand/ion atoms (Å²)	45	44
Clashscore	0.38	0.96
Clashscore percentile (100)	-1	-1
Rotamer outliers (<1%)	0.35	0.35
Ramachandran outliers (<0.2%)	0.00	0.00
Ramachandran favored (>98%)	98.77	98.46
Residues with bad bonds (<0%)	0.46	1.07
Residues with bad angles (<0.1%)	0.46	1.99
MolProbity score	0.64	0.79

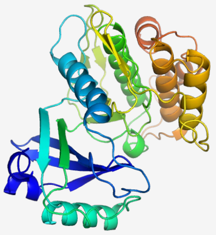

Map cc barchart:

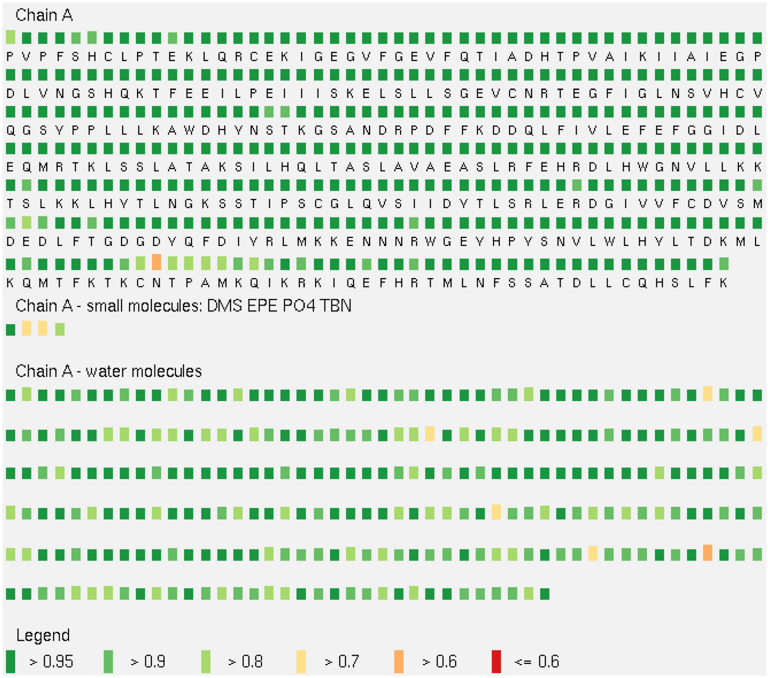

Small molecules with map:

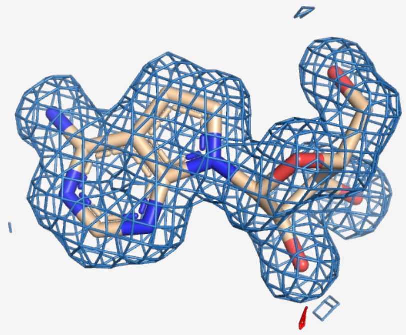

Small molecule 1: '2-(4-AMINO-PYRROLO[2,3-D]PYRIMIDIN-7-YL)-5-HYDROXYMETHYL-TETRAHYDRO-FURAN-3,4-DIOL (TBN) A 800 map cc 0.99

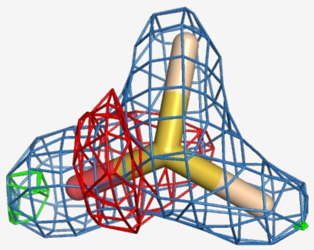

Small molecule 2: DIMETHYL SULFOXIDE (DMS) A 801 map cc 0.80

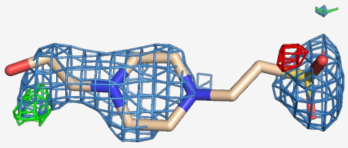

Small molecule 3: 4-(2-HYDROXYETHYL)-1-PIPERAZINEETHANESULFONICACID (EPE) A 802 map cc 0.78

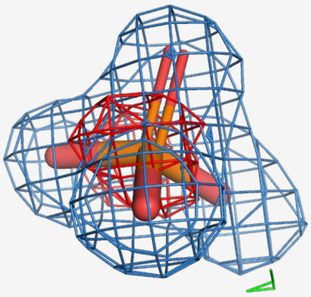

Small molecule 4: PHOSPHATE ION (PO4) A 803 map cc 0.88

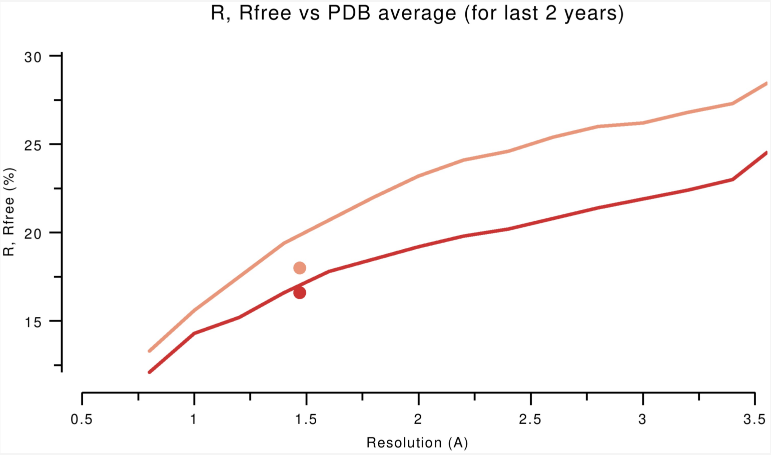

Credits:

HKL-3000
"Processing of X-ray Diffraction Data Collected in Oscillation Mode"
Z.Otwinowski, W.Minor
Methods in Enzymology, Volume 276: Macromolecular Crystallography, part A, p307-326 (1997)
"HKL-3000: the integration of data reduction and structure solution - from diffraction images to an initial model in minutes"
W.Minor, M.Cymborowski, Z.Otwinowski, M.Chruszcz
Acta Cryst. D62: 859-866 (2006)

CCP4 suite
"Overview of the CCP4 suite and current developments"
M.D.Winn et al.
Acta. Cryst. D67, 235-242 (2011)

Refmac5
"REFMAC5 for the refinement of macromolecular crystal structures"
G.N.Murshudov, P.Skubak, A.A.Lebedev, N.S.Pannu, R.A.Steiner, R.A.Nicholls, M.D.Winn, F.Long and A.A.Vagin
Acta Cryst. D67, 355-367 (2011)

Coot
"Features and Development of Coot"
P.Emsley, B.Lohkamp, W.Scott, and K.Cowtan
Acta Cryst. D66, 486-501 (2010)

Data collection and refinement statistics for project **hepes_4e8r** crystal **crystal1**

model **/home/asia/epe-like-validation/RESULTS/HKL_rerefine/lowres/4e8r/structure_mr/build_model_1/hkl_import.pdb** vs
model **/home/asia/epe-like-validation/RESULTS/HKL_rerefine/lowres/4e8r/structure_mr/build_model_1/hkl_refine_7.pdb**

**Data collection**		
	**4E8R**	**Re-refinement**
Resolution (Å)	48.50 - 3.36 (3.45 - 3.36)	48.50 - 3.36
Wavelength (Å)	1.4861	
Space group	P212121	P212121
a, b, c (Å)	90.20, 94.92, 225.71	90.20, 94.92, 225.71
α, β, γ (°)	90, 90, 90	90, 90, 90
Completeness (%)	97.5 (96.8)	100.0 (100.0)
Reflections used	26102	
<I> / <Sigma I>	15.1 (2.0)	
Redundancy	7.0 (6.7)	
Rmerge	0.081	
Rpim		
CC1/2 last shell		
**Refinement**		
Rwork / Rfree	0.200 / 0.258	0.210 / 0.268
Resolution (Å)	48.50 - 3.36	48.51 - 3.36
Reflections all	26102	27485
Reflections for Rfree	1384, 5.0%	1384, 5.0%
Bond lengths rmsd (Å)	0.007	0.042
Bond angles rmsd (°)	2.39	2.48
Mean B value (Å^2)	134	130
Number of water atoms	26	26
Mean B value for water atoms (Å^2)	80	83
Number of RNA atoms	8412	8412
Mean B value for RNA atoms (Å^2)	135	131
Number of ligand/ion atoms	122	122
Mean B value for ligand/ion atoms (Å^2)	113	114
Clashscore	26.75	30.02
Clashscore percentile (100)	-1	-1
Rotamer outliers (<1%)	0.00	0.00
Ramachandran outliers (<0.2%)	0.00	0.00
Ramachandran favored (>98%)	0.00	0.00
Residues with bad bonds (<0%)	2.81	2.55
Residues with bad angles (<0.1%)	115.56	114.29
MolProbity score	3.06	3.11

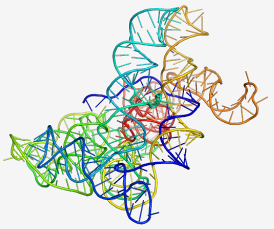

Map cc barchart:

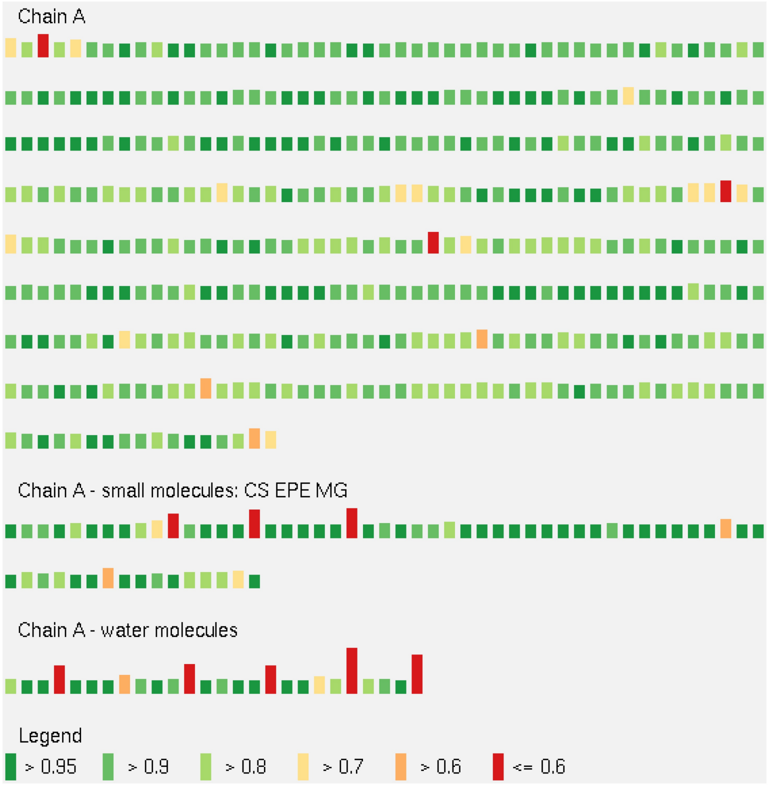

Small molecules with map:

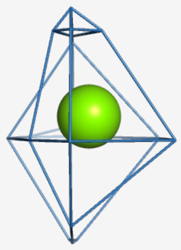

Small molecule 1: MAGNESIUM ION (MG) A 401 map cc 0.98

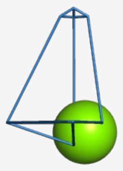

Small molecule 2: MAGNESIUM ION (MG) A 402 map cc 0.95

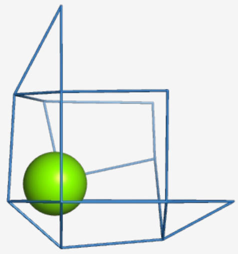

Small molecule 3: MAGNESIUM ION (MG) A 403 map cc 0.94

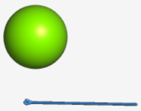

Small molecule 4: MAGNESIUM ION (MG) A 404 map cc 0.98

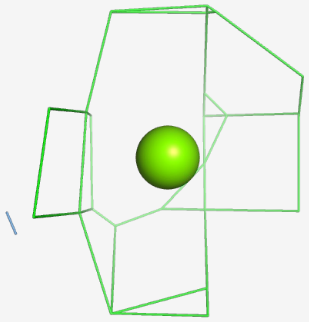

Small molecule 5: MAGNESIUM ION (MG) A 405 map cc 0.89

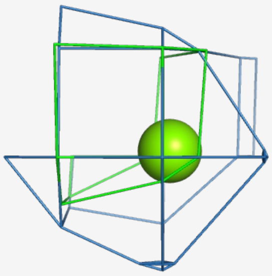

Small molecule 6: MAGNESIUM ION (MG) A 406 map cc 0.97

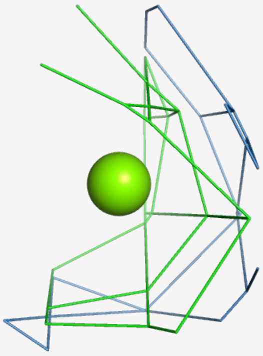

Small molecule 7: MAGNESIUM ION (MG) A 407 map cc 0.95

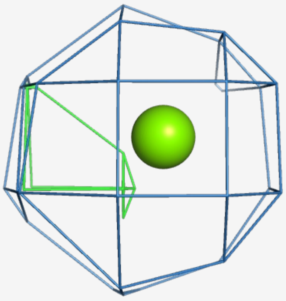

Small molecule 8: MAGNESIUM ION (MG) A 408 map cc 0.97

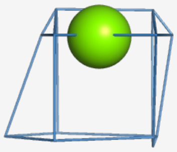

Small molecule 9: MAGNESIUM ION (MG) A 409 map cc 0.88

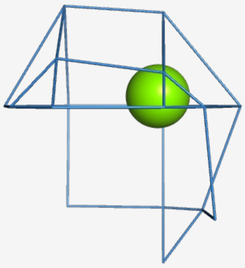

Small molecule 10: MAGNESIUM ION (MG) A 410 map cc 0.73

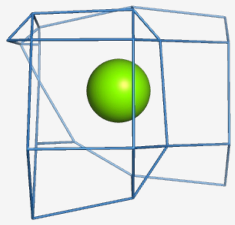

Small molecule 11: MAGNESIUM ION (MG) A 411 map cc 0.44

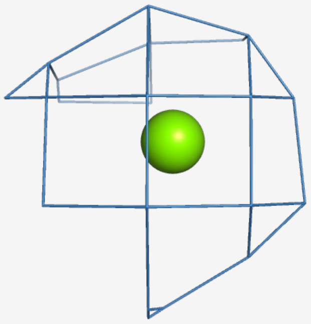

Small molecule 12: MAGNESIUM ION (MG) A 412 map cc 0.94

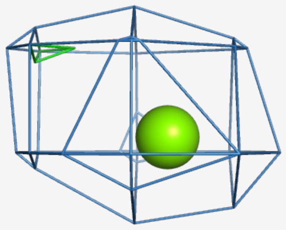

Small molecule 13: MAGNESIUM ION (MG) A 413 map cc 0.99

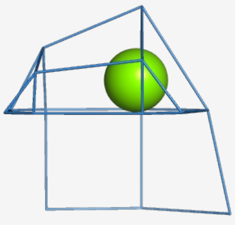

Small molecule 14: MAGNESIUM ION (MG) A 414 map cc 0.98

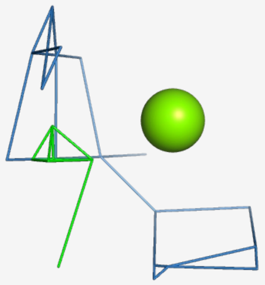

Small molecule 15: MAGNESIUM ION (MG) A 415 map cc 1.00

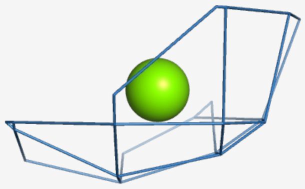

Small molecule 16: MAGNESIUM ION (MG) A 416 map cc 0.26

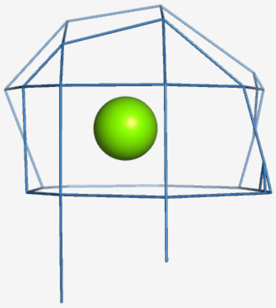

Small molecule 17: MAGNESIUM ION (MG) A 417 map cc 0.96

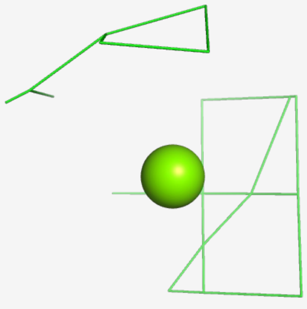

Small molecule 18: MAGNESIUM ION (MG) A 418 map cc 0.99

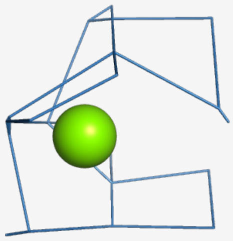

Small molecule 19: MAGNESIUM ION (MG) A 419 map cc 0.98

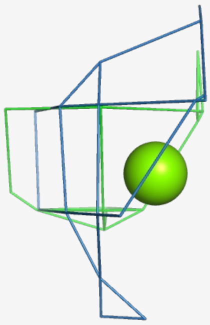

Small molecule 20: MAGNESIUM ION (MG) A 420 map cc 1.00

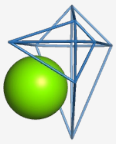

Small molecule 21: MAGNESIUM ION (MG) A 421 map cc 0.99

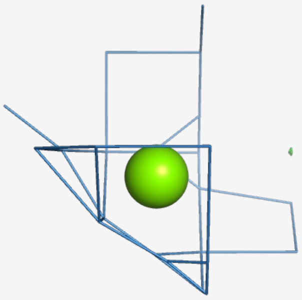

Small molecule 22: MAGNESIUM ION (MG) A 422 map cc 0.14

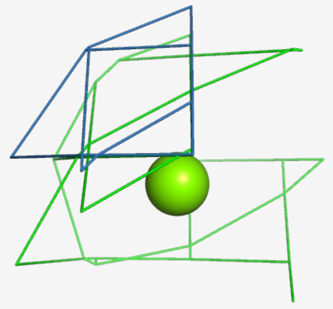

Small molecule 23: MAGNESIUM ION (MG) A 423 map cc 0.98

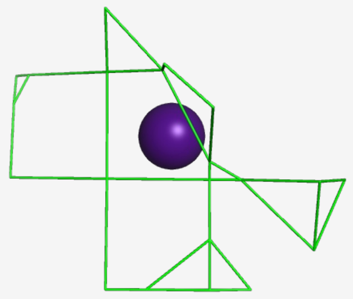

Small molecule 24: CESIUM ION (CS) A 424 map cc 0.93

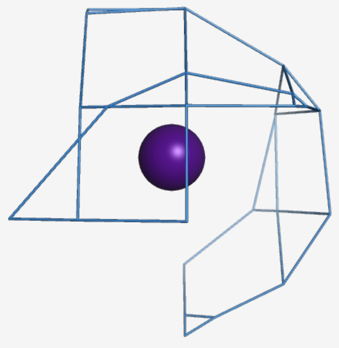

Small molecule 25: CESIUM ION (CS) A 425 map cc 0.99

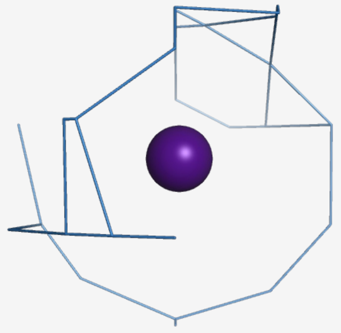

Small molecule 26: CESIUM ION (CS) A 426 map cc 0.95

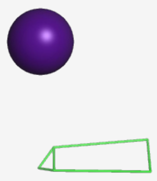

Small molecule 27: CESIUM ION (CS) A 427 map cc 0.95

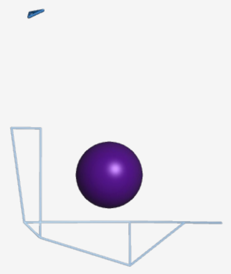

Small molecule 28: CESIUM ION (CS) A 428 map cc 0.81

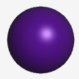

Small molecule 29: CESIUM ION (CS) A 429 map cc 1.00

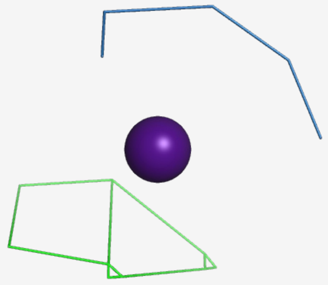

Small molecule 30: CESIUM ION (CS) A 430 map cc 1.00

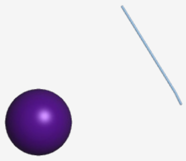

Small molecule 31: CESIUM ION (CS) A 431 map cc 1.00

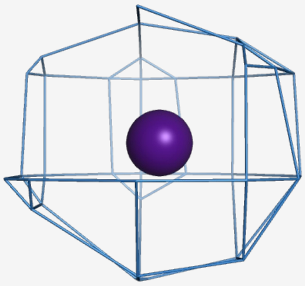

Small molecule 32: CESIUM ION (CS) A 432 map cc 1.00

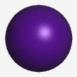

Small molecule 33: CESIUM ION (CS) A 433 map cc 0.99

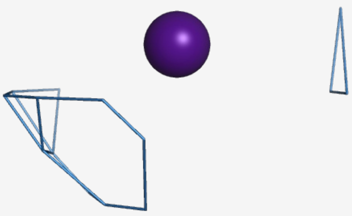

Small molecule 34: CESIUM ION (CS) A 434 map cc 1.00

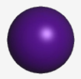

Small molecule 35: CESIUM ION (CS) A 435 map cc 0.99

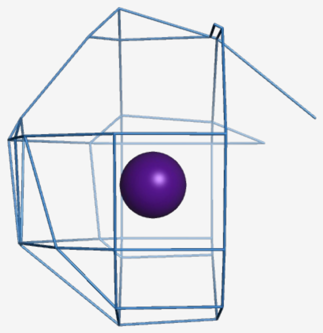

Small molecule 36: CESIUM ION (CS) A 436 map cc 0.98

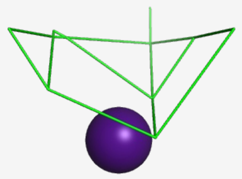

Small molecule 37: CESIUM ION (CS) A 437 map cc 1.00

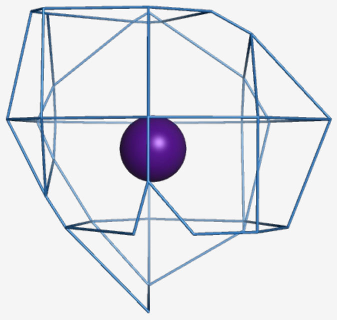

Small molecule 38: CESIUM ION (CS) A 438 map cc 0.92

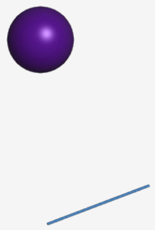

Small molecule 39: CESIUM ION (CS) A 439 map cc 1.00

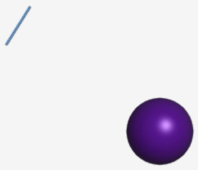

Small molecule 40: CESIUM ION (CS) A 440 map cc 0.97

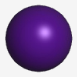

Small molecule 41: CESIUM ION (CS) A 441 map cc 0.98

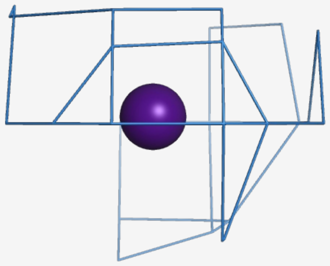

Small molecule 42: CESIUM ION (CS) A 442 map cc 1.00

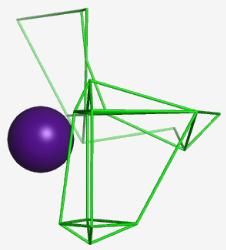

Small molecule 43: CESIUM ION (CS) A 443 map cc 1.00

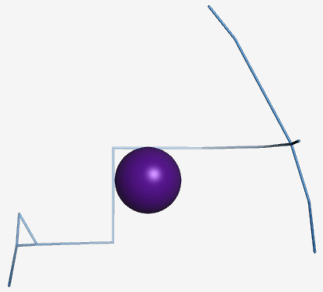

Small molecule 44: CESIUM ION (CS) A 444 map cc 0.99

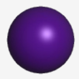

Small molecule 45: CESIUM ION (CS) A 445 map cc 0.67

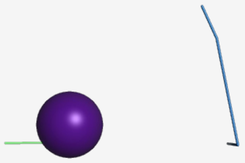

Small molecule 46: CESIUM ION (CS) A 446 map cc 1.00

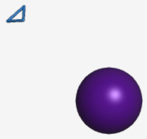

Small molecule 47: CESIUM ION (CS) A 447 map cc 0.97

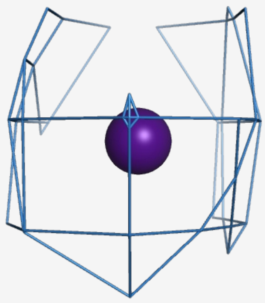

Small molecule 48: CESIUM ION (CS) A 448 map cc 0.99

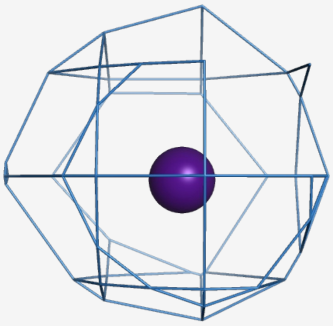

Small molecule 49: CESIUM ION (CS) A 449 map cc 0.81

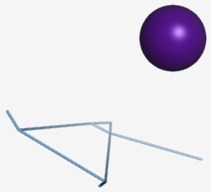

Small molecule 50: CESIUM ION (CS) A 450 map cc 0.90

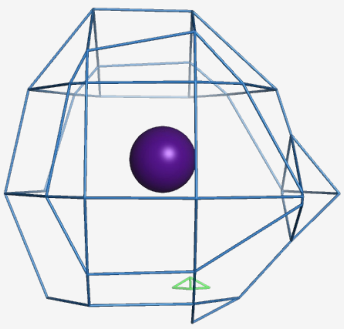

Small molecule 51: CESIUM ION (CS) A 451 map cc 0.87

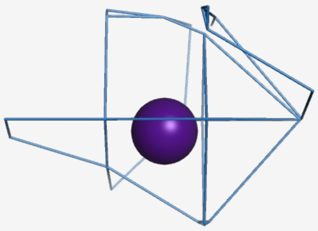

Small molecule 52: CESIUM ION (CS) A 452 map cc 1.00

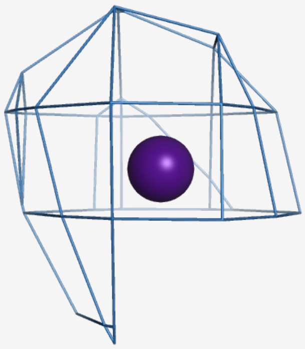

Small molecule 53: CESIUM ION (CS) A 453 map cc 1.00

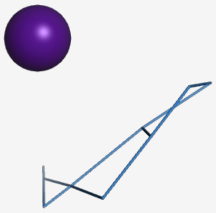

Small molecule 54: CESIUM ION (CS) A 454 map cc 0.66

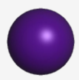

Small molecule 55: CESIUM ION (CS) A 455 map cc 1.00

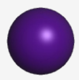

Small molecule 56: CESIUM ION (CS) A 456 map cc 1.00

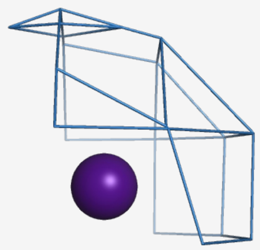

Small molecule 57: CESIUM ION (CS) A 457 map cc 0.92

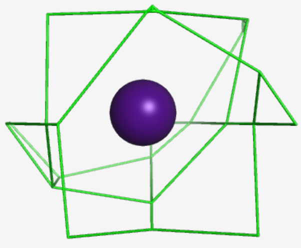

Small molecule 58: CESIUM ION (CS) A 458 map cc 0.98

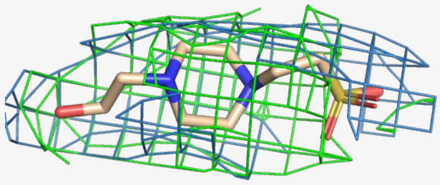

Small molecule 59: 4-(2-HYDROXYETHYL)-1-PIPERAZINEETHANESULFONICACID (EPE) A 459 map cc 0.86

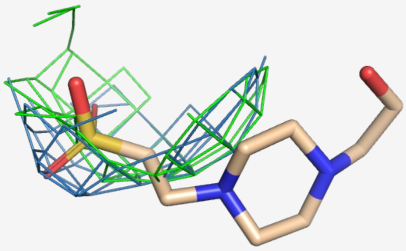

Small molecule 60: 4-(2-HYDROXYETHYL)-1-PIPERAZINEETHANESULFONICACID (EPE) A 460 map cc 0.85

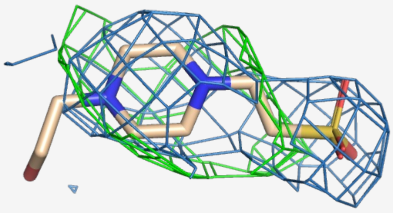

Small molecule 61: 4-(2-HYDROXYETHYL)-1-PIPERAZINEETHANESULFONICACID (EPE) A 461 map cc 0.83

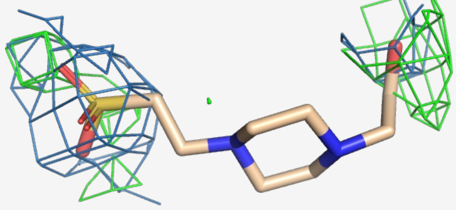

Small molecule 62: 4-(2-HYDROXYETHYL)-1-PIPERAZINEETHANESULFONICACID (EPE) A 462 map cc 0.79

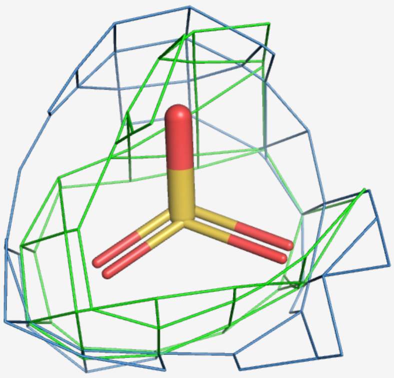

Small molecule 63: 4-(2-HYDROXYETHYL)-1-PIPERAZINEETHANESULFONICACID (EPE) A 463 map cc 0.99

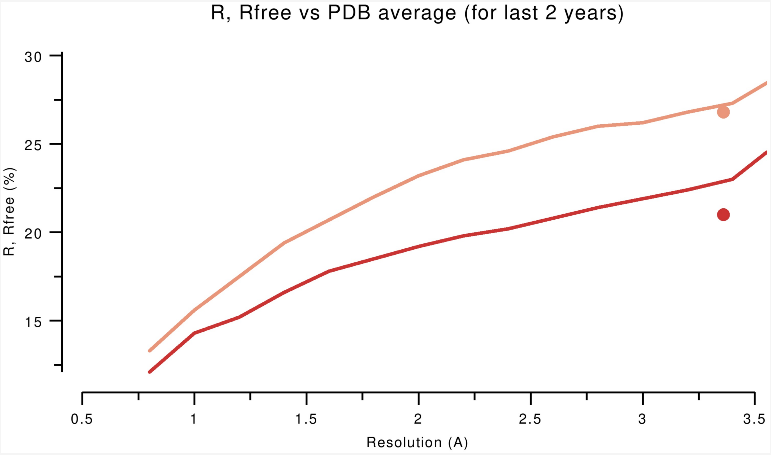

Credits:

HKL-3000
"Processing of X-ray Diffraction Data Collected in Oscillation Mode"
Z.Otwinowski, W.Minor
Methods in Enzymology, Volume 276: Macromolecular Crystallography, part A, p307-326 (1997)
"HKL-3000: the integration of data reduction and structure solution - from diffraction images to an initial model in minutes"
W.Minor, M.Cymborowski, Z.Otwinowski, M.Chruszcz
Acta Cryst. D62: 859-866 (2006)

CCP4 suite
"Overview of the CCP4 suite and current developments"
M.D.Winn et al.
Acta. Cryst. D67, 235-242 (2011)

Refmac5
"REFMAC5 for the refinement of macromolecular crystal structures"
G.N.Murshudov, P.Skubak, A.A.Lebedev, N.S.Pannu, R.A.Steiner, R.A.Nicholls, M.D.Winn, F.Long and A.A.Vagin
Acta Cryst. D67, 355-367 (2011)

Coot
"Features and Development of Coot"
P.Emsley, B.Lohkamp, W.Scott, and K.Cowtan
Acta Cryst. D66, 486-501 (2010)

Data collection and refinement statistics for project **hepes_4e8r** crystal **crystal1**

model **/home/asia/epe-like-validation/RESULTS/HKL_rerefine/lowres/4e8r/structure_mr/build_model_1/hkl_import.pdb** vs
model **/home/asia/epe-like-validation/RESULTS/HKL_rerefine/lowres/4e8r/structure_mr/build_model_1/hkl_refine_8.pdb**

**Data collection**		
	**4E8R**	**Re-refinement**
Resolution (Å)	48.50 - 3.36 (3.45 - 3.36)	48.50 - 3.36
Wavelength (Å)	1.4861	
Space group	P212121	P212121
a, b, c (Å)	90.20, 94.92, 225.71	90.20, 94.92, 225.71
α, β, γ (°)	90, 90, 90	90, 90, 90
Completeness (%)	97.5 (96.8)	100.0 (100.0)
Reflections used	26102	
<I> / <Sigma I>	15.1 (2.0)	
Redundancy	7.0 (6.7)	
Rmerge	0.081	
Rpim		
CC1/2 last shell		
**Refinement**		
Rwork / Rfree	0.200 / 0.258	0.199 / 0.260
Resolution (Å)	48.50 - 3.36	48.51 - 3.36
Reflections all	26102	27485
Reflections for Rfree	1384, 5.0%	1384, 5.0%
Bond lengths rmsd (Å)	0.007	0.009
Bond angles rmsd (°)	2.39	1.80
Mean B value (Å²)	134	133
Number of water atoms	26	26
Mean B value for water atoms (Å²)	80	80
Number of RNA atoms	8412	8412
Mean B value for RNA atoms (Å²)	135	133
Number of ligand/ion atoms	122	122
Mean B value for ligand/ion atoms (Å²)	113	115
Clashscore	26.75	7.41
Clashscore percentile (100)	-1	-1
Rotamer outliers (<1%)	0.00	0.00
Ramachandran outliers (<0.2%)	0.00	0.00
Ramachandran favored (>98%)	0.00	0.00
Residues with bad bonds (<0%)	2.81	3.57
Residues with bad angles (<0.1%)	115.56	56.89
MolProbity score	3.06	2.56

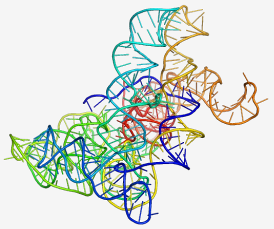

Map cc barchart:

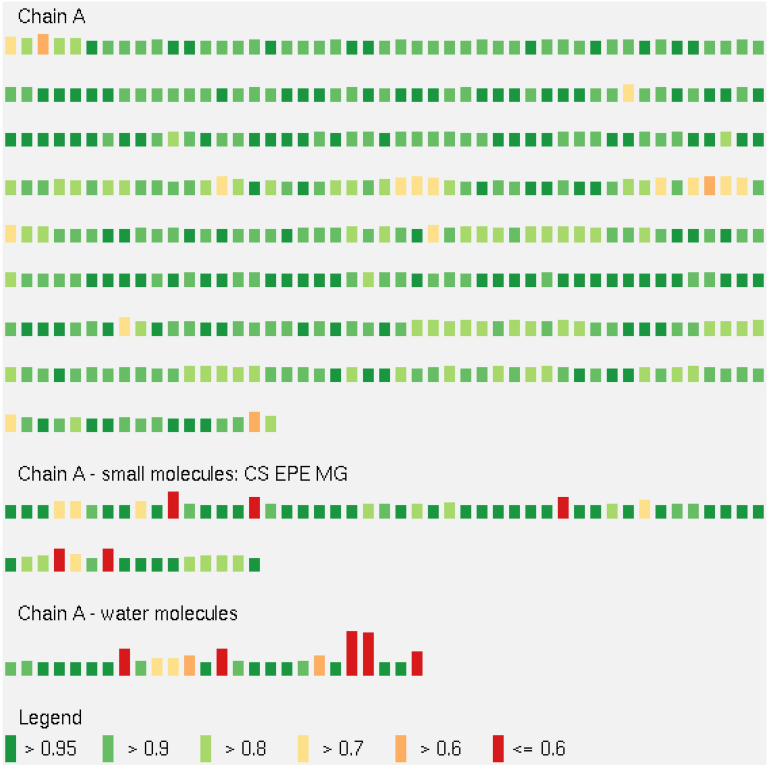

Small molecules with map:

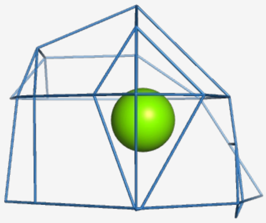

Small molecule 1: MAGNESIUM ION (MG) A 401 map cc 0.99

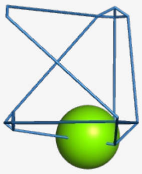

Small molecule 2: MAGNESIUM ION (MG) A 402 map cc 1.00

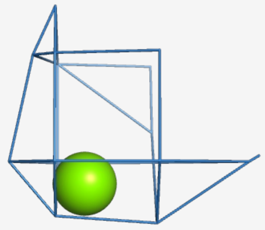

Small molecule 3: MAGNESIUM ION (MG) A 403 map cc 0.98

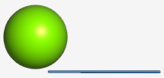

Small molecule 4: MAGNESIUM ION (MG) A 404 map cc 0.76

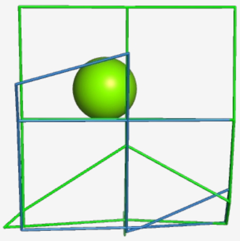

Small molecule 5: MAGNESIUM ION (MG) A 405 map cc 0.75

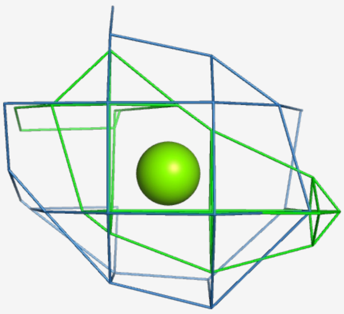

Small molecule 6: MAGNESIUM ION (MG) A 406 map cc 0.95

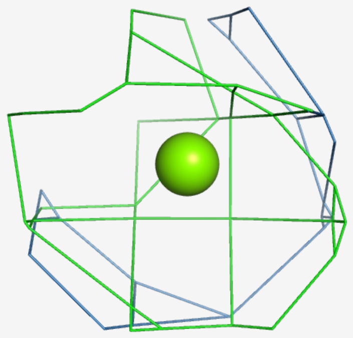

Small molecule 7: MAGNESIUM ION (MG) A 407 map cc 1.00

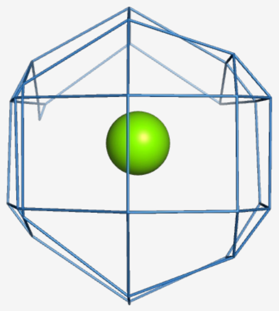

Small molecule 8: MAGNESIUM ION (MG) A 408 map cc 0.99

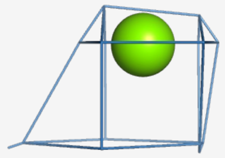

Small molecule 9: MAGNESIUM ION (MG) A 409 map cc 0.74

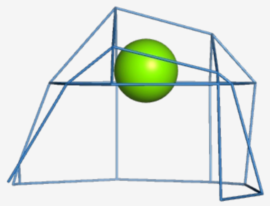

Small molecule 10: MAGNESIUM ION (MG) A 410 map cc 1.00

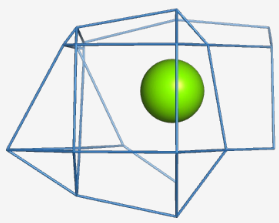

Small molecule 11: MAGNESIUM ION (MG) A 411 map cc 0.31

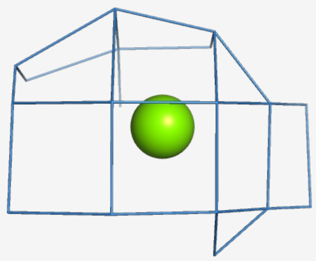

Small molecule 12: MAGNESIUM ION (MG) A 412 map cc 0.92

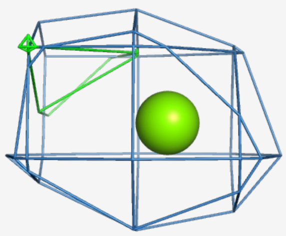

Small molecule 13: MAGNESIUM ION (MG) A 413 map cc 0.98

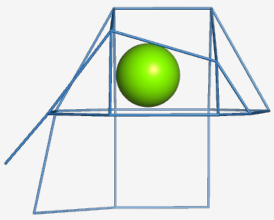

Small molecule 14: MAGNESIUM ION (MG) A 414 map cc 0.99

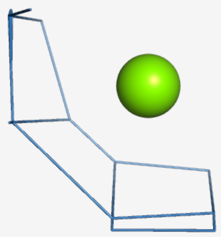

Small molecule 15: MAGNESIUM ION (MG) A 415 map cc 1.00

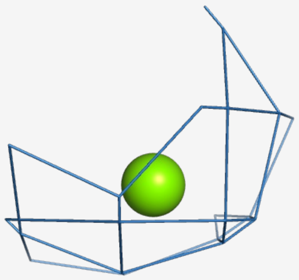

Small molecule 16: MAGNESIUM ION (MG) A 416 map cc 0.54

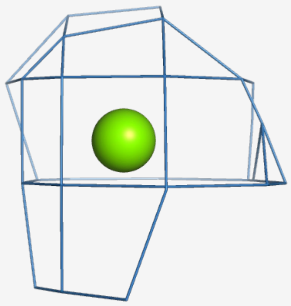

Small molecule 17: MAGNESIUM ION (MG) A 417 map cc 0.91

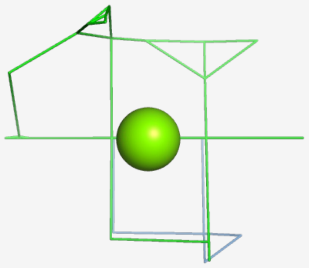

Small molecule 18: MAGNESIUM ION (MG) A 418 map cc 1.00

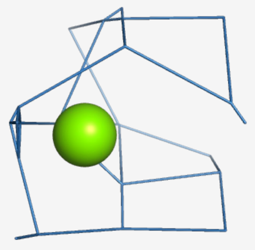

Small molecule 19: MAGNESIUM ION (MG) A 419 map cc 0.99

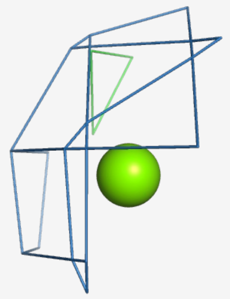

Small molecule 20: MAGNESIUM ION (MG) A 420 map cc 1.00

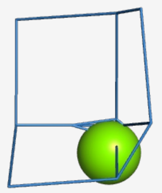

Small molecule 21: MAGNESIUM ION (MG) A 421 map cc 0.95

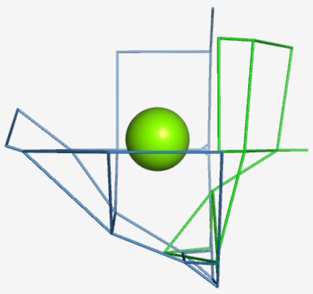

Small molecule 22: MAGNESIUM ION (MG) A 422 map cc 0.98

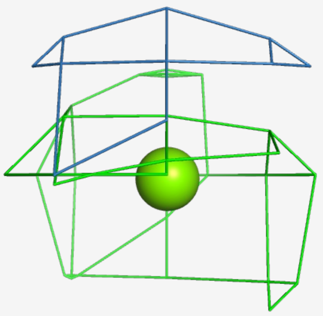

Small molecule 23: MAGNESIUM ION (MG) A 423 map cc 0.89

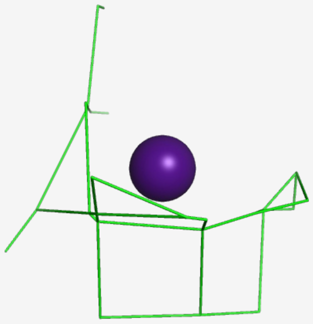

Small molecule 24: CESIUM ION (CS) A 424 map cc 0.93

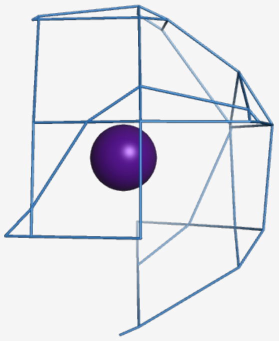

Small molecule 25: CESIUM ION (CS) A 425 map cc 0.97

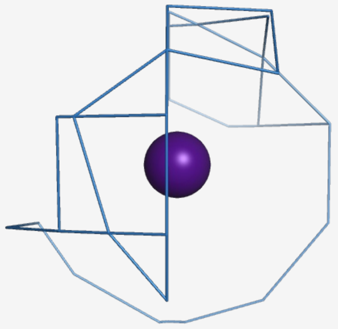

Small molecule 26: CESIUM ION (CS) A 426 map cc 0.88

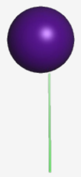

Small molecule 27: CESIUM ION (CS) A 427 map cc 0.96

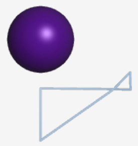

Small molecule 28: CESIUM ION (CS) A 428 map cc 0.86

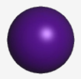

Small molecule 29: CESIUM ION (CS) A 429 map cc 1.00

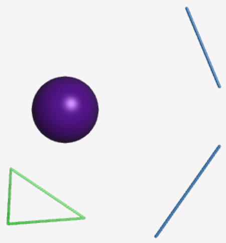

Small molecule 30: CESIUM ION (CS) A 430 map cc 1.00

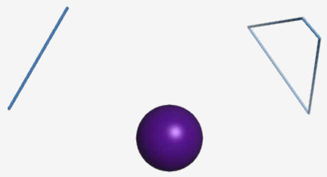

Small molecule 31: CESIUM ION (CS) A 431 map cc 1.00

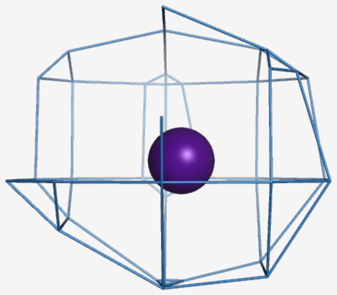

Small molecule 32: CESIUM ION (CS) A 432 map cc 1.00

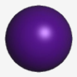

Small molecule 33: CESIUM ION (CS) A 433 map cc 0.98

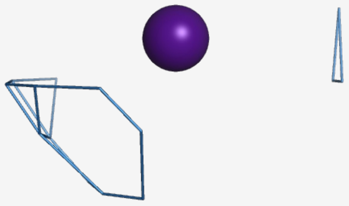

Small molecule 34: CESIUM ION (CS) A 434 map cc 0.99

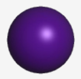

Small molecule 35: CESIUM ION (CS) A 435 map cc 0.58

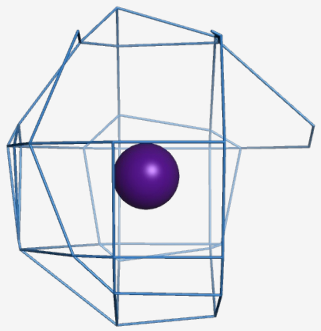

Small molecule 36: CESIUM ION (CS) A 436 map cc 0.97

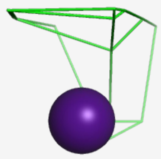

Small molecule 37: CESIUM ION (CS) A 437 map cc 1.00

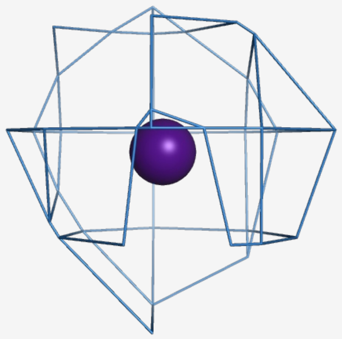

Small molecule 38: CESIUM ION (CS) A 438 map cc 0.89

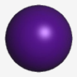

Small molecule 39: CESIUM ION (CS) A 439 map cc 0.99

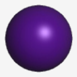

Small molecule 40: CESIUM ION (CS) A 440 map cc 0.72

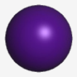

Small molecule 41: CESIUM ION (CS) A 441 map cc 1.00

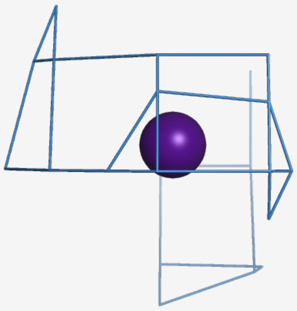

Small molecule 42: CESIUM ION (CS) A 442 map cc 0.92

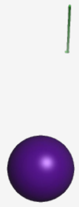

Small molecule 43: CESIUM ION (CS) A 443 map cc 0.92

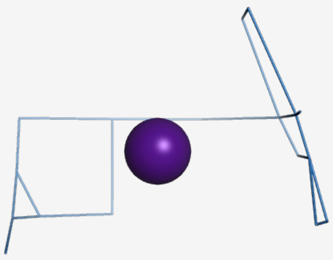

Small molecule 44: CESIUM ION (CS) A 444 map cc 1.00

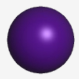

Small molecule 45: CESIUM ION (CS) A 445 map cc 0.96

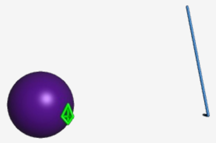

Small molecule 46: CESIUM ION (CS) A 446 map cc 1.00

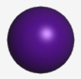

Small molecule 47: CESIUM ION (CS) A 447 map cc 0.96

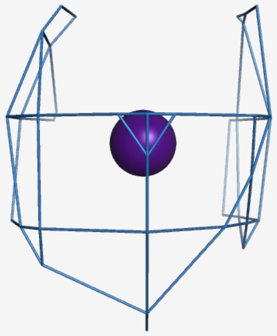

Small molecule 48: CESIUM ION (CS) A 448 map cc 0.97

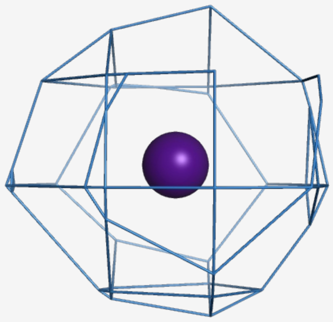

Small molecule 49: CESIUM ION (CS) A 449 map cc 0.90

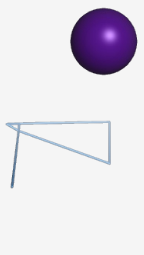

Small molecule 50: CESIUM ION (CS) A 450 map cc 0.82

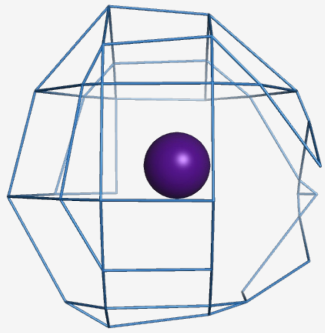

Small molecule 51: CESIUM ION (CS) A 451 map cc 0.49

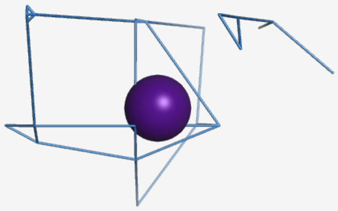

Small molecule 52: CESIUM ION (CS) A 452 map cc 0.77

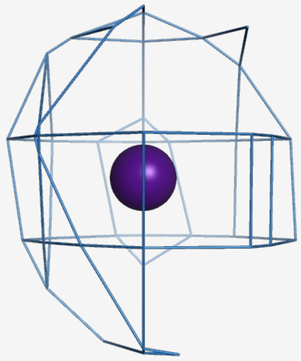

Small molecule 53: CESIUM ION (CS) A 453 map cc 0.94

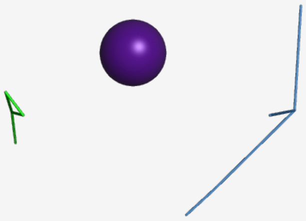

Small molecule 54: CESIUM ION (CS) A 454 map cc 0.49

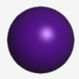

Small molecule 55: CESIUM ION (CS) A 455 map cc 1.00

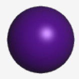

Small molecule 56: CESIUM ION (CS) A 456 map cc 1.00

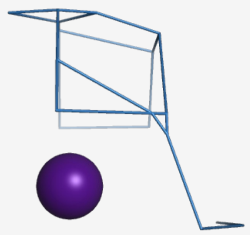

Small molecule 57: CESIUM ION (CS) A 457 map cc 1.00

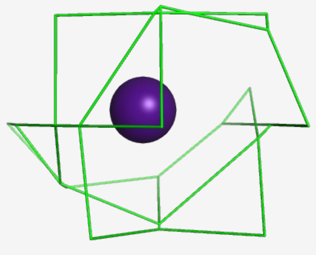

Small molecule 58: CESIUM ION (CS) A 458 map cc 0.98

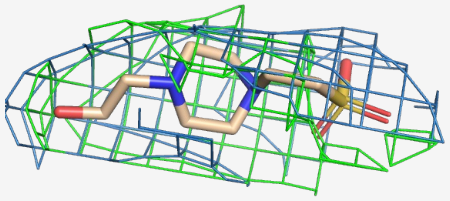

Small molecule 59: 4-(2-HYDROXYETHYL)-1-PIPERAZINEETHANESULFONICACID (EPE) A 459 map cc 0.88

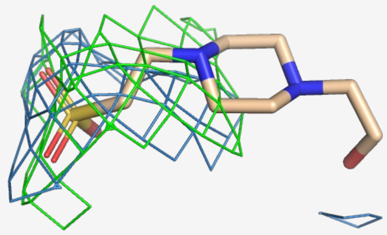

Small molecule 60: 4-(2-HYDROXYETHYL)-1-PIPERAZINEETHANESULFONICACID (EPE) A 460 map cc 0.80

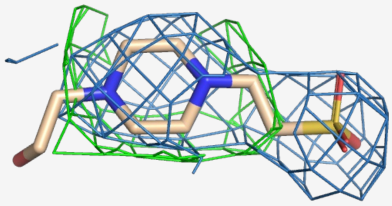

Small molecule 61: 4-(2-HYDROXYETHYL)-1-PIPERAZINEETHANESULFONICACID (EPE) A 461 map cc 0.86

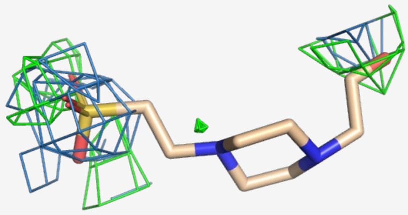

Small molecule 62: 4-(2-HYDROXYETHYL)-1-PIPERAZINEETHANESULFONICACID (EPE) A 462 map cc 0.82

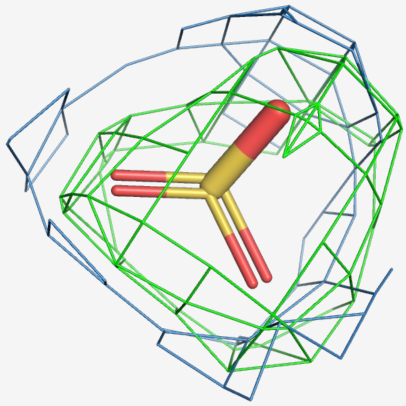

Small molecule 63: 4-(2-HYDROXYETHYL)-1-PIPERAZINEETHANESULFONICACID (EPE) A 463 map cc 1.00

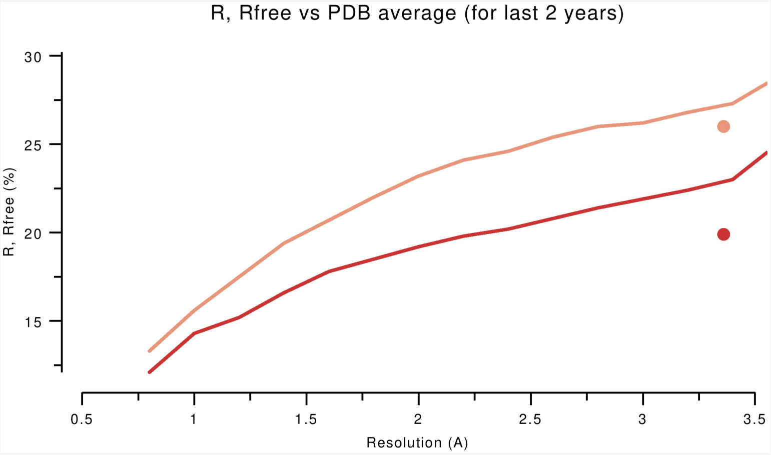

Credits:

HKL-3000
"Processing of X-ray Diffraction Data Collected in Oscillation Mode"
Z.Otwinowski, W.Minor
Methods in Enzymology, Volume 276: Macromolecular Crystallography, part A, p307-326 (1997)
"HKL-3000: the integration of data reduction and structure solution - from diffraction images to an initial model in minutes"
W.Minor, M.Cymborowski, Z.Otwinowski, M.Chruszcz
Acta Cryst. D62: 859-866 (2006)

CCP4 suite
"Overview of the CCP4 suite and current developments"
M.D.Winn et al.
Acta. Cryst. D67, 235-242 (2011)

Refmac5
"REFMAC5 for the refinement of macromolecular crystal structures"
G.N.Murshudov, P.Skubak, A.A.Lebedev, N.S.Pannu, R.A.Steiner, R.A.Nicholls, M.D.Winn, F.Long and A.A.Vagin
Acta Cryst. D67, 355-367 (2011)

Coot
"Features and Development of Coot"
P.Emsley, B.Lohkamp, W.Scott, and K.Cowtan
Acta Cryst. D66, 486-501 (2010)

Data collection and refinement statistics for project **mes_4z91** crystal **crystal1**

model **/home/asia/epe-like-validation/RESULTS/HKL_rerefine/lowres/4z91/structure_mr/build_model_1/hkl_import.pdb** vs
model **/home/asia/epe-like-validation/RESULTS/HKL_rerefine/lowres/4z91/structure_mr/build_model_1/hkl_refine_3.pdb**

Data collection		
	**4Z91**	**Re-refinement**
Resolution (Å)	40.01 - 3.39 (3.46 - 3.39)	40.01 - 3.39
Wavelength (Å)	0.9199	
Space group	P21	P21
a, b, c (Å)	105.76, 267.60, 111.37	105.76, 267.60, 111.37
α, β, γ (°)	90, 107.80, 90	90, 107.80, 90
Completeness (%)	98.1 (91.6)	100.0 (100.0)
Reflections used	156498	
<I> / <Sigma I>	12.2	
Redundancy	7.0 (6.6)	
Rmerge	0.146	
Rpim		
CC1/2 last shell		
**Refinement**		
Rwork / Rfree	0.192 / 0.247	0.207 / 0.256
Resolution (Å)	34.89 - 3.39	40.01 - 3.39
Reflections all	156498	79300
Reflections for Rfree	3986, 2.5%	2018, 2.5%
Bond lengths rmsd (Å)	0.011	0.017
Bond angles rmsd (°)	1.43	1.96
Mean B value (Å²)	116	122
Number of protein atoms	25270	25270
Mean B value for protein atoms (Å²)	116	122
Number of water atoms	81	81
Mean B value for water atoms (Å²)	91	90
Number of ligand/ion atoms	180	180
Mean B value for ligand/ion atoms (Å²)	160	172
Clashscore	13.52	13.50
Clashscore percentile (100)	-1	-1
Rotamer outliers (<1%)	2.27	2.27
Ramachandran outliers (<0.2%)	0.33	0.33
Ramachandran favored (>98%)	92.02	92.02
Residues with bad bonds (<0%)	0.00	0.03
Residues with bad angles (<0.1%)	0.00	0.03
MolProbity score	2.40	2.40

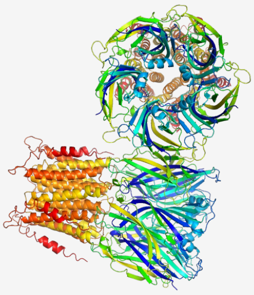

Map cc barchart:

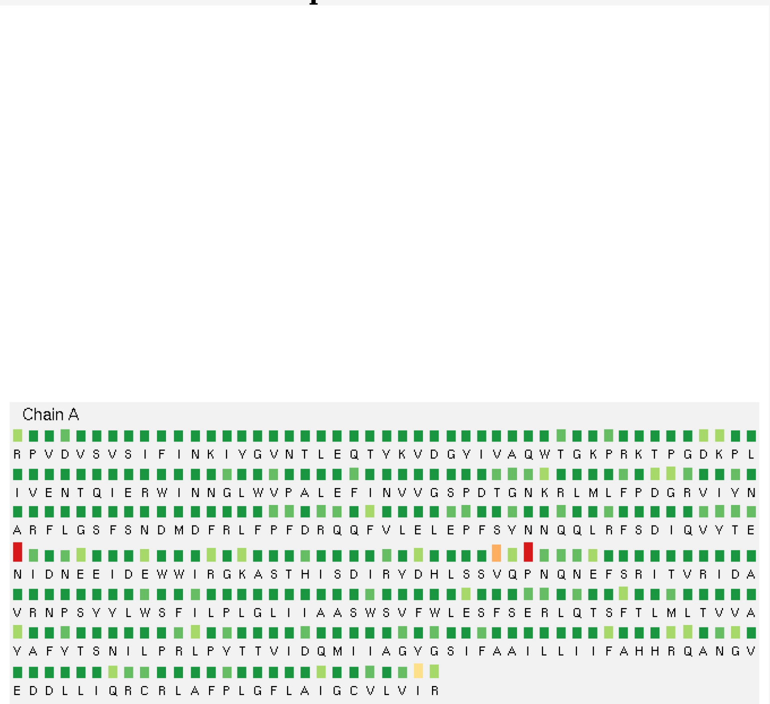

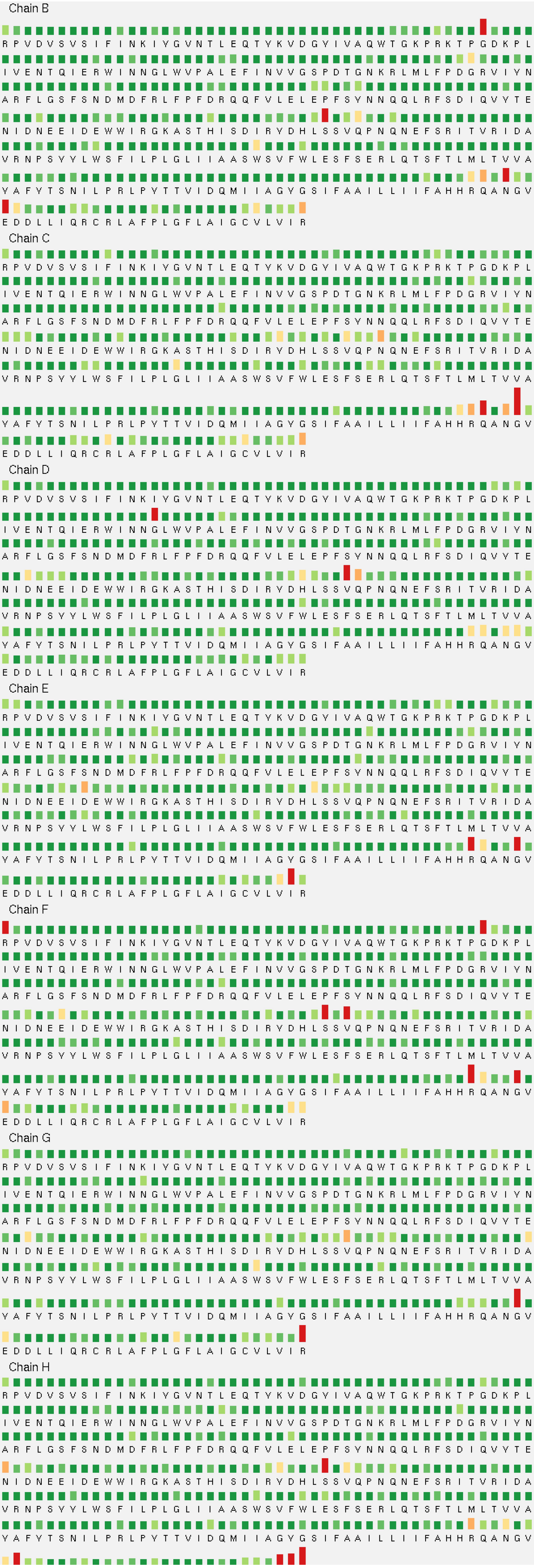

Chain B
R P V D V S V S I F I N K I Y G V N T L E Q T Y K V D G Y I V A Q W T G K P R K T P G D K P L
I V E N T Q I E R W I N N G L W V P A L E F I N V V G S P D T G N K R L M L F P D G R V I Y N
A R F L G S F S N D M D F R L F P F D R Q Q F V L E L E P F S Y N N Q Q L R F S D I Q V Y T E
N I D N E E I D E W W I R G K A S T H I S D I R Y D H L S S V Q P N Q N E F S R I T V R I D A
V R N P S Y Y L W S F I L P L G L I I A A S W S V F W L E S F S E R L Q T S F T L M L T V V A
V A F Y T S N I L P R L P Y T T V I D Q M I I A G Y G S I F A A I L L I I F A H H R Q A N G V
E D D L L I Q R C R L A F P L G F L A I G C V L V I R
Chain C
R P V D V S V S I F I N K I Y G V N T L E Q T Y K V D G Y I V A Q W T G K P R K T P G D K P L
I V E N T Q I E R W I N N G L W V P A L E F I N V V G S P D T G N K R L M L F P D G R V I Y N
A R F L G S F S N D M D F R L F P F D R Q Q F V L E L E P F S Y N N Q Q L R F S D I Q V Y T E
N I D N E E I D E W W I R G K A S T H I S D I R Y D H L S S V Q P N Q N E F S R I T V R I D A
V R N P S Y Y L W S F I L P L G L I I A A S W S V F W L E S F S E R L Q T S F T L M L T V V A
V A F Y T S N I L P R L P Y T T V I D Q M I I A G Y G S I F A A I L L I I F A H H R Q A N G V
E D D L L I Q R C R L A F P L G F L A I G C V L V I R
Chain D
R P V D V S V S I F I N K I Y G V N T L E Q T Y K V D G Y I V A Q W T G K P R K T P G D K P L
I V E N T Q I E R W I N N G L W V P A L E F I N V V G S P D T G N K R L M L F P D G R V I Y N
A R F L G S F S N D M D F R L F P F D R Q Q F V L E L E P F S Y N N Q Q L R F S D I Q V Y T E
N I D N E E I D E W W I R G K A S T H I S D I R Y D H L S S V Q P N Q N E F S R I T V R I D A
V R N P S Y Y L W S F I L P L G L I I A A S W S V F W L E S F S E R L Q T S F T L M L T V V A
V A F Y T S N I L P R L P Y T T V I D Q M I I A G Y G S I F A A I L L I I F A H H R Q A N G V
E D D L L I Q R C R L A F P L G F L A I G C V L V I R
Chain E
R P V D V S V S I F I N K I Y G V N T L E Q T Y K V D G Y I V A Q W T G K P R K T P G D K P L
I V E N T Q I E R W I N N G L W V P A L E F I N V V G S P D T G N K R L M L F P D G R V I Y N
A R F L G S F S N D M D F R L F P F D R Q Q F V L E L E P F S Y N N Q Q L R F S D I Q V Y T E
N I D N E E I D E W W I R G K A S T H I S D I R Y D H L S S V Q P N Q N E F S R I T V R I D A
V R N P S Y Y L W S F I L P L G L I I A A S W S V F W L E S F S E R L Q T S F T L M L T V V A
V A F Y T S N I L P R L P Y T T V I D Q M I I A G Y G S I F A A I L L I I F A H H R Q A N G V
E D D L L I Q R C R L A F P L G F L A I G C V L V I R
Chain F
R P V D V S V S I F I N K I Y G V N T L E Q T Y K V D G Y I V A Q W T G K P R K T P G D K P L
I V E N T Q I E R W I N N G L W V P A L E F I N V V G S P D T G N K R L M L F P D G R V I Y N
A R F L G S F S N D M D F R L F P F D R Q Q F V L E L E P F S Y N N Q Q L R F S D I Q V Y T E
N I D N E E I D E W W I R G K A S T H I S D I R Y D H L S S V Q P N Q N E F S R I T V R I D A
V R N P S Y Y L W S F I L P L G L I I A A S W S V F W L E S F S E R L Q T S F T L M L T V V A
V A F Y T S N I L P R L P Y T T V I D Q M I I A G Y G S I F A A I L L I I F A H H R Q A N G V
E D D L L I Q R C R L A F P L G F L A I G C V L V I R
Chain G
R P V D V S V S I F I N K I Y G V N T L E Q T Y K V D G Y I V A Q W T G K P R K T P G D K P L
I V E N T Q I E R W I N N G L W V P A L E F I N V V G S P D T G N K R L M L F P D G R V I Y N
A R F L G S F S N D M D F R L F P F D R Q Q F V L E L E P F S Y N N Q Q L R F S D I Q V Y T E
N I D N E E I D E W W I R G K A S T H I S D I R Y D H L S S V Q P N Q N E F S R I T V R I D A
V R N P S Y Y L W S F I L P L G L I I A A S W S V F W L E S F S E R L Q T S F T L M L T V V A
V A F Y T S N I L P R L P Y T T V I D Q M I I A G Y G S I F A A I L L I I F A H H R Q A N G V
E D D L L I Q R C R L A F P L G F L A I G C V L V I R
Chain H
R P V D V S V S I F I N K I Y G V N T L E Q T Y K V D G Y I V A Q W T G K P R K T P G D K P L
I V E N T Q I E R W I N N G L W V P A L E F I N V V G S P D T G N K R L M L F P D G R V I Y N
A R F L G S F S N D M D F R L F P F D R Q Q F V L E L E P F S Y N N Q Q L R F S D I Q V Y T E
N I D N E E I D E W W I R G K A S T H I S D I R Y D H L S S V Q P N Q N E F S R I T V R I D A
V R N P S Y Y L W S F I L P L G L I I A A S W S V F W L E S F S E R L Q T S F T L M L T V V A
V A F Y T S N I L P R L P Y T T V I D Q M I I A G Y G S I F A A I L L I I F A H H R Q A N G V

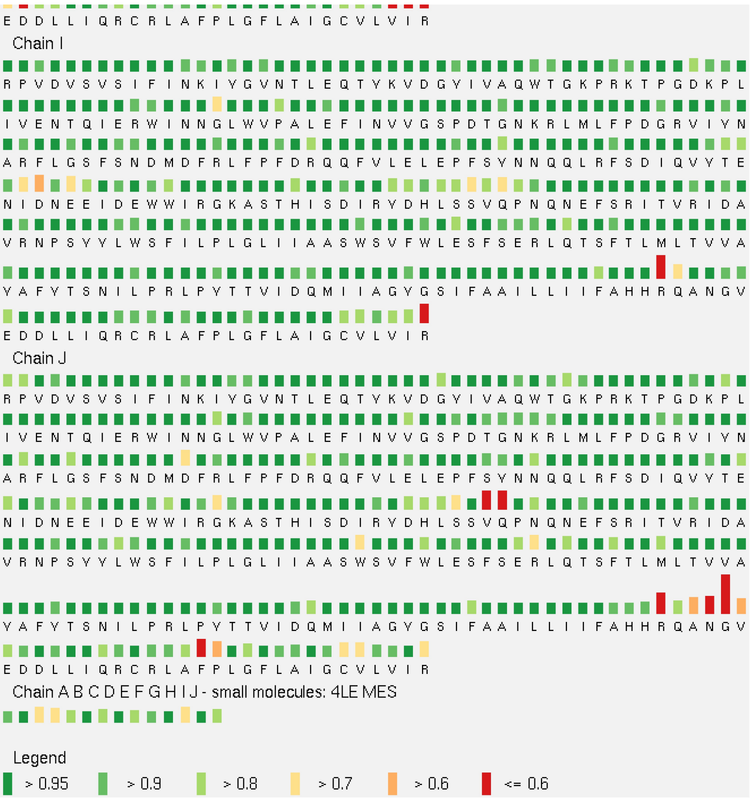

Small molecules with map:

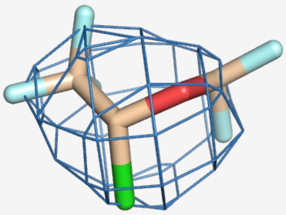

Small molecule 1: (2R)-2-chloro-2-(difluoromethoxy)-1,1,1-trifluoroethane (4LE) A 401 map cc 0.95

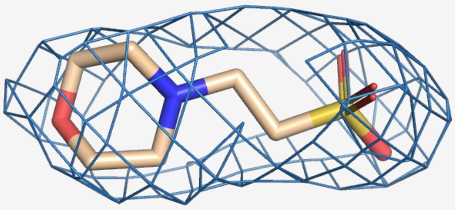

Small molecule 2: 2-(N-MORPHOLINO)-ETHANESULFONIC ACID (MES) A 402 map cc 0.96

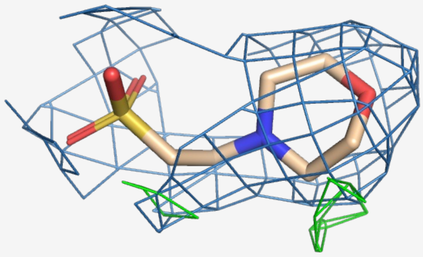

Small molecule 3: 2-(N-MORPHOLINO)-ETHANESULFONIC ACID (MES) B 401 map cc 0.73

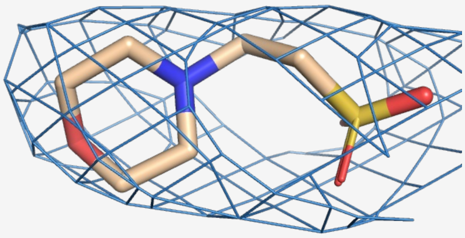

Small molecule 4: 2-(N-MORPHOLINO)-ETHANESULFONIC ACID (MES) C 401 map cc 0.78

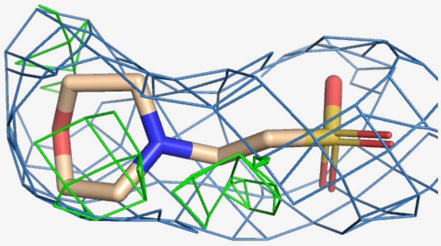

Small molecule 5: 2-(N-MORPHOLINO)-ETHANESULFONIC ACID (MES) D 401 map cc 0.88

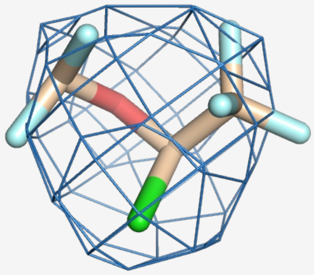

Small molecule 6: (2R)-2-chloro-2-(difluoromethoxy)-1,1,1-trifluoroethane (4LE) E 401 map cc 0.98

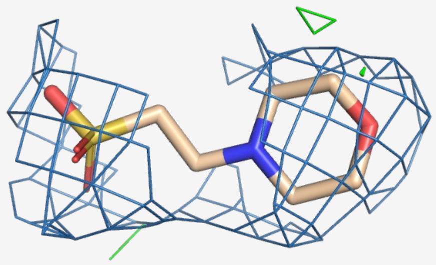

Small molecule 7: 2-(N-MORPHOLINO)-ETHANESULFONIC ACID (MES) E 402 map cc 0.81

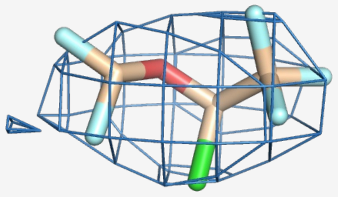

Small molecule 8: (2R)-2-chloro-2-(difluoromethoxy)-1,1,1-trifluoroethane (4LE) F 401 map cc 0.99

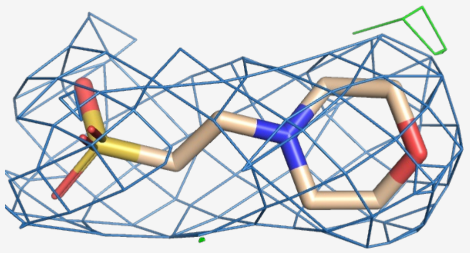

Small molecule 9: 2-(N-MORPHOLINO)-ETHANESULFONIC ACID (MES) F 402 map cc 0.90

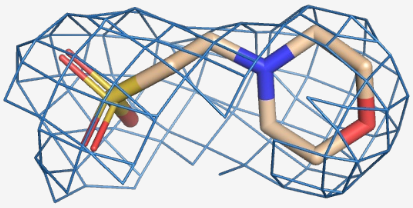

Small molecule 10: 2-(N-MORPHOLINO)-ETHANESULFONIC ACID (MES) G 401 map cc 0.94

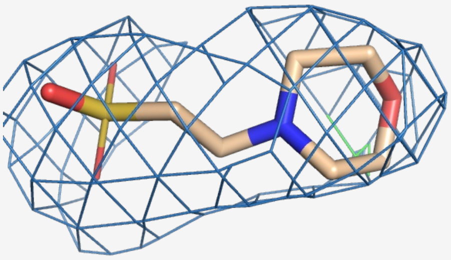

Small molecule 11: 2-(N-MORPHOLINO)-ETHANESULFONIC ACID (MES) H 401 map cc 0.96

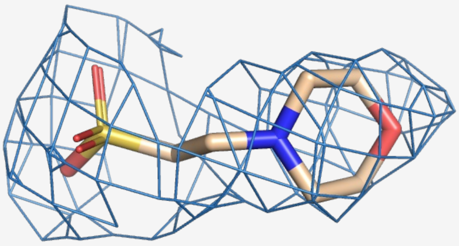

Small molecule 12: 2-(N-MORPHOLINO)-ETHANESULFONIC ACID (MES) I 401 map cc 0.72

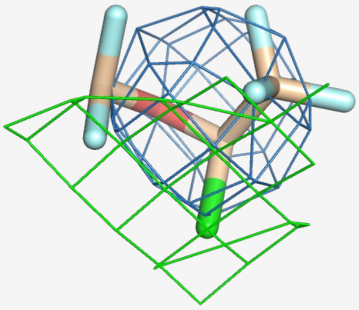

Small molecule 13: (2R)-2-chloro-2-(difluoromethoxy)-1,1,1-trifluoroethane (4LE) J 401 map cc 0.97

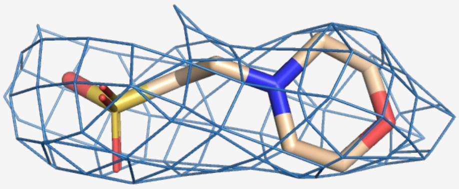

Small molecule 14: 2-(N-MORPHOLINO)-ETHANESULFONIC ACID (MES) J 402 map cc 0.84

Credits:

HKL-3000
"Processing of X-ray Diffraction Data Collected in Oscillation Mode"
Z.Otwinowski, W.Minor
Methods in Enzymology, Volume 276: Macromolecular Crystallography, part A, p307-326 (1997)
"HKL-3000: the integration of data reduction and structure solution - from diffraction images to an initial model in minutes"
W.Minor, M.Cymborowski, Z.Otwinowski, M.Chruszcz
Acta Cryst. D62: 859-866 (2006)

CCP4 suite
"Overview of the CCP4 suite and current developments"
M.D.Winn et al.
Acta. Cryst. D67, 235-242 (2011)

Refmac5
"REFMAC5 for the refinement of macromolecular crystal structures"
G.N.Murshudov, P.Skubak, A.A.Lebedev, N.S.Pannu, R.A.Steiner, R.A.Nicholls, M.D.Winn, F.Long and A.A.Vagin
Acta Cryst. D67, 355-367 (2011)

Coot
"Features and Development of Coot"
P.Emsley, B.Lohkamp, W.Scott, and K.Cowtan
Acta Cryst. D66, 486-501 (2010)

Data collection and refinement statistics for project **mes_4z91** crystal **crystal1**

model **/home/asia/epe-like-validation/RESULTS/HKL_rerefine/lowres/4z91/structure_mr/build_model_1/hkl_import.pdb** vs
model **/home/asia/epe-like-validation/RESULTS/HKL_rerefine/lowres/4z91/structure_mr/build_model_1/hkl_refine_4.pdb**

**Data collection**		
	**4Z91**	**Re-refinement**
Resolution (Å)	40.01 - 3.39 (3.46 - 3.39)	40.01 - 3.39
Wavelength (Å)	0.9199	
Space group	P21	P21
a, b, c (Å)	105.76, 267.60, 111.37	105.76, 267.60, 111.37
α, β, γ (°)	90, 107.80, 90	90, 107.80, 90
Completeness (%)	98.1 (91.6)	100.0 (100.0)
Reflections used	156498	
<I> / <Sigma I>	12.2	
Redundancy	7.0 (6.6)	
Rmerge	0.146	
Rpim		
CC1/2 last shell		
**Refinement**		
Rwork / Rfree	0.192 / 0.247	0.186 / 0.251
Resolution (Å)	34.89 - 3.39	40.01 - 3.39
Reflections all	156498	79300
Reflections for Rfree	3986, 2.5%	2018, 2.5%
Bond lengths rmsd (Å)	0.011	0.007
Bond angles rmsd (°)	1.43	1.65
Mean B value (Å²)	116	127
Number of protein atoms	25270	25270
Mean B value for protein atoms (Å²)	116	127
Number of water atoms	81	81
Mean B value for water atoms (Å²)	91	89
Number of ligand/ion atoms	180	180
Mean B value for ligand/ion atoms (Å²)	160	180
Clashscore	13.52	7.78
Clashscore percentile (100)	-1	-1
Rotamer outliers (<1%)	2.27	4.95
Ramachandran outliers (<0.2%)	0.33	2.44
Ramachandran favored (>98%)	92.02	87.10
Residues with bad bonds (<0%)	0.00	0.32
Residues with bad angles (<0.1%)	0.00	1.26
MolProbity score	2.40	2.57

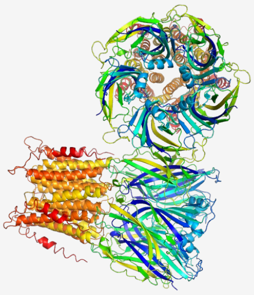

Map cc barchart:

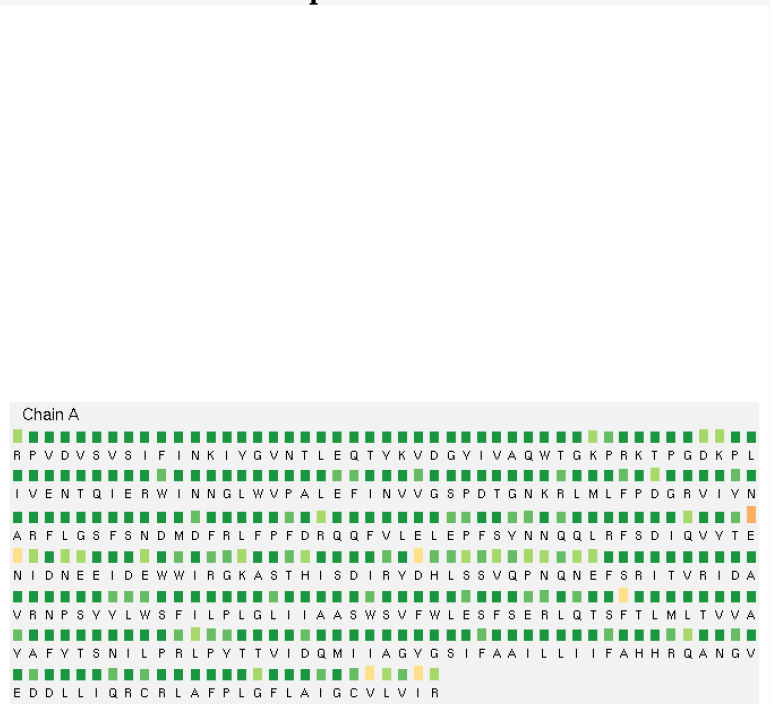

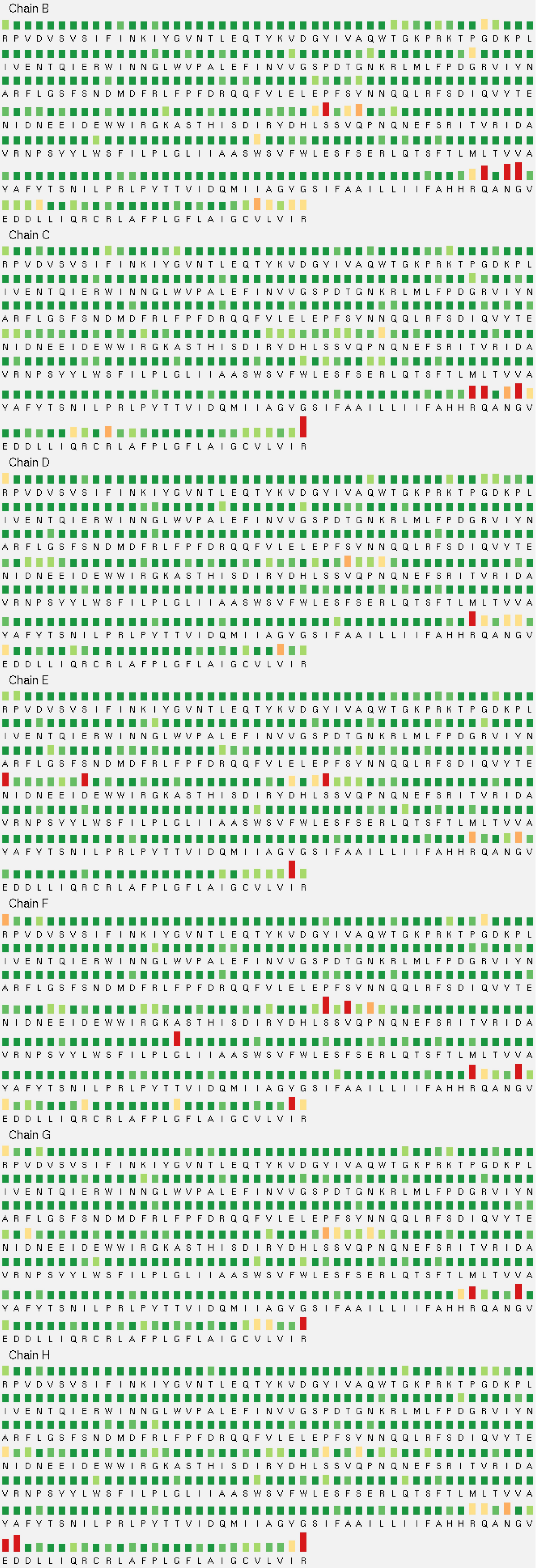
Chain B
R P V D V S V S I F I N K I Y G V N T L E Q T Y K V D G Y I V A Q W T G K P R K T P G D K P L
I V E N T Q I E R W I N N G L W V P A L E F I N V V G S P D T G N K R L M L F P D G R V I Y N
A R F L G S F S N D M D F R L F P F D R Q Q F V L E L E P F S Y N N Q Q L R F S D I Q V Y T E
N I D N E E I D E W W I R G K A S T H I S D I R Y D H L S S V Q P N Q N E F S R I T V R I D A
V R N P S Y Y L W S F I L P L G L I I A A S W S V F W L E S F S E R L Q T S F T L M L T V V A
V A F Y T S N I L P R L P Y T T V I D Q M I I A G Y G S I F A A I L L I I F A H H R Q A N G V
E D D L L I Q R C R L A F P L G F L A I G C V L V I R
Chain C
R P V D V S V S I F I N K I Y G V N T L E Q T Y K V D G Y I V A Q W T G K P R K T P G D K P L
I V E N T Q I E R W I N N G L W V P A L E F I N V V G S P D T G N K R L M L F P D G R V I Y N
A R F L G S F S N D M D F R L F P F D R Q Q F V L E L E P F S Y N N Q Q L R F S D I Q V Y T E
N I D N E E I D E W W I R G K A S T H I S D I R Y D H L S S V Q P N Q N E F S R I T V R I D A
V R N P S Y Y L W S F I L P L G L I I A A S W S V F W L E S F S E R L Q T S F T L M L T V V A
V A F Y T S N I L P R L P Y T T V I D Q M I I A G Y G S I F A A I L L I I F A H H R Q A N G V
E D D L L I Q R C R L A F P L G F L A I G C V L V I R
Chain D
R P V D V S V S I F I N K I Y G V N T L E Q T Y K V D G Y I V A Q W T G K P R K T P G D K P L
I V E N T Q I E R W I N N G L W V P A L E F I N V V G S P D T G N K R L M L F P D G R V I Y N
A R F L G S F S N D M D F R L F P F D R Q Q F V L E L E P F S Y N N Q Q L R F S D I Q V Y T E
N I D N E E I D E W W I R G K A S T H I S D I R Y D H L S S V Q P N Q N E F S R I T V R I D A
V R N P S Y Y L W S F I L P L G L I I A A S W S V F W L E S F S E R L Q T S F T L M L T V V A
V A F Y T S N I L P R L P Y T T V I D Q M I I A G Y G S I F A A I L L I I F A H H R Q A N G V
E D D L L I Q R C R L A F P L G F L A I G C V L V I R
Chain E
R P V D V S V S I F I N K I Y G V N T L E Q T Y K V D G Y I V A Q W T G K P R K T P G D K P L
I V E N T Q I E R W I N N G L W V P A L E F I N V V G S P D T G N K R L M L F P D G R V I Y N
A R F L G S F S N D M D F R L F P F D R Q Q F V L E L E P F S Y N N Q Q L R F S D I Q V Y T E
N I D N E E I D E W W I R G K A S T H I S D I R Y D H L S S V Q P N Q N E F S R I T V R I D A
V R N P S Y Y L W S F I L P L G L I I A A S W S V F W L E S F S E R L Q T S F T L M L T V V A
V A F Y T S N I L P R L P Y T T V I D Q M I I A G Y G S I F A A I L L I I F A H H R Q A N G V
E D D L L I Q R C R L A F P L G F L A I G C V L V I R
Chain F
R P V D V S V S I F I N K I Y G V N T L E Q T Y K V D G Y I V A Q W T G K P R K T P G D K P L
I V E N T Q I E R W I N N G L W V P A L E F I N V V G S P D T G N K R L M L F P D G R V I Y N
A R F L G S F S N D M D F R L F P F D R Q Q F V L E L E P F S Y N N Q Q L R F S D I Q V Y T E
N I D N E E I D E W W I R G K A S T H I S D I R Y D H L S S V Q P N Q N E F S R I T V R I D A
V R N P S Y Y L W S F I L P L G L I I A A S W S V F W L E S F S E R L Q T S F T L M L T V V A
V A F Y T S N I L P R L P Y T T V I D Q M I I A G Y G S I F A A I L L I I F A H H R Q A N G V
E D D L L I Q R C R L A F P L G F L A I G C V L V I R
Chain G
R P V D V S V S I F I N K I Y G V N T L E Q T Y K V D G Y I V A Q W T G K P R K T P G D K P L
I V E N T Q I E R W I N N G L W V P A L E F I N V V G S P D T G N K R L M L F P D G R V I Y N
A R F L G S F S N D M D F R L F P F D R Q Q F V L E L E P F S Y N N Q Q L R F S D I Q V Y T E
N I D N E E I D E W W I R G K A S T H I S D I R Y D H L S S V Q P N Q N E F S R I T V R I D A
V R N P S Y Y L W S F I L P L G L I I A A S W S V F W L E S F S E R L Q T S F T L M L T V V A
V A F Y T S N I L P R L P Y T T V I D Q M I I A G Y G S I F A A I L L I I F A H H R Q A N G V
E D D L L I Q R C R L A F P L G F L A I G C V L V I R
Chain H
R P V D V S V S I F I N K I Y G V N T L E Q T Y K V D G Y I V A Q W T G K P R K T P G D K P L
I V E N T Q I E R W I N N G L W V P A L E F I N V V G S P D T G N K R L M L F P D G R V I Y N
A R F L G S F S N D M D F R L F P F D R Q Q F V L E L E P F S Y N N Q Q L R F S D I Q V Y T E
N I D N E E I D E W W I R G K A S T H I S D I R Y D H L S S V Q P N Q N E F S R I T V R I D A
V R N P S Y Y L W S F I L P L G L I I A A S W S V F W L E S F S E R L Q T S F T L M L T V V A
V A F Y T S N I L P R L P Y T T V I D Q M I I A G Y G S I F A A I L L I I F A H H R Q A N G V
E D D L L I Q R C R L A F P L G F L A I G C V L V I R

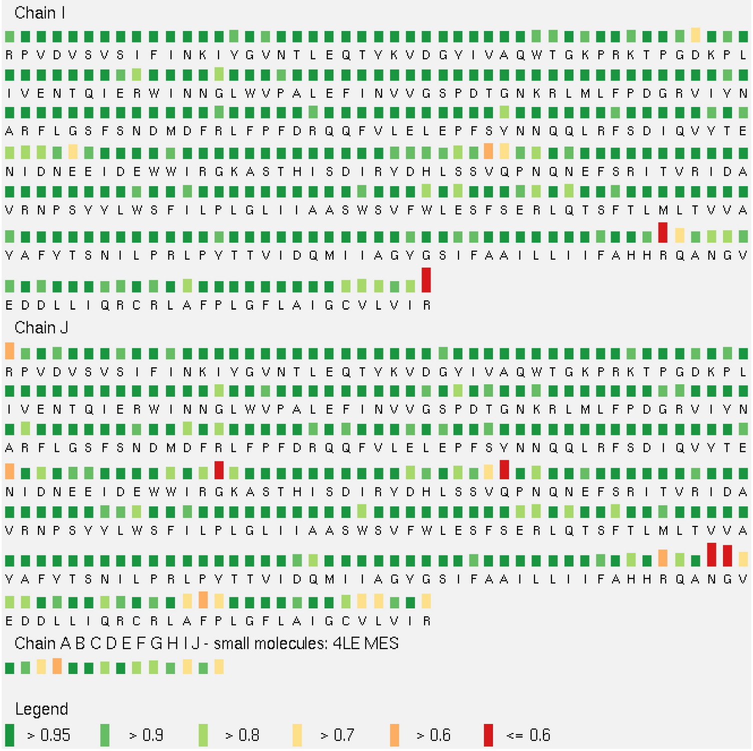

Small molecules with map:

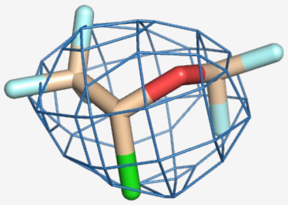

Small molecule 1: (2R)-2-chloro-2-(difluoromethoxy)-1,1,1-trifluoroethane (4LE) A 401 map cc 0.98

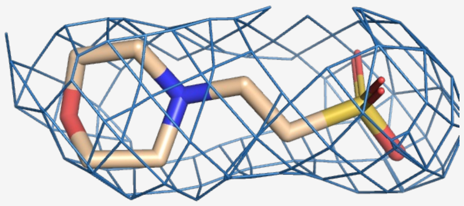

Small molecule 2: 2-(N-MORPHOLINO)-ETHANESULFONIC ACID (MES) A 402 map cc 0.93

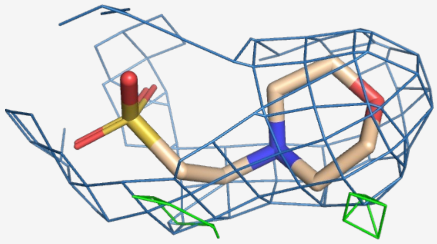

Small molecule 3: 2-(N-MORPHOLINO)-ETHANESULFONIC ACID (MES) B 401 map cc 0.78

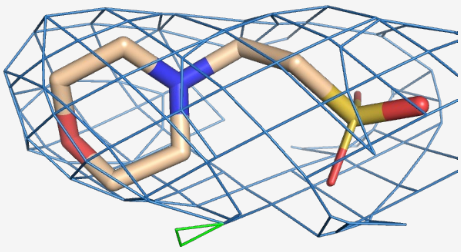

Small molecule 4: 2-(N-MORPHOLINO)-ETHANESULFONIC ACID (MES) C 401 map cc 0.70

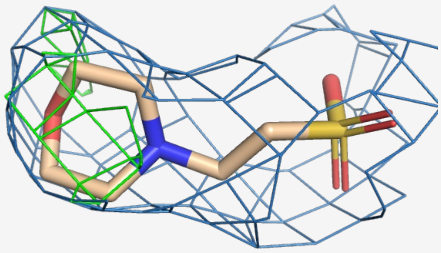

Small molecule 5: 2-(N-MORPHOLINO)-ETHANESULFONIC ACID (MES) D 401 map cc 0.96

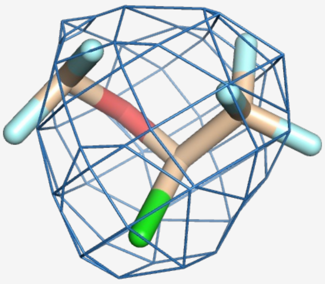

Small molecule 6: (2R)-2-chloro-2-(difluoromethoxy)-1,1,1-trifluoroethane (4LE) E 401 map cc 0.97

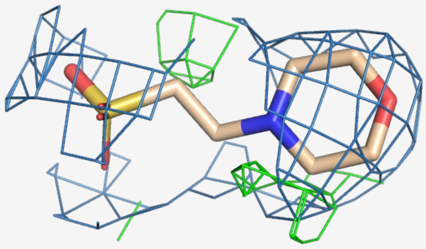

Small molecule 7: 2-(N-MORPHOLINO)-ETHANESULFONIC ACID (MES) E 402 map cc 0.89

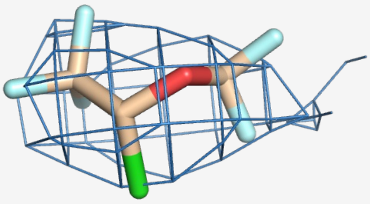

Small molecule 8: (2R)-2-chloro-2-(difluoromethoxy)-1,1,1-trifluoroethane (4LE) F 401 map cc 0.97

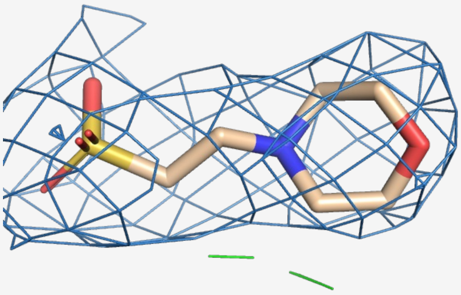

Small molecule 9: 2-(N-MORPHOLINO)-ETHANESULFONIC ACID (MES) F 402 map cc 0.89

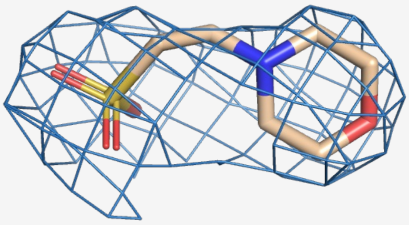

Small molecule 10: 2-(N-MORPHOLINO)-ETHANESULFONIC ACID (MES) G 401 map cc 0.84

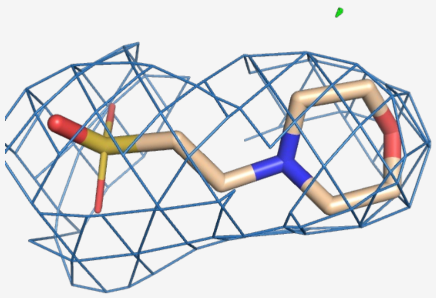

Small molecule 11: 2-(N-MORPHOLINO)-ETHANESULFONIC ACID (MES) H 401 map cc 0.94

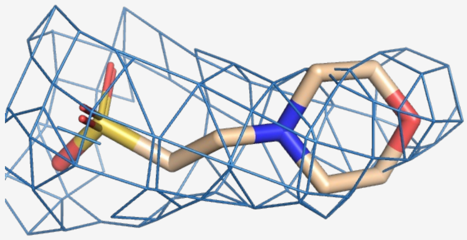

Small molecule 12: 2-(N-MORPHOLINO)-ETHANESULFONIC ACID (MES) I 401 map cc 0.76

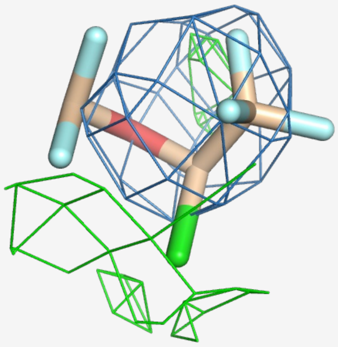

Small molecule 13: (2R)-2-chloro-2-(difluoromethoxy)-1,1,1-trifluoroethane (4LE) J 401 map cc 0.94

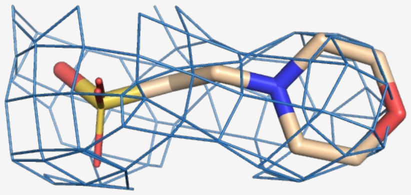

Small molecule 14: 2-(N-MORPHOLINO)-ETHANESULFONIC ACID (MES) J 402 map cc 0.78

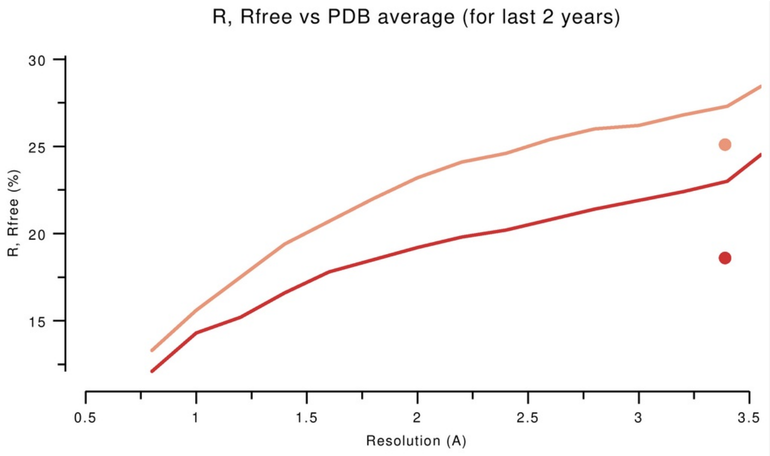

Credits:

HKL-3000
"Processing of X-ray Diffraction Data Collected in Oscillation Mode"
Z.Otwinowski, W.Minor
Methods in Enzymology, Volume 276: Macromolecular Crystallography, part A, p307-326 (1997)
"HKL-3000: the integration of data reduction and structure solution - from diffraction images to an initial model in minutes"
W.Minor, M.Cymborowski, Z.Otwinowski, M.Chruszcz
Acta Cryst. D62: 859-866 (2006)

CCP4 suite
"Overview of the CCP4 suite and current developments"
M.D.Winn et al.
Acta. Cryst. D67, 235-242 (2011)

Refmac5
"REFMAC5 for the refinement of macromolecular crystal structures"
G.N.Murshudov, P.Skubak, A.A.Lebedev, N.S.Pannu, R.A.Steiner, R.A.Nicholls, M.D.Winn, F.Long and A.A.Vagin
Acta Cryst. D67, 355-367 (2011)

Coot
"Features and Development of Coot"
P.Emsley, B.Lohkamp, W.Scott, and K.Cowtan
Acta Cryst. D66, 486-501 (2010)

Data collection and refinement statistics for project **mes_3e9f** crystal **crystal1**

model **/home/asia/epe-like-validation/RESULTS/HKL_rerefine/flat/3e9f/structure_mr/build_model_1/hkl_import.pdb** vs
model **/home/asia/epe-like-validation/RESULTS/HKL_rerefine/flat/3e9f/structure_mr/build_model_1/hkl_refine_3.pdb**

Data collection

	3E9F	Re-refinement
Resolution (Å)	50.00 - 1.80 (1.86 - 1.80)	25.81 - 1.80
Wavelength (Å)	1.0000	
Space group	C2221	C2221
a, b, c (Å)	34.62, 130.34, 56.21	34.62, 130.34, 56.21
α, β, γ (°)	90, 90, 90	90, 90, 90
Completeness (%)	99.3	100.0 (100.0)
Reflections used	12156	
<I> / <Sigma I>		
Redundancy	6.5 (5.8)	
Rmerge	0.110	
Rpim		
CC1/2 last shell		
Wilson B factor (Å^2)	22.0	

Refinement

	3E9F	Re-refinement
Rwork / Rfree	0.188 / 0.217	0.182 / 0.174
Resolution (Å)	25.81 - 1.80	25.81 - 1.80
Reflections all	12141	12142
Reflections for Rfree	610, 5.0%	580, 4.8%
Bond lengths rmsd (Å)	0.015	0.018
Bond angles rmsd (°)	1.57	1.87
Mean B value (Å^2)	26	28
Number of protein atoms	818	818
Mean B value for protein atoms (Å^2)	24	27
Number of water atoms (expected)	123 (123)	123 (123)
Mean B value for water atoms (Å^2)	38	40
Number of ligand/ion atoms	12	12
Mean B value for ligand/ion atoms (Å^2)	37	38
Clashscore	3.66	3.65
Clashscore percentile (100)	-1	-1
Rotamer outliers (<1%)	2.33	2.33
Ramachandran outliers (<0.2%)	0.00	0.00
Ramachandran favored (>98%)	97.89	97.89
Residues with bad bonds (<0%)	1.01	2.02
Residues with bad angles (<0.1%)	0.00	0.00
MolProbity score	1.46	1.46

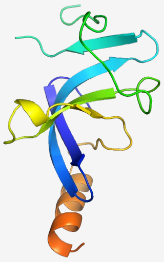

Map cc barchart:

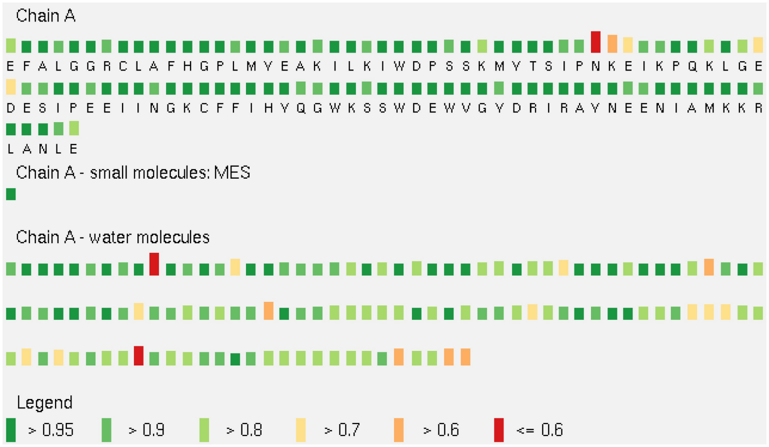

Small molecules with map:

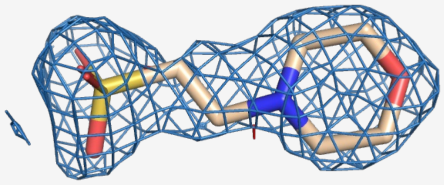

Small molecule 1: 2-(N-MORPHOLINO)-ETHANESULFONIC ACID (MES) A 122 map cc 0.97

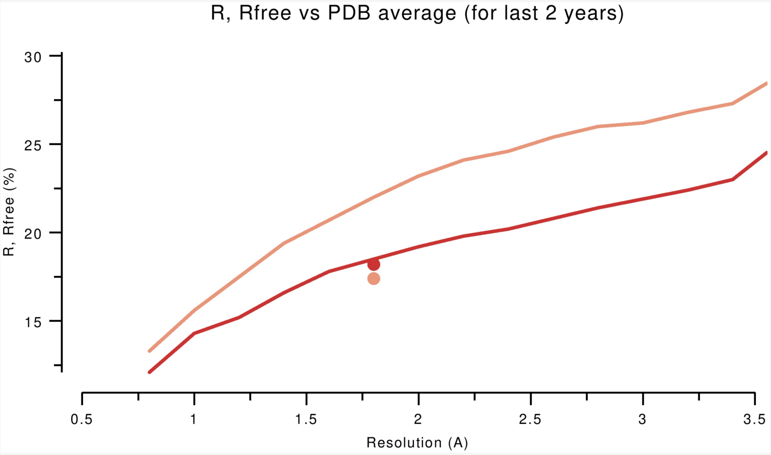

Credits:

HKL-3000
"Processing of X-ray Diffraction Data Collected in Oscillation Mode"
Z.Otwinowski, W.Minor
Methods in Enzymology, Volume 276: Macromolecular Crystallography, part A, p307-326 (1997)
"HKL-3000: the integration of data reduction and structure solution - from diffraction images to an initial model in minutes"
W.Minor, M.Cymborowski, Z.Otwinowski, M.Chruszcz
Acta Cryst. D62: 859-866 (2006)

CCP4 suite
"Overview of the CCP4 suite and current developments"
M.D.Winn et al.
Acta. Cryst. D67, 235-242 (2011)

Refmac5
"REFMAC5 for the refinement of macromolecular crystal structures"
G.N.Murshudov, P.Skubak, A.A.Lebedev, N.S.Pannu, R.A.Steiner, R.A.Nicholls, M.D.Winn, F.Long and A.A.Vagin
Acta Cryst. D67, 355-367 (2011)

Coot
"Features and Development of Coot"
P.Emsley, B.Lohkamp, W.Scott, and K.Cowtan
Acta Cryst. D66, 486-501 (2010)

Data collection and refinement statistics for project **mes_3e9f** crystal **crystal1**

model **/home/asia/epe-like-validation/RESULTS/HKL_rerefine/flat/3e9f/structure_mr/build_model_1/hkl_import.pdb** vs
model **/home/asia/epe-like-validation/RESULTS/HKL_rerefine/flat/3e9f/structure_mr/build_model_1/hkl_refine_5.pdb**

Data collection

	3E9F	Re-refinement
Resolution (Å)	50.00 - 1.80 (1.86 - 1.80)	25.81 - 1.80
Wavelength (Å)	1.0000	
Space group	C2221	C2221
a, b, c (Å)	34.62, 130.34, 56.21	34.62, 130.34, 56.21
α, β, γ (°)	90, 90, 90	90, 90, 90
Completeness (%)	99.3	100.0 (100.0)
Reflections used	12156	
<I> / <Sigma I>		
Redundancy	6.5 (5.8)	
Rmerge	0.110	
Rpim		
CC1/2 last shell		
Wilson B factor (Å^2)	22.0	

Refinement

	3E9F	Re-refinement
Rwork / Rfree	0.188 / 0.217	0.175 / 0.193
Resolution (Å)	25.81 - 1.80	25.81 - 1.80
Reflections all	12141	12142
Reflections for Rfree	610, 5.0%	580, 4.8%
Bond lengths rmsd (Å)	0.015	0.012
Bond angles rmsd (°)	1.57	1.74
Mean B value (Å^2)	26	28
Number of protein atoms	818	818
Mean B value for protein atoms (Å^2)	24	26
Number of water atoms (expected)	123 (123)	123 (123)
Mean B value for water atoms (Å^2)	38	39
Number of ligand/ion atoms	12	12
Mean B value for ligand/ion atoms (Å^2)	37	38
Clashscore	3.66	3.65
Clashscore percentile (100)	-1	-1
Rotamer outliers (<1%)	2.33	0.00
Ramachandran outliers (<0.2%)	0.00	0.00
Ramachandran favored (>98%)	97.89	100.00
Residues with bad bonds (<0%)	1.01	2.02
Residues with bad angles (<0.1%)	0.00	1.01
MolProbity score	1.46	1.16

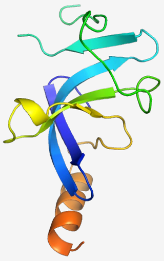

Map cc barchart:

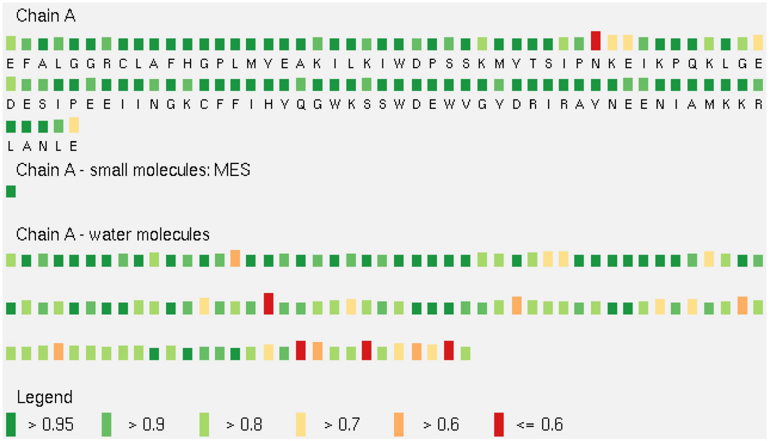

Small molecules with map:

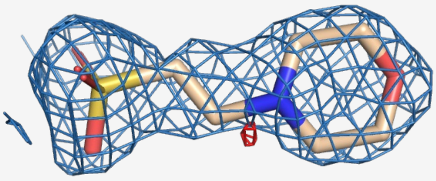

Small molecule 1: 2-(N-MORPHOLINO)-ETHANESULFONIC ACID (MES) A 122 map cc 0.97

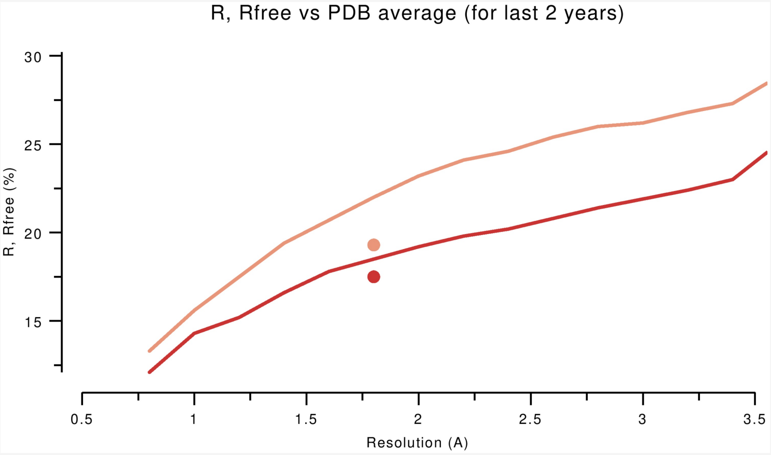

Credits:

HKL-3000
"Processing of X-ray Diffraction Data Collected in Oscillation Mode"
Z.Otwinowski, W.Minor
Methods in Enzymology, Volume 276: Macromolecular Crystallography, part A, p307-326 (1997)
"HKL-3000: the integration of data reduction and structure solution - from diffraction images to an initial model in minutes"
W.Minor, M.Cymborowski, Z.Otwinowski, M.Chruszcz
Acta Cryst. D62: 859-866 (2006)

CCP4 suite
"Overview of the CCP4 suite and current developments"
M.D.Winn et al.
Acta. Cryst. D67, 235-242 (2011)

Refmac5
"REFMAC5 for the refinement of macromolecular crystal structures"
G.N.Murshudov, P.Skubak, A.A.Lebedev, N.S.Pannu, R.A.Steiner, R.A.Nicholls, M.D.Winn, F.Long and A.A.Vagin
Acta Cryst. D67, 355-367 (2011)

Coot
"Features and Development of Coot"
P.Emsley, B.Lohkamp, W.Scott, and K.Cowtan
Acta Cryst. D66, 486-501 (2010)

Data collection and refinement statistics for project **mes_1mos** crystal **crystal1**

model **/home/asia/epe-like-validation/RESULTS/HKL_rerefine/flat/1mos/structure_mr/build_model_1/hkl_import.pdb** vs
model **/home/asia/epe-like-validation/RESULTS/HKL_rerefine/flat/1mos/structure_mr/build_model_1/hkl_refine_3.pdb**

**Data collection**		
	**1MOS**	**Re-refinement**
Resolution (Å)	25.00 - 2.00 (2.03 - 2.00)	24.67 - 2.00
Wavelength (Å)		
Space group	H32	R32
a, b, c (Å)	143.90, 143.90, 172.80	143.90, 143.90, 172.80
α, β, γ (°)	90, 90, 120	90, 90, 120
Completeness (%)	98.7 (95.7)	100.0 (100.0)
Reflections used	45840	
<I> / <Sigma I>	3.6 (3.6)	
Redundancy	9.1 (2.6)	
Rmerge	0.058	
Rpim		
CC1/2 last shell		
Wilson B factor (Å²)	27.4	
**Refinement**		
Rwork / Rfree	/ 0.287	0.205 / 0.245
Resolution (Å)	12.00 - 2.00	24.67 - 2.00
Reflections all	45626	45840
Reflections for Rfree	, 5.0%	2338, 5.1%
Bond lengths rmsd (Å)	0.015	0.013
Bond angles rmsd (°)		1.79
Mean B value (Å²)	37	39
Number of protein atoms	2824	2824
Mean B value for protein atoms (Å²)	37	39
Number of water atoms (expected)	180 (315)	180 (315)
Mean B value for water atoms (Å²)	44	43
Number of ligand/ion atoms	44	44
Mean B value for ligand/ion atoms (Å²)	42	42
Clashscore	7.83	3.83
Clashscore percentile (100)	-1	-1
Rotamer outliers (<1%)	3.28	1.64
Ramachandran outliers (<0.2%)	0.00	0.00
Ramachandran favored (>98%)	98.36	98.90
Residues with bad bonds (<0%)	2.45	0.82
Residues with bad angles (<0.1%)	8.99	2.18
MolProbity score	1.82	1.33

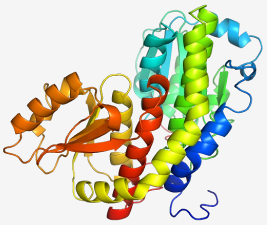

Map cc barchart:

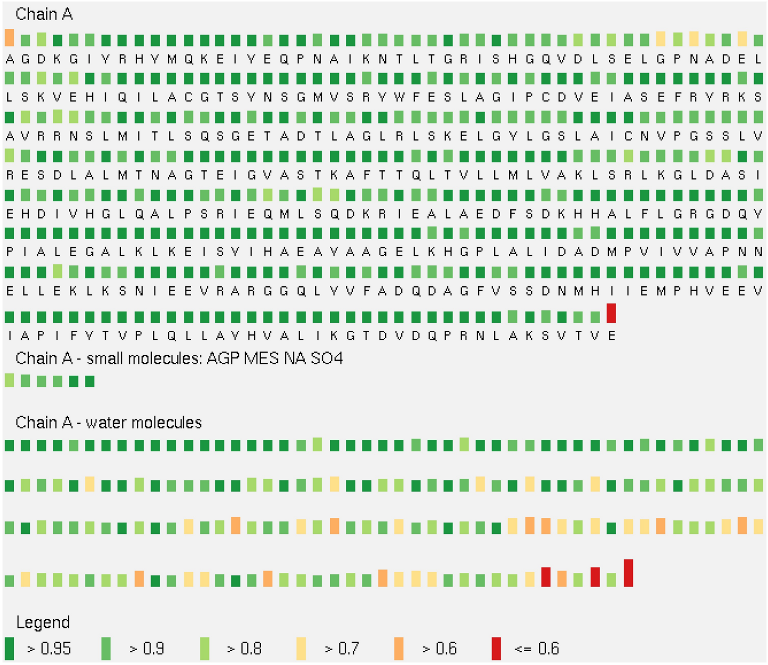

Small molecules with map:

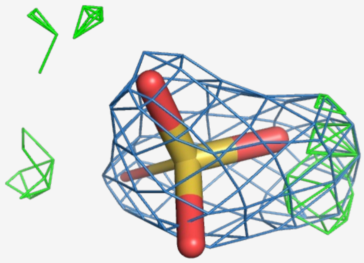

Small molecule 1: SULFATE ION (SO4) A 611 map cc 0.85

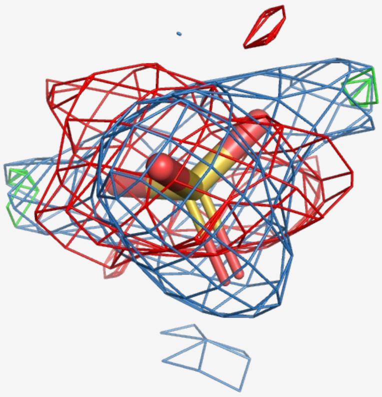

Small molecule 2: SULFATE ION (SO4) A 612 map cc 0.93

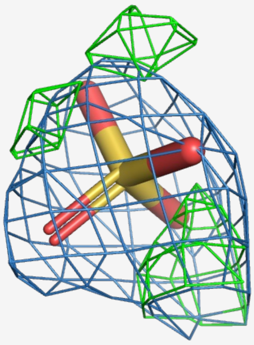

Small molecule 3: SULFATE ION (SO4) A 613 map cc 0.94

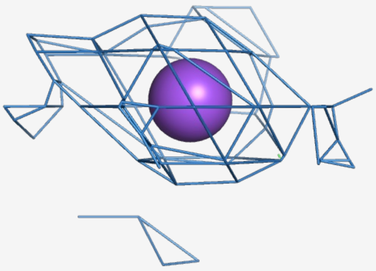

Small molecule 4: SODIUM ION (NA) A 614 map cc 0.95

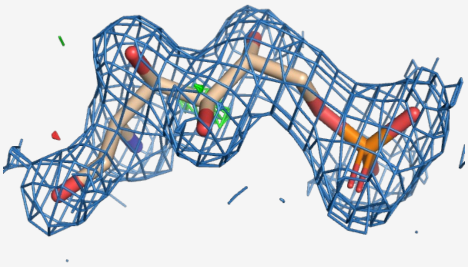

Small molecule 5: 2-DEOXY-2-AMINO GLUCITOL-6-PHOSPHATE (AGP) A 609 map cc 0.98

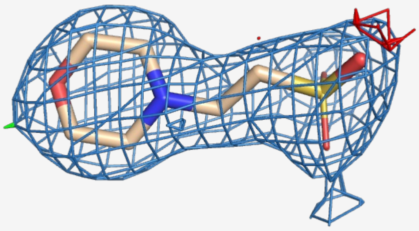

Small molecule 6: 2-(N-MORPHOLINO)-ETHANESULFONIC ACID (MES) A 610 map cc 0.97

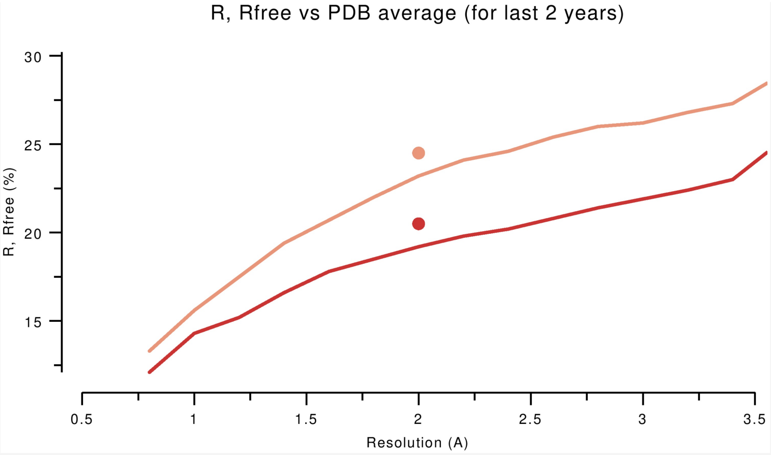

Credits:

HKL-3000
"Processing of X-ray Diffraction Data Collected in Oscillation Mode"
Z.Otwinowski, W.Minor
Methods in Enzymology, Volume 276: Macromolecular Crystallography, part A, p307-326 (1997)
"HKL-3000: the integration of data reduction and structure solution - from diffraction images to an initial model in minutes"
W.Minor, M.Cymborowski, Z.Otwinowski, M.Chruszcz
Acta Cryst. D62: 859-866 (2006)

CCP4 suite
"Overview of the CCP4 suite and current developments"
M.D.Winn et al.
Acta. Cryst. D67, 235-242 (2011)

Refmac5
"REFMAC5 for the refinement of macromolecular crystal structures"
G.N.Murshudov, P.Skubak, A.A.Lebedev, N.S.Pannu, R.A.Steiner, R.A.Nicholls, M.D.Winn, F.Long and A.A.Vagin
Acta Cryst. D67, 355-367 (2011)

Coot
"Features and Development of Coot"
P.Emsley, B.Lohkamp, W.Scott, and K.Cowtan
Acta Cryst. D66, 486-501 (2010)

Data collection and refinement statistics for project **mes_1mos** crystal **crystal1**

model **/home/asia/epe-like-validation/RESULTS/HKL_rerefine/flat/1mos/structure_mr/build_model_1/hkl_import.pdb** vs
model **/home/asia/epe-like-validation/RESULTS/HKL_rerefine/flat/1mos/structure_mr/build_model_1/hkl_refine_4.pdb**

**Data collection**		
	**1MOS**	**Re-refinement**
Resolution (Å)	25.00 - 2.00 (2.03 - 2.00)	24.67 - 2.00
Wavelength (Å)		
Space group	H32	R32
a, b, c (Å)	143.90, 143.90, 172.80	143.90, 143.90, 172.80
α, β, γ (°)	90, 90, 120	90, 90, 120
Completeness (%)	98.7 (95.7)	100.0 (100.0)
Reflections used	45840	
<I> / <Sigma I>	3.6 (3.6)	
Redundancy	9.1 (2.6)	
Rmerge	0.058	
Rpim		
CC1/2 last shell		
Wilson B factor (Å²)	27.4	
**Refinement**		
Rwork / Rfree	/ 0.287	0.205 / 0.246
Resolution (Å)	12.00 - 2.00	24.67 - 2.00
Reflections all	45626	45840
Reflections for Rfree	, 5.0%	2338, 5.1%
Bond lengths rmsd (Å)	0.015	0.013
Bond angles rmsd (°)		1.79
Mean B value (Å²)	37	39
Number of protein atoms	2824	2824
Mean B value for protein atoms (Å²)	37	39
Number of water atoms (expected)	180 (315)	180 (315)
Mean B value for water atoms (Å²)	44	43
Number of ligand/ion atoms	44	44
Mean B value for ligand/ion atoms (Å²)	42	42
Clashscore	7.83	3.13
Clashscore percentile (100)	-1	-1
Rotamer outliers (<1%)	3.28	1.64
Ramachandran outliers (<0.2%)	0.00	0.00
Ramachandran favored (>98%)	98.36	98.90
Residues with bad bonds (<0%)	2.45	0.54
Residues with bad angles (<0.1%)	8.99	1.63
MolProbity score	1.82	1.27

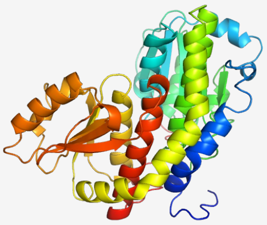

Map cc barchart:

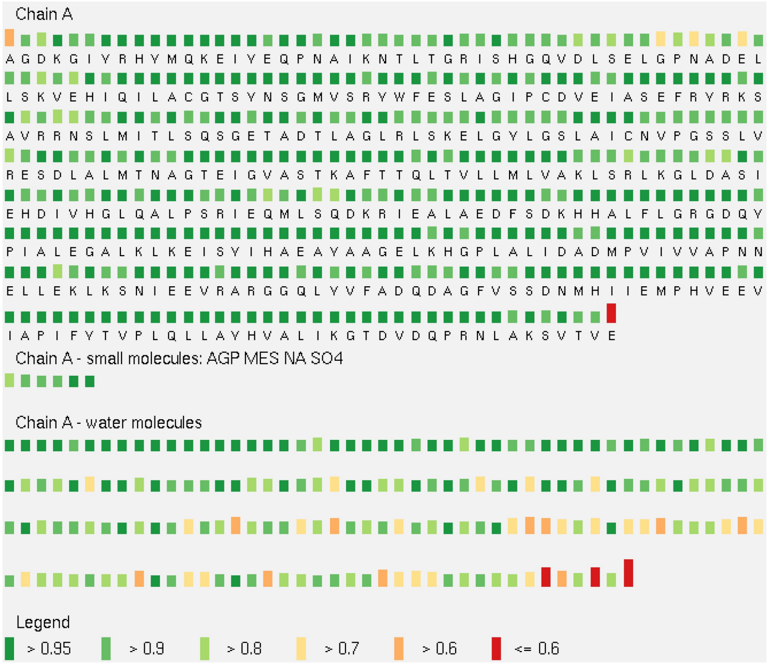

Small molecules with map:

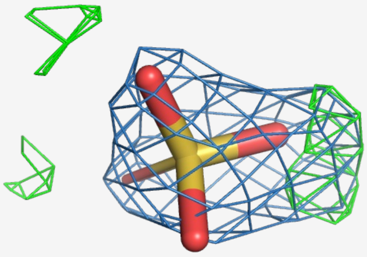

Small molecule 1: SULFATE ION (SO4) A 611 map cc 0.85

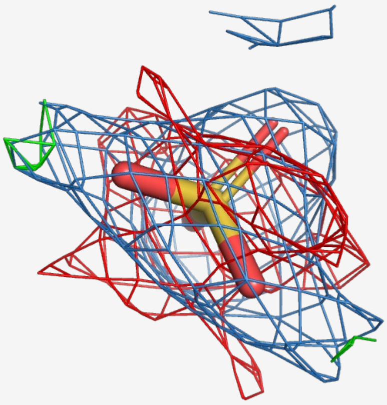

Small molecule 2: SULFATE ION (SO4) A 612 map cc 0.93

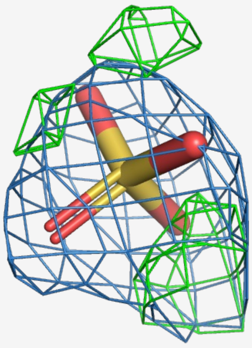

Small molecule 3: SULFATE ION (SO4) A 613 map cc 0.94

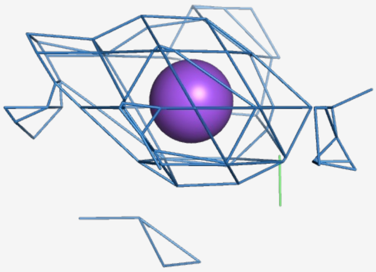

Small molecule 4: SODIUM ION (NA) A 614 map cc 0.95

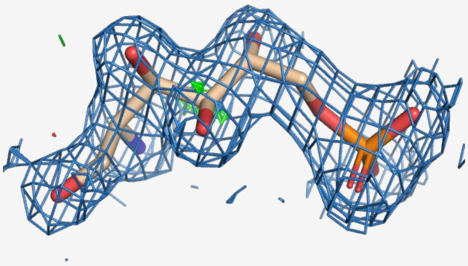

Small molecule 5: 2-DEOXY-2-AMINO GLUCITOL-6-PHOSPHATE (AGP) A 609 map cc 0.98

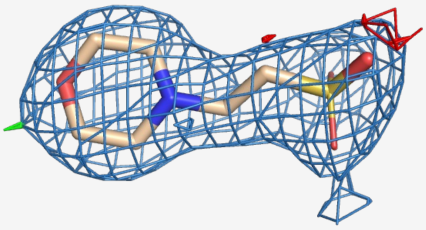

Small molecule 6: 2-(N-MORPHOLINO)-ETHANESULFONIC ACID (MES) A 610 map cc 0.97

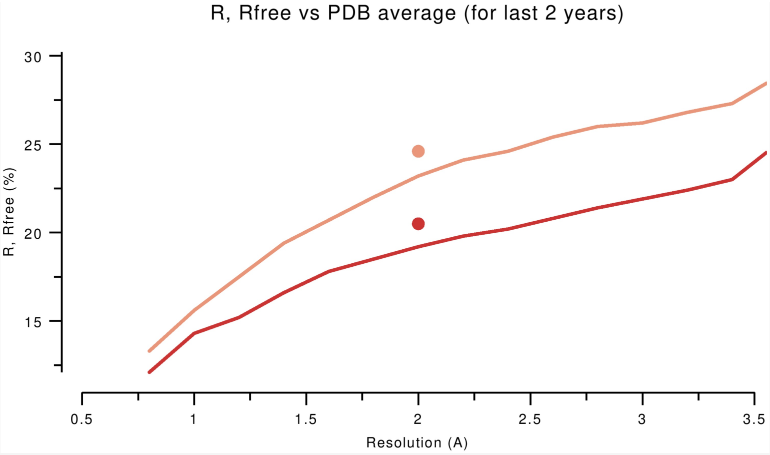

Credits:

HKL-3000
"Processing of X-ray Diffraction Data Collected in Oscillation Mode"
Z.Otwinowski, W.Minor
Methods in Enzymology, Volume 276: Macromolecular Crystallography, part A, p307-326 (1997)
"HKL-3000: the integration of data reduction and structure solution - from diffraction images to an initial model in minutes"
W.Minor, M.Cymborowski, Z.Otwinowski, M.Chruszcz
Acta Cryst. D62: 859-866 (2006)

CCP4 suite
"Overview of the CCP4 suite and current developments"
M.D.Winn et al.
Acta. Cryst. D67, 235-242 (2011)

Refmac5
"REFMAC5 for the refinement of macromolecular crystal structures"
G.N.Murshudov, P.Skubak, A.A.Lebedev, N.S.Pannu, R.A.Steiner, R.A.Nicholls, M.D.Winn, F.Long and A.A.Vagin
Acta Cryst. D67, 355-367 (2011)

Coot
"Features and Development of Coot"
P.Emsley, B.Lohkamp, W.Scott, and K.Cowtan
Acta Cryst. D66, 486-501 (2010)

Data collection and refinement statistics for project **mes_1moq** crystal **crystal1**

model **/home/asia/epe-like-validation/RESULTS/HKL_rerefine/flat/1moq/structure_mr/build_model_1/hkl_import.pdb** vs
model **/home/asia/epe-like-validation/RESULTS/HKL_rerefine/flat/1moq/structure_mr/build_model_1/hkl_refine_3.pdb**

**Data collection**		
	**1MOQ**	**Re-refinement**
Resolution (Å)	30.00 - 1.57 (1.60 - 1.57)	28.17 - 1.57
Wavelength (Å)		
Space group	H32	R32
a, b, c (Å)	143.70, 143.70, 173.60	143.70, 143.70, 173.60
α, β, γ (°)	90, 90, 120	90, 90, 120
Completeness (%)	99.5 (96.2)	100.0 (100.0)
Reflections used	95543	
<I> / <Sigma I>	39.2 (5.7)	
Redundancy	6.5 (3.3)	
Rmerge	0.040	
Rpim		
CC1/2 last shell		
Wilson B factor (Å²)	18.0	
**Refinement**		
Rwork / Rfree	0.185 /	0.133 / 0.140
Resolution (Å)	10.00 - 1.57	28.19 - 2.50
Reflections all	94943	23962
Reflections for Rfree	, %	1180, 4.9%
Bond lengths rmsd (Å)	0.014	0.019
Bond angles rmsd (°)		2.65
Mean B value (Å²)	25	24
Number of protein atoms	2825	2825
Mean B value for protein atoms (Å²)	22	22
Number of water atoms (expected)	416 (445)	416 (445)
Mean B value for water atoms (Å²)	40	37
Number of saccharide atoms	16	16
Mean B value for saccharide atoms (Å²)	14	13
Number of ligand/ion atoms	46	46
Mean B value for ligand/ion atoms (Å²)	38	34
Clashscore	5.71	5.36
Clashscore percentile (100)	-1	-1
Rotamer outliers (<1%)	0.98	0.98
Ramachandran outliers (<0.2%)	0.00	0.00
Ramachandran favored (>98%)	98.35	98.35
Residues with bad bonds (<0%)	0.55	0.27
Residues with bad angles (<0.1%)	4.78	4.37
MolProbity score	1.31	1.29

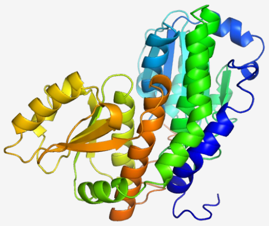

Map cc barchart:

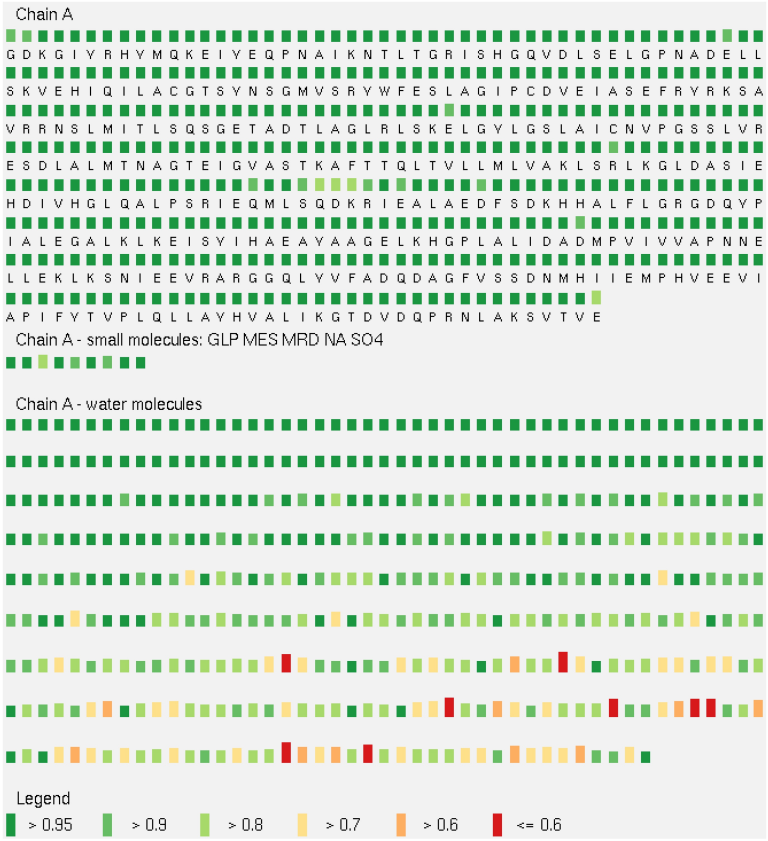

Small molecules with map:

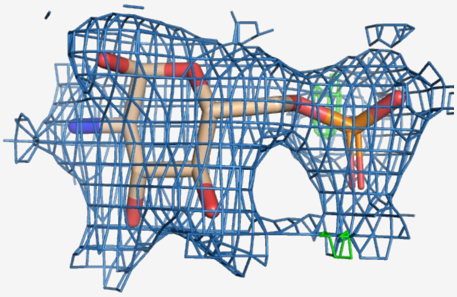

Small molecule 1: GLUCOSAMINE 6-PHOSPHATE (GLP) A 609 map cc 0.99

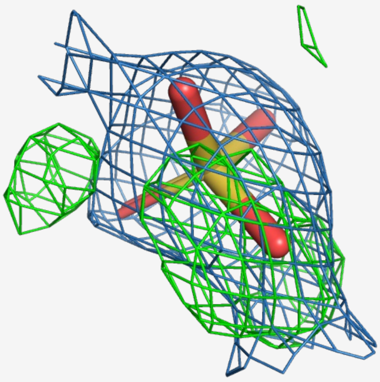

Small molecule 2: SULFATE ION (SO4) A 612 map cc 0.97

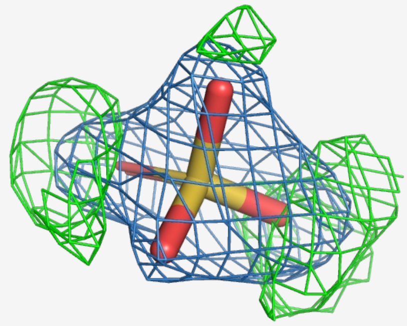

Small molecule 3: SULFATE ION (SO4) A 613 map cc 0.86

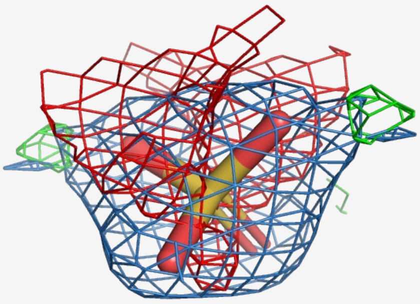

Small molecule 4: SULFATE ION (SO4) A 614 map cc 0.98

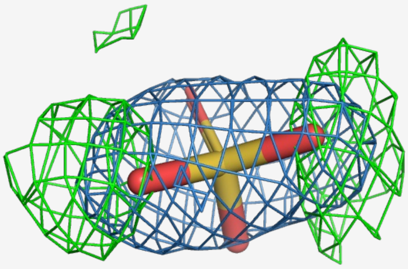

Small molecule 5: SULFATE ION (SO4) A 615 map cc 0.94

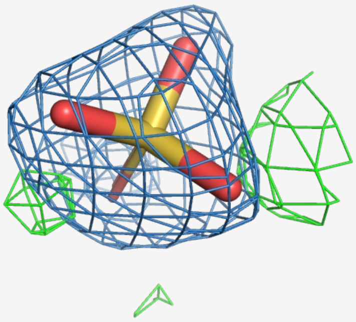

Small molecule 6: SULFATE ION (SO4) A 616 map cc 0.97

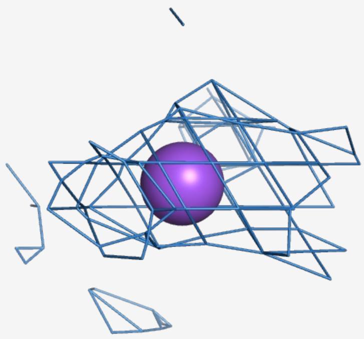

Small molecule 7: SODIUM ION (NA) A 617 map cc 0.91

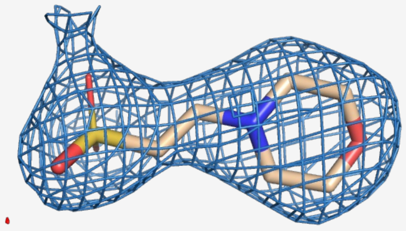

Small molecule 8: 2-(N-MORPHOLINO)-ETHANESULFONIC ACID (MES) A 610 map cc 0.99

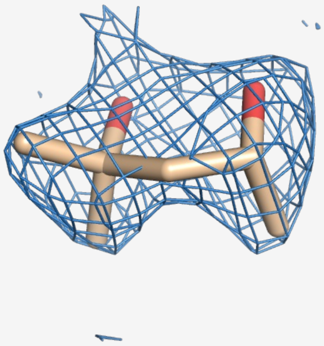

Small molecule 9: (4R)-2-METHYLPENTANE-2,4-DIOL (MRD) A 611 map cc 0.98

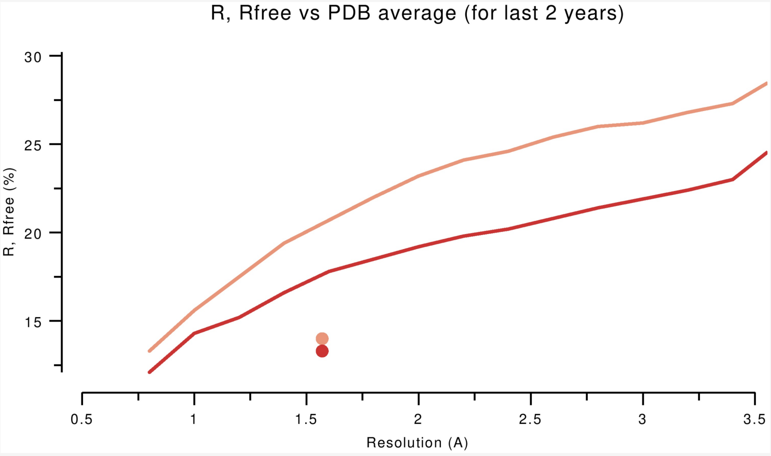

Credits:

HKL-3000

"Processing of X-ray Diffraction Data Collected in Oscillation Mode"
Z.Otwinowski, W.Minor
Methods in Enzymology, Volume 276: Macromolecular Crystallography, part A, p307-326 (1997)
"HKL-3000: the integration of data reduction and structure solution - from diffraction images to an initial model in minutes"
W.Minor, M.Cymborowski, Z.Otwinowski, M.Chruszcz
Acta Cryst. D62: 859-866 (2006)

CCP4 suite
"Overview of the CCP4 suite and current developments"
M.D.Winn et al.
Acta. Cryst. D67, 235-242 (2011)

Refmac5
"REFMAC5 for the refinement of macromolecular crystal structures"
G.N.Murshudov, P.Skubak, A.A.Lebedev, N.S.Pannu, R.A.Steiner, R.A.Nicholls, M.D.Winn, F.Long and A.A.Vagin
Acta Cryst. D67, 355-367 (2011)

Coot
"Features and Development of Coot"
P.Emsley, B.Lohkamp, W.Scott, and K.Cowtan
Acta Cryst. D66, 486-501 (2010)

Data collection and refinement statistics for project **mes_1moq** crystal **crystal1**

model **/home/asia/epe-like-validation/RESULTS/HKL_rerefine/flat/1moq/structure_mr/build_model_1/hkl_import.pdb** vs
model **/home/asia/epe-like-validation/RESULTS/HKL_rerefine/flat/1moq/structure_mr/build_model_1/hkl_refine_4.pdb**

**Data collection**		
	**1MOQ**	**Re-refinement**
Resolution (Å)	30.00 - 1.57 (1.60 - 1.57)	28.17 - 1.57
Wavelength (Å)		
Space group	H32	R32
a, b, c (Å)	143.70, 143.70, 173.60	143.70, 143.70, 173.60
α, β, γ (°)	90, 90, 120	90, 90, 120
Completeness (%)	99.5 (96.2)	100.0 (100.0)
Reflections used	95543	
<I> / <Sigma I>	39.2 (5.7)	
Redundancy	6.5 (3.3)	
Rmerge	0.040	
Rpim		
CC1/2 last shell		
Wilson B factor (Å^2)	18.0	
**Refinement**		
Rwork / Rfree	0.185 /	0.121 / 0.156
Resolution (Å)	10.00 - 1.57	28.19 - 2.50
Reflections all	94943	23962
Reflections for Rfree	, %	1180, 4.9%
Bond lengths rmsd (Å)	0.014	0.014
Bond angles rmsd (°)		1.83
Mean B value (Å^2)	25	24
Number of protein atoms	2825	2825
Mean B value for protein atoms (Å^2)	22	23
Number of water atoms (expected)	416 (445)	416 (445)
Mean B value for water atoms (Å^2)	40	36
Number of saccharide atoms	16	16
Mean B value for saccharide atoms (Å^2)	14	14
Number of ligand/ion atoms	46	46
Mean B value for ligand/ion atoms (Å^2)	38	35
Clashscore	5.71	3.80
Clashscore percentile (100)	-1	-1
Rotamer outliers (<1%)	0.98	1.30
Ramachandran outliers (<0.2%)	0.00	0.00
Ramachandran favored (>98%)	98.35	98.63
Residues with bad bonds (<0%)	0.55	0.96
Residues with bad angles (<0.1%)	4.78	1.23
MolProbity score	1.31	1.26

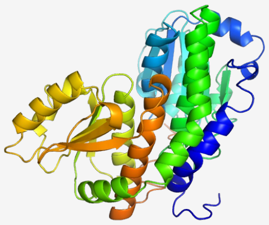

Map cc barchart:

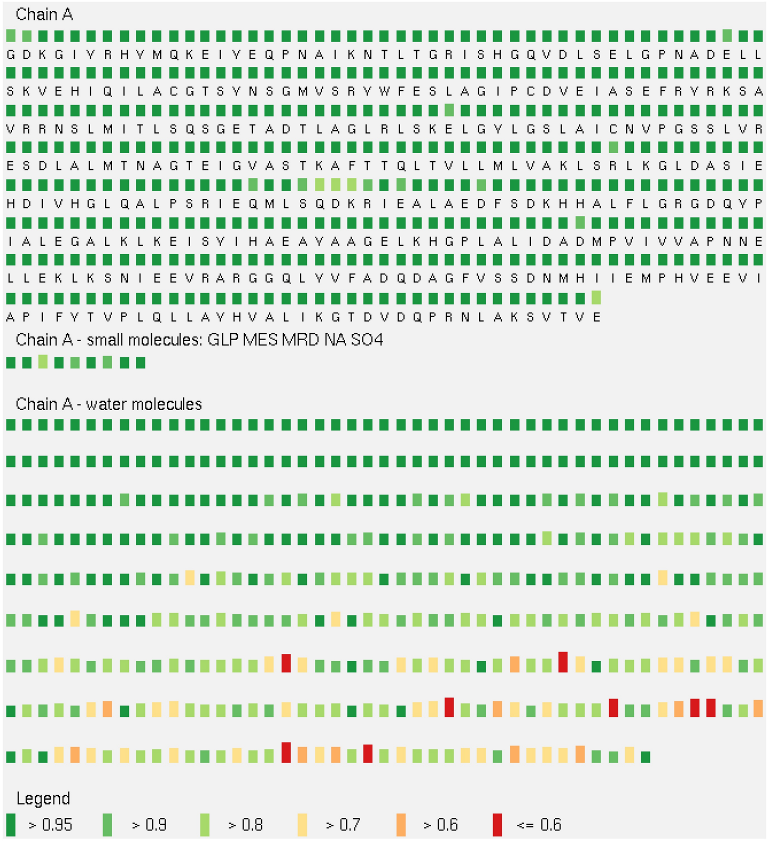

Small molecules with map:

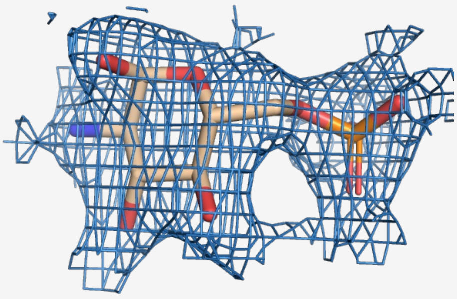

Small molecule 1: GLUCOSAMINE 6-PHOSPHATE (GLP) A 609 map cc 0.99

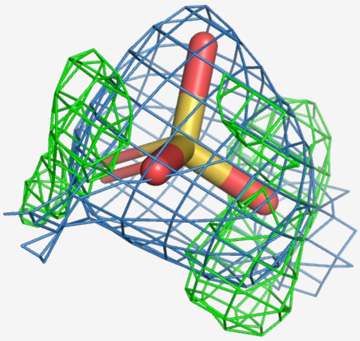

Small molecule 2: SULFATE ION (SO4) A 612 map cc 0.97

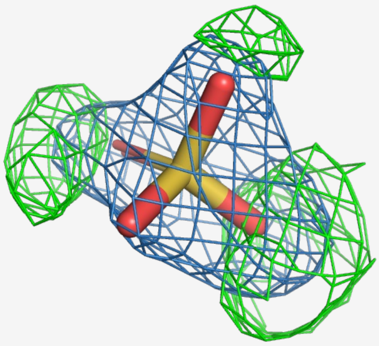

Small molecule 3: SULFATE ION (SO4) A 613 map cc 0.86

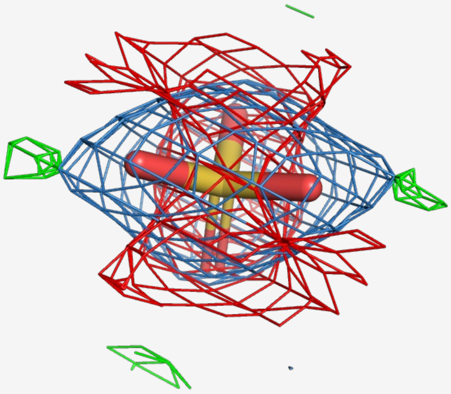

Small molecule 4: SULFATE ION (SO4) A 614 map cc 0.98

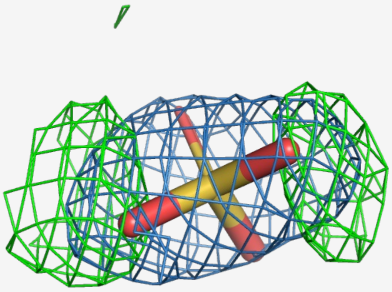

Small molecule 5: SULFATE ION (SO4) A 615 map cc 0.94

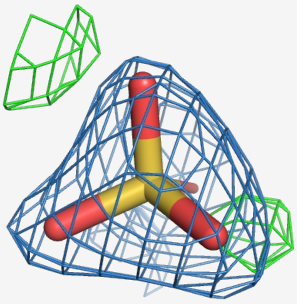

Small molecule 6: SULFATE ION (SO4) A 616 map cc 0.97

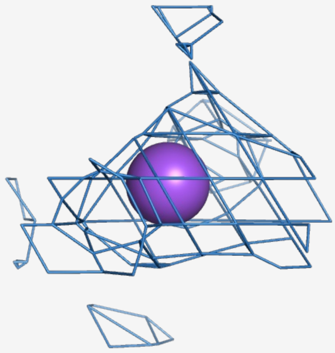

Small molecule 7: SODIUM ION (NA) A 617 map cc 0.91

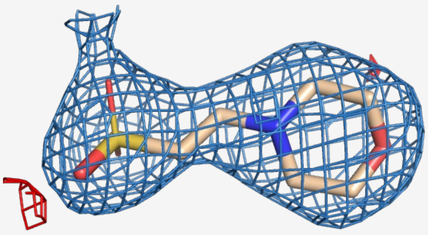

Small molecule 8: 2-(N-MORPHOLINO)-ETHANESULFONIC ACID (MES) A 610 map cc 0.99

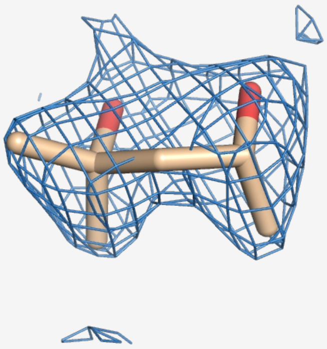

Small molecule 9: (4R)-2-METHYLPENTANE-2,4-DIOL (MRD) A 611 map cc 0.98

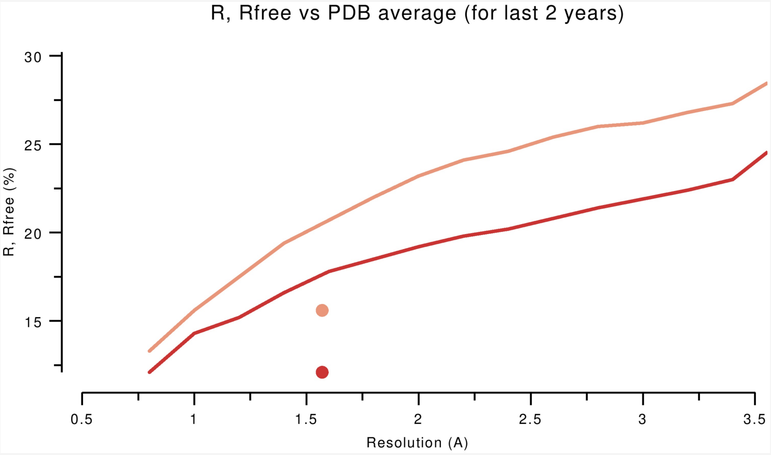

Credits:

HKL-3000

"Processing of X-ray Diffraction Data Collected in Oscillation Mode"
Z.Otwinowski, W.Minor
Methods in Enzymology, Volume 276: Macromolecular Crystallography, part A, p307-326 (1997)
"HKL-3000: the integration of data reduction and structure solution - from diffraction images to an initial model in minutes"
W.Minor, M.Cymborowski, Z.Otwinowski, M.Chruszcz
Acta Cryst. D62: 859-866 (2006)

CCP4 suite
"Overview of the CCP4 suite and current developments"
M.D.Winn et al.
Acta. Cryst. D67, 235-242 (2011)

Refmac5
"REFMAC5 for the refinement of macromolecular crystal structures"
G.N.Murshudov, P.Skubak, A.A.Lebedev, N.S.Pannu, R.A.Steiner, R.A.Nicholls, M.D.Winn, F.Long and A.A.Vagin
Acta Cryst. D67, 355-367 (2011)

Coot
"Features and Development of Coot"
P.Emsley, B.Lohkamp, W.Scott, and K.Cowtan
Acta Cryst. D66, 486-501 (2010)
